# The iHealth-T2D study, prevention of type 2 diabetes amongst South Asians with central obesity and prediabetes: study protocol for a randomised controlled trial

**DOI:** 10.1186/s13063-021-05803-7

**Published:** 2021-12-18

**Authors:** Anuradhani Kasturiratne, Khadija I. Khawaja, Sajjad Ahmad, Samreen Siddiqui, Khurram Shahzad, Lathika K. Athauda, Ranil Jayawardena, Sara Mahmood, Mirthe Muilwijk, Tayyaba Batool, Saira Burney, Matthew Glover, Saranya Palaniswamy, Vodathi Bamunuarachchi, Manju Panda, Suren Madawanarachchi, Baldeesh Rai, Iqra Sattar, Wnurinham Silva, Swati Waghdhare, Marjo-Riitta Jarvelin, Ravindra P. Rannan-Eliya, Heather M. Gage, Irene G. M. van Valkengoed, Jonathan Valabhji, Gary S. Frost, Marie Loh, Ananda R. Wickremasinghe, Jaspal S. Kooner, Prasad Katulanda, Sujeet Jha, John C. Chambers

**Affiliations:** 1 Department of Public Health, Faculty of Medicine, University of Kelaniya, PO Box 06, Thalagolla Road, Ragama, 11010 Sri Lanka; 2 Department of Endocrinology & Metabolism, Services Institute of Medical Sciences, Services Hospital, Ghaus ul Azam, Jail Road, Lahore, 54700 Pakistan; 3 Punjab Institute of Cardiology, Jail Road, Lahore, Pakistan; 4 Institute of Endocrinology, Diabetes & Metabolism, Max Super Speciality Hospital, 2, Press Enclave Road, Saket, New Delhi, 110017 India; 5 Department of Physiology, Faculty of Medicine, University of Colombo, Colombo, Sri Lanka; 6 Department of Public and Occupational Health, Amsterdam Public Health research institute, Amsterdam UMC, University of Amsterdam, Meibergdreef 9, 1105 AZ Amsterdam, The Netherlands; 7 Department of Endocrinology & Metabolism, Diabetes Management Centre, Services Hospital, Ghaus-ul-Azam, Jail Road, Lahore, 540000 Pakistan; 8 School of Biosciences and Medicine, Faculty of Health and Medical Sciences, University of Surrey, Daphne Jackson Road, Guildford, GU2 7WG Surrey, England; 9 School of Public Health, Epidemiology and Biostatistics, Imperial College London, St Mary’s Campus, Norfolk Place, London, W2 1PG UK; 10 Center for Life Course Health Research, Faculty of Medicine, University of Oulu, Oulu, Finland; 11 Diabetes Research Unit, Faculty of Medicine, University of Colombo, Colombo, Sri Lanka; 12 Department of Life Sciences, College of Health and Life Sciences, Brunel University London, Kingston Lane, Uxbridge, Middlesex, UB8 3PH UK; 13 Unit of Primary Care, Oulu University Hospital, Oulu, Finland; 14 Institute for Health Policy, 72 Park Street, Colombo, 00200 Sri Lanka; 15 Surrey Health Economics Centre, Department of Clinical and Experimental Medicine, University of Surrey, Leggett Building, Daphne Jackson Road, Guildford, GU2 7WG Surrey, England; 16 Department of Diabetes and Endocrinology, 1st Floor Mint Wing, St Mary’s Hospital, Imperial College Healthcare NHS Trust, London, W2 1NY UK; 17 6th Floor Commonwealth Building, Faculty of Medicine, Imperial College London, Hammersmith Campus, Ducane Road, London, W12 ONN UK; 18 Lee Kong Chian School of Medicine, Nanyang Technological University, Singapore, 308232 Singapore; 19 National Heart and Lung Institute, Imperial College London, Hammersmith Hopsital Campus, Ducane Road, London, W12 ONN UK; 20 Uxbridge Road, Southall, Middlesex, UB1 3HW UK; 21 Department of Clinical Medicine, Faculty of Medicine, University of Colombo, Colombo, Sri Lanka

## Abstract

**Background:**

People from South Asia are at increased risk of type 2 diabetes (T2D). There is an urgent need to develop approaches for the prevention of T2D in South Asians that are cost-effective, generalisable and scalable across settings.

**Hypothesis:**

Compared to usual care, the risk of T2D can be reduced amongst South Asians with central obesity or raised HbA1c, through a 12-month lifestyle modification programme delivered by community health workers.

**Design:**

Cluster randomised clinical trial (1:1 allocation to intervention or usual care), carried out in India, Pakistan, Sri Lanka and the UK, with 30 sites per country (120 sites total). Target recruitment 3600 (30 participants per site) with annual follow-up for 3 years.

**Entry criteria:**

South Asian, men or women, age 40–70 years with (i) central obesity (waist circumference ≥ 100 cm in India and Pakistan; ≥90 cm in Sri Lanka) and/or (ii) prediabetes (HbA1c 6.0–6.4% inclusive). Exclusion criteria: known type 1 or 2 diabetes, normal or underweight (body mass index < 22 kg/m^2^); pregnant or planning pregnancy; unstable residence or planning to leave the area; and serious illness.

**Endpoints:**

The primary endpoint is new-onset T2D at 3 years, defined as (i) HbA1c ≥ 6.5% or (ii) physician diagnosis and on treatment for T2D. Secondary endpoints at 1 and 3 years are the following: (i) physical measures: waist circumference, weight and blood pressure; (ii) lifestyle measures: smoking status, alcohol intake, physical activity and dietary intake; (iii) biochemical measures: fasting glucose, insulin and lipids (total and HDL cholesterol, triglycerides); and (iv) treatment compliance.

**Intervention:**

Lifestyle intervention (60 sites) or usual care (60 sites). Lifestyle intervention was delivered by a trained community health worker over 12 months (5 one-one sessions, 4 group sessions, 13 telephone sessions) with the goal of the participants achieving a 7% reduction in body mass index and a 10-cm reduction in waist circumference through (i) improved diet and (ii) increased physical activity. Usual care comprised a single 30-min session of lifestyle modification advice from the community health worker.

**Results:**

We screened 33,212 people for inclusion into the study. We identified 10,930 people who met study entry criteria, amongst whom 3682 agreed to take part in the intervention. Study participants are 49.2% female and aged 52.8 (*SD* 8.2) years. Clinical characteristics are well balanced between intervention and usual care sites. More than 90% of follow-up visits are scheduled to be complete in December 2020. Based on the follow-up to end 2019, the observed incidence of T2D in the study population is in line with expectations (6.1% per annum).

**Conclusion:**

The iHealth-T2D study will advance understanding of strategies for the prevention of diabetes amongst South Asians, use approaches for screening and intervention that are adapted for low-resource settings. Our study will thus inform the implementation of strategies for improving the health and well-being of this major global ethnic group.

**IRB approval:**

16/WM/0171

**Trial registration:**

EudraCT 2016-001350-18. Registered on 14 April 2016. ClinicalTrials.govNCT02949739. Registered on 31 October 2016, First posted on 31/10/2016.

**Supplementary Information:**

The online version contains supplementary material available at 10.1186/s13063-021-05803-7.

## Background

South Asians, who represent one-quarter of the world’s population, are at high risk of type 2 diabetes (T2D) [1]. India alone has ~ 77 million people with T2D, the second highest number in the world [2]. T2D prevalence is currently ~ 5% in rural India and ~ 11% in urban India [3]. Similar patterns are observed amongst South Asians in Pakistan, Bangladesh and Sri Lanka [4]. The adverse consequences of diabetes are further magnified by the earlier age of onset of T2D and by the low availability and financial barriers to obtaining high-quality care in South Asia [5]. South Asians living in Europe have a 2–4 times higher risk of T2D compared to Europeans, for reasons that remain to be determined [5,6]. The high burden of T2D in South Asians thus represents a major public health challenge.

T2D is a preventable disorder. Amongst overweight and obese Europeans with impaired glucose tolerance, a programme of intensive lifestyle modification (increased physical activity, dietary change and weight loss) is associated with an ~ 60% relative risk reduction in the incidence of T2D [7]. The benefits are maintained for at least 10 years after the intervention has stopped [8]. Meta-analysis of published studies supports the potential for lifestyle intervention to reduce the risk of T2D amongst South Asians [9]. However, completed studies have been predominantly carried out in urban settings, and with under-representation of women, limiting the generalisability of findings. Furthermore, the trials have typically relied on the identification of high-risk individuals using the oral glucose tolerance test and on delivery of the lifestyle modification by trained healthcare teams, approaches that are resource intensive and difficult to scale up. As a result, interventions for the prevention of T2D are not routinely available to South Asian communities, especially in LMIC settings.

Delivery of health promotion for the prevention of chronic disease has traditionally been the responsibility of physicians and allied health professionals. However, recent research supports the view that community health workers (CHWs) can make an effective contribution to the prevention and early diagnosis of chronic disease and deliver improved outcomes compared to usual care [10]. Studies in rural India show that CHWs can provide health education and support management of hypertensive individuals [11]. A comprehensive multicomponent intervention in Bangladesh, Pakistan and Sri Lanka showed that trained CHWs working in partnership with public healthcare systems improved blood pressure control amongst adults with hypertension [12]. Whether CHWs can deliver lifestyle modification for prevention of T2D in South Asians that is effective, cost-effective and potentially scalable remains to be determined.

We therefore established the iHealth-T2D study, as a large-scale cluster randomised clinical trial, to test the hypothesis that the risk of T2D can be reduced amongst South Asians with central obesity or raised HbA1c, through a 12-month lifestyle modification programme delivered by CHWs.

## Preparatory work

### Development of a lifestyle intervention programme for delivery by CHWs

Our intervention programme was developed by a team with expertise in nutrition, dietetics, epidemiology and public health from the study centres in South Asia and the UK and informed by the design of the Diabetes Prevention Programme [7]. The primary objective was to achieve a 7% reduction in body mass index and a 10-cm reduction in waist circumference through (i) improved diet and (ii) increased physical activity. *Improved diet* comparised a 500-kcal energy reduction in energy intake for the overweight and obese. The profile of the diet was based on health eating guidelines, which are common across non-communicable disease prevention programmes, and included attention to increasing fruit and vegetable consumption, decreasing sugar intake, reducing alcohol consumption (where appropriate) and portion sizes, and identifying cooking substitutions to reduce fat. *Increased physical activity* includes finding enjoyable physical activities to pursue regularly and incorporating physical activity into daily routines. The target was to achieve 150 min of moderate physical activity every week.

Core design elements of the programme included (1) goal-based behavioural intervention, with goals set in partnership with the individual on an ongoing basis; (2) personalised support for the participant during delivery of the intervention; (3) frequent contact to help participants achieve and maintain the weight and physical activity goals; and (4) “Toolbox” strategies to tailor the intervention to the individual participant. Components specific to the current effort included (1) use of CHWs, supported by local experts for delivery of lifestyle intervention; (2) cultural adaptation included the development of meal plans and behaviour change advice suitable for South Asian communities; (3) incorporating a family-based approach to the intervention, intervention where one member of the patients family attended the education sessions, given the evidence for improved response to lifestyle advice when family members are included in the education [13,14]; and (4) making use of telephone and group sessions to bring peer support, to improve accessibility of the intervention and to maximise the efficiency of delivery. Relevant stakeholders (potential participants, CHWs and other healthcare providers) were involved in the design and adaptation process.

The final lifestyle modification programme comprised 22 contact sessions over 12 months. The 22 sessions comprised five one-one meetings (duration up to 60 min), four group sessions (up to 90 min) and 13 telephone sessions (up to 15 min). The use of group and telephone contact sessions may improve participation, engagement and outcomes of intervention programmes compared to wholly face-face strategies [15–17]. Session content and timing are summarised in Table 1 and in the Supplementary Online Materials. The intervention programme was supported by written materials for participants, translated into local languages. This participant Handbook contained educational information about the importance of obesity and diabetes, the potential benefits of lifestyle modification, and the key components of a healthy lifestyle (focused on improved and increased physical activity). The aim of the Handbook was to guide participants through the lifestyle modification sessions and support self-monitoring of their lifestyle behaviours, goal setting, goal review and waist circumference.

**Table 1:** Timetable for the lifestyle intervention programme delivered by the community health workers. Overall, there were four one to one sessions (121), five group sessions (group) and 13 telephone support sessions (phone)

Session number	Week	Session type	Duration (min)	Weight, waist	Food diary	Activity scoring	Education	Goal setting	Reinforce and support
1	1	121	60	X	X	X	X	X	
2	2	Phone	15						X
3	3	121	60	X	X		X	X	
4	5	Phone	15						X
5	7	Group	90	X			X	X	
6	9	Phone	15						X
7	11	Group	90	X			X	X	
8	13	Phone	15						X
9	15	Group	90	X			X	X	
10	17	Phone	15						X
11	19	121	60	X	X	X	X	X	
12	21	Phone	15						X
13	23	Phone	15						X
14	25	Group	90	X			X	X	
15	28	Phone	15						X
16	31	Phone	15						X
17	34	Phone	15						X
18	37	Group	90	X			X	X	
19	40	Phone	15						X
20	43	Phone	15						X
21	46	Phone	15						X
22	49	121	60	X	X		X	X	

### Identification and training of CHWs

CHWs were graduates with a biological science background and were recruited from amongst the local community in which they worked, to ensure natural cultural awareness. Training of CHWs was provided by local experts in diabetes, nutrition and exercise, according to the principles and practices described in the intervention protocols. Training covered theoretical knowledge as well as practical hands-on learning in communication and delivery of the intervention and lasted 2–3 weeks. The study handbook summarised the protocols to be followed and provided toolkits for promoting a healthy diet and physical activity. All trainee CHWs were assessed for knowledge, skills and attitude by the trainers prior to commencing intervention. Each CHW was expected to initiate an intervention for 80–100 new participants per year, followed by delivery of the complete programme over the 12 months.

### Identification of South Asians at increased risk of diabetes

The glucose tolerance test has been the mainstay for the identification of future risk for T2D. However, this is a resource-intensive approach that is not well-suited for implementation at a population scale. To address this limitation, we used our longitudinal studies of South Asian populations to identify non-laboratory and laboratory markers of risk that may be better suited to the identification of susceptible individuals from the general population. We compared a range of clinically relevant, routinely available measures for prediction of new-onset T2D, amongst the 17,000 South Asian men and women aged 35 to 75 years, under long-term follow-up in the London Life Science Prospective Population (LOLIPOP) study [6]. Our results show that waist circumference is a strong predictor of incident T2D in South Asians, which offers better discrimination than body mass index or waist-hip ratio (Table 2). Waist circumference is a simple, readily available clinical measure of adiposity that is well-suited for use as a tool for community-wide risk stratification, especially in low-middle-income countries. Our results also identify HbA1c as a highly predictive biochemical tool for the identification of high-risk South Asians [6] that achieves similar discrimination for T2D to fasting glucose (Table 2). HbA1c has the additional advantage of being a non-fasting assay, which is simpler and cheaper to administer than an oral glucose tolerance test. HbA1c is potentially well-suited for community-wide and opportunistic screening. These observations provide the rationale to investigate the clinical utility of HbA1c and waist circumference for the identification of South Asians at high risk of T2D, in the present study.

**Table 2:** Risk factors for incident T2D (5 years) amongst South Asians in the LOLIPOP study [6]

Risk factor	Prevalence	T2D incidence	Sensitivity	RR for T2D
*Biochemical*				
Fasting glucose ≥ 6.0 mmol/L (WHO)	5.0%	55.6%	19.3%	6.66 (4.89–9.07)
HbA1c ≥ 6.0% (WHO)	16.3%	68.4%	35.0%	13.9 (10.4–18.6)
*Anthropometric*				
Body mass index > 28 kg/m^2^ (WHO)	17.0%	30.1%	32.6%	2.35 (1.96–2.82)
Waist-hip ratio (M > 1.0, F > 0.9)	28.0%	27.1%	46.2%	2.21 (1.88–2.60)
Waist: M or F ≥ 100 cm	28.9%	31.3%	49.6%	2.56 (2.21–3.06)

## Methods

### Study design

iHealth-T2D is a multi-centre, cluster randomised clinical trial to evaluate intensive lifestyle modification delivered by CHWs for prevention of T2D, compared to usual care. Study entry criteria were South Asian, men or women, age 40–70 years with (i) central obesity (waist circumference ≥ 100 cm in India and Pakistan; ≥90 cm in Sri Lanka) and/or (ii) prediabetes (HbA1c 6.0–6.4% inclusive). Exclusion criteria were known type 1 or 2 diabetes, normal or underweight (body mass index < 22 kg/m^2^); pregnant or planning pregnancy; unstable residence or planning to leave the area; and serious illness. We aimed to recruit 3600 participants from 120 locations across 4 countries (India, Pakistan, Sri Lanka and the UK) comprising 900 participants and 30 sites per country. Participants were cluster randomised based on study site to receive either intensive lifestyle modification (*N* = 60 sites) or usual care (*N* = 60 sites). Participants will be followed at 12, 24 and 36 months after enrolment. The primary endpoint is the incidence of T2D at 3 years. The study was approved by an Institutional Review Board (IRB) in each participating country (ref 16/WM/0171) and was registered on the EudraCT database (2016-001350-18). Primary funding for the study was from the European Commission (award 643774). Imperial College London acted as the sponsor for the study. The complete study protocol and study materials are available on our website: www.ihealth-t2d.org.

### Study locations

Participants were recruited from 120 sites divided equally between India, Pakistan, Sri Lanka and the UK (i.e. 30 recruitment sites per country). On the Indian subcontinent, study sites were distributed across a range of socio-economic and geographic settings, to increase the generalisability of the findings. Locations were identified based on the knowledge and advice of local experts and available administrative data for the region. In the UK, the study sites comprised GP surgeries in West London (London boroughs of Ealing, Harrow and Hounslow), selected as those serving populations with a high proportion of South Asians.

In India, our 30 study sites were distributed around five regional hubs comprising New Delhi (Delhi) and cities in four North Indian states: Gurugram (Haryana), Vaishali (Ghaziabad, Uttar Pradesh), Mohali (Punjab) and Muzaffarpur (Bihar). Delhi, officially known as the National Capital Territory (NCT), is a city and a union territory of India which includes New Delhi, the capital of India. There were six study sites (three rural and three urban or semi-urban) for each regional hub.

In Pakistan, the study area was the metropolitan district of Lahore, the second most populous city of Pakistan. It has an estimated population of approx.10 million with an urban:rural population in a 2:1 ratio. The city is divided into 10 basic administrative divisions (towns) that are further divided into smaller units termed as Union Councils (urban) and Mouzas (rural/village). Our 30 sites represented all 10 towns with an average of 3 sites per town. The boundaries of each site were based on the respective Union Council/Mouzas defined by the main roads encompassing the area. There were 20 urban and 10 rural sites. The urban sites were congested, thus smaller and represented one union council. Rural sites had less population density and thus were large and represented 2 or more Mouzas or villages. The 30 study sites were co-ordinated through three hubs: Services Institute of Medical Sciences (SIMS), Punjab Institute of Cardiology (PIC) and Sharif Hospital.

In Sri Lanka, the study was conducted in Colombo and Gampaha districts, the two most urban and populous districts of the country. Colombo district has 13 administrative divisions. We selected two of these divisions, each representing predominantly urban and predominantly rural populations. Eight (*n* = 8) clusters were selected from the urban setting and seven clusters were selected from the rural setting. A cluster was defined as a *Grama Niladhari* division. Gampaha district has 13 administrative divisions. Only seven of these divisions have urban populations. We sampled one urban cluster (*n* = 7) from each of the divisions with urban populations. One cluster each was selected from the other six divisions with only rural populations (*n* = 6). Two additional rural clusters were selected from the two most populous divisions that have both urban and rural populations (*n* = 2). A cluster was defined as a *Grama Niladhari* division with a Primary Care Hospital (Primary Medical Care Unit/Divisional Hospital) or a Maternal and Child Health clinic where screening can be conducted.

The 120 research sites were cluster randomised to intervention or usual care (1:1 allocation). Randomisation was done by the project management team at Imperial College London, using computer-generated random numbers, and as stratified by country to ensure 15 intervention and 15 usual care sites per country. Randomisation was carried out before recruitment started.

### Recruitment to the study

We invited South Asian men and women aged 40–70 years and without known diabetes to be considered for the study. Recruitment was a two-stage process: (i) an initial screening visit to assess eligibility for the intervention trial; (ii) an enrolment visit, to confirm eligibility and to obtain consent for inclusion in the intervention phase of the trial.

In South Asia, our primary recruitment strategy comprised an open invitation to individuals living in the community at study sites, to attend for an iHealth-T2D Screening visit. We first discussed the study with the relevant administrative and health authorities to engage their support and permission to operate. We held discussions with community leaders and open meetings to identify suitable approaches for engaging local communities in the project. We distributed knowledge of the project and invited people to attend for screening through trusted sources of health information (e.g. health centres, physicians and healthcare providers, grass root level non-physician health workers, accredited social health activists and volunteer groups). People were encouraged to discuss the project with neighbours and friends to help widen engagement. The screening health assessment was held in local community health centres easily accessible to the target community.

In the UK, our primary strategy was postal invitation. We sent letters by post, to men and women aged 40–70 years, registered to the practice lists of collaborating general practitioners and who were recorded to be of South Asian ethnicity, and not known to have diabetes. We sent 14,564 invitations, from which 3031 attended a screening visit (22%). From these, 408 attended enrolment and 240 were recruited to trial (i.e. 1.6% of people invited were ultimately recruited to the intervention phase). Screening was carried out in local GP surgeries, and subsequent visits at a dedicated research clinic based at Ealing Hospital. As a secondary strategy in the UK, we also took advantage of a database comprising the results of cardiovascular health assessments recently carried out amongst 9699 South Asians in the study sites, as part of the LOLIPOP study (2010–2015). These baseline assessments were done using methods identical to those of the iHealth-T2D Screening visit and are thus directly comparable. This approach enabled identification of South Asians meeting the study entry criteria based on HbA1c or waist circumference, who were then invited directly to a study enrolment visit using a single letter of invitation (2872 invitations sent; 1026 attended; 36% response rate). From these, 579 were recruited to trial (i.e. 20.2% of people invited). This secondary strategy also enables the evaluation of the cost-effectiveness for recruitment to lifestyle intervention based on existing, routinely collected healthcare data, compared to using systematic population-based screening strategies.

### Screening and enrolment visits

**Fig. 1:**
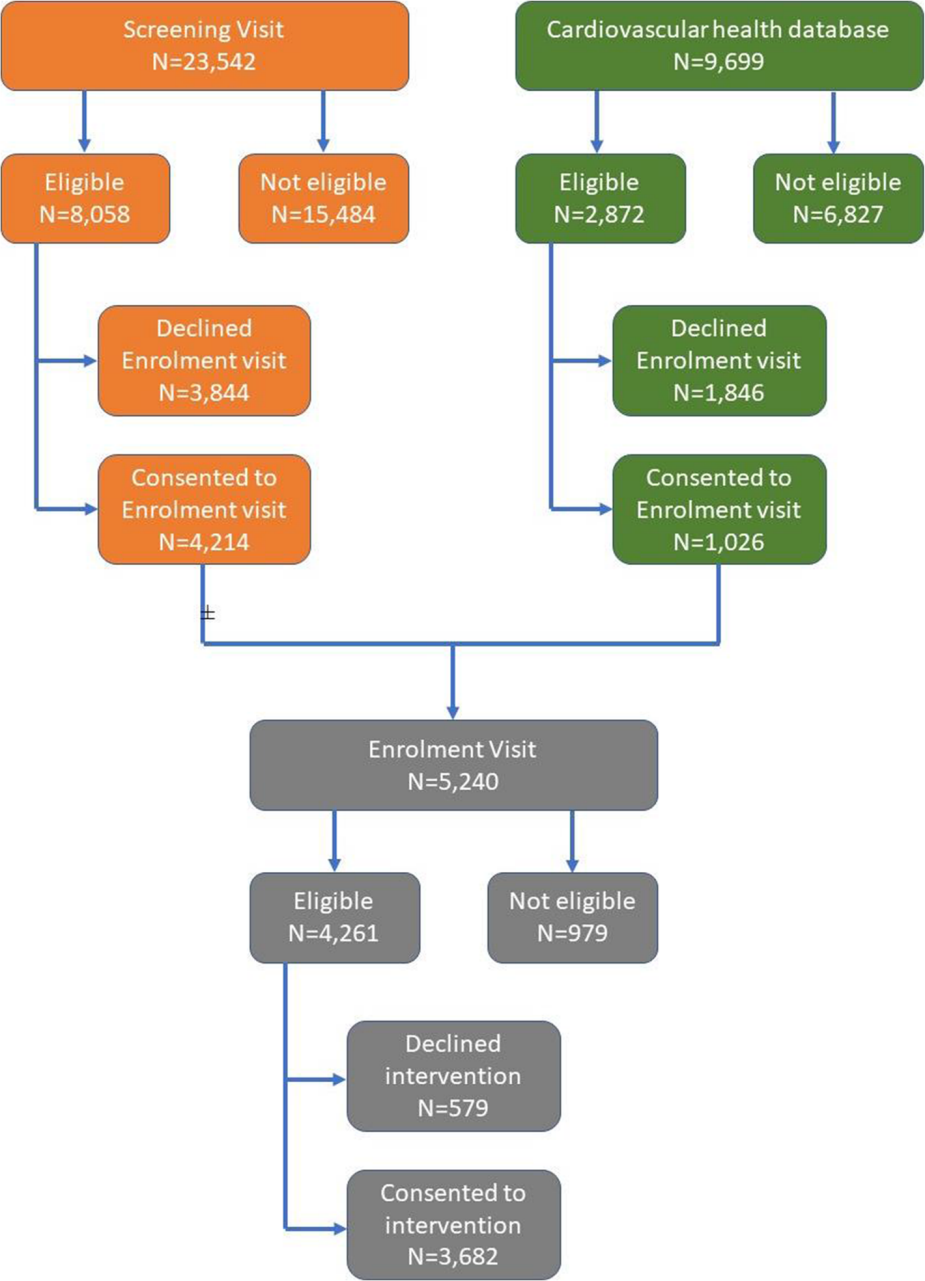
Overview of recruitment to the study

Potential participants were invited to attend an initial screening visit to assess them for eligibility (Table 3). This comprised (i) questionnaire (socio-demographic details, medical and drug history with emphasis on diabetes and cardiovascular disease, smoking and alcohol consumption), (ii) physical measurements (waist circumference, height, weight and blood pressure) and (iii) fasting blood samples (overnight) for measurement of HbA1c (study entry criterion), and for storage to enable epidemiological research into biomarkers for metabolic and cardiovascular health. Data collection forms are available on the study website (www.iHealth-T2D.org), and study methods in the protocol. People reaching the study entry criteria based on the initial screening assessment were invited to attend for an enrolment visit. The data collected at enrolment is the baseline data against which subsequent health outcomes will be assessed. In addition to repeating the measures collected in the screening visit, participants also completed the Global Physical Activity Questionnaire (GPAQ) and Health-related quality of life questionnaire (EQ-5D-5L). South Asians who continued to meet the study eligibility criteria were invited to enrol into the intervention phase of the study (Fig. 1).

**Table 3:** Schedule visits for the iHealth-T2D study. *GPAQ* generalised physical activity questionnaire, *Quality of life* EQ-5D-5L

	Screening	Enrolment	Follow-up 1	Follow-up 2	Follow-up 3
Time	−30 days	0	12 months	24 months	36 months
Consent	X	X			
Demographics	X				
Alcohol intake	X	X	X	X	X
Tobacco intake	X	X	X	X	X
Medications	X	X	X	X	X
Medical history	X	X	X	X	X
EQ-5D-5L		X	X	X	X
GPAQ		X	X	X	X
24-h food diary		X	X	X	X
Height	X				
Weight, waist, hip	X	X	X	X	X
Blood pressure	X	X	X	X	X
HbA1c	X	X	X	X	X
Biological samples	X	X	X	X	X

All measures were carried out according to standardised protocols. Waist circumference was measured at the midpoint between the lower margin of the ribs and the top of the iliac crest. Hip circumference was measured at the level of the greater trochanters. Waist and hip measurements were measured three times and the average calculated. Weight was measured in light clothing using portable digital scales accurate to 0.1 kg, and height using a portable stadiometer. Blood pressure was measured in the seated position using Omron digital devices. Three measurements were obtained from each participant, 1 min apart. All people attending for a study visit were given a printed report of their results, along with written guidance on their interpretation. HbA1c was measured using Biorad D10 or Variant II turbo assays. Assays were performed in local laboratories that participate in external quality control, and meet the standards of the International Federation of Clinical Chemists. All participants with newly detected medical conditions were referred to the local health service.

### Study intervention

At the 60 study sites randomised to the intervention, lifestyle modification was provided by the trained study CHW to the index cases, over 12 months (22 contact sessions), using the approaches set out in the study protocol. Family members of the index case living in the same household were encouraged to take part in lifestyle modification. Participation of family members was optional.

At the 60 study sites randomised to usual care, participants received a single episode of brief lifestyle intervention for diabetes prevention, supported by written material. This session lasted 30–60 min and was delivered by the CHW.

### Follow-up visits

All participants enrolled to the study were asked to attend follow-up visits at 12, 24 and 36 months after recruitment. Follow-up assessments were conducted by a member of the research team, and not by the CHW providing the intervention. At each follow-up visit, study participants completed a structured assessment similar to the enrolment evaluation. This included (i) follow-up questionnaire, (ii) physical measurements (waist circumference, weight and blood pressure), (iii) GPAQ and EQ-5D-5L and (iv) fasting venous blood sample for measurement of HbA1c and serum biomarkers. Protocols and equipment were identical to those used for the baseline evaluation.

### Blinding and data handling

As the trial involves active intervention on both arms, it was not possible for participants and intervention staff to be blinded. However, outcome assessors and data analysts were kept blind to the allocation. The trial established procedures to maintain separation between staff that take outcome measurements and staff that deliver the intervention. Staff members who obtained outcome measurements were not directly informed of the group assignment. Intervention staff and dieticians who delivered the intervention did not take outcome measurements during follow-up. All investigators, staff and participants were kept masked to outcome measurements and trial results. Personal and research data were collected by the local study team. Research data were coded (de-identified) with removal of personal identifiers, prior to sharing for analysis. The personal identifiers were not shared outside the local investigator team. The final trial dataset is accessible to the principal investigators at the partner institutions and their teams. It is also available to other researchers on request to the Study Steering Committee.

### Study administration

The Steering Committee provided overall supervision of trial conduct. Day-to-day management of the study was co-ordinated through the Project Management Team, based at Imperial College London working in partnership with the project co-ordinators at each of the study centres. The Project Management Team was responsible for day-day administrative and scientific management of the project, including study planning, organisation of meetings, delivery of the annual reports, trial master files and compliance. The trial was monthly reviewed for study activity, data completeness and timeliness by the Project Management Team. In addition, the Project Management Team from Imperial College London visited study sites annually visited to audit trial conduct. The funder was not involved in trial monitoring or audit. Further details about the project are available on the project website (www.ihealth-t2d.eu). Changes to study protocol will be co-ordinated by the Project Management Team at Imperial College and agreed by the study Steering Committee. They will be submitted to the study sponsor and the involved ethics committees for approval prior to implementation and published on the trial registry website (clinicaltrials.gov).

### Dissemination

Results of the study will be submitted for publication and reported at national and international meetings. Results will also be made available through the study website. International Committee of Medical Journal Editors (ICMJE) Recommendations will be followed for authorship eligibility. No professional writers will be involved. Statistical code will be available through GitHub or equivalent at publication. Study datasets are available through application to the Steering Committee. After the end of the research, an anonymised dataset will be made publicly available.

### Statistical analysis and data management

All analyses will be carried out according to our published statistical analysis plan (SAP) [18]. Full details of the statistical approach are described in the SAP and are briefly summarised here.

### Study endpoints

The primary endpoint is new-onset T2D in the index case, defined as (i) HbA1c ≥ 6.5% or (ii) physician diagnosis and on treatment for T2D. Secondary endpoints are the following: (i) physical measures: waist circumference, weight and blood pressure; (ii) lifestyle measures: smoking status, alcohol intake, physical activity and dietary intake; (iii) biochemical measures: fasting glucose, insulin and lipids (total and HDL cholesterol, triglycerides); and (iv) treatment compliance. Any adverse events were reported to the local principal investigator. Serious adverse events were reported to the study sponsor as well.

### Sample size and power calculations

The study aimed to include 3600 participants. Assuming that even rates for T2D for usual care are 6.8% per year, the study has 81% power to identify at *p* < 0.05 a reduction in T2D incidence of 35%, after a follow-up period of 3 years with an estimated drop-out rate of 10% within the total population [9].

### Data analysis

The primary analyses, conducted after 3 years of follow-up, will investigate whether intensive lifestyle modification reduces the risk of new-onset T2D (primary endpoint) compared to usual care amongst South Asians with (i) central obesity (*N* ~ 2700 participants), (ii) raised HbA1c (*N* ~ 900 participants) and (iii) overall (central and/or prediabetes, all ~ 3600 participants). The analyses will be performed according to the intention to treat principle with data from all participants enrolled in the study [19]. Multiple imputation will be used to address missing data. Random effects logistic regression will be used to estimate odds ratios for incidence of T2D and *95% CI*. Secondary outcomes will be evaluated both after 1 and 3 years of follow-up, which will allow for comparisons of both short- and long-term effects of the lifestyle intervention. Subgroup analyses amongst participants included in the study based on HbA1c and/or waist circumference will be performed. The differences between the two treatment arms will be estimated with a multi-level linear mixed-effects regression model.

## Health economic analysis

The primary aim of the health economic analysis is to determine the cost-effectiveness of lifestyle modification vs usual care for prevention of T2D amongst (i) South Asians on the Indian subcontinent and (ii) South Asians in Europe. The health economic analyses will include pre-specified subgroup analyses of cost-effectiveness between (i) South Asians identified to be at high risk of T2D based on central obesity vs raised HbA1c ≥ 6.0%; (ii) low-middle-income (Indian subcontinent) vs high-income (UK) regions; and (iii) socio-demographic: sex, socio-economic classes and across ages. The results of the cost-effectiveness analyses will be used to describe the potential clinical and financial implications of implementing the approaches into standard practice in the local and national health economies. Effectiveness will be expressed in quality-adjusted life years (QALYs), using responses to the EQ-5D-5L, as well as T2D cases prevented. Cost-effectiveness analyses will be performed based on the follow-up period, estimating incremental costs and QALYs using multi-level linear regression models in line with statistical analysis of other study endpoints, as well as using parametric extrapolation techniques to evaluate the intervention over longer time horizons. The estimates will enable assessment of the scalability of an intensive lifestyle modification intervention delivered by CHWs for the prevention of T2D, using central obesity and/or HbA1c for screening. The analyses of the costs of scaling-up will take into account available and future resource envelopes in the South Asian countries, and also current and future pricing for assays such as HbA1c, to understand current and future feasibility.

## Results

Participants were recruited between 15/06/2016 and 05/03/2019. Figure 1 shows the trial profile. In total, 33,212 people were screened for inclusion into the study. There were 23,542 people who attended an iHealth-T2D Screening Visit (Table 4). There were 10,930 people who met study entry criteria after the screening visit and who were invited to attend for the enrolment visit. Amongst these, 5240 people attended, of whom 4261 people (81.3%) continued to meet study inclusion criteria. The primary reasons for participants not being eligible at the enrolment assessment were waist circumference initially raised, but below the threshold on repeat (*N* = 251); HbA1c initially 6.0–6.4%, but < 6.0% on repeat (*N* = 375); repeat HbA1c ≥ 6.5% (*N* = 200) or now diagnosed T2D (*N* = 42); repeat BMI < 22 kg/m^2^ (*N* = 34); and incomplete data (*N* = 41).

**Table 4:** Characteristics of the 23,543 participants attending a screening visit

	India	Pakistan	Sri Lanka	UK
*N*	6913	6962	6535	3132
Female gender (%)	37.7%	47.6%	68.2%	53.1%
Age (years)	51.6 (8.8)	51.8 (8.6)	53.2 (8.7)	54.6 (8.8)
Current smoking (%)	6.2%	9.3%	7.3%	7.1%
Skilled worker (%)	58.0%	25.6%	28.3%	51.9%
Married (%)	96.2%	94.0%	84.8%	84.2%
Body mass index (kg/m^2^)	27.5 (4.6)	28.7 (5.7)	25.3 (4.4)	27.2 (4.5)
Systolic BP (mmHg)	133.8 (18.1)	130.4 (20.4)	134.2 (19.8)	127.8 (17.0)
Diastolic BP (mmHg)	81.9 (10.6)	80.8 (12.1)	78.6 (11.0)	79.3 (10.1)
Waist (cm)	98.1 (11.2)	97.9 (12.1)	87.8 (10.9)	93.1 (11.9)
Central obesity (%)	46.9%	43.9%	26.4%	27.1%
HbA1c (%)	6.1 (1.4)	6.3 (1.5)	6.0 (1.2)	5.6 (0.5)
Prediabetes (%)	19.2%	31.2%	23.5%	18.8%
HbA1c ≥ 6.5% (%)	21.8%	24.2%	14.4%	2.7%

Of these 4261 people meeting study entry criteria after completion of the enrolment visit, 3682 (86.4%) agreed to take part in the intervention phase (Table 5). There were no differences in gender between those who agreed or declined to take part in the intervention (agreed: male 86.6%, female 84.8%, *P* = 0.09). There were also no material differences in age (53.6 ± 8.7 vs 55.0 ± 8.7 years), body mass index (30.3 ± 4.5 vs 30.5 ± 4.9 kg/m^2^), waist (104.0 ± 9.3 vs 103.0 ± 10.7 cm) or HbA1c (5.76 ± 0.43 vs 5.77 ± 0.47 %) amongst those who agreed to take part in the intervention compared with those who declined, respectively (all *P* > 0.05).

**Table 5:** Characteristics of the 3682 participants starting intervention

	India	Pakistan	Sri Lanka	UK
*N*	909	1070	884	819
Female gender (%)	46.8%	48.5%	67.9%	32.5%
Age (years)	50.5 (8.0)	51.7 (7.8)	53.4 (7.8)	59.3 (8.5)
Current smoking (%)	4.1%	7.6%	5.2%	5.6%
Body mass index (kg/m^2^)	30.2 (4.2)	31.2 (5.2)	28.9 (3.9)	30.7 (4.2)
Systolic BP (mmHg)	131.4 (14.8)	127.9 (18.4)	131.2 (16.9)	135.7 (16.4)
Diastolic BP (mmHg)	81.3 (9.1)	81.9 (11.4)	79.6 (10.5)	82.3 (9.6)
Waist (cm)	105.5 (7.4)	105.1 (9.8)	97.9 (8.3)	107.0 (8.9)
Central obesity (%)	91.9%	78.5%	73.6%	88.0%
HbA1c (%)	5.6 (0.5)	5.9 (0.4)	5.8 (0.4)	5.7 (0.4)
Prediabetes (%)	32.9%	54.7%	56.6%	38.1%

The 3682 study participants are 49.2% female and aged 52.8 (*SD* 8.2) years. Clinical characteristics are well balanced between intervention and usual care sites (Table 6). The median number of participants recruited at each study site is 30 (range 13–54). More than 90% of follow-up visits are scheduled to be complete in December 2020. Based on the follow-up to end 2019, the observed incidence of T2D in the study population is 6.1% per annum. This compares favourably with the incidence predicted in the study protocol and power calculation.

**Table 6:** Characteristics of participants in active and usual care sites. *P* values are adjusted for differences in age, gender and country

	Active	Usual care	***P***
*N*	1844	1838	
Age	53.3 (8.7)	53.7 (8.6)	0.18
Sex (female %)	52.1%	49.3%	0.09
Never smoked	86.2%	86.1%	0.97
Height	162.7 (9.7)	161.9 (9.7)	0.32
Weight	80.3 (13.7)	79.1 (13.5)	0.31
BMI	30.3 (4.5)	30.2 (4.6)	0.41
Waist	103.9 (9.3)	103.8 (9.4)	0.70
SBP	130.7 (16.9)	131.9 (17.1)	0.51
DBP	81.0 (10.2)	81.6 (10.4)	0.42
HbA1c	5.76 (0.43)	5.74 (0.43)	0.49

## Discussion

We describe the rationale, design and implementation of the iHealth-T2D study, a cluster randomised trial that sets out to determine whether lifestyle intervention delivered by community health workers, to South Asians with central obesity or prediabetes, reduces the risk of T2D over 3 years. Our study is notable for the large sample size, multi-country design and the use of tools for screening and intervention that have been adapted for low-resource settings.

T2D is a major chronic disease, usually requiring lifelong pharmacological treatment. Having T2D substantially increases a person’s risk of future myocardial infarction, stroke, heart failure, visual impairment, renal impairment, cancer and other long-term conditions [20]. T2D is thus a leading global cause of morbidity, mortality and healthcare expenditure. The burden of T2D is especially high in South Asia [4–6]. The World Health Organization, United Nations and the International Diabetes Federation all recognise that there is an urgent need for action to reverse the current epidemic of T2D in South Asians.

T2D is preventable by lifestyle intervention comprising improved diet, weight loss and increased physical activity [7,8]. Although many of the studies have been carried out in European populations, lifestyle interventions have been confirmed to reduce the risk of T2D in South Asians [9]. However, the available literature suggests that the benefits of lifestyle intervention may be lower in South Asians. The reasons are not known but may include differences in disease aetiology, limited understanding or compliance with the intervention, reduced availability of alternate healthy foods or facilities for physical activity, social pressures that inhibit behaviour change, or the fidelity of implementation. The evidence for heterogeneity of effect between populations demonstrates the need for specific assessment of the clinical and cost-effectiveness for diabetes interventions in South Asian individuals from the varied ethnic and population subgroups, and in diverse settings. Furthermore, the evidence-based approaches currently described have not been scaled up and are not routinely available in clinical practice. Obstacles to sustainable implementation include reliance on the oral glucose tolerance test to identify high-risk individuals, as well as the use of dieticians and other qualified healthcare workers for the delivery of the intervention. The human and financial resources needed for implementation of these established approaches have are major obstacles to deployment at scale.

On this background, we designed the iHealth-T2D study, with the overall goal of advancing the prevention of T2D in South Asian populations. iHealth-T2D was designed to build on previous work, address key limitations in knowledge and explore more scalable approaches to the delivery of lifestyle intervention amongst South Asians. Our study includes three key innovations compared to previous work. First, we use waist circumference and HbA1c for the identification of South Asians at increased risk of T2D. Central obesity is widely recognised to be a defining characteristic for susceptibility to T2D amongst South Asians and can be assessed rapidly, reproducibly and accurately, using low-technology, low-cost approaches, by people with minimal training, and without a qualified healthcare background. Waist circumference is ideally suited to resource-poor settings. The choice of HbA1c was informed by our longitudinal population data, which identifies HbA1c as a highly predictive marker of risk for future diabetes in South Asians that can be rapidly measured using point of contact devices, using a single blood test, and without the need for fasting. Together, these characteristics make HbA1c well-suited to population screening in low-resource settings. Second, we use community health workers, combined with group and telephone contacts, for the delivery of the intervention with the goal of improving the cost-effectiveness, scalability and sustainability of implementation. Multiple studies from South Asia, and other low-resource settings, support the view that community health workers can provide clinically effective care for chronic diseases, such as hypertension and diabetes [10–12]. A role in diabetes prevention has not previously been evaluated. The results of the present study are thus anticipated to inform ongoing task shifting in primary healthcare systems, in support of improved prevention and control for non-communicable disease. Finally, our study is notable for its multi-country design and large sample size. This will enable investigation of the effectiveness on the intervention in key population subgroups, including different cultural, geographic and socio-economic settings. Our results will thus substantially extend knowledge for implementation and effectiveness for diabetes prevention in a range of contexts relevant to South Asian communities.

Our study does also have some limitations. Our recruitment methods did not enable the assessment of response rates in South Asia, raising the possibility of responder bias. However, the characteristics of participants are similar to those reported in other studies, arguing against significant population stratification. Our intervention still involves intervention by a health worker, which may represent an obstacle to scale-up. However, we implement task shifting from traditional healthcare teams to community health workers, to promote cost-effectiveness and sustainability. We also note that the intensive interventions using peer-support or digital strategies for lifestyle modification have not shown evidence for diabetes prevention in South Asians [21,22]. Our experiment will thus add to the spectrum of approaches to delivery and aims to deliver an optimal combination of resource utilisation and clinical effectiveness. We will not be able to collect individual-level health expenditure data, which will limit the accuracy of our health economic evaluation. We will mitigate against this through the use of curated datasets that we have created for health-related costs across South Asia.

In summary, the iHealth-T2D study of 3682 South Asians with central obesity or prediabetes will determine whether lifestyle modification delivered by community health workers will reduce the risk of T2D over 3 years. The iHealth-T2D study offers the opportunity to advance understanding of how best to prevent diabetes amongst South Asians and thereby address a key public health challenge in this major global ethnic group.

## Supplementary Information

**Additional file 1: S1**. CHW Handbook. Training manual summarising the content and delivery of the lifestyle intervention. Translated versions available through study website. **S2.** CHW Workbook. Case report form / intervention delivery workbook for the lifestyle intervention. Translated versions available through study website. **S3**. Participant Handbook for Lifestyle intervention. Written materials provided to the participant to support the CHW delivered lifestyle intervention. Translated versions available through study website. **S4.** Participant Handbook for Usual care. Translated versions available through study website. Supplementary Table 1: The a-priori power calculations under alternate assumptions with a sample size of 3,600 participants.

## Data Availability

Data will be available to others on completion of the research, by application to the Steering Committee.
