# Supplementary material for: The iHealth-T2D study, prevention of type 2 diabetes amongst South Asians with central obesity and prediabetes: study protocol for a randomised controlled trial
Source: Trials. 2021 Dec 18;22:928. doi: 10.1186/s13063-021-05803-7 (PMC8684177; doi:10.1186/s13063-021-05803-7)
Supplement: Supplementary file 1 — Additional file 1: S1. CHW Handbook. Training manual summarising the content and delivery of the lifestyle intervention. Translated versions available through study website. S2. CHW Workbook. Case report form / intervention delivery workbook for the lifestyle intervention. Translated versions available through study website. S3. Participant Handbook for Lifestyle intervention. Written materials provided to the participant to support the CHW delivered lifestyle intervention. Translated versions available through study website. S4. Participant Handbook for Usual care. Translated versions available through study website. Supplementary Table 1: The a-priori power calculations under alternate assumptions with a sample size of 3,600 participants. [file 13063_2021_5803_MOESM1_ESM.zip › Supplementary informationR1.docx]

Supplementary information

Supplementary Table 1: The a-priori power calculations under alternate assumptions with a sample size of 3,600 participants.

| **Assumptions** | | **Risk reduction^*^ detectable in South Asians with** | | |
| --- | --- | --- | --- | --- |
| **Event rate** | **Drop out rate** | **Waist ≥ 100 cm** | **HbA1c ≥ 6.0%** | **HbA1c ≥ 6.0% and / or waist ≥ 100cm** |
| Baseline | 10% | 30% | 24% | 24% |
| 20% lower | 10% | 32% | 26% | 27% |
| 40% lower | 10% | 37% | 30% | 30% |
| Baseline | 20% | 31% | 25% | 26% |
| Baseline | 30% | 33% | 27% | 27% |

^*^Reduction in risk of T2D for intensive lifestyle modification vs usual care detectable with 80% power at *P*<0.017, since adjustment for multiple testing was considered. Baseline predicted event rates for usual care are: 5.9% in South Asians with waist ≥100 cm, 14.6% in South Asians with HbA1c ≥ 6.0%, and 6.8% overall ^15^.
