# Supplementary material for: The iHealth-T2D study, prevention of type 2 diabetes amongst South Asians with central obesity and prediabetes: study protocol for a randomised controlled trial
Source: Trials. 2021 Dec 18;22:928. doi: 10.1186/s13063-021-05803-7 (PMC8684177; doi:10.1186/s13063-021-05803-7)
Supplement: Supplementary file 1 — Additional file 1: S1. CHW Handbook. Training manual summarising the content and delivery of the lifestyle intervention. Translated versions available through study website. S2. CHW Workbook. Case report form / intervention delivery workbook for the lifestyle intervention. Translated versions available through study website. S3. Participant Handbook for Lifestyle intervention. Written materials provided to the participant to support the CHW delivered lifestyle intervention. Translated versions available through study website. S4. Participant Handbook for Usual care. Translated versions available through study website. Supplementary Table 1: The a-priori power calculations under alternate assumptions with a sample size of 3,600 participants. [file 13063_2021_5803_MOESM1_ESM.zip › Addcorr2.zip!/S1 CHW-Handbook.pdf]

Family-based intervention to improve healthy lifestyle and prevent Type 2 Diabetes amongst South Asians with central obesity and prediabetes

Community Health Worker's Handbook

How to use this handbook?

This handbook is for you to keep and use during your time as a CHW on the iHealth project. You are strongly encouraged to read through this before and during your initial training. It is intended to provide both educational information and to be used as a resource to refer back to throughout your work as a CHW with iHealth.

The handbook provides:

- **Learning Modules:** Background information on many topics that you need to be familiar with in order to educate participants. The final module is a description of the iHealth protocol and the content of each clinic visit with an example script.
- **Standard operating procedures:** Standard procedures are explained and described so that each CHW will be taking measurements at the same time and in the same way. This is very important that these 'how-to' guides are adhered to throughout the study.
- **Appendix:** Additional material for your reference

These chapters will complement and add to the information you will learn about within your initial training.

Contents

Learning Module 1: Diabetes .... 6_
1.0 Diabetes 7
1.1 Type 1 diabetes mellitus (T1D) 7
1.2 Type 2 diabetes mellitus (T2D) 7
1.3 Risk factors for T2D 8
1.3.1 Being South Asian is a risk factor for type 2 diabetes 8
1.3.1.1 Why are south Asians more likely to develop T2D? 9
1.3.2 Body fat is the major risk factor for T2D 11
1.3.2.1 Body Mass Index (BMI) 12
1.3.2.2 Waist circumference 12
1.4 Symptoms and diagnosis of T2D 13
1.5 What is prediabetes? 13
1.6 Complications of T2D 14
1.7 Treatment of T2D 15
1.8 Cost of T2D 15

Learning Module 2: Diabetes Prevention 16
2.0 Diabetes is preventable 17
2.1 Key findings from diabetes prevention studies 18
2.1.1 Lifestyle intervention drastically reduces the risk of getting T2D 18
2.1.2 Weight loss is by far the major predictor of T2D risk reduction 18
2.1.3 Weight loss is not the only predictor of T2D risk reduction. 18
2.1.3.1 Physical activity is important 18
2.1.3.2 Healthy diet is important 19
2.1.4 Achieving as many goals as possible is best: weight loss, physical activity and healthy eating goals 19
2.1.5 References: Diabetes prevention studies 20
2.1.5.1 Finnish Diabetes Prevention Study (DPS) 20
2.1.5.2 US Diabetes Prevention Programme (DPP) 20
2.1.5.2 Indian Diabetes Prevention Program (IDPP) 20
2.2 Benefits of 5-10% weight loss 21

2.3 Recommendations for setting up a successful T2D prevention programme ... 23
2.3.1 Goals of a T2D prevention programme ... 23
2.3.2 Design of a T2D prevention programme ... 23
2.3.3 Dietary advice given in a T2D prevention programme ... 24
2.3.4 Physical activity in a T2D prevention programme ... 24
2.3.5 Behaviour Change in a T2D programme ... 25
2.3.6 References: Guidelines for setting up T2D programmes ... 26

Learning Module 3: Basics of nutrition ... 27
3.0 Basics of nutrition ... 28
3.1 Carbohydrates ... 30
3.2 Protein ... 32
3.3 Fat ... 33
3.3.1 Dietary sources of fat ... 34
3.4 Energy ... 35
3.4.1 What is energy and where does it come from? ... 35
3.4.2 Energy balance ... 37
3.5 Healthy Eating ... 39
3.5.1 Food groups ... 39
3.5.2 The Eat Well Plate ... 41
3.5.3 Healthy eating messages ... 43

Learning Module 4: Behaviour change ... 44
4.0 Behaviour Change ... 45
4.1 Person-centred approach ... 46
4.2 Information provision ... 47
4.3 Exploration and reinforcement of participants' reasons for wanting to change (risks, benefits, barriers and motivations and confidence about making changes) ... 48
4.3.1 The stages of change model ... 48
4.3.2 Overcoming barriers to change ... 51
4.3.2.1 Cost ... 51
4.3.2.2 Time constraints ... 51
4.3.2.3 Friends/family pressure ... 51
4.3.2.4 Skills and knowledge ... 51

4.4 Goal setting and action planning 52
4.4.1 SMART goals 52
4.4.2 Rewards 53
4.5 Record keeping 55
4.6 Coping plans and relapse prevention 56
4.6.1 Triggers 56
4.6.2 Eating behaviour chains 56
4.6.3 Dealing with pressures to eat 56
4.6.4 Thought patterns 57
4.6.5 Eating out 57
4.6.6 Shopping 57
4.7 Encourage family support 58
4.8 Encourage self-regulation and self-monitoring techniques 59

Learning Module 5: iHealth 60
5.0 Introduction to iHealth 61
5.1 Aims of the Family-based Lifestyle Modification Programme 62
5.2 Participants 63
5.3 Role of the community health worker 64
5.3.1 Community Health Worker competencies 64
5.3.1.1 Knowledge 64
5.3.1.2 Key skills 64
5.3.2 Data recording by the CHW 65
5.4 Clinic visit protocols and scripts: 68
5.4.1 Clinic Visit 1 68
5.4.2 Clinic Visit 2 73
5.4.3 Clinic Visit 3 Group session: Focus on fat 77
5.4.4 Clinic visit 4 Group session: Carbohydrates and triggers 80
5.4.5 Clinic visit 5 Group session: Food labels and eating out 84
5.4.6 Clinic Visit 6 Review session 88
5.4.7 Clinic Visit 7 Group session 91
5.4.8 Clinic Visit 8 Group session 93
5.4.9 Clinic visit 9 Review 95
5.4.10 Telephone call script 98

Standard operating procedures 101

SOP 1: Measuring Height 102

SOP 2: Measuring Body Weight 102

SOP 3: Measuring Waist Circumference 103

SOP 4: Measuring Hip Circumference 104

SOP 5: Multi-pass, 24hour dietary recall 105

SOP 6: History of weight gain 111

SOP 7: Diet and physical activity history 111

SOP 8: Estimating energy requirements 112

SOP 9: Food Group Portions Chart 116

SOP 10: Taking Informed Consent 117

SOP 11: How to conduct GPAQ 118

Appendix 121

Appendix 1: Informed Consent form for Family Members 122

Appendix 2: Participant Information Sheet for Family Members 124

Appendix 3: Inclusion and Exclusion Criteria for Family Members 122

Appendix 4: Frequently asked questions from participants about nutrition 125

Learning Module 1
Diabetes

1.0 Diabetes

Diabetes is the fifth leading cause of death worldwide and a major contributor to the development of coronary heart disease, stroke, peripheral vascular disease and end-stage renal failure. It is estimated that 7-11% of the world's population has diabetes and the vast majority of this, approximately 90%, is type 2 diabetes mellitus (T2D). The remaining 10% is type 1 diabetes mellitus (T1D) and a much smaller contribution comes from gestational diabetes and rarer forms.

Diabetes is a chronic condition that occurs when the body cannot produce enough insulin or cannot use insulin. Diabetes is diagnosed by observing raised levels of glucose in the blood (also known as hyperglycaemia).

What are insulin and glucose?
Insulin is a hormone produced by the pancreas that acts like a 'key' to let glucose pass from the blood stream into the cells where it can be used as energy. Carbohydrates are a large part of the human diet and found, in varying amounts, in almost all foods. When these foods are eaten the carbohydrates are broken down into individual units of glucose (a type of sugar). Glucose travels around the body in the bloodstream so that it can reach all the cells to provide them with energy. Insulin transfers the glucose from the blood stream into the cells.

1.1 Type 1 diabetes mellitus (T1D)

T1D is created by an autoimmune reaction in which the body's defence system destroys its own insulin-producing cells in the pancreas. Therefore, the pancreas is unable to produce insulin which means that the glucose that enters the bloodstream cannot pass out of the blood into the cells and so the amount of glucose in the blood keeps increasing to very high levels. Without access to daily insulin injections to control blood glucose levels, people with T1D will die.

The cause of T1D remains largely unknown, however unlike T2D, lifestyle factors such as diet, bodyweight and physical inactivity are not thought to play a large part in developing T1D.

1.2 Type 2 diabetes mellitus (T2D)

T2D is characterised by high blood glucose due to insufficient insulin production and/or an inability to use the insulin that has been produced (also called *insulin resistance*).

As with T1D, the exact cause is unknown, however T2D is very strongly associated with lifestyle factors such as poor diet, physical inactivity and body weight. <u>In fact, close to 90% of people with T2D are overweight or obese.</u>

1.3 Risk factors for T2D

A risk factor is any attribute, characteristic or exposure of an individual that increases the likelihood of developing a disease.

Risk factors for developing T2D are categorised as 'modifiable' and 'non-modifiable' risk factors. A modifiable risk factor is one that can be changed to improve or worsen the individual's risk of developing T2D. A non-modifiable risk factor is one that cannot be changed to improve or worsen the individual's risk of developing T2D.

Modifiable Risk Factors	Non-modifiable Risk Factors
**Overweight/obese - often measured by:**<br>• **BMI**<br>• **Waist circumference**	**Family history of diabetes**
**Unhealthy diet**	**Increasing age**
**Physical inactivity**	**Ethnicity**
**High blood pressure**	
**Smoking**	

Table 1.1: Modifiable and non-modifiable risk factors for T2D

1.3.1 Being South Asian is a risk factor for type 2 diabetes

Type 2 diabetes is increasing across the globe but it is increasing at a particularly fast rate in South Asian countries (India, Pakistan, Bangladesh, Nepal and Sri Lanka) as more people migrate from rural areas into urban areas which leads to a reduction in physical activity and dietary changes which creates a population with an increasing body weight. Alongside these lifestyle changes, South Asians are at a greater risk of developing T2D than white Caucasians because of biological and genetic differences; South Asians appear to develop T2D at an earlier age (10 years earlier in some reports) and at a lower body weight and progress to serious complications quicker than their white Caucasian counterparts.

Given that 20% (one fifth) of the world population is South Asian and access to prevention and treatment is variable in these regions, T2D imposes a serious and urgent global health concern.

India currently has the highest number of people with diabetes in any country and in some urban areas of India it is estimated that upto 17% of the population has T2D.

	Diabetes cases	National prevalence (%)
**India**	61,258,000	8.3%
**Bangladesh**	8,406,000	9.6%
**Sri Lanka**	1,078,000	7.8%
**Pakistan**	7,028,000	6.9%
**UK**	2,858,600	6.2%

Table 1.2: Prevalence of T2D by country (International Diabetes Federation 2015)

South Asians living in Europe have been reported to be 2- 5 times more likely to have T2D than white Europeans. The graph below shows estimates of diabetes prevalence in England by ethnic group in 2010.

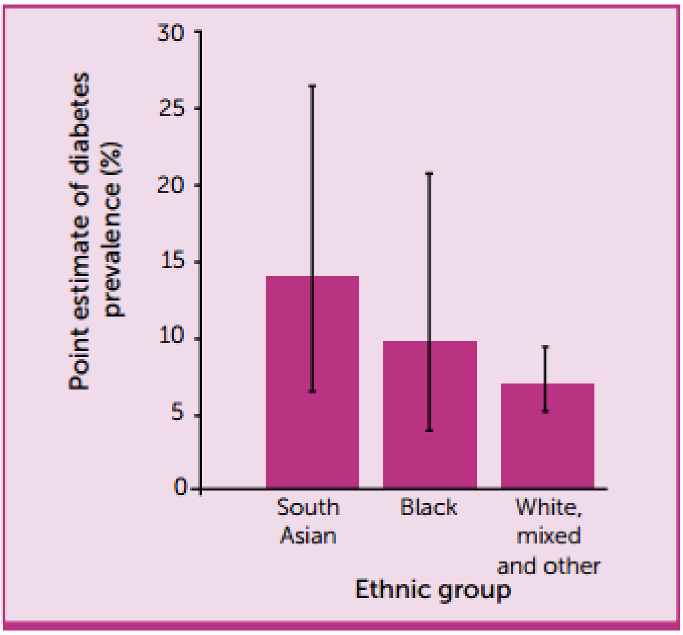

Figure 1.1: Estimates of diabetes prevalence by ethnicity for England in 2010

1.3.1.1 Why are south Asians more likely to develop T2D?

The answer to this question is still largely unknown. However, it is thought to be a mixture of lifestyle factors and biological factors.

Insulin resistance and body composition:

South Asians are more insulin resistant compared to other ethnicities. This greater degree of insulin resistance is present in children and continues into adulthood. It is partly due body composition differences but also metabolic differences.

South Asians have a higher amount of total body fat compared to Caucasians of the same body weight and a lower amount of lean body tissue. South Asians also have greater amounts of central fat (abdominal fat) compared to Caucasians of the same BMI. This difference is seen in infancy through to adulthood and is thought to be a major contributing factor to the increased prevalence of insulin resistance and development of T2D in South Asians.

Contrary to popular belief, body fat (adipose tissue) is a metabolically active tissue; it releases hormones and inflammatory factors that contribute to insulin resistance and dyslipidaemia and the

development of T2D and cardiovascular disease. Central fat (intra-abdominal fat around the organs) is more metabolically active than peripheral fat and poses a higher risk to health. Therefore waist circumference is a better predictor for T2D than BMI.

Lifestyle factors:

There is rapid urbanisation and migration happening in South Asian regions. This results in increased body weight and waist circumference and increased risk of T2D due to changing dietary and physical activity habits.

Physical activity: People living in rural areas do more physical activity throughout the day than people living in urban areas, during both work and spare time. Energy expenditure reduces significantly as people move into cities. It has also been shown that South Asians in Europe do upto 75% less physical activity than Europeans. Physical inactivity is strongly linked to insulin resistance and T2D.

People living in urban areas consume more energy-dense foods, more refined carbohydrates and fewer high-fibre carbohydrates than those living in rural areas which may contribute to the increased risk of T2D. Compared to Europeans, South Asian immigrants appear to consume more saturated fat, trans-fat and less monounsaturated and polyunsaturated fat, however this difference disappears as the time lived in the country increases.

1.3.2 Body fat is the major risk factor for T2D

Irrespective of ethnicity, being overweight or obese is the biggest risk factor for T2D. It is estimated that 80-85% of diabetes is caused by being overweight.

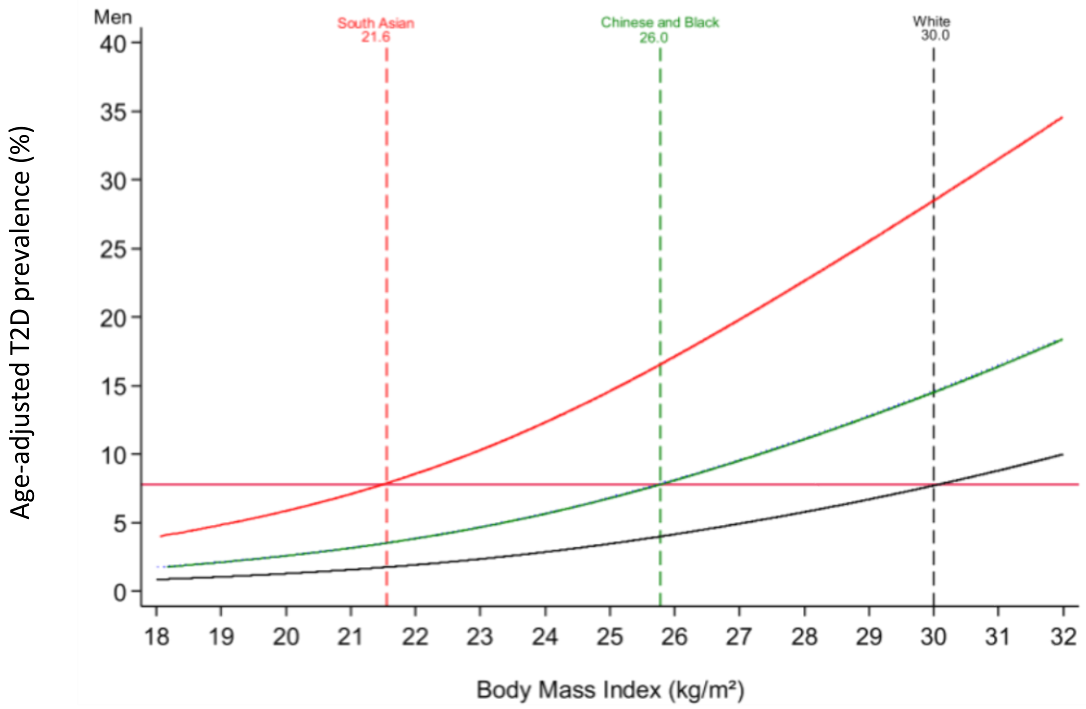

Figure 1.2: Age-adjusted T2D prevalence against BMI. The graph demonstrates that as BMI increases, the prevalence of T2D increases in all ethnic groups but at different rates. The horizontal line intersects to show the equivalent BMI in the South Asian and Chinese and Black groups that poses the same risk as a BMI of 30kg/m^2 in the White group. (Ntuk et al, 2014. UK Biobank data)

Because T2D presents at a much lower BMI and waist circumference in South Asians, there are separate classifications for overweight and obesity for South Asians and white Caucasians.

1.3.2.1 *Body Mass Index (BMI)*

The World Health Organisation definitions of overweight and obesity should be used to assess health risk. The definition of obesity in adults is usually based on the Body Mass Index (BMI).

BMI = Weight (kg)/Height (m^2)

However, because it is generally accepted that South Asians develop T2D in a lower range of BMI than Caucasians there are lower BMI classifications to assess obesity and the risk of diabetes in South Asians.

BMI (Kg/m^2)	South Asian classification	International classification	Risk of Co-morbidities
Underweight	< 18.5	< 18.5	Low (but increased risk of other clinical problems)
Healthy Weight	18.5 – 22.9	18.5 – 24.9	Average
Overweight	23 – 24.9	25 – 29.9	Mildly increased
Obese	≥ 25	≥ 30.0	Significantly increased

Table 1.3: BMI classification by ethnicity

1.3.2.2 *Waist circumference*

Simple measurements of BMI do not take into consideration the issue of fat distribution. Waist circumference measurements are an important risk factor assessment, indicating the accumulation of excess intra-abdominal fat. Individuals may be classified as being at a healthy weight according to BMI, yet have high levels of visceral abdominal obesity. Conversely, individuals with more muscle bulk than average e.g. athletes or individuals with manual jobs, might be misclassified as overweight according to BMI.

Waist circumference gives a reliable measure of visceral abdominal obesity and is now recognised to be a more accurate indicator of the health risks of obesity than BMI. This is especially true of some ethnic populations, particularly those of South Asian descent who are particularly prone to central obesity.

Increased risk levels for South Asians compared to Caucasian Europeans for waist circumference are shown below:

	South Asian	White European
Male	>90cm	>102cm
Female	>80cm	>88cm

Table 1.4: Waist circumference classification by ethnicity

1.4 Symptoms and diagnosis of T2D

T2D may be first detected by the presentation of symptoms such as feeling tired, being very thirsty, urinating often, blurred vision and poor wound healing. These are all symptoms of having high blood glucose levels.

T2D is diagnosed by testing the blood for high glucose. It can be done by looking at fasting glucose levels or glucose levels two hours after consuming a glucose drink (called an oral glucose tolerance test). Another blood test can be used which measures HbA1c in the blood. The HbA1c test measures your average blood glucose over the past 2-3 months in one sample – this is advantageous because it is relatively inexpensive and the blood sample can be taken at any time of day without the need for the patient to be fasted or to consume a glucose drink. **T2D is diagnosed if HbA1c is 6.5% or more.**

1.5 What is prediabetes?

Before people get T2D they will almost always have prediabetes. Prediabetes is when you have higher than normal glucose levels, but not high enough to be diagnosed with diabetes. **Prediabetes is present when HbA1c is over 6% and less than 6.5%.**

People who have prediabetes are at a very high risk of developing T2D but this does not mean that everyone with prediabetes will develop diabetes. By improving diet, increasing physical activity and reducing body weight, high blood glucose levels can return to normal which reduces the likelihood of developing T2D greatly.

Some people can have prediabetes for a long time before it is detected because the symptoms mentioned above may not be present. Therefore, early and regular screening for prediabetes in high-risk groups and the provision of lifestyle intervention programmes is very important in reducing the level of T2D developing across the world.

1.6 Complications of T2D

If T2D isn't treated it can lead to a number of serious complications and premature mortality. Raised blood glucose levels (even mildly raised) can damage blood vessels, nerves and organs. It is thought that people with diabetes will die 10 years earlier than people without diabetes

Heart disease and stroke: Prolonged, poorly controlled blood glucose levels increase the likelihood of atherosclerosis (where the blood vessels become clogged up and narrowed by fatty substances). This may result in poor blood supply to the heart which causes angina (chest pain). It also increases the chance that a blood vessel in the heart or brain will become blocked, leading to a heart attack or stroke. People with diabetes are 5 times more likely to have a cardiovascular disease or stroke.

Kidney disease: If the blood vessels in the kidney become blocked or leaky, kidneys will work less efficiently. It's usually associated with high blood pressure, and treating this is a key part of management. In severe cases, kidney disease can lead to kidney failure. Dialysis, or sometimes kidney transplantation, will be necessary. Almost half of all new cases of kidney failure are due to diabetes.

Eye damage: Diabetic retinopathy is when the retina (the light-sensitive layer of tissue at the back of the eye) becomes damaged. Blood vessels in the retina can become blocked or leaky or can grow haphazardly. This prevents light from fully passing through to the retina and damages vision.

Nerve damage: High blood glucose levels can damage the small blood vessels in the nerves. This can cause a tingling, burning pain or numbness that starts in the hands and feet and moves up through the limbs. If the nerves to the digestive system are affected it may lead to nausea, vomiting, diarrhoea or constipation.

Foot problems: Damage to the nerves of the foot can mean small nicks and cuts aren't noticed, and this, in combination with poor circulation, can lead to a foot ulcer. Diabetes is the leading cause of all non-traumatic lower limb amputations.

Sexual dysfunction: Men with diabetes, particularly those who smoke, may have nerve and blood vessel damage in the penis that can lead to erection problems. Women with diabetes may experience a reduced sex drive, less pleasure and less ability to orgasm, vaginal dryness and pain during sex.

Miscarriage and stillbirth: Pregnant women with diabetes have an increased risk of miscarriage and stillbirth. If blood glucose levels aren't controlled during the early stages of pregnancy, there's also an increased risk of birth defects.

Depression: People with diabetes are twice as likely to suffer an episode of depression.

1.7 Treatment of T2D

The aim of treatment for T2D is to lower and maintain healthy blood glucose levels.

Lifestyle modification is an important part of treatment of T2D. Blood glucose levels can be reduced and managed by improving diet (reducing intake of fat and saturated fat and increasing fibre intake) increasing physical activity, reducing body fat and maintaining a healthy body weight.

Medication to manage blood glucose levels (such as oral metformin and insulin injections) are required in more progressed T2D, but should still be used in conjunction with lifestyle management.

1.8 Cost of T2D

T2D is very expensive because alongside efforts to manage glucose levels there are many associated complications which if present, require specific management and medication. Twelve percent of global health expenditure is spent on diabetes.

Learning Module 2
Diabetes Prevention

2.0 Diabetes is preventable

Large, high-quality clinical trials show that modest changes to diet and physical activity through a structured, 1 year, lifestyle intervention can reduce the incidence of T2D by more than 50% in people at high risk of T2D.

There have been three large T2D prevention studies from which we can take learnings and from which guidelines/recommendations have been developed as to how to set up a successful diabetes prevention programme:

- Finnish Diabetes Prevention Study (DPS)
- US Diabetes Prevention Programme (DPP)
- Indian Diabetes Prevention Program (IDPP)

All three studies used behaviour-change strategies to help people increase physical activity, eat more healthily and lose (or maintain) body weight. As you can see in the table below, they all had similar, specific goals.

Trial Goals	Physical activity	Weight loss	Increase fibre intake	Reduce total fat intake	Reduce saturated fat intake	Other dietary goals
**Finnish diabetes prevention study (DPS) (Tuomelihto et al. 2001)**	More than 4 hours/week, moderate intensity	5% or more of initial body weight	Over15 g per 1000 kcals	Reduce to less than 30% of energy intake	Less than 10% of energy intake	
**Diabetes prevention program (DPP) USA (DPP Research Group. 2002)**	At least 150 minutes/ week moderate intensity	7% of initial body weight		Adopt a low-fat diet		Low calorie
**Indian diabetes prevention program (IDPP) (Ramachandran et al. 2006)**	At least 30 minutes brisk walking or cycling a day		Include fibre rich foods	Reduction in fats		Reduce total calorie and refined carbohydrate intake. Avoid sugar

Table 2.1: Goals of the major diabetes prevention trials

2.1 Key findings from diabetes prevention studies

2.1.1 Lifestyle intervention drastically reduces the risk of getting T2D

DPP – reduced the risk of T2D by 58%
DPS - reduced the risk of T2D by 58%
IDPP - reduced the risk of T2D by 28.5%

2.1.2 Weight loss is by far the major predictor of T2D risk reduction

Just 5-7% weight loss is effective at reducing the risk of T2D. It is estimated that for every 1kg lost, the risk of T2D is reduced by 16%.

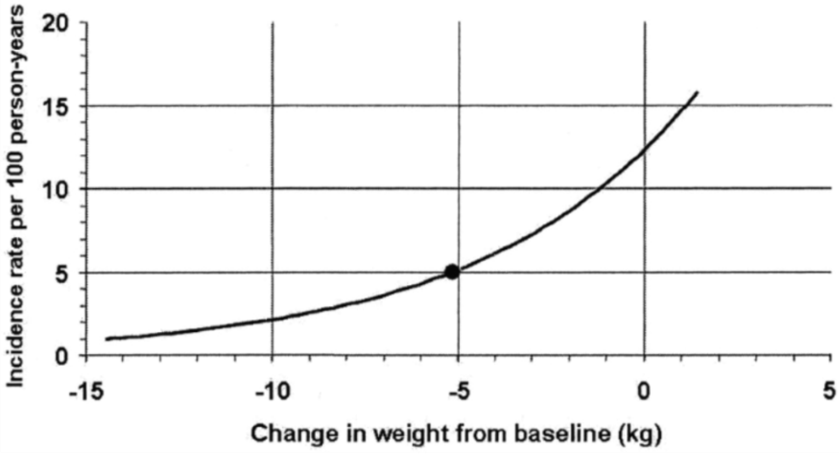

Figure 2.1: T2D incidence by change in weight from baseline among DPP participants.

2.1.3 Weight loss is not the only predictor of T2D risk reduction.

For example, in the IDPP study, participants did not lose a significant amount of body weight yet still reduced their T2D risk by adopting other lifestyle changes.

2.1.3.1 Physical activity is important

DPP participants who did not meet the weight loss goal, but met the activity goal had a 44% reduction in T2D incidence, independent of weight loss.
DPP also showed that increasing physical activity predicted long term success in maintaining the initial weight loss

2.1.3.2 Healthy diet is important

DPS found that increasing fibre intake and decreasing fat intake reduces the risk of T2D, independently of weight loss and physical activity.

2.1.4 Achieving as many goals as possible is best: weight loss, physical activity and healthy eating goals

DPS had 5 lifestyle goals (see table above). The risk of developing T2D is significantly reduced if only one of the 5 goals is achieved but the risk continues to reduce as the number of goals achieved increases. Of those who achieved 4 or more goals, not one person developed T2D at three year follow-up.

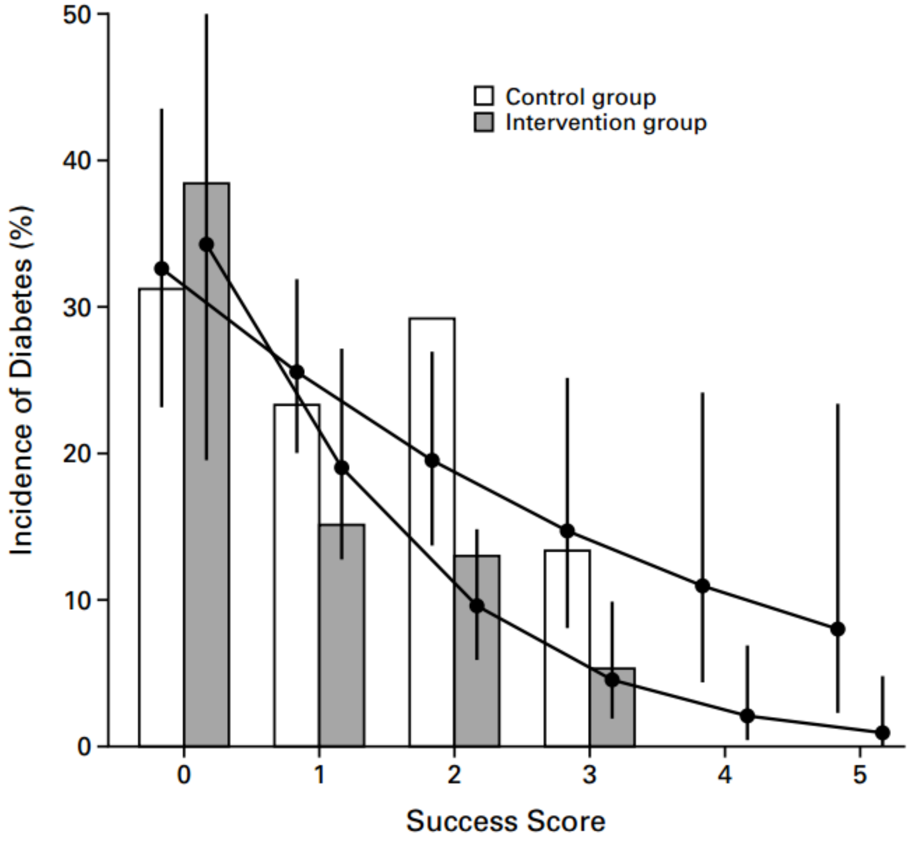

No. with Diabetes/Total No.						
Intervention group	5/13	10/66	9/69	2/38	0/25	0/24
Control group	15/48	25/107	14/48	2/15	0/11	0/4

Figure 2.2: Incidence of T2D according to the number of lifestyle goals achieved in DPS

Similarly, DPP, participants who lost even more than 5–7% body weight, and who met physical activity and dietary fat goals, reduced their T2D risk by over 90%.

2.1.5 References: Diabetes prevention studies

2.1.5.1 Finnish Diabetes Prevention Study (DPS)

Tuomilehto et al (2001) *N Engl J Med*:344:1343-50
Lindström et al (2006) *Diabetologia* 49: 912–920

2.1.5.2 US Diabetes Prevention Programme (DPP)

DPP Research Group (2002) *N Engl J Med:* 346:393-403
Hamman et al (2006) *Diabetes Care*: 29(9): 2102–2107.

2.1.5.2 Indian Diabetes Prevention Program (IDPP)

Ramachandran et al (2006) *Diabetologia*; 49: 289–297

2.2 Benefits of 5-10% weight loss

Weight loss of 5-10% can reduce the risk of T2D by half

**Reduces ill-health and mortality:**	**Reduces risk factors for heart disease:**
↓ of > 20% in total mortality	↓ in systolic BP of 10mm Hg
↓ of > 30% in T2D deaths	↓ in diastolic BP of 20mm Hg
↓some types of cancer	↓ of 10% in total cholesterol
↓heart disease	↓ of 15% in LDL cholesterol
↓stroke	↓ of 30% in triglycerides
↓osteoarthritis/joint pain	↑ Of 8% in HDL cholesterol
↓back pain	

**Other personal benefits:**	
Have more energy and vitality	Feel better/healthier
Do more with family	Improve mood
Ability to exercise more easily	Feel self-empowered
Improve mobility	Sleep better
May improve fertility	Fit into different clothes

2.3 Recommendations for setting up a successful T2D prevention programme

2.3.1 Goals of a T2D prevention programme

Intensive lifestyle-change programmes should offer ongoing tailored advice, support and encouragement to help people:

- Undertake ≥150 minutes of 'moderate-intensity' physical activity per week
- Reduce weight by 5–10%. This will substantially lower the risk of T2D. Gradually work towards a BMI within the healthy range.
- Increase consumption of foods high in dietary fibre
- Reduce total fat and saturated fat in the diet

2.3.2 Design of a T2D prevention programme

- Identify people at high-risk of T2D
- Ensure programmes are delivered by practitioners with relevant knowledge and skills
- Ensure programmes adopt a person-centred, empathy-building approach. This includes finding ways to help participants make gradual changes by understanding their beliefs, needs and preferences. It also involves building their confidence and self-efficacy over time.
- Ensure programme components are delivered in a logical progression. For example: discussion of the risks and potential benefits of lifestyle change; exploration of someone's motivation to change; action planning; self-monitoring and self-regulation.
- Maximize frequency and number of contacts with the participant:
  - Participants should have at least 16 hours of contact time either within a group, on a one-to-one basis or using a mixture of both approaches over a period of 9-18 months
  - Groups of 10–15 people
  - Offer more intensive support at the start of the programme by delivering core sessions frequently (for example, weekly or fortnightly). Reduce the frequency of sessions over time to encourage more independent lifestyle management.
- Allow time between sessions for participants to make gradual changes to their lifestyle – and to reflect on and learn from their experiences. Also allow time during sessions for them to share this learning with the group.
- From the outset, emphasis should be placed on long-term weight maintenance.
- Engage social support for the planned behaviour change

2.3.3 Dietary advice given in a T2D prevention programme

Find out what people already know about the types and amounts of food and drink that can help reduce the risk of type 2 diabetes. Provide this information where necessary. Explain that increasing dietary fibre intake and reducing fat intake (particularly saturated fat) can help reduce the chances of developing type 2 diabetes.

Help people to assess their diet and identify where and how they could make it healthier, taking into account their individual needs, preferences and circumstances. (For example, take into account whether they need to lose weight or if they have a limited income.)

Encourage people to:

- Increase their consumption of foods that are high in fibre, such as wholegrain bread and cereals, beans and lentils, vegetables and fruit.
- Choose foods that are lower in fat and saturated fat, for example, by replacing products high in saturated fat (such as butter, ghee, some margarines or coconut oil) with versions made with vegetable oils that are high in unsaturated fat, or using low-fat spreads.
- Choose skimmed or semi-skimmed milk and low-fat yoghurts, instead of cream and full fat milk and dairy products.
- Choose fish and lean meats instead of fatty meat and processed meat products (such as burgers).
- Grill, bake, poach or steam food instead of frying or roasting (for example, choose a baked potato instead of chips).
- Avoid food high in fat such as mayonnaise, chips, crisps, pastries, poppadums (papads) and samosas.
- Choose fruit, unsalted nuts or low-fat yoghurt as snacks instead of cakes, biscuits, bombay mix or crisps.

2.3.4 Physical activity in a T2D prevention programme

Find out what people already know about the benefits of physical activity and the problems associated with a sedentary lifestyle. Where necessary, provide this information.

- Explain that being more physically active can help reduce their risk of type 2 diabetes, even when that is the only lifestyle change they make.
- Explain that the minimum of 150 minutes of 'moderate-intensity' activity per week can be taken in bouts of 10 minutes or more. Include resistance and aerobic exercises.
- Explain that even small increases in physical activity will be beneficial, even if it is unrealistic for them to achieve the recommended minimum
- Explain that people should also reduce the amount of time they spend sitting at a computer or watching TV. Encourage them to be more active during work breaks, for example, by going for a walk at lunchtime.

- Help people to identify which of their activities involve 'moderate' or 'vigorous' physical activity and the extent to which they are meeting the minimum recommendation.

- Encourage people to choose physical activities they enjoy or that fit easily within their daily lives.

- Encourage people to set short and long-term goals.

- Encourage people to keep a record of their activity and to record the things that make it easier or harder. Help them to find other ways to identify and overcome any barriers to physical activity.

- Provide information on local opportunities for physical activity.

2.3.5 Behaviour Change in a T2D programme

Use evidence-based behaviour-change techniques to help overweight and obese people eat less, be more physically active and make long term changes that result in steady weight loss.

- Information provision: to raise awareness of the benefits of and types of lifestyle changes needed to achieve and maintain a healthy weight, building on what participants already know.

- Exploration and reinforcement of participants' reasons for wanting to change and their confidence about making changes. This may include using motivational interviewing or similar techniques suitably adapted for use in groups.

- Goal setting: prompting participants to set achievable and personally relevant short and long-term goals.

- Action planning: prompting participants to produce action plans detailing what specific physical activity or eating behaviour they intend to change – and when, where and how this will happen. They should start with achievable and sustainable short-term goals and set graded tasks (starting with an easy task and gradually increasing the difficulty as they progress towards their goal). The aim is to move over time towards long-term, lifestyle change.

- Coping plans and relapse prevention: prompting participants to identify and find ways to overcome barriers to making permanent changes to their exercise and eating habits. This could include the use of strategies such as impulse-control techniques (to improve management of food cravings).

- Participants should be encouraged to involve a family member, friend or carer who can offer emotional, information, planning or other practical support to help them make the necessary changes. (It may sometimes be appropriate to encourage the participant to get support from the whole family.)

- Participants should be encouraged to use self-regulation techniques. This includes self-monitoring (for example, by weighing themselves, or measuring their waist circumference or both). They should also review their progress towards achieving their goals, identify and find ways to solve problems and then revise their goals and action plans, where necessary. The aim is to encourage them to learn from experience

2.3.6 References: Guidelines for setting up T2D programmes

The recommendations above have been taken from the following guidelines:

Type 2 diabetes: prevention in people at high risk. NICE guidelines [PH38] Published date: July 2012. https://www.nice.org.uk/guidance/ph38

Behaviour change: general approaches. NICE guidelines [PH6] Published date: October 2007. https://www.nice.org.uk/guidance/ph6

A European Evidence-Based Guideline for the Prevention of Type 2 Diabetes (2010) *Horm Metab Res*; 42 (Suppl. 1): S3–S36

Learning Module 3
Basics of nutrition

3.0 Basics of nutrition

Food provides us with vital energy and nutrients without which we cannot survive. As well as consuming enough water, there are two groups of nutrients that we must eat in order to stay alive, we call these micronutrients and macronutrients:

Micronutrients

Micronutrients is the collective name for vitamins and minerals. 'Micro' means 'small'. We only need to consume micronutrients in very tiny amounts. However they are essential to life because some processes in the body cannot happen without them (for example, building tissue and bone and maintaining fluid balance). By not eating any one vitamin or mineral will lead to a specific deficiency disease and eventually death. We get micronutrients from many different food sources, therefore by eating a varied diet we will consume enough vitamins and minerals to remain healthy. (See table 3.2 for vitamins and minerals and their major food sources)

Macronutrients

Macronutrient is the collective term for the nutrients that give us energy. All life-forms require energy to live. 'Macro' means 'big'. We need to eat macronutrients in large amounts. Food is mostly made up of water and macronutrients in varying proportions. There are four macronutrients:

1. Carbohydrate (sugars and starches)
2. Fat
3. Protein
4. Alcohol

Carbohydrate, fat and protein are essential to life. Alcohol does provide us with energy and is therefore considered a macronutrient but it is not essential to life. See the table below to see the energy provided by one gram of each macronutrient. Energy is shown here in calories (kcal).

Macronutrient	Energy (kcal/g)
**Carbohydrate**	4kcal/g
**Fat**	9kcal/g
**Protein**	4kcal/g
**Alcohol**	7kcal/g

Table 3.1: Calories contained in 1gram of each macronutrient

Vitamins	Food source	Minerals	Food source
**Vitamin A**	Liver, whole milk, cheese, butter, orange coloured fruit/veg and dark, green leafy veg. (other fat spreads are fortified with vitamin A in some countries, such as the UK)	**Calcium (Ca)**	Dairy, some green leafy vegetables such as broccoli/cabbage (not spinach), fish eaten with bones such as whitebait/sardines/tinned salmon. (Bread flour and soya products may be fortified with Ca in some countries, such as the UK)
**Vitamin B_1 (Thiamin)**	Whole grains, nuts, meat, fruit and vegetables. (Breakfast cereals and bread flour is fortified with vitamin B_1 in some countries, such as the UK)	**Phosphorous (P)**	red meat, dairy products, fish, poultry, bread, rice and oats
**Vitamin B_2 (Riboflavin)**	Milk, eggs, liver, legumes, mushrooms and green vegetables. (Breakfast cereals may be fortified with vitamin B_2 in some countries, such as the UK)	**Magnesium (Mg)**	green leafy vegetables, nuts, bread, fish, meat and dairy products
**Vitamin B_3 (Niacin)**	Meat, wheat and maize flour, eggs, dairy products and yeast	**Sodium (Na)**	Most foods contain sodium. Salt is sodium chloride (NaCl) so most people consume too much sodium by adding salt to food and eating processed foods which contain a lot of salt. The amount of salt eaten should be minimised.
**Vitamin B_6 (Pyridoxine)**	Poultry, white fish, milk, eggs, whole grains, soya beans, peanuts and some vegetables	**Potassium (K)**	Most foods contain potassium. Fruit (particularly bananas), vegetables, meat, fish, shellfish, nuts, seeds, pulses and milk.
**Vitamin B_9 (folic acid or folate)**	Green leafy vegetables, brown rice, peas, oranges, bananas (Breakfast cereals may be fortified with vitamin B_9 in some countries, such as the UK)	**Iron (Fe)**	Liver, red meat, pulses, nuts, eggs, dried fruits, poultry, fish, whole grains and dark green leafy vegetables
**Vitamin B_{12} (Cyanocobalamin)**	Meat, fish, milk, cheese, eggs, yeast extract (Breakfast cereals may be fortified with vitamin B_{12} in some countries, such as the UK)	**Zinc (Zn)**	Meat, milk, cheese, eggs, shellfish, wholegrain cereals, nuts and pulses
**Vitamin C (Ascorbic acid)**	Fresh fruits especially citrus fruits and berries; green vegetables, peppers and tomatoes; potatoes (especially new potatoes)	**Iodine (I)**	Sea fish, shellfish, seaweed, milk. The amount in plant sources varies depending on the amount of iodine in the soil. In some countries salt and bread may be fortified with iodine.
**Vitamin D**	Oily fish, eggs, meat, fat spreads and some cereals may be fortified with vitamin D. Sunlight is another source of vitamin D.	**Flouride (Fl)**	Tea and fish. However most people cannot get enough from the diet so many countries add fluoride to the water system and to toothpaste.
**Vitamin E**	Vegetable oils, nuts and seeds	**Copper (Cu)**	shellfish, liver, kidney, nuts and wholegrain cereals
**Vitamin K**	Green leafy vegetables, dairy products and meat	**Selenium (Se)**	Brazil nuts, bread, fish, meat and eggs. The amount in plant sources varies depending on the soil content.
		**Manganese (Mn)**	vegetables, cereals, nuts, tea.
		**Chromium (Cr)**	meat, nuts, cereal grains, brewer's yeast and molasses

Table 3.2: Vitamins and minerals and the major foods from which we get them

3.1 Carbohydrates

There are three groups of carbohydrates:

1. Sugar
2. Starch
3. Fibre (sometimes referred to as indigestible carbohydrate)

The body's cells need a constant supply of glucose which we get by eating carbohydrates. At least 50% of the energy we consume should come from carbohydrates. During digestion starchy foods are broken down into glucose which is absorbed from the small intestines into the bloodstream. Similarly, table sugar is broken down into glucose and fructose which are absorbed into the bloodstream. The hormone insulin is then needed to transfer the glucose from the blood into the cells where it can be used as energy.

Main sources of sugar are: table sugar, sugar from fruit and dairy products, honey and other sources.

Main sources of starchy carbohydrates are: cereals such as rice, maize (corn), oats, wheat, barley, potatoes.

Although both starch and sugar are broken down into the same thing, the carbohydrate source in a healthy diet should come from mostly starchy foods and only a small amount from sugar. This is because of three reasons:

1. Starchy foods takes longer to digest than sugars and so the rise in blood glucose is slower when we eat starch which is thought to be better for us.

2. Sugar added to foods (usually table sugar) is sometimes referred to as 'empty calories', which means that it only provides energy and no other nutrients, whereas starchy foods can provide many other micronutrients and fibre along with the energy. Adding 'empty calories' to the diet is not helpful in places where food is plentiful and increasing body weight is a problem in the population.

3. Tooth decay is strongly associated with the frequency of sugar consumption. Starchy foods too can cause tooth decay however it is thought not to be as detrimental as sugar.

Fibre, or indigestible carbohydrate, comes from the cell walls in plants (cereals and fruit and vegetables). However, during food processing of cereals the fibre is often removed by the removal of the husk (plant cell wall). White pasta, white rice and white chapatti/bread flour still contain plenty of starch but they have had most of the fibre removed. Brown or wild rice, brown or wholewheat pasta, brown or wholemeal chapatti flour/bread and oats, beans and lentils are fibre-rich foods and can be used as a healthier, fibre-rich alternatives to 'white', processed cereals.

Fibre plays a different role to starches and sugars because it isn't readily available as an energy source because humans are unable to break it down into its component sugars, hence its alternative

name ‘indigestible carbohydrate’. Therefore, when we eat fibre it travels through the small intestines without being digested or absorbed and remains intact until it reaches the large intestine (also known as the bowel). Here it meets the resident bacteria that live within all of our large intestines. The bacteria can ferment the fibre (fermenting is another way to break down the carbohydrates) and use the end products of this fermentation as their own energy source. The host (the body) can absorb some of the products of fermentation as energy but not all. Eating a diet high in fibre is considered healthy for three reasons:

1. Bowel health. Some studies suggest that populations with a high intake of fibre-rich foods experience a lower incidence of large bowel cancer than populations with low intakes of these foods.

2. Heart health: Fibre rich foods may reduce cholesterol levels in the blood and reduce the risk of cardiovascular disease (CVD).

3. Obesity: As we cannot absorb all the energy from fibre, fibre-rich foods provide us with less energy than other foods and so provide the body with fewer calories which is useful for people who are trying to maintain a healthy body weight. Some studies also suggest that fibre contributes to feelings of fullness and satisfaction after a meal which means that people may eat less at each sitting and also reduce snacking between meals when they are consuming a fibre-rich diet.

3.2 Protein

Protein should provide the body with approximately 10 to 15% of its dietary energy. Aside from providing energy, proteins are fundamental structural and functional elements within every cell of the body and are involved in a wide range of metabolic interactions. Therefore protein is essential for growth and repair and the maintenance of good health.

Proteins are made up of amino acids joined together in long chains which then fold up into 3D structures (protein) that have very specific shapes and functions depending on the order and composition of the amino acids that make up the chain. Amino acids can be thought of as the building blocks of proteins.

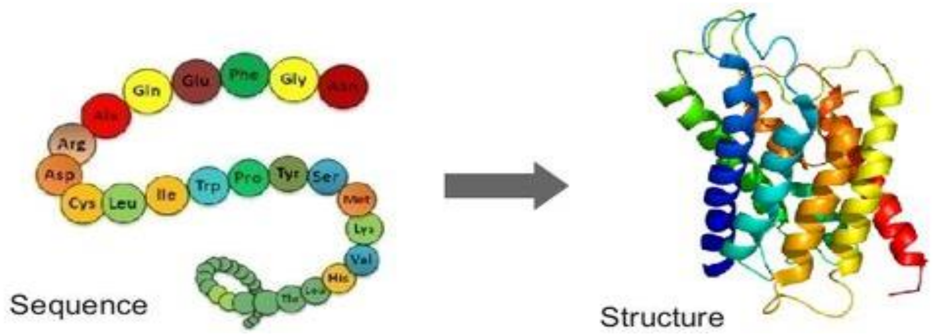

Figure 3.1: Proteins are made up of a chain of amino acids known as a sequence. The chain folds on itself to form a 3D structure which is essential to the function of the protein

There are about 20 different amino acids found in plants and animals. Eight of these amino acids are called 'essential amino acids' because the adult human body cannot make them by itself and so must consume them in food. The remaining amino acids are called 'non-essential amino acids' because the body can make them itself and so they do not need to be eaten.

Essential amino acids		Non-essential amino acids	
**Leucine**	**Methionine**	**Arginine**	**Proline**
**Isoleucine**	**Phenylalanine**	**Aspartic acid**	**Serine**
**Valine**	**Tryptophan**	**Cysteine**	**Tyrosine**
**Threonine**	**Lysine**	**Glutamic acid**	**Asparagine**
		**Glutamine**	**Selenocysteine**
		**Glycine**	

Table 3.3: Essential and non-essential amino acids

As all cells in both plants and animals contain proteins, amino acids will be found in almost all foods. However, different foods contain different amounts and different combinations of amino acids. Protein from animal sources (meat, fish, eggs and dairy products) contains the full range of essential

amino acids needed by the body. However, proteins from plant sources contain only some of the essential amino acids which means that vegans and populations who do not have sufficient animal sources of protein, must combine different plant sources of proteins to get the full range of essential amino acids, for example by combining lentils and rice.

3.3 Fat

We all need some fat in our diet to help provide energy and to provide a small amount of essential fats that our bodies cannot make. Also some vitamins are called fat-soluble vitamins which means we can only get them from foods that contain fat (Vitamins A, D, E and K are fat-soluble).

As stated earlier, over 50% of our energy comes from carbohydrates, 15% of our energy should come from protein and the remainder which is about 30% should come from fat. However, as fat contains a lot of energy (9kcal per gram) this equates to only 95g of fat per day for an average man and 70g fat for an average woman.

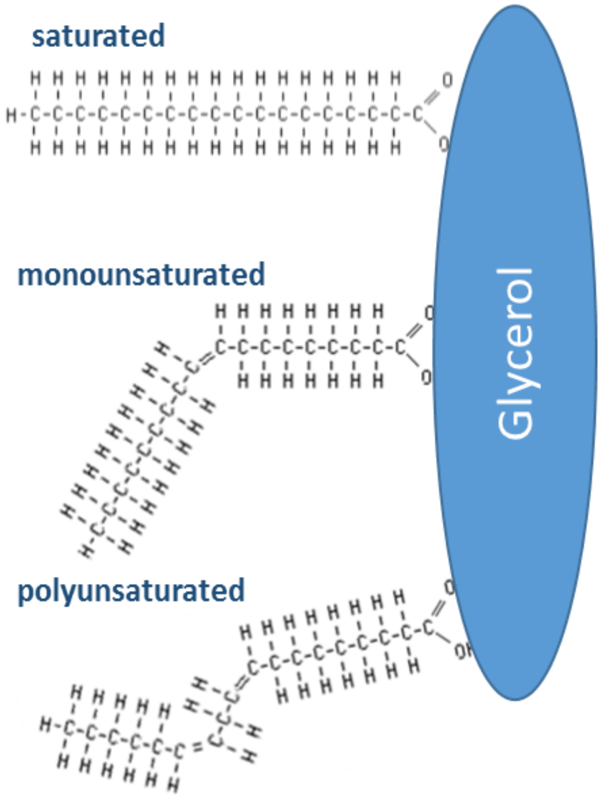

Figure 3.2: A triglyceride composed of a glycerol molecule with three fatty acid chains attached. This diagram shows a triglyceride with a saturated, a monounsaturated and a polyunsaturated fatty acid attached.

When we talk about fat we are actually talking about fatty acids which are chains of carbon and hydrogen atoms. They travel around the body and are stored as a group of three fatty acids attached to a glycerol molecule; together this is called a triglyceride.

Fatty acids can be classified into 3 groups: saturated, monounsaturated or polyunsaturated.

A saturated fatty acid has no double bonds in the chain which leaves it straight. A monounsaturated fatty acid has one double bond which leaves it with a bend in the chain. A polyunsaturated fatty acid has more than one double bond in the chain which leaves it with more than one bend.

When we consume fat in food there will be all of these types of fatty acid present but we classify the foods based on the type of fat that is present in the highest quantity. For example, we call butter a saturated fat because it contains more saturated fatty acids than monounsaturated and polyunsaturated fatty acids.

The majority of the fatty acids that our body needs to make cell walls and signalling molecules can be made by the body. There are two fatty acids that we cannot make and so are called essential fatty acids because we have to eat them. They are both polyunsaturated fatty acids: omega-3 and omega-6. (The names are simply a way to identify where the double bonds lie in the chain).

3.3.1 Dietary sources of fat

Saturated fat is considered unhealthy, they are often solid at room temperature and come from animal sources. There are a few exceptions such as palm oil and coconut oil which are saturated fats from plant sources.

Monounsaturated and polyunsaturated fats are considered healthier and are often liquid at room temperature. They come from plant sources and oily fish.

Saturated fat is considered unhealthy because it raises cholesterol in our blood which leads to high blood pressure and cardiovascular disease. Monounsaturated and polyunsaturated fats don't raise cholesterol and may lower cholesterol if used instead of saturated fat.

However, all fats, whether they are saturated or not, are high in calories (9kcal per gram) so if eaten in large quantities they will all contribute to weight gain.

Type of fat	Main sources
**Monounsaturated**	Olive, rapeseed, peanut (groundnut) oils and spreads made from these. Nuts and nut spreads. Avocados
**Polyunsaturated fats**	
**Omega-6**	Vegetable, sunflower, corn and soya oils and spreads made from these.
**Omega-3**	Oily fish. Flaxseed, rapeseed and soya oils. Walnuts and green leafy vegetables
**Saturated fats**	Butter, ghee (and foods made from these such as cakes, biscuits, puddings, mithai, pastries and pies), dairy products (cream, full fat milk, cheese, full fat yoghurt), meat and meat products (sausages, burgers).Palm and coconut oil

Table 3.4: Types and sources of dietary fat

Trans-fat: You may have heard of another group of fatty-acids called trans-fats (or hydrogenated vegetable oil). This type of fat is found mostly in processed foods. It is made by changing the structure of the unsaturated fats into something that resembles a saturated fat and is used by the food industry because it gives a better mouth-feel and a longer shelf-life. Trans-fats are considered unhealthy, just as saturated fat is. It should be eaten as little as possible.

3.4 Energy

3.4.1 What is energy and where does it come from?

Energy is a difficult concept to understand because it can exist in a number of forms such as light and heat, electrical, mechanical, chemical (such as that found in food), or nuclear energy. It is often referred to as the capacity or power to do work, such as the capacity to move an object by the application of force.

The most important thing to remember about energy is that it cannot be created nor destroyed but it can be transformed into any of its different forms. (Note: This will be important later in this chapter when we discuss energy balance in relation to body fat)

All life on earth needs energy to exist. Energy is needed to perform internal chemical processes, to grow, to move and to reproduce. Without energy there is no life.

The sun is the source of energy (in the form of light energy) for all living things on this planet. Plants can directly capture the energy from the sun. Animals can't do this and so they must eat plants (or other animals that have eaten plants) to get the energy from the sun indirectly.

Plants capture the sun's energy and trap it in their cells in the form of glucose through a process called photosynthesis. Photosynthesis is the process by which plants (and some algae and bacteria) combine carbon dioxide and water (CO_2 from the air and H_2O from the soil) and energy from the sun into a usable form of chemical energy called glucose which they can store and use when necessary. In order to ensure the water in their cells does not turn into a thick sugar syrup with all the glucose production, plants join thousands of glucose molecules together to form starch granules which allows the water to remain free around the granules. Now that plants have a source of energy (and access to minerals in the soil) they can also create fats and proteins which are essential to their structure and growth.

Animals cannot use the sun as an energy source because they cannot photosynthesise. Instead animals must eat plants (or eat other animals that have eaten plants) to obtain the chemical energy in glucose, fats and proteins and store them in their own cells for later use. As mentioned above, we call those energy providing nutrients (carbohydrate, fat and protein) 'macronutrients'.

Animals (including humans) then use that chemical energy in macronutrients and turn it into heat and movement energy of muscles and to power all the internal processes of the body. We do this by adding oxygen (O_2 from the air that we breathe in) to the macronutrients to 'burn' them. This process is called respiration, and interestingly, it is the exact opposite reaction to photosynthesis. See the diagram overleaf:

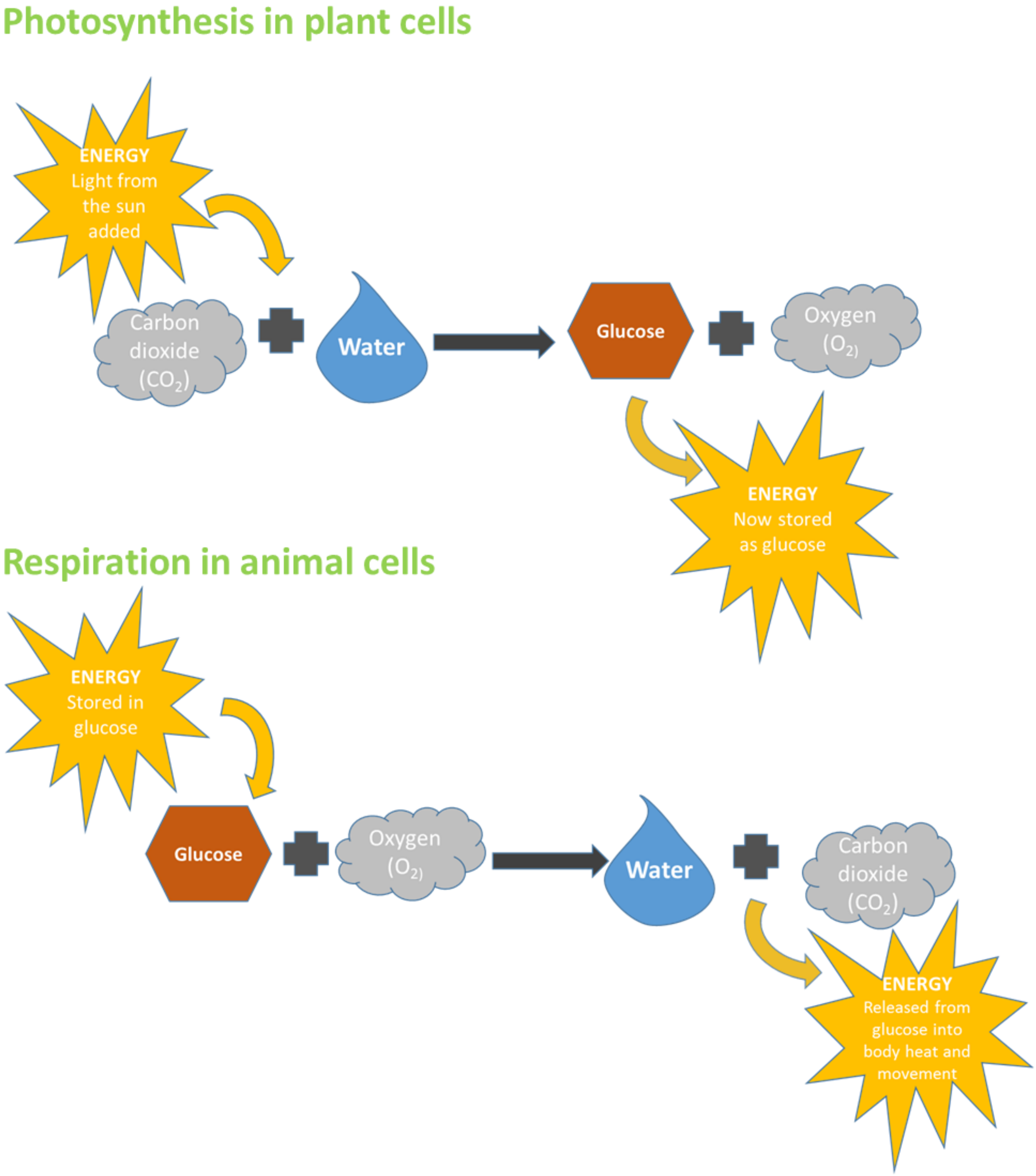

Figure 3.3: Photosynthesis and respiration: Photosynthesis is the process by which plants capture energy from the sun and turn it into glucose to use and to store. Animals then eat the glucose from the plants to use as energy when they burn it (by adding oxygen to the glucose) in their cells. This is called respiration.

3.4.2 Energy balance

Earlier in the chapter we said that energy cannot be created nor destroyed. This is important when we talk about energy balance in the body. We consume energy and we turn it into heat energy when we burn it by moving our muscles and during all the other internal chemical processes that happen in our body all the time. So what happens when we don't burn all the energy that we have consumed? It cannot just disappear or be destroyed, it has to exist somewhere.

Our bodies are very efficient at saving all that unused energy for times when we might need it. The way we save it, and store it, is as fat (also called lipid or triglycerides) inside cells. We have an almost infinite capacity to store fat which means that we will continue to lay down fat as long as we are still consuming more energy than we use up. Most fat is stored in adipose cells in a layer beneath the skin, we call this subcutaneous or peripheral fat. We also store fat around the organs in the abdomen, we call this visceral or central fat. Some people will lay down more central fat which increases the risk of type 2 diabetes more than the people who lay down peripheral fat.

All the macronutrients (carbohydrate, fat, protein and alcohol) that we consume can be turned into fat for storage.

In clinic visit 2 you will discuss the concept of energy balance with your participants using page 2 of participant book 2.

There are four things you need to explain as you work through that page:

1. We take in energy from food (measured as calories or kilojoules) and we burn it to keep our bodies alive and to create movement or exercise. Energy cannot disappear or be destroyed and therefore, if we don't use up all that we consume it has to be stored as fat.

2. When we burn as much as we consume we remain weight stable.

3. When we burn more energy than we consume we lose body weight because our bodies begin to burn the stored fat as an energy reserve.

4. When we do not burn all the energy we consume the excess energy has to be stored as fat and so we put on body weight.

To maintain your weight, the amount of energy you take in from food and drink has to equal the amount of energy you use up in your daily activities.

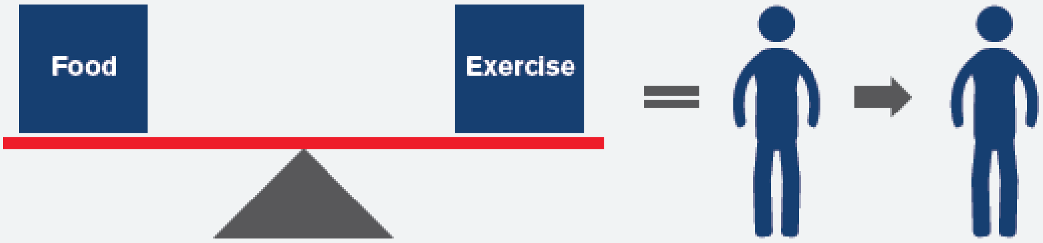

To put on weight, means that over time, the energy you have taken in has been more than the energy you have used up. The difference is usually small, typically only 50-100 extra calories daily.

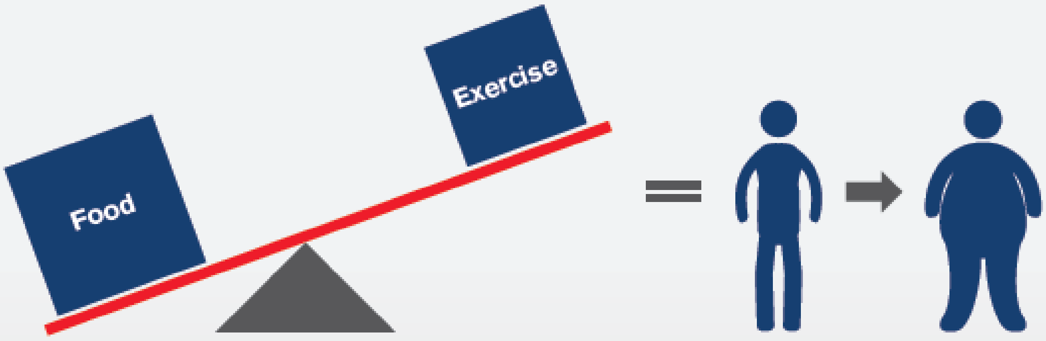

To lose weight, you need to eat fewer calories and/or do more physical activity. Your eating plan has been personally designed for you. By following this plan you should hope to lose at least ½ kg or 1lb per week.

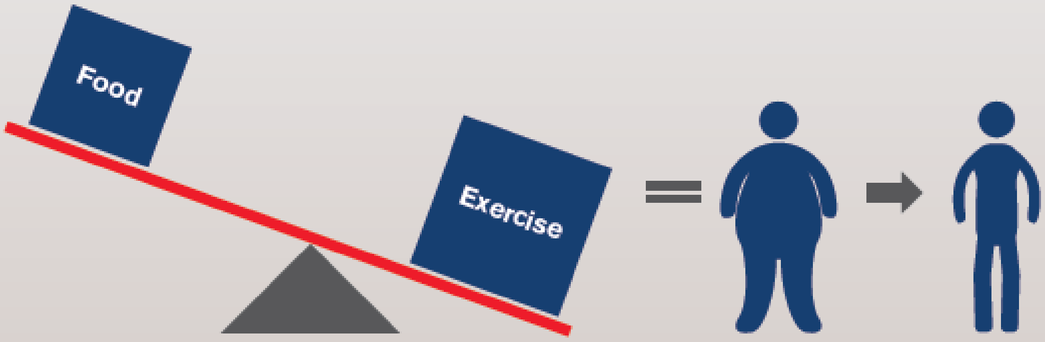

2

Figure 3.4: Page 2 of Participant Book 2: The concept of energy balance.

3.5 Healthy Eating

A healthy diet is one that provides sufficient energy and nutrients to prevent deficiency, optimize health and reduce the risk of disease. It is clear that diet plays a role in the development of many life-threatening conditions such as heart disease, obesity, type 2 diabetes and certain types of cancer as well as those causing debilitating conditions such as constipation, osteoporosis and dental caries.

Healthy eating has many other benefits too. It may help people sleep better, have more energy and better concentration.

To eat healthily does not mean completely giving up foods that you enjoy most. However, some foods should not be eaten too often or in large quantities if you want to be sure of enjoying good health.

Healthy eating is about eating a wide variety of foods in the right amounts to give your body what it needs.

3.5.1 Food groups

Foods are grouped together based on the nutrients they provide. We should eat foods from all of these groups every day. There are 5 food groups:

1. Bread, chapatti, rice, potatoes and other starchy foods
2. Fruit and vegetables
3. Meat, fish, eggs, lentils, soya, beans and non-dairy sources of protein
4. Milk and dairy foods
5. Foods high in fat and/or sugar

Foods included	Amount to be consumed	Main nutrients provided
**Bread, rice and other starchy foods**		
**Bread, rice, chapatis, potatoes, cereals, pasta, noodles.**	- About 1/3 of your plate should be starchy foods<br>- Choose wholegrain versions as they contain more fibre, vitamins and other nutrients than white or refined starchy foods and they can help make us feel full for longer.	Carbohydrate<br>Fibre<br>B vitamins<br>Calcium<br>Iron
**Fruit and vegetables**		
**All types of fruit and vegetables (except potatoes)**	- About 1/3 of your plate should be vegetables/fruit.<br>- These can be fresh, frozen, dried, juiced or tinned.<br>- You should try to eat at least five portions of fruit and vegetables every day	Vitamin C<br>β-carotene (like vitamin A)<br>Folate<br>Fibre<br>Potassium
**Milk and dairy products**		
**Milk, cheese, paneer, yoghurt, fromage frais**	- You should have 2-3 servings per day of e.g. 1/3 pint of milk, a small pot of yoghurt, small piece (40g) hard cheese.<br>-Choose low fat versions when possible.	Calcium<br>Protein<br>Riboflavin<br>Vitamins A<br>Vitamin D
**Meat, fish and alternatives**		
**Meat, fish, eggs, pulses, nuts, seeds and soya products**	- About 2-3 servings per day. - Choose plant sources such as pulses and beans as well as fish and lean meat	Protein<br>Iron<br>B vitamins<br>Zinc<br>Magnesium
**Fat rich and sugar rich foods**		
Fat rich foods:* All oils, butter, ghee, margarines, cream, coconut milk, pastry, fried foods, crisps, chevra, sev, mayonnaise and oil based dressings**<br>Sugar rich foods:* Cakes, biscuits**<br>**Mithai, ice cream**<br>**kulfi, puddings, chocolate and sweets, sherbet, soft and fizzy drinks**	These should only be eaten in small amounts. Swap fatty foods for healthier versions or keep them for special occasions only<br><br>Use food labels to guide you to healthier versions or alternatives.	Some vitamins and essential fatty acids

Table 3.5: Food groups

3.5.2 The Eat Well Plate

The Eat Well Plate is a useful resource that helps people to understand which foods belong to which food group and how much of these foods (shown as a proportion) you should eat to have a well-balanced and healthy diet.

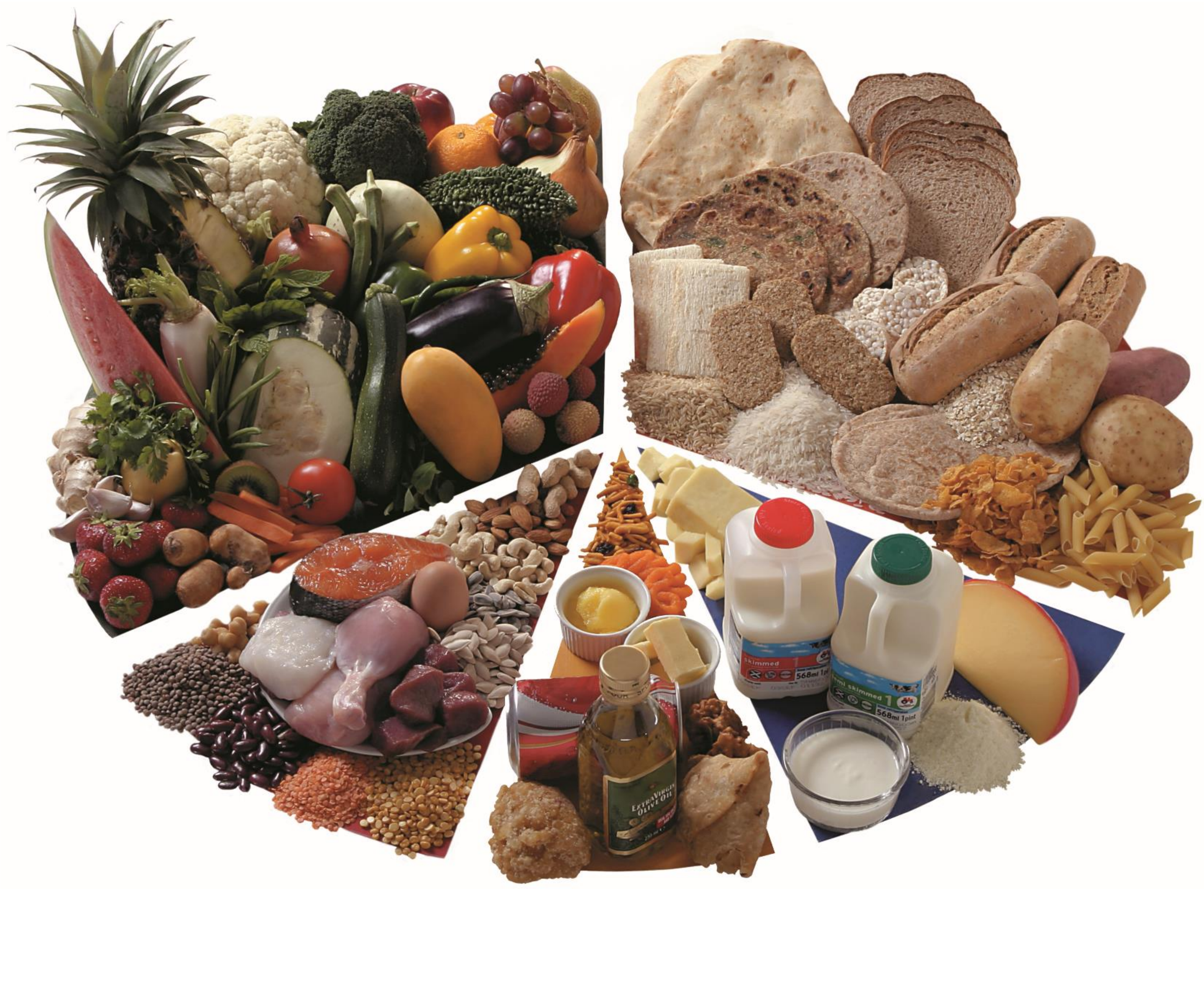

The Eat Well Plate is designed for the general population. However, in iHealth we are often working with people on a weight reducing diet. For a weight reducing diet we alter the proportions in the plate design slightly. At clinic visit 2 you will discuss this with your participant using page 3 of participant book 2. This page shows 3 plates. The first is a ‘traditional’ plate which is the way most people eat at the moment. The second plate represents the Eat Well Plate, which should provide a healthy, balanced diet for the general population. The third plate is the ‘weight reducing’ plate. It consists of a greater proportion of the diet coming from fruit and vegetables and less from the starchy group and the meat and alternatives group, so that it contains fewer calories. The aim is to work with your participant to make dietary changes to move them away from the first plate towards the second plate, and eventually onto the third ‘weight reducing’ plate.

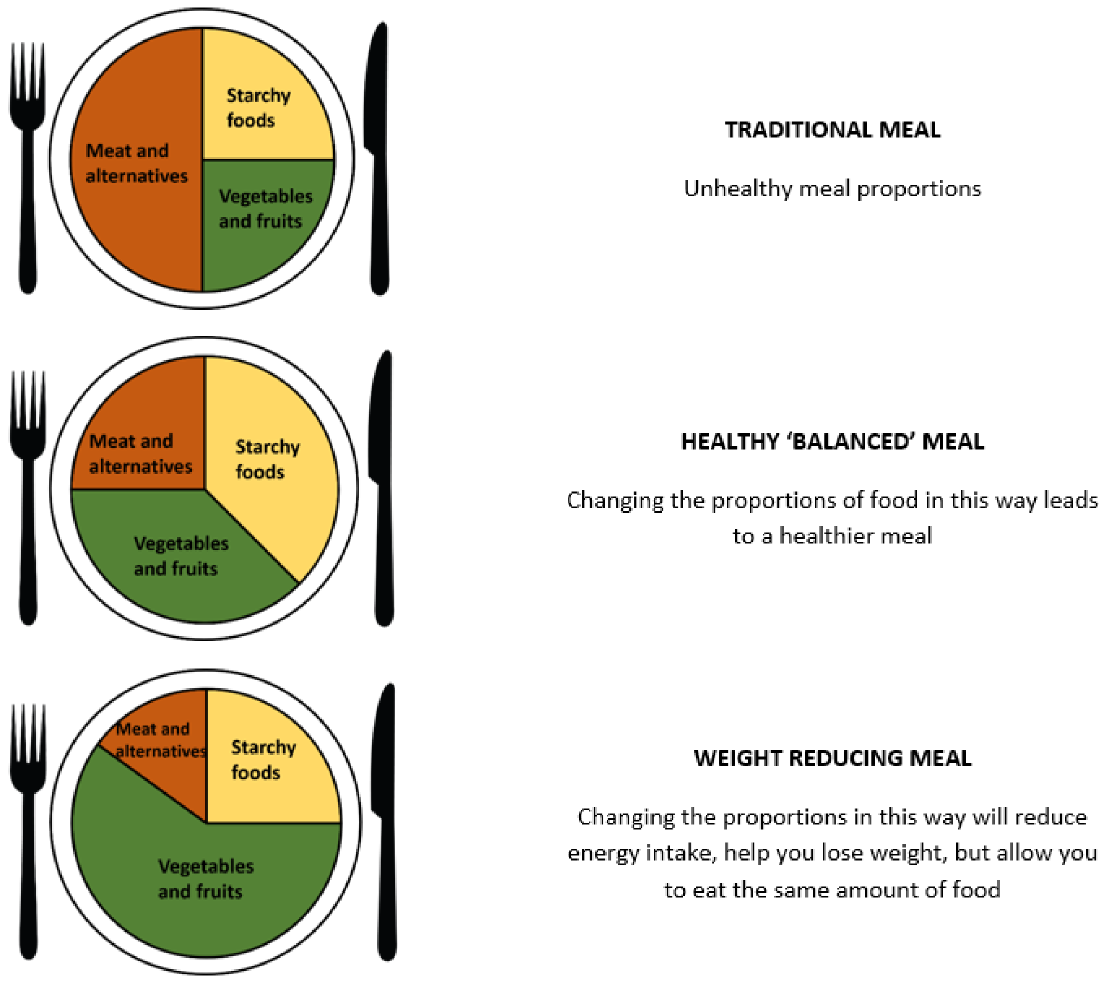

Figure 3.5: Page 3 of Participant Book 2. Food Plates

3.5.3 Healthy eating messages

There are many confusing messages in the media and in communities about what it is to eat healthily, often they are exaggerations or misrepresentations of the facts. There is no single 'super-food' that is the key to a healthy diet, it is all about balance and eating a variety of foods from all the groups. As a health educator it is important that you do not give false information. Keep to the healthy eating messages below:

1. Eat regular meals
2. Eat a range of foods from the food groups to make sure you have a balanced diet
3. Eat the right amount of food for how active you are. Keep an eye on portion sizes. Eat the right amount to be a healthy weight.
4. Eat at least five portions of fruit and vegetables a day
5. Base your meals on starchy foods. Try to choose wholegrain and high fibre varieties
6. Choose lean cuts of meat and try to swap some of your meat for fish and vegetarian sources of protein such as lentils and beans
7. Cut down on fatty and sugary foods
8. Eat less salt
9. If you drink alcohol, drink sensibly. Men and women should not drink more than 14 units in the week.
10. Enjoy your food!

You will encounter questions from participants about something they may have read in the paper or heard from friends about the latest 'fad diet' or 'super-food'. Your response should gently lead them back to the health messages above. Remind them that there is often no evidence to support the claim that they have heard and the best way to lose weight and maintain that weight loss is to make healthy, balanced dietary changes that they will be able to maintain for the rest of their lives. (See Appendix 4 for FAQs about nutrition)

Learning Module 4
Behaviour change

4.0 Behaviour Change

Lifestyle behaviour change is crucial to improving and maintaining good health, whether it is being more physically active or adopting a healthy diet or preferably both. For most people, making a lifestyle change is not easy. Habits and routines that participants are trying to change will have been ingrained over a lifetime. Maintaining change is even harder, so from the very beginning of the intervention emphasis should be placed on how changes will be maintained in the long-run.

Changing behaviour is a process not an event. It takes time and effort. From the outset it is important that the participant understands this. There will be set-backs but together with the CHW they should be able to discover strategies to recover after a set-back and to make the changes longer lasting.

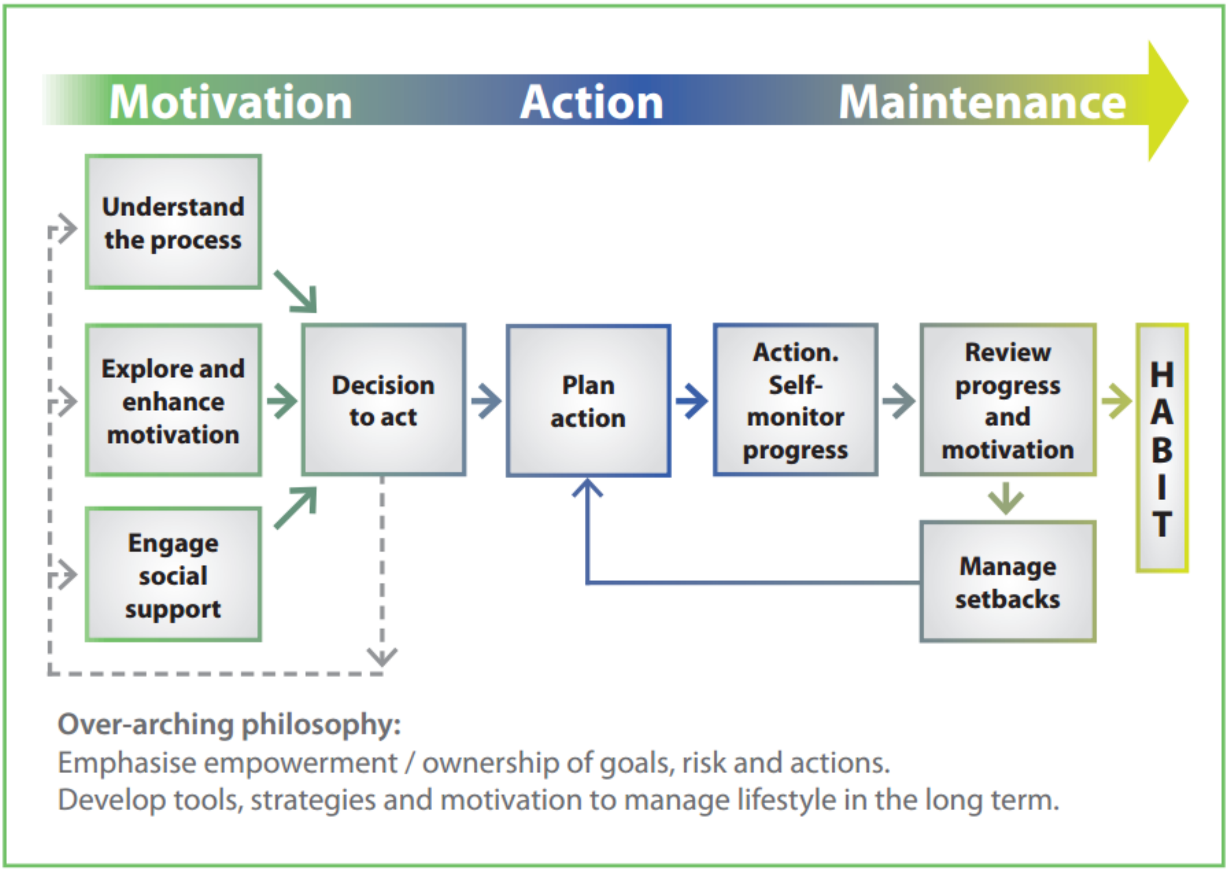

Figure 4.1: A process model for supporting lifestyle behaviour change. (Greaves and Sheppard 2009)

The CHWs role is to support the participant by using a number of behaviour change techniques:

- Person-centred approach
- Information provision
- Exploration and reinforcement of participants' reasons for wanting to change (risks, benefits, barriers and motivations and confidence about making changes)
- Goal setting and action planning
- Record keeping
- Coping plans and relapse prevention
- Encourage family support
- Encourage self-regulation and self-monitoring techniques

4.1 Person-centred approach

Behaviour change must be led by the participant which means using a person-centred approach. Using a person-centred approach, the CHW works in collaboration with the participant as equal partners to decide on the design and delivery of the change. This approach takes into account each participants social, cultural and economic context, their needs for support from family members, their motivation and skills, including any potential barriers they face to achieving and maintaining behaviour change. Person-centred care involves compassion, dignity and respect.

"Behaviour change communication is not a therapy, it is simply a way of arranging conversations with people, so they can hear themselves think and talk themselves into change if they choose"

4.2 Information provision

The CHW should be able to provide information about T2D, the health risks associated with it, and the benefits and practicalities of making changes to diet and physical activity. The participant will be provided with the Participant Booklets each week which aim to provide much of this information.

It is important that CHWs begin a topic for discussion by first understanding what the participant already knows and then filling in the gaps in their knowledge, rather than lecturing.

Topics to be covered:

- Their personal health risks of prediabetes and T2D
- The concept of energy balance
- Benefits of physical activity
- Healthy eating
- Personalised calorie and diet prescription and how to use portion allocation
- What they may expect to encounter during the behaviour change process

4.3 Exploration and reinforcement of participants' reasons for wanting to change (risks, benefits, barriers and motivations and confidence about making changes)

In the first visit, after discussing risks and benefits of making changes or staying the same, the CHW will discuss with the participant their reasons for weight gain which will often give insight into how to tackle weight loss and identify some barriers to change. There is also a scoring system to identify how motivated and how confident the participant feels to be able to make changes at the current moment. Writing down their motivations for change is very important and there is a page in Participant Book 1 for them to do this. The CHW should help to explore these motivations and try to ensure they are specific to the participant rather than generic platitudes.

This introductory discussion will help to identify how ready the participant is to make a lifestyle behaviour change.

4.3.1 The stages of change model

Everyone goes through five stages when changing any ingrained behaviour.

The following tool is based on the work of Prochaska and Diclemente and is used to assess the stage of change the participant is in. Not all participants will be ready to take action to change their lifestyle and simply telling them to do so is unlikely to have much effect. It is the role of the CHW to try to understand each individual participant and to discuss with them their personal set of barriers and motivations and any actions they feel they could implement – no matter how small.

The approach taken by the CHW should be one of understanding and not of judgment. The role of the CHW is to assist the participant in understanding their current situation and how think they would like to proceed.

It is useful to think of the stages of change as a cycle because even the most motivated of people will relapse. Making life long lifestyle changes is a daunting task and very hard to do.

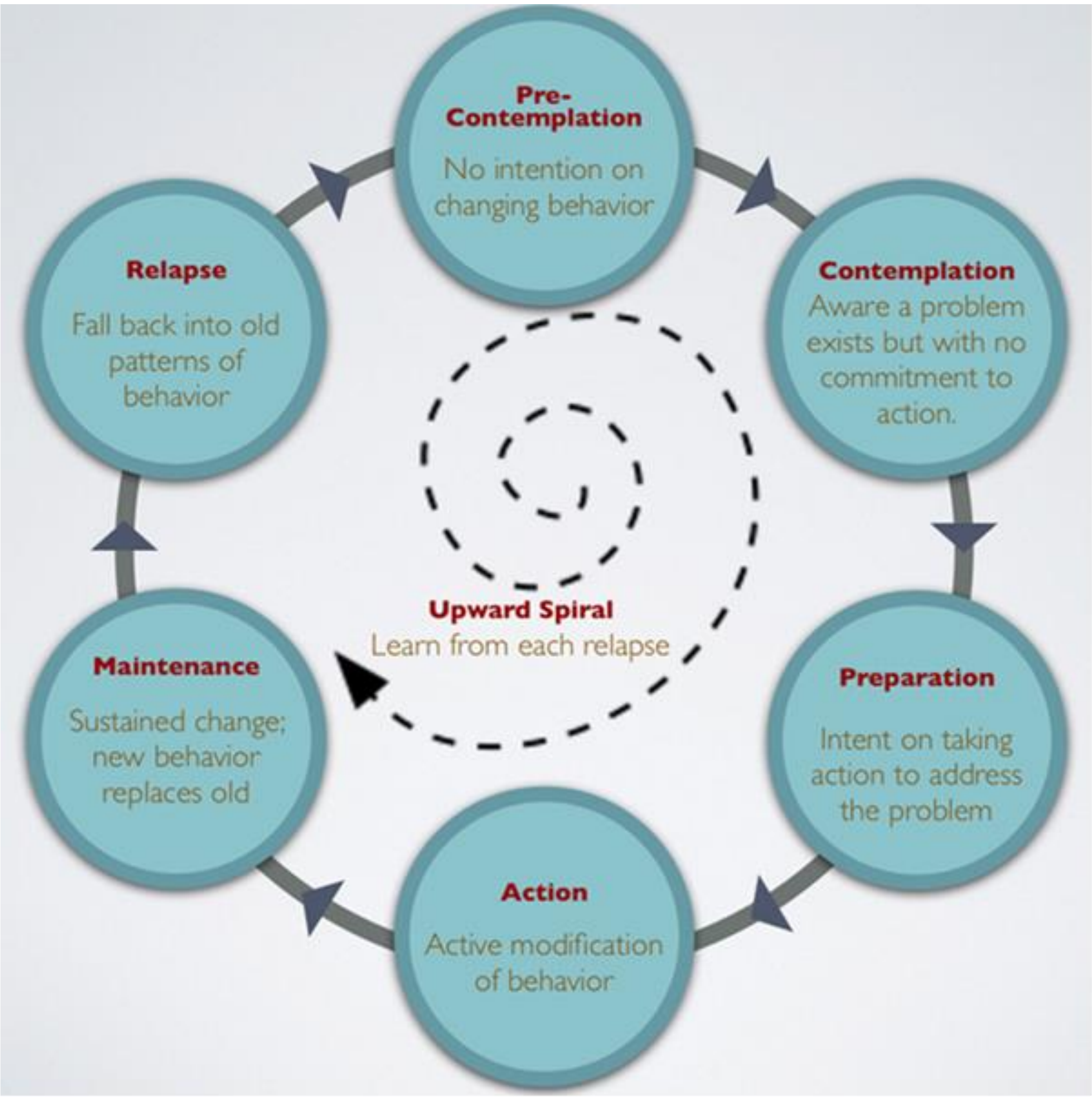

Figure 4.2: Stages of change model

Through your conversations about barriers and motivations you will probably be able to have an idea of where they are on the cycle, however there are some questions which may help.

Q1 In the past month, have you been actively trying to lose weight/not gain weight? (Y/N)
Q2 Are you seriously considering trying to lose weight to reach your goal in the next 6 months? (Y/N)
Q3 Have you reached/maintained your desired weight in the last 6 months? (Y/N)

Stage	Q1	Q2	Q3	CHW actions
**Pre-Contemplation**	N	N		These patients are not considering losing weight at the moment.<br>• Encourage the participant to consider the possibility of change<br>• Raise awareness of the links between diet and health<br>• Give information on personal level of risk<br>• Address any mistaken beliefs<br>• Reassess readiness to change at future appointments
**Contemplation**	N	Y		This group includes those patients who are seriously considering losing weight.<br>• Help the participant explore options for change and identify the best option for them.<br>• Identify 'high-risk' situations and develop strategies to cope with them<br>• Reinforce benefits to the participant of change to develop confidence and commitment.
**Preparation**				• Help the participant explore options for change and identify the best option for them.<br>• Identify 'high-risk' situations and develop strategies to cope with them<br>• Reinforce benefits to the participant of change to develop confidence and commitment.
**Action**	Y		N	Patients who are actively trying to lose weight or have been successful but for less than six months.<br>• Reinforce all changes and encourage.<br>• Give additional support as required.<br>○ Dietary & physical activity advice<br>○ Recipe adaptation & cooking skills<br>○ Understanding food labels & shopping skills<br>○ Goal setting<br>○ Rewards<br>○ Develop strategies for coping with lapses
**Maintenance**	Y		Y	Patients who have successfully maintained their weight loss for at least six months. Reinforce all changes and encourage.<br>• Help the participant consolidate changes and maintain motivation<br>• Revisit benefits of change and highlight achievements<br>• Revisit high-risk situations and further develop coping strategies to prevent lapse & relapse
**Relapse**				• Help see relapse as a learning experience<br>• Discuss trigger to relapse and develop strategies to help overcome this (eg. Consider behaviour chains)

Table 4.1: Questions that can be asked to assess which stage of change a participant is in

4.3.2 Overcoming barriers to change

Barriers provide reasons for not making a change. They are different for each person but once your participant identifies the barriers, you can help them to develop strategies to overcome them and get them on track.

The kinds of lifestyle barriers many people experience include the cost of making the behaviour change, time constraints, family pressures, not having the skills or knowledge to make the change and the temptation to stick or revert to their old lifestyle.

4.3.2.1 Cost

It's a common misconception that a healthy lifestyle has to be expensive. Your participants do not have to enrol in a gym or buy expensive food items; there are plenty of inexpensive options available.

Simple ways to include more exercise that won't cost them money include walking or running outdoors, gardening, taking the stairs instead of the lift and parking the car further from their destination. Money at the supermarket can be saved by buying fruits and vegetables on special offers or buying only those that are in season. Encourage them to try local market stalls and traders; and if they go later in the day, they are more likely to pick up end-of-the-day bargains. Suggest using leftovers. Leftover vegetables can go into soup, meat into dhals, and stir fries, and over-ripe fruit is perfect when blended to make a smoothie.

4.3.2.2 Time constraints

For many people, finding time to exercise and prepare healthy meals will seem like an impossible challenge. However by suggesting minor changes to their daily routine and a bit of pre-planning this too can be overcome.

4.3.2.3 Friends/family pressure

The social pressures to eat and drink, which come from family members, friends and work colleagues, may make it difficult for the participant to stick to their plan (see pg on dealing with pressures to eat)

4.3.2.4 Skills and knowledge

Even a person with strong motivation to change will not be able to make changes effectively without appropriate skills and knowledge. This is where the CHW can step in and steer their learning regarding nutrition, physical activity and behaviour change.

4.4 Goal setting and action planning

4.4.1 SMART goals

Most people know what they should be doing for better health but actually doing it gets difficult. Goal setting is a powerful technique that can help participants lose weight. They can have big and small goals but it is important to break any big goals down into small practical steps, each of which will take them a little way towards their overall goal of losing weight and doing more physical exercise. At each visit, encourage the participant to decide upon a health behaviour they want to change, we call these personal targets. Your role is to help the client set a target that is detailed and likely to be achieved. Targets should be SMART:

**Specific**	Specific – some targets can be vague and difficult to measure. It is important to set targets that are clear and precise. A vague target would be 'being fit and athletic', whereas a specific target would be "I will work out at the local gym for at least 30 minutes twice a week at 7pm on Monday and Thursday." You can ask the four W's to help make it specific:<br>**What are you going to do?** **When are you going to do it?**<br>**Where are you going to do it?** **With whom are you going to do it?**
**Measurable**	Measurable – making the target specific means that it should be easy to measure whether or not the participant has achieved it. The example above is measurable. The participant can record the number of times they went to the gym in one week, and also how long they worked out for each time.
**Achievable**	Achievable – set targets that are within the participant's reach. Failing to achieve a target can have a negative effect on their motivation to work towards their target. An unrealistic target could be 'to stop eating chocolate or sweets'. A more realistic target could be 'reduce the amount of chocolate or sweets I eat to no more than 3 times in the next seven days'. It is important to make the first targets achievable to boost the participant's self-confidence and encourage them to continue. Participants should remember that the best way of changing behaviour and maintaining change is to build on small successes.
**Relevant**	Relevant – does the participant think that the target is relevant to them? You will probably have covered this with the participant during your discussion, however, you need to check with your participant that they see a clear link between their target and their health or how they feel, and that it is a behaviour that they want to change.
**Timely**	Timely –set a time frame in which the target can be achieved. If you don't set a target date for the completion, it could go on and on without the participant ever achieving it. If your next session with the participant is a week away, aim for the target to have been completed by that time. If the target requires a longer time frame, decide together whether there are any smaller targets that the participant could achieve in time for the next session.

Table 4.2: SMART targets

4.4.2 Rewards

People don't often think of rewarding themselves with treats, but this is an effective way of changing behaviour. Adding a reward onto the goal set can have a positive effect on their motivation to change their behaviour, and on increasing the likelihood that the behaviour will occur.

Rewards are referred to as positive reinforcement. This term means using rewards to increase the chance that a behaviour will be performed. Ask the participant what kinds of things they would like as rewards for achieving mini-targets and main goals. Rewards don't have to cost money. Participants could also 'save up' for rewards, eg, put aside a small amount for every day they do some physical activity, and spend the money on a reward at the end of the week/month. Here are some examples:

Rewards that don't cost money	Rewards that cost money
• Having a relaxing bath<br>• Borrowing a book or magazine<br>• Inviting friends round<br>• Having some 'me' time<br>• Listening to music<br>• Going for a walk<br>• Watching your favourite TV programme<br>• Doing some gardening<br>• Asking friends or family to look after your children so you can have some time for yourself • Asking friends or family to notice and praise you when you have achieved something	• Buying yourself a CD/magazine<br>• Buying yourself new clothes<br>• Going to the cinema/football match/concert/theatre<br>• Buying yourself flowers<br>• Buying yourself sports equipment<br>• Going out for a meal<br>• Renting a DVD<br>• Booking a holiday or weekend break<br>• Buying yourself some perfume/aftershave.

Table 4.3: Rewards

Remember: Try not to choose rewards that are too unhealthy.

4.5 Record keeping

Strongly encourage participants to keep records for 6 months.

People who keep records tend to lose weight and maintain that weight loss better than those who don't.

At visit 1, participants will be provided with a 7 day food and activity diary and asked to fill this in every day and bring it back in visit 2 so they and the CHW can review it together.

Ask them to record 3 things:

- Food
- Physical activity
- And any associated feelings or incidents around that occasion. For example it could be emotional (eg. I felt sad so I ate chocolate) or situational (eg. There were biscuits in the office and so I ate them)

Strongly encourage participants to continue to keep daily records for 6 months in a note book that they carry with them. Encourage this at every visit.

Record keeping is useful for a number of reasons:

- Whilst filling in the record, it helps the participant to be aware of the decisions they are making at every eating occasion

- Before a change is made, record keeping helps to identify where they would like to make a change in their habits.

- After making a change, record keeping helps them to see whether that change has been made.

The SMART targets mentioned above are also an important part of record keeping.

4.6 Coping plans and relapse prevention

4.6.1 Triggers

For many people there are particular foods or situations which cause them to lose control over their eating. Sometimes it is not what they eat, but when and how they eat it that causes them to lose control. Examples of some common trigger situations include:

- I eat very little all day, but snack all evening
- I often eat while watching TV
- I often finish off whatever is left on the children's plates
- I often eat out with friends after work
- I eat on my way home from work
- I eat immediately when I come home from work

Identifying trigger situations is the first step towards coping with them.

4.6.2 Eating behaviour chains

A trigger can lead to a sequence of events or behaviours that finishes with eating a food. This is called a behaviour chain. Try to encourage participants to break down these behaviour chains and identify where they can break the chain and therefore plan for future occurrences when that behaviour chain is triggered again. Use Participant Book 4 to work through the exercises with the participant.

4.6.3 Dealing with pressures to eat

There will always be occasions when it may be more difficult for the participant to stick to their healthy eating plan. It could be holidays, parties or even a hard day at work.

These situations can be challenging for the participants. Your role as the CHW is to encourage a *problem solving approach* to these situations.

The problem solving approach has 3 elements:
1. Encourage them to stop and think about the problem
2. Encourage them to look at their options
3. Encourage them to respond positively and assertively

Work through the section in Participant Book 5 with the participants.

4.6.4 Thought patterns

Negative or unhelpful thought patterns can often be a barrier to making positive lifestyle changes.

Sometimes people give themselves too hard a time about trying to lose weight and set impossible targets and deadlines. This can lead to them feeling unhappy with themselves and their abilities to achieve change of any kind. They fall into the trap of expecting too much too soon. This type of thought process is called an “attitude trap”.

Recognising their negative (and often emotional) thought processes is essential to be able to acknowledge them and rearrange them into more helpful, rational thoughts which can influence behaviour for the better.

4.6.5 Eating out

Eating out usually means we have little control over how food is prepared or how large the portion is. If the participant is having the odd unhealthy meal when eating out is unlikely to make a difference to their weight loss plan in the long run. But these days it’s not just the special occasion meals we eat away from home; many of us are eating out more than ever. If the participant eats out regularly (twice a month or more) the choices they make are important and will affect their ability to lose weight. If however they rarely eat out (once a month or less) it will have less of an impact on their progress.

4.6.6 Shopping

A trip to the shops to buy food sounds simple but often people buy far more than they originally planned. To help avoid this and to encourage participants make healthier choices suggest:

- Plan ahead and write a list. (This could save time and money too)
- Try not to shop when hungry, stressed, tired, angry or depressed.
- Be cautious about “bargains”. Special offers may save some money but these foods may be high in saturated fat, salt or sugar and may be provided in larger quantities.
- Know food labels. Being able to understand food labels can help the participants make more informed choices about packaged foods.

4.7 Encourage family support

A supportive atmosphere is important. Eating is a highly social activity. By sharing their goals with others, they will move a step forward in enlisting their support. It is often easier to make lifestyle changes at the beginning when the participants are highly motivated, but as time goes on, they will find it easier to stick to their resolve if they surround themselves with people who respect their goals and aspirations. Their support network can take different forms, such as:

- Joining the participant in exercise or eating healthily. (Changing the lifestyle of the household can be easier than having to do it alone)
- Practical help such as freeing up the participants time so that they can do exercise
- Encouragement: Offering praise or reward for trying to change (whatever the result) and support when the participant is finding it difficult to stay motivated
- Being interested: Simply by asking about their progress or listening when they describe the goals they are trying to achieve or have achieved can be positive

4.8 Encourage self-regulation and self-monitoring techniques

The aim of self-monitoring and self-regulation is to enable the participant to maintain their healthy lifestyle changes for life, building self-sufficiency during the second half of the intervention when they have fewer contacts with the CHW and eventually after the intervention when they have no contact with the CHW.

Self-monitoring: This should be encouraged from day 1. Participants will be encouraged to record what they eat and do every day, to weigh themselves and measure their waist circumference regularly and to monitor their SMART targets.

Evaluating and reviewing these records informs which changes are working and what future changes can be implemented. At the beginning of the intervention the CHW should work with the participant to enable them to start to recognise and review their own progress towards their goals. Reviewing what went wrong and what went well is important and will give the participants greater self-knowledge and will help them to plan how to achieve their next goal.

Self-regulating:

Self-regulation involves a number of things such as developing ways to maintain their own motivation, being able to recognise triggers and eating behaviour chains and pressures to eat and developing ways to break these habits and coping strategies.

Learning Module 5
iHealth

5.0 Introduction to iHealth

This study aims to determine whether an intensive, year-long family-based lifestyle intervention delivered by community health workers (CHW) versus usual care is clinically- effective and cost-effective for prevention of type-2 diabetes (T2D) amongst South Asians with central obesity and/or prediabetes, on the Indian subcontinent and Europe.

The results will provide an evidence-based strategy for efficient, effective, equitable, sustainable and scalable implementation of lifestyle modification to promote health and prevent T2D amongst South Asians in diverse settings, and thereby help reduce the global burden of T2D in this high-risk population.

This programme has been developed to provide a framework for the delivery of the family-based lifestyle modification. It is based on good practice and aims to establish a more consistent and participant-centred approach.

The programme lasts 12 months and includes a total of 22 contact points with the participant, nine of which are face-to-face clinic appointments and 13 of which are telephone contacts. Other family members are encouraged to attend the clinic appointments, particularly if they are overweight or the main cook of the family unit.

The programme consists of two phases:

Weight Loss Phase: Week 0-19 comprises six sessions. The first two visits are face to face sessions with the index case (and any accompanying family members), followed by three group sessions and completed with a face to face review session with the index case (and accompanying family members). In between each visit the index case will be contacted by telephone so that they have contact with their CHW approximately every two weeks.

Weight maintenance phase: Week 20-49 comprises 3 sessions. Two are group sessions and the final visit is a face to face review session with the index case (and accompanying family members). In between visits the index case will be contacted by telephone so that they have contact with their CHW approximately every 3 weeks.

Staff	Time	Episode	Length	Visit type
Nurse	Month -1	Episode 1	1h	Screening
Nurse	Time 0	Episode 2	1h	Enrolment
CHW	week 1	Episode 3	1.5h	CLINIC -Face-face individual/family consultation
CHW	week 2	Episode 4	15min	Telephone call
CHW	week 3	Episode 5	1.5h	CLINIC -Face-face individual/family consultation
CHW	week 5	Episode 6	15min	Telephone call
CHW	week 7	Episode 7	1.5h	CLINIC- Group Session
CHW	week 9	Episode 8	15min	Telephone call
CHW	week 11	Episode 9	1.5h	CLINIC- Group Session
CHW	week 13	Episode 10	15min	Telephone call
CHW	week 15	Episode 11	1.5h	CLINIC- Group Session
CHW	week 17	Episode 12	15min	Telephone call
CHW	week 19	Episode 13	1.5h	CLINIC -Face-face individual/family consultation
CHW	week 21	Episode 14	15min	Telephone call
CHW	week 23	Episode 15	15min	Telephone call
CHW	week 25	Episode 16	1.5h	CLINIC- Group Session
CHW	week 28	Episode 17	15min	Telephone call
CHW	week 31	Episode 18	15min	Telephone call
CHW	week 34	Episode 19	15min	Telephone call
CHW	week 37	Episode 20	1.5h	CLINIC- Group Session
CHW	week 40	Episode 21	15min	Telephone call
CHW	week 43	Episode 22	15min	Telephone call
CHW	week 46	Episode 23	15min	Telephone call
CHW	week 49	Episode 24	1.5h	CLINIC -Face-face individual/family consultation
Nurse	Month 12	Episode 25	1h	Follow-up 1
Nurse	Month 24	Episode 26	1h	Follow-up 2
Nurse	Month 36	Episode 27	1h	Follow-up 3

Table 5.1: Timetable of 12 month lifestyle-modification programme

Staff	Time	Episode	Length	Visit type
Nurse	Month -1	Episode 1	1h	Screening
Nurse	Time 0	Episode 2	1h	Enrolment
CHW	week 1	Episode 3	1.5h	CLINIC -Face-face individual/family consultation
Nurse	Month 12	Episode 25	1h	Follow-up 1
Nurse	Month 24	Episode 26	1h	Follow-up 2
Nurse	Month 36	Episode 27	1h	Follow-up 3

Table 5.2: Timetable of the ‘usual care’ programme

5.1 Aims of the Family-based Lifestyle Modification Programme

1. To advise on behaviour modification to help reduce or prevent the medical consequences of central obesity and/or prediabetes.
2. To advise, motivate, support and educate participants on ways to achieve negative energy balance, thus leading to weight loss.
3. To advise on the goal amount of weight to be lost and a realistic time-scale within which to lose this weight (normally a 10% reduction in 6 months).
4. To advise on weight maintenance and the prevention of weight gain.
5. To encourage improvements in activity levels to support weight loss
6. To encourage the participation of other family members, particularly those who are also overweight and/or the main cook of the family.

5.2 Participants

Each of the four participating countries (India, Pakistan, Sri Lanka and UK) has identified 30 field-work sites which cover a range of socio-economic settings. Each field work site will recruit 30 South Asians to the study (15 male, 15 female, free from T2D) who have central obesity (waist ≥100cm) and/or prediabetes (HbA1c ≥6.0%). Thus 3600 index cases will be recruited to the study (N=2700 on the Indian subcontinent, N=900 in Europe).

Inclusion criteria: Waist circumference: ≥100cm OR HbA1c: ≥6.0%. South Asian, Male or Female, and age 40-70 years

Exclusion criteria: Known type 1 or 2 diabetes, fasting glucose: ≥7.0 mmol/L or HbA1c ≥6.5%; normal or underweight (body mass index<22kg/m2); pregnant or planning pregnancy; unstable residence or planning to leave the area; serious illness.

5.3 Role of the community health worker

- Retain a visible clinical presence with responsibility for the specific protocols related to the study and caseloads of patients
- To assess the patients understanding of the informed consent process prior to any study related procedures
- To deliver the diet/nutrition component of the intervention
- To educate and counsel patients, and family members
- To maintain accurate and accessible records
- To maintain patient confidentially at all times
- To discuss any concerns raised by patients and family with senior staff
- Maintain effective communication with all members of the team
- Actively participate in and support the research team

5.3.1 Community Health Worker competencies

5.3.1.1 Knowledge

- Thorough understanding of the protocol
- Background knowledge of T2D, diabetes prevention, nutrition and physical activity, behaviour change techniques.

5.3.1.2 Key skills

- Relationship building
- Active listening
- Keeping accurate notes of each visit
- Calculate estimated energy expenditure
- Able to adapt a diet to suit an individual
- Conduct a face-face session
- Conduct a group session
- Conduct telephone session
- Anthropometrics
- Conducting a 24 h recall
- Obtain consent from family members
- Administering GPAQ
- Database entry/accurate record keeping

5.3.2 Data recording by the CHW

The tables below are an example of the clinic report form (CRF). It is essential to the study that these are filled out accurately by the CHW at each contact. The white boxes indicate that a figure must be inputted. Greyed out boxes can be ignored for that visit.

Also in the CRF you will find space to record:

- 24h food recalls at visit 1, 2, 6 and 9
- physical activity questionnaire (GPAQ) at visit 1 and 6
- diet history at visit 1
- notes and targets set/reviewed for every visit
- notes from each phone call

The notes the CHW makes in the CRF for each visit and phone call are very important. The purpose is so that you (or a colleague) can see what was discussed each week. This could be barriers, concerns, achievements etc. The targets the participants set at each visit must be recorded too so that they can be reviewed the following visit – you cannot rely on the participant remembering to bring their participant books.

Clinic visit	Visit 1	Visit 2	Visit 3	Visit 4	Visit 5
Visit type	121	121	Group	Group	Group
Date	DD/MM/YY	DD/MM/YY	DD/MM/YY	DD/MM/YY	DD/MM/YY
CHW ID					
**Participant**					
Weight (kg)					
Waist (cm)					
Hip (cm)					
Importance scale (1-10)					
Confidence scale (1-10)					
**Lifestyle data collection**					
24 hour recall completed	Y/N (p21-22)	Y/N (p23-24)			
FFQ completed	Y/N (p28)				
GPAQ completed	Y/N (p30-34)				
7 day food diary collected		Y/N			
**Treatment**					
Targets set / reviewed	Y/N (p4)	Y/N (p6)	Y/N (p7)	Y/N (p8)	Y/N (p9)
Lose / maintain weight					
Calorie prescription (kcal)					
Menu plan	Standard / Personal	Standard / Personal	Standard / Personal	Standard / Personal	Standard / Personal
**Materials given out**					
Participant booklet	Y/N	Y/N	Y/N	Y/N	Y/N
Tape measure	Y/N				
7 day food diary	Y/N				
**Relatives**					
Family members attended	Y/N	Y/N	Y/N	Y/N	Y/N
Family data recorded	Y/N (p40)	Y/N (p40)	Y/N (p40)	Y/N (p40)	Y/N (p40)

Table 5.3: Clinic report form (CRF) visit 1-5

Clinic visit	Visit 6	Visit 7	Visit 8	Visit 9
Visit type	121	Group	121	121
Date	DD/MM/YY	DD/MM/YY	DD/MM/YY	DD/MM/YY
CHW ID				
**Participant**				
Weight (kg)				
Waist (cm)				
Hip (cm)				
Importance scale (1-10)				
Confidence scale (1-10)				
**Lifestyle data collection**				
24 hour recall completed	Y/N (p25-26)			Y/N (p27-28)
FFQ completed				Y/N (p29)
GPAQ completed	Y/N (p35-39)			
7 day food diary collected				
**Treatment**				
Targets set / reviewed	Y/N (p10)	Y/N (p12)	Y/N (p13)	Y/N (p14)
Lose / maintain weight	Lose / Maintain			Lose / Maintain
Calorie prescription (kcal)				
Menu plan	Standard / Personal	Standard / Personal	Standard / Personal	Standard / Personal
**Materials given out**				
Participant booklet	Y/N	Y/N	Y/N	Y/N
**Relatives**				
Family members attended	Y/N	Y/N	Y/N	Y/N
Family data recorded	Y/N (p40)	Y/N (p40)	Y/N (p40)	Y/N (p40)

Table 5.4: Clinic report form (CRF) visit 6-9.

5.4 Clinic visit protocols and scripts:

5.4.1 Clinic Visit 1

Overview

1. Hello and introductions
2. Take measurements: Height, weight, hip, waist. 24h recall.
3. Discuss T2D, risks and complications
4. Explain the programme goals for lifestyle intervention
5. Fill in PB1 page 8-11 with participant
6. Discuss record keeping
7. Discuss lapses
8. Take a diet and physical activity history
9. Discuss benefits of physical activity
10. Target setting

You will need:

- CRF
- Family consent forms
- Participant booklet 1
- 7 day food record
- Weighing scales, height chart, measuring tape
- Food portions book and cutlery/crockery

Appointment length: 1-1.5h

Hello and introductions:

- Introduce yourself and your role
- Introduce the study
    - To initiate this you can ask: 'So what do you understand this year intervention involves?'
    - Fill in the gaps in their knowledge based on their response
    - Introduce Participant Book 1 and show them the timetable and where you will write the appointment times for them
- Ask family members if they would like to sign the consent form to take part

Script:
Hello **[NAME]**, my name is **[NAME], how are you today?**

I am your CHW **and we'll be working together for the next 12 months** to help you make lifestyle chan**ges to try to prevent T2D. By lifestyle changes we mean changing health behaviours around healthy eating, physical activity and for some people, weight loss.**

What do you understand about the timetable for this year-long intervention?
There are a total of 22 contacts, 9 will be clinic visits and 13 will be telephone contacts in between those visits.

-The first two sessions are personal sessions where we'll discuss many aspects of your current diet and physical activity and how we might be able to improve that.
-The next three visits are group sessions which you will attend with other people doing this intervention, the aims of the group visits are to spend more time discussing healthy eating and learning to ways to identify and manage the difficulties you may come across whilst trying to make changes to your lifestyle.

-The sixth visit is another personal session. It will be in about 5 months from now and we'll review the progress you've made so far and make a decision about the best way forward for the 2nd half of the year.
-Visits 7 & 8 are group visits again
-Visit 9 is the final visit at around 12 months from now – it will be another review of your progress and a discussion or planning session about how you will go forward now that the intervention period is over.
- The telephone calls in between visits are just an opportunity to discuss how you're getting on and for you to ask any questions you might have.

Do you have any family members who would like to attend the visits with you? We strongly encourage it because it gives you support and it is particularly useful if they are overweight too or the main cook of the family.

PARTICIPANT BOOKLET: This is your participant booklet for the first visit, and here on this page is the timetable of visits and your appointment dates – does this make sense so far? Do you have any questions about the visits?

The participant booklet is for you to keep. You'll get a new chapter at each visit and we'll work through some pages together during the visits, but you can also use it at home as a reference. It should contain most of the information we discuss at each visit. It will be useful for you to bring this back with you for the next visit.

Take measurements:

- Height, weight, waist and hip
- Take a 24h recall
- Record this in the CRF

Script: OK, are you happy if we take some measurements now?
[Follow SOPs for measurements and 24h recall and record in CRF]

Discuss T2D:

- Risk factors:
    - To initiate, ask: 'Why do you think you have been invited to take part in this intervention?'. Respond by filling in the gaps in their knowledge
        - It is because they are at very high risk of developing T2D within the next few years because they have some/all of the risk factors. South Asian, over 40y, high HbA1c, overweight, large waist circumference.
        - However, T2D can be prevented with lifestyle change
- Complications of T2D
    - To initiate: 'Do you know any of the health complications associated with having T2D?' Respond by filling in the gaps in their knowledge
        - Use PB1 page 6

Script:
So, why do you think you have been invited to take part in this intervention?

You are considered to be pre-diabetic which means there is a very high risk of you developing T2D within the next few years. This can be because of a number of things:

- One of the reasons is because you are South Asian. There is nothing we can change about this of course but we do know that T2D is far more common amongst South Asians than other ethnicities. South Asians tend to develop T2D at an earlier age and even if they are not overweight. However it's not really known why this happens.
- Another reason you are at risk of getting T2D is because of your body weight. Here is your BMI, which takes into account your weight and height, and you can see on this chart that you are in the overweight category. People in this category are at a greater risk of getting T2D – in fact being overweight is thought to be the main cause of T2D.
- We also measured your waist circumference which gives us an indication of risk, similar to BMI. Fat around the belly adds to a person's risk of T2D more than fat elsewhere, for example on your hips.

**- You gave a blood sample to the nurse at screening and this may have shown that you have high blood sugar which also puts you at risk of developing T2D.**<br><br>**It's important for you to know that over 50% of people with prediabetes will develop T2D within 5 years unless steps are taken to improve lifestyle management. The good news is that half of all T2D cases are prevented when a healthy lifestyle is adopted. This involves a healthy diet, increased physical activity and weight loss if necessary.**<br><br>**What do you know about the health complications that can occur when you are overweight or have constantly elevated blood sugar levels such as in prediabetes and T2D?**<br><br>**OK, take a look at this diagram [PB1 page 6] which shows the areas of health that can be damaged. Diabetes can be debilitating. High blood glucose levels disrupt the blood flow all around the body. Blood vessels become hard and clots can form which can lead to heart attack and stroke. Eye damage leading to blindness is common as is damage to the nerves causing pain or numbness in the extremities and poor wound healing which can lead to foot amputation. Being overweight can also cause joint pain, particularly in the knees, due to the extra load.**
**Explain the programme goals for lifestyle intervention:**<br>• **10% weight loss**<br>• **At least 150 minutes of physical activity each week**<br>• **Work through and fill in page 7 of PB1 with them**
**Script: The aim of this lifestyle intervention is to do all we can to try to prevent you from developing T2D. There are two overall goals of this intervention that we are encouraging participants to aim towards during the year intervention. The first one is weight loss – even a small weight loss between 5-10% can significantly improve health outcomes. The second aim is to increase physical activity to at least 150minutes eack week. That's 30 minutes a day for 5 days a week. Even without weight loss, just increasing physical activity will have health benefits.**<br><br>**[Work through PB1 page 7] Your current weight is 80kg, so a 10% weight loss for you would be 8kg. The aim is to lose that gradually, about 0.5-1kg per week over the coming few months with our support. How does this sound to you?** Are you happy with this target? If not what **weight loss** target would you like to set yourself?<br><br>**Alongside weight, a reduction in waist circumference is very beneficial to health so we'll monitor that as we go along too.**
**Fill in PB1 page 8-11 with participant**<br>• **Ask the questions and let them speak**<br>• **This is where you will begin to understand their reasons for weight gain (and therefore some of the barriers they may face), the importance and confidence scales (this is how you begin to understand which stage of change they are in and therefore how you approach discussions with them)**<br>• **Record importance and confidence scales in CRF**<br>• **Encourage them to give detail to the answers and help them to shape their motivations to be very specific to them . Eg rather than 'I don't want to get T2D' ask which of the complication of T2D they most want to avoid**
**Script: Let's work together on the next few pages [Fill in PB1 page 8-11].**<br>Losing weight can be very difficult. Understanding the reasons why you have gained weight can provide insight into how best to tackle losing weight. Think about some of these reasons. Let's write them down**…**
**Take a diet and physical activity history**<br>• **Ask what they would eat on a typical day**<br>• **Which meals do they buy outside of the house**<br>• **Which meals do they prepare**<br>• **Who is the main cook**

- **Who does the food shopping**
- **Record this in the CRF**

Script: I would like to find out a bit more about what your current diet and lifestyle is like so can you talk to me about what a typical day looks like to you in terms of what you eat, when you eat and where you eat it? Perhaps start with a typical morning.
Do you do most of the cooking? If not who does and do you think we could encourage them to come along to these sessions with you?
Who does the food shopping?
Are you vegetarian?
Do you drink alcohol?

Discuss record keeping

- **Give them a 7 day food record. It is important they fill this in and bring it to clinic visit 2**
- **Explain that we are strongly encouraging people to keep records for 6 months in a note book that they carry with them**
- **People who keep records tend to lose weight and maintain that weight loss better than those who don't**
- **Record keeping is useful to help them see where they would like to make changes and then when they've decided to make a change it helps them to see whether that change has been made. It's useful for you both to work with**
- **Ask them to also record physical activity and perhaps triggers – these could be emotional (ie. 'I ate this because I was stressed') or situational (ie. ' I ate these biscuits because there are always biscuits in the office')**

Discuss lapses

- **They will inevitably happen. They should be expected.**
- **What happens after the lapse is important**
- **After a lapse try to pick up and carry on**
- **Let them know that they should never feel like they cannot attend the visits because they think they have failed. You are here to work with them to discuss the lapses and try to find solutions with them to avoid similar lapses**

Script: Studies have shown that people who keep records **o**f what they are eating are more likely to succeed at losing weight. Keeping records makes you more aware of what, why and when you are eating. So to get a better insight into your eating pattern, here is a 7 day food dairy which I would like you to fill in for the next week until your next appointment.(go through sample of food diary).Record everything you eat, no matter how big or small. The more descriptive you are the better. We also want you to record triggers that may have led to that eating occasion, for example were you sad, angry or anxious, or situational triggers such as where you were or who you were with that may have led to that eating occasion. It is very helpful to know what triggers a change in your eating habits.

Please bring this 7 day food diary with you when you come for visit 2.This is so you and I can begin to understand your eating habits and discuss aspects that you may wish to change about your diet. This will help us greatly in working out the right plan which suits you and which will help you reach your target.

Be as open as possible with this diary. Losing weight and changing habits is extremely difficult and you should expect to lapse every now and again. That is normal. What is important is that you do not give up because of one lapse. We are working together through the whole year on this, and as we begin to understand perhaps what caused the lapses we can work on solutions to help prevent them more and more. The most important thing is that when you do lapse, you don't dwell on it or give up. All positive changes to your lifestyle are helpful, however small they may seem, one lapse will not undo all of the healthy things that you have been doing before.

Discuss physical activity

- **Explain what physical activity is. Anything that makes you slightly hot and increases your breathing rate.**
- **Ask what physical activity they enjoy and what kind of physical activity they would find easy to introduce into their day/week.**

- **Record this with the diet history in the CRF**

Script: I'd like to discuss physical activity with you now.
Exercise is not just important to help you lose weight but being active has lots of other benefits such as lowering blood pressure, improving cholesterol and importantly it predicts long term success for losing weight and keep it off for good.
Any activity that makes you slightly hot and increases you breathing rate is considered exercise.

What exercise you are currently doing during the day or week? What exercise would you like to do more of or would be easy for you to do more of? [Record in the CRF with the diet history]

As we discussed earlier, one of the aims of this intervention is to work towards doing at least 30minutes a day, on 5 days of the week. It doesn't have to be 30minutes in one go, it can be broken down to 10 or 15 minute bouts. How do you feel about reaching this target?

It is important for weight loss to combine healthy eating and physical activity.

Target setting

- **Ask them what target they would like to set. Explain that for visit 1 we would very much like them to set at a physical activity target. However if they would like to set a dietary target as well, that is great.**
- **Encourage them to set an achievable/easy target this week**
- **Help them to shape their target into a SMART target**
- **Record in CRF and PB2 page 18**

Script: At the end of every session you are going to set yourself a target until the next visit. I am here to help you to frame it so that it is realistic and achievable. We will try to make them very specific so you know exactly what you're intending to do and can keep track of how much of it you have completed.

Do you have something in mind that you would like to begin to change over the next two weeks in relation to diet or physical activity?
If not, give some examples but still let them come up with their own idea. For week one, it is important that they set a physical activity target.
[Record the targets in PB1 page 18 and in the CRF]

Goodbye

- **Remind them to bring their PB and 7 day diary to the next visit**
- **Encourage them to bring family members, particularly the cook of the household**
- **Encourage record keeping**
- **Remind them of the importance of attending all visits**
- **Date of next appointment**

5.4.2 Clinic Visit 2

Overview

1. Welcome back
2. Take measurements: Weight, hip, waist. 24h recall.
3. How have you found the past 2 weeks?
4. Review last week's targets
5. Review 7 day food record
6. Introduce PB2 and this week's topics
7. Discuss energy balance
8. Discuss food groups and food plates
9. Explain the 7 healthy eating messages
10. Work through My Eating Plan and fill in kcal prescription and portions
11. Ask the participant to fill in confidence and importance scales
12. Target setting

You will need:

- CRF
- Family consent forms
- Participant booklet 2
- My Eating Plan and set menu plans
- Weighing scales, measuring tape
- Food portions book and cutlery/crockery
- Food plate mat and BHF food cards

Appointment length: 1-1.5h

Welcome back

Give positive feedback that they have returned

How have you found the last 2 weeks?

Script: Hello again, it's nice to see you. Just turning up to the appointments is a huge thing, it should show you that you have already begun to take steps towards making some changes so well done.
How have you found the last 2 weeks?

Take measurements:

- **Weight, waist and hip**
- **Take a 24h recall**
- **Record this in the CRF**
- **Give positive feedback or reassurance**

Script: Are you happy to take some measurements again?
[Follow SOPs for measurements and 24h recall and record in CRF]
You have lost 0.5kg since the last time, that's great.
Or
You are the same weight as last time but don't worry, it's good that your weight hasn't increased and we can discuss later on why you think you may not have lost what you hoped. This is a long process and we'll build on it each week.

Review last week's targets

- **Record in CRF and PB1 page 19**
- **Give positive feedback for achievements, however small they may be**
- **Non-judgemental**
- **Try to uncover the barriers that prevented them from achieving**
- **Remind them that this is a long process, changing habits is difficult and you will be working with them for the rest of the year**

Script: What targets did you set yourself last week? Did you manage to achieve them in part/fully? Did you have any difficulties? Tell me a bit more about that. *Problem solve with the participant to address any barriers*.

Review 7 day food record

- **Look through the record with them**
- **Ask them if they have identified any patterns or aspects of their diet they think they could change**
- **Highlight any obvious snacking or high calorific foods to them and ask them what they think about it. Try not to suggest they change it – let them lead the changes they want to make**

Script: Did you bring the 7 day food diary? How did you find filling it it? Do you think just the act of noting what you ate made any differences to your diet?
Let's have a look through it. Did you notice any patterns or aspects where you would like to make changes?
If not: OK, so I can see here that you tend to snack in the afternoons – is that something you have noticed?
[You do not have to make changes at this point – it is just a discussion]

Introduce PB2 and this week's topics

Script: Here is your booklet for this week. We'll be talking about healthy eating today and some of the concepts behind weight loss. After that we'll go on to develop your own eating plan. Does this sound ok?

Discuss energy balance

- **Use PB2 page 2**

Script:
Energy cannot be created or destroyed – it is just moved around. We take in energy from food and we use up that energy in daily living and when we exercise. If we don't use it all up, the energy has to be stored as fat – there is nowhere else it can go.
Look at these balances:
If we use up the same amount of energy that we consume we will remain weight stable
If we consume more than we use up the extra energy has to be stored as fat, so we will gain weight
If we use more than we consume we have to burn fat to make up for the energy deficit, so we lose weight
Does this make sense to you?

Discuss food groups and food plates

- **Use PB2 pages 3-4**

Script:
Please feel free to ask any questions as we go through this.
Different foods belong to different groups depending on the nutrients they provide. We call them food groups. This is important for you to understand when we get to your personal eating plan.
So look here at this plate, it is divided into 5 groups **[PB2 page 4].**
This first section is starchy foods or carbohydrates – it includes thing like rice, bread, pasta etc
The next group is fruits and vegetables and it is quite self-explanatory – the only thing to note really is that we consider potatoes to be part of the starchy group rather than a vegetable because of the amount of starch it contains.
Meat and alternative is the foods that provide us with protein – so meat, fish and eggs go in here. Dahls, lentils and beans are considered to be a meat alternative because they contain a good amount of protein.
Dairy foods is a smaller section – we should be aiming to have 2 portions of dairy foods each day. They are things like milk, yogurt, curd, paneer and other cheeses. Be aware that dairy foods can contain a lot of fat so always try to choose low fat varieties.
The final and smallest section on this plate is fats and sugary foods. These should be eaten only occasionally and should not make up a large part of your diet.
Do you have any questions?
OK, now look at these three plates **[PB2 page 3**]. This is trying to show us how much of each food group should be on our plate. The top plate is showing a 'traditional' plate, ie this is what people normally do. You can see there is quite a large amount of meat and starch and not so much from the vegetables section. The next plate is a 'healthy' plate. This is what everyone should be aiming for. It has more vegetables than the traditional plate and a smaller amount of meat and starch.
The third plate is what we would like you to be aiming for because you are trying to lose weight. Over half of this plate is vegetable foods. Only a quarter is starchy foods and a small amount is meat.

Over the intervention we'll work together to try to move you down the page so that your plate resembles the 'weight-reducing' plate more than the 'traditional' plate which will help you to lose weight and eat a healthy diet.

Explain the 7 healthy eating messages

- **Use PB2 pages 5-6**

Script: Healthy eating messages are in every newspaper and online and they can be very confusing. Usually the things you read or hear will not have any evidence to back them up or they will be exaggerated or misrepresentations. Try not to take too much notice of them.
Healthy eating advice is actually rather simple and it boils down to 7 messages that you can see here on this page.

1.Reduce the amount of fat you eat: You will discuss fat in the first group session in more detail but all you need to know is that fat provides more than twice the calories as protein or carbohydrate – so you only have to eat a small amount to take in a large amount of energy. Use fat sparingly in cooking and be aware of foods that are high in fat. Fats are things like oil, butter, ghee.
2.Fill up with fibre: Fibre makes us feel full so can prevent us from snacking between meals. It's also very good for bowel and heart health. High fibre foods are things like brown bread/rice/pasta and fruits, vegetables and lentils.
3.Be aware of added sugar: Again, you'll discuss this in more detail in the group session. Not only is sugar bad for teeth, it also adds calories to your food. We call added sugar 'empty calories' because it provides nothing useful to the body except extra calories. Some foods are very high in added sugar like fizzy drinks. Limit these sugary foods in your diet.
4.Reduce salt: This does not affect weight loss but it is very important for your heart health. Salt in the diet increases your blood pressure which can lead to heart attacks and stroke. Try to add as little as possible to cooking and food. We get more than enough salt naturally from foods that we don't need to add it.
5.Alcohol contains a lot of calories and should be drank sparingly if you want to lose weight.
6.Learn to read food labels: Food labels tell you how many calories a food provides and how much fat, sugar, salt and fibre are in there. Again you will learn more about this at the group sessions.
7.Control portion sizes. This is of course important for weight loss. Even the healthiest of diets can make you put on weight if you eat enough of them.

Work through My Eating Plan and fill in kcal prescription and portions

Script: Now we get onto your personal eating plan.
If you want to lose 10% body weight in the next few months, at a healthy steady rate of 0.5-1kg per week, we calculate, based on your current weight, height and sex, that you should be consuming around 1500kcal each day.
Don't worry we don't expect you to count calories as that can be very difficult. This plan uses portion lists. So for a 1500kcal plan you can have 7 portions from the starchy group, 6 portions of fruit and veg, 2 portions of meat/alternative, 2 portions of dairy, 3 portions of fat and you have 150kcal spare to eat as you wish.

Look at these portion lists. There is a page per food group and it tells you what 1 portion of each of the foods is.

Let's look at the starch group – pick a food you often eat. OK so you eat rice and bread - it says here that 2 tablespoons of rice is 1 portion and 1 slice of bread is also 1 portion. You are allowed 7 portions in total throughout the day. So for example, if you eat 2 pieces of toast for breakfast and eat 4 tablespoons of rice at dinner you will have had 4 portions, that leaves you with three portions to eat through the rest of the day.
Does this make sense? **[Repeat this process with each food list, asking them to select foods they often eat – or refer to their 7 day diary to pick out foods]**

What we'll do now is divide these portions up into your mealtimes based on what you normally would eat. This page is called Look How well you can eat. So, looking at your food diary and your diet history it looks like you tend to have 2 pieces of toast for breakfast every day, is that right? In that case let's allocate 2 portions of starch to breakfast time. Your evening meal looks like it is the largest meal of your day. So we should allocate at least 3 portions for dinner.
[Continue to complete the Look how well you can eat page. Involve the participant as much as possible, some will be able to lead this section and tell you how they divide up their portions. Others will need a bit more guidance, but you must ensure they understand the concept and are involved in the decision making. If you have a participant who really does not understand or who flatly refuses to engage with this method then you can give them a standard pre-made 1500kcal diet plan to take away with them and encourage them to look at the food lists when they get home to try to see how it relates to what they're eating currently and follow-up on the phone call.]

The idea is that you can start to work with this plan to suit your likes and dislikes. It is completely flexible so you don't have to stick to a rigid eating plan. When you get home have a think about what you eat often and if you come across something that isn't covered on the lists we can talk about it over the phone and give it a place on the lists and a portion size.

You may be able to make all the changes in one go to fit perfectly with this eating plan, however for most people that would be a very difficult thing to do and stick to. The idea is that now you know what you should be eating we'll start to implement changes bit by bit so that your diet starts to look more and more like the eating plan.

What are your thoughts about this way of thinking about your food? Do you have any questions?

Ask the participant to fill in confidence and importance scales

- **Use PB2 page 9**
- **Record in CRF**

Script: OK, let's repeat the importance and confidence scales, they are the same as last week.

Target setting

- **Ask them what target they would like to set. Explain that for visit 1 we would very much like them to set at a physical activity target. However if they would like to set a dietary target as well, that is great.**
- **Encourage them to set an achievable/easy target this week**
- **Help them to shape their target into a SMART target**
- **Fill in PB2 page 10**
- **Record in CRF and PB2 page 10**

Script: Target setting. Would you like to keep last week's targets as they are or would you like to change anything about them?

Now as we've discussed food for most of this session, let's set a dietary target this time. Is there anything you think you could try to change over the next few weeks?

Goodbye

- **Remind them to bring their PB to the next visit**
- **Encourage them to bring family members, particularly the cook of the household**
- **Encourage record keeping**
- **Remind them to continue with their previous targets**
- **Remind them of the importance of attending all visits**
- **Date of next appointment**

5.4.3 Clinic Visit 3 Group session: Focus on fat

Overview

1. Take measurements and review targets
2. Introduce yourself and the content of the session to the group
3. Ice breaker
4. Cover topics:
   - What is fat and why is it important in weight loss
   - Types of fat
   - Hidden fats
   - Tips to reduce fat in our diet
5. Target setting

You will need:

- CRFs for everyone
- Family consent forms
- Participant booklet 3
- Weighing scales, measuring tape
- Flip chart and marker pen
- Fat quiz sheets (and answer sheet for you) Pens
- BHF food cards
- Bottles of fats and oils of the 3 main types of fat displayed on table

Saturated fats	Polyunsaturated fats	Monounsaturated fats
Ghee, butter, coconut oil	Sunflower oil<br>Corn oil<br>Soya	Olive oil<br>Rapeseed oil
Products from these eg. Cheese	Spreads made from these e.g. Flora, Pure.<br>Nuts	Spreads made from these eg. Bertolli,

Appointment length: 1-1.5h

Take measurements and review targets:

- **As people arrive take weight, waist and hip in private**
- **Review target's**
- **Record this in the CRF**
- **Give positive feedback or reassurance**

Script: Hello again, are you happy to take some measurements again?
What targets did you set yourself last week? Did you manage to achieve them in part/fully? Did you have any difficulties? Tell me a bit more about that. *Problem solve with the participant to address any barriers*.
[Follow SOPs for measurements and record measurements and target review in CRF]

Introduce yourself and the content of the session to the group

- **What fats are and why they're important in weight loss**
- **Different types of fat**
- **Discuss the sources of hidden fats**
- **Practical tips to reduce fat intake and making healthier choices**
- **Target setting**
- **Hand out participant books**

Script: Hello everyone, thanks for coming. We have 3 group sessions over the next few weeks so we should all get to know each other well. Today we're focussing of fat. We're going to talk about what fat is and why it's important in weight loss. We'll learn about the different types of fat, hidden fats in foods and then we'll discuss some ideas of the best ways to reduce fat in your diet.
These sessions are very informal and quite fun, so feel free to ask questions whenever you need.

Ice breaker

- **Aim is to get the participant's to talk and feel involved from the beginning**
- **Help's the group to bond and feel relaxed**

Script: Let's begin by introducing ourselves, I would like each of you to tell everybody your first name and also your favourite dish/food. I'll begin, my name is [NAME] and my favourite food is [FOOD]. Next person…

How have you all found the last few weeks since your last appointment? Does anybody want to share what they have found difficult or enjoyed since starting this intervention?

What is fat and why is it important in weight loss?
What are the different types of fat?

- **Use flip chart**
- **Use fats/oils display**
- **Use fat quiz**

Script: Today we are going to focus on fat. Why? Because 1 g of fat has more than twice the calories of other nutrients. So a high fat food will almost always have more calories in it than a high carbohydrate food of similar size or weight. Therefore cutting down on the amount of fat we eat is one of the easiest ways to reduce the amount of calories we eat.
Have a look on the table, which oils/fats do you all use at home?
Does anyone know how many different types of fats there are in our diet? [Wait for the answers, dispel any falsehoods then go through the fats/oils on the display table]

All fats contain a mixture of saturated, monounsaturated and polyunsaturated fatty acids. They are classified according to which type of fatty acids they contain in the greatest amount.
Saturated fats mostly come from animal sources such as fatty meat and dairy products such as butter, ghee, cream and full fat milk. Palm oil, coconut oil, coconut cream and coconut milk are also high in saturated fat. This type of fat raises blood cholesterol, increasing your risk of heart disease.

Unsaturated fats made up of monounsaturates and polyunsaturates are mainly found in vegetable oils such as corn oil, sunflower, olive and rapeseed. These can have a good effect on cholesterol levels if you use them instead of saturated fats.

OK we're going to do a quiz now, do you all have a pen? Have a guess at the answers that you don't know, you can even chat amongst yourselves to come up with answers. Then we'll all go through the answers together.
[Give enough time for all to complete and then go through the answers]

Hidden fats

- **Use BHF food cards**

Script:
[Shuffle the cards and share them between the participants. 5-6 cards each is good. Deal them with the food picture facing upwards and ask them not to turn them over]

I would like you to try to guess which of the foods on these cards contains a lot of fat.
Place all the cards/foods that you think are medium to high fat content in the middle of the table, keep hold of the cards you think are low in fat.
[Give enough time for all to complete this task, then pick up each card and discuss with the group whether it is in the correct place. The underside of the cards lets you know if they are high fat or not]

The cards that have not been put on the table, should now be turned over by the participants so they can confirm that these have no/low fat.

Tips to reduce fat in our diets

- **Use flip-chart**
- **After this, hand out PB3 and direct them to the page on tips to reduce fat**

Script:

What tips can you think of to reduce our fat intake?
[Brainstorming with the group, write their suggestions on the board. If they have missed any obvious ones, write those up too]

From each of you I'd like you to say which of those tips you think would have the biggest impact on your fat intake, and which of those tips you would find most easy to incorporate into your diet?

Here is the participant book for this week. Have a look through it when you get home, it is all based around what we discussed today. On page 6 you'll find a long list of tips to reduce fat intake.

Target setting

- **Record in CRF**
- **Ask them to write their own in their PB3**

Script: So now we're going to set a target to add to the one's you have already started doing.
I'd like to go round the group and ask each of you to suggest the target you would like to make for this week that is based around fat in your diet?
[Help them to formulate them into SMART targets]

Physical Activity reminder

Script: Before you go I would like to remind you of how important it is to keep being physically active. Including physical activity into your routine will make weight loss easier and help keep that weight off.

It is sometimes easier to exercise with other people because it makes it a fun, social occasion and you can encourage each other. As you are all in the same situation and working towards the same goals, one idea is that you arrange amongst yourselves as a group, or in pairs (as a buddy system), to set a date to go for a walk or something else active outside these sessions. Put your hand up if that idea would be of interest to any of you?

OK, is there anyone here who is willing to organise the first activity meet? We can do it now if you wish or we can make time to arrange it the next time we meet when you all know each other a bit better.
All of you that are interested would need to exchange telephone numbers.

Goodbye

- **Remind them to bring their PBs to the next visit**
- **Encourage them to bring family members, particularly the cook of the household**
- **Encourage record keeping**
- **Remind them to continue with their previous targets**
- **Remind them of the importance of attending all visits**
- **Date of next appointment**

5.4.4 Clinic visit 4 Group session: Carbohydrates and triggers

Overview

1. Take measurements and review targets
2. Introduce yourself and the content of the session to the group
3. Ice breaker
4. Cover topics:
   - Types of carbohydrate
   - Hidden sugars
   - Healthier carbohydrate options
   - Carbohydrate food groups
   - Triggers exercise
5. Target setting

You will need:

- CRFs for everyone
- Family consent forms
- Participant booklet 4
- Weighing scales, measuring tape
- Flip chart and marker pen
- Carbohydrate quiz sheets (and answer sheet for you) Pens
- BHF food cards, food models and foot plate mat
- At least 1 glass and 1 tsp and a bag of sugar to use
- Commonly eaten carbohydrate. Use real foods and models
- Discretely label (on the underside) the number of tsps of sugar contained in a few of the sugary drinks/food

Sugary foods	Starchy foods	Fibre rich foods
Sugar, honey bottles of sugary drinks. Mithai, jam, biscuits/chocolate/sweets	Rice, pasta, bread	Brown rice/pasta/bread<br>Fruits and vegetables<br>Dahls, lentils

Appointment length: 1-1.5h

Take measurements and review targets:

- **As people arrive take weight, waist and hip in private**
- **Review target's**
- **Record this in the CRF**
- **Give positive feedback or reassurance**

Script: Hello again, are you happy to take some measurements again?
What targets did you set yourself last week? Did you manage to achieve them in part/fully? Did you have any difficulties? Tell me a bit more about that. *Problem solve with the participant to address any barriers*.
[Follow SOPs for measurements and record measurements and target review in CRF]

Introduce yourself and the content of the session to the group

- **What are the different types of carbohydrate**
- **Hidden sugars**
- **Healthy carbohydrate choices**
- **Carbohydrate food groups**
- **Triggers**
- **Target setting**

Script: Hello everyone, thanks for coming. This is our 2nd group session together.
In the last session we focused on reducing fat intake, so how did it go? What problems did you have? What could you do differently?

Eating less fat is essential to losing weight but its only one important part of healthy eating. Today we're focussing on carbohydrates. We're going to talk about what carbohydrates are and the different types and where we can find them. At the end of the session we're also going to discuss some of the trigger foods and situations that may lead to you eating something you did not want to.

These sessions are very informal and quite fun, so feel free to ask questions whenever you need.

Ice breaker

- **Aim is to get the participant's to talk and feel involved from the beginning**
- **Help's the group to bond and feel relaxed**

Script: Let's introduce ourselves again, I would like each of you to tell everybody your first name and this time also give your favourite sweet/sugary food. I'll begin, my name is [NAME] and my favourite sweet food is [FOOD]. Next person…

What are the different types of carbohydrate?

- **Use carbohydrate display**
- **Use carbohydrate quiz**

Script:
We need carbohydrates in our diet everyday as they provide our main energy supply for the body to keep us and our organs functioning.

Does anyone know what the different types of carbohydrate are?
[Wait for answers and then discuss]
There are 3 types of carbohydrate. Sugars, starch and fibre (indigestible carbohydrate). Have a look at the three groups on the table. [Talk them through the 3 groups]

Sugar and starches are actually very similar once they've been digested however, starches are digested more slowly than sugars and so they are absorbed more slowly into our blood – this is better for us Also starchy foods contain many other nutrients that are good for us.. Added sugar, for example table sugar or in drinks like cola, contribute no other nutrients to the body, they are simply just adding extra calories – we call this 'empty calories'. Half of our energy should come from starchy foods and only a very small part of our diet should come from sugary foods.

The third group is fibre, or indigestible carbohydrates. Fibre is good for bowel health and heart health and makes us feel full when we eat it so it can help to reduce snacking between meals. Also because we can't digest it fully it means we absorb about a quarter of the calories from fibre as we would from starch.
Fibre is found in the cell wall of plant cells so it is in cereals and fruits and vegetables. However when we refine cereals to make white rice and white flour we remove the cell walls and so the fibre too. Brown varieties of rice flour and other cereals still have the cell walls present and so they are fibre-rich – they are a healthier alternative to white varieties. Fibre intake is one of the reasons you should eat plenty of fruits and vegetables.

What type of flour do you use to make your chapattis?
As an alternative to white flour you can also make them from a flour mix of medium brown atta and besan (chick pea flour), which would increase the fibre and protein content of the chapatti.

OK we're going to do a quiz now on sugar, do you all have a pen? Have a guess at the answers that you don't know, you can even chat amongst yourselves to come up with answers. Then we'll all go through the answers together.
[Give enough time for all to complete and then go through the answers]

Carbohydrates and food groups

- **Lay out the food plate mat on the floor**
- **Use BHF food cards**

Script:

**[Shuffle the cards and share them between the participants. 5-6 cards each is good. Deal them with the food picture facing upwards]**<br><br>I would like you to try to guess which of the foods on these cards contain a lot of carbohydrate and then place them in the appropriate section on the food plate. With your remaining cards I'd like you to place them in the other food groups where you think they best fit.<br>**[Give enough time for all to complete this task, then pick up each card and discuss with the group whether it is in the correct place. Start with the starchy food group first, then continue with the sugar/fats section and then the rest of the food groups. Make a point to mention the amount of sugar in some of the sugary foods and compare to the starchy foods]**
**Hidden sugar**<br>• **At least 1 glass, 1 tsp and a bag of sugar**<br>• **Some sugary foods that you have discretely labelled underneath with how much sugar they contain**
**Script:** How many teaspoons of sugar do you think this bottle contains? **[Hold up one of the sugary products and let them reach a consensus as a group]** Could I ask one of you to please spoon into this glass that amount of sugar so we can see what that looks like?<br><br>The actual amount in this bottle is # tsp of sugar. **[If very different from their guess show them the real amount in the glass].** It's interesting to see it like this because most people are happy to drink a whole bottle of this drink but would not be happy to eat that amount of raw sugar in one go. **[Repeat with 2-3 products]**<br><br>Each teaspoon of sugar is about 20kcal so 10 teaspoons is 200kcal. Can you see how a decision about what drink you have at lunch time can add hundreds of calories to your daily intake?
**Tips to make healthier carbohydrate choices**<br>• **Use flip-chart**
**Script:** What tips can you think of to make healthier carbohydrate choices?<br>**[Brainstorming with the group, write their suggestions on the board. If they have missed any obvious ones, write those up too]**<br><br>From each of you I'd like you to say which of those tips you think would have the biggest impact on your own diet and which of those tips you would find most easy to incorporate into your diet?<br><br>Here is the participant book for this week. Have a look through it when you get home, it is all based around what we discussed today.
**Target setting**<br>• **Record in CRF**<br>• **Hand out PB4, ask them to write their targets in their books**
**Script:** So now we're going to set a target to add to the one's you have already started doing.<br>I'd like to go round the group and ask each of you to suggest the target you would like to make for this week that is based around carbohydrates in your diet?<br>**[Help them to formulate them into SMART targets]**
**Triggers**<br>**Use flip chart**
**Script:**<br>We're going to change topic now and move onto triggers.<br>One reason we eat is because of hunger but what about those times when you have a desire to eat without being actually hungry?<br>We call these triggers, they can be emotional or situational.<br>Can you give me an example of one of your own personal triggers?<br>**[Write responses on flip chart, add any obvious missing triggers]**

Now I'd like you to think of some coping strategies for these triggers. A coping strategy would be something you could do to make a healthier choice when that trigger occurs.
[Write responses on flip chart, add any obvious coping strategies that are missing]

Take a look at your booklet, when you get home work through the exercises on triggers and eating behaviour. We can discuss it again on the next telephone call.

Any questions?

Physical Activity reminder

Before you go I would like to remind you of how important it is to keep being physically active. Including physical activity into your routine will make weight loss easier and help keep that weight off.

Did any of you meet up with each other outside this session to do some activity?

Is anyone willing to organise the next activity meet? All of you that are interested would need to exchange telephone numbers.

Goodbye

- **Remind them to bring their PBs to the next visit**
- **Encourage them to bring family members, particularly the cook of the household**
- **Encourage record keeping**
- **Remind them to continue with their previous targets**
- **Remind them of the importance of attending all visits**
- **Date of next appointment**
- **Please try to bring a menu from your favourite take-away or restaurant next visit**

5.4.5 Clinic visit 5 Group session: Food labels and eating out

Overview

1. Take measurements and review targets
2. Introduce yourself and the content of the session to the group
3. Ice breaker
4. Cover topics:
   - Food labels
   - Eating out healthily
   - Dealing with pressures to eat
5. Target setting

You will need:

- CRFs for everyone
- Family consent forms
- Participant booklet 5
- Weighing scales, measuring tape
- Flip chart and marker pen
- Food labels – enough for everyone
-

Appointment length: 1-1.5h

Take measurements and review targets:

- **As people arrive take weight, waist and hip in private**
- **Review target's**
- **Record this in the CRF**
- **Give positive feedback or reassurance**

Script: Hello again, are you happy to take some measurements again?
What targets did you set yourself last week? Did you manage to achieve them in part/fully? Did you have any difficulties? Tell me a bit more about that. *Problem solve with the participant to address any barriers*.
[Follow SOPs for measurements and record measurements and target review in CRF]

Introduce yourself and the content of the session to the group

- **Food labels**
- **Target setting**

Script: Hello everyone, thanks for coming. This is our 3rd group session together.
In the last two sessions we focused on reducing fat intake and on cutting calories from carbohydrates and trigger situations. Today we are going to look at food labels so that we can make healthier choices of what we choose to eat. Then we'll discuss eating out and how to deal with social pressures to eat.
Feel free to ask questions whenever you need.

How did it go in the time since we saw you? What went well? What problems did you have? What could you do differently?

Ice breaker

- **Aim is to get the participant's to talk and feel involved from the beginning**
- **Help's the group to bond and feel relaxed**

Script: Once again, let's introduce ourselves again, I would like each of you to tell everybody your first name and this time also say what fruit you are most like and why.
I'll begin, my name is [NAME] and if I was a fruit I would be a [FRUIT] because I am [FILL IN THE BLANK].
Next person…

Food labels

- **Hand out a food label to each participant**
- **Use flip chart (pre-written)**
- **Hand out PB5**

Script:
Let's discuss Food labels now. How many of you look at food labels before you buy that product?
For those that do read the labels, what do you understand about them?

[Go through pre written text on the flipchart on labels, ask them to look at the labels while you do this]

Nutrition tables: Lists the nutrients found in foods
Can you all see that there are two columns labelled per serving and per 100g?
Per serving: Gives the amount of nutrient consumed is eating a single portion – the label will tell you what a portion is. Can you find the portion size on the packet?
Per 100g: Useful for comparing different products. Again gives the amount of nutrient consumed if you ate 100g
Energy: Written in kcal and kj. Both can be used. 1kcal = 4.2kj
Carbohydrate: Amount of total carbohydrate and the amount of which comes from sugar
Fat: Amount of total fat and the amount of which comes from saturated fat
Fibre: The higher the better. Aim to eat at least 18g per day. Choose foods that say 'high in fibre' or 'a good source of fibre'
Salt: The lower the better. Aim to eat less than 6g per day. Choose products that say "reduced salt" or "no added salt" on the pack.

You probably won't ever look at the ingredients list however, it can sometimes be useful. The ingredients lists all the ingredients in the product in order of quantity, from the most to the least. If there are any types of fat listed in the first 3 ingredients, the food is almost always a high fat food.

Fat can be in the ingredients list in many different names for example, butter, oil, milk solids, coconut, coconut oil, palm oil, hydrogenated vegetable oils, monounsaturated, polyunsaturated and saturated fats etc.

Sugar can be written as raw sugar, brown sugar, molasses, honey, golden syrup, sucrose, lactose, dextrose, and maltose. (Any word ending with –ose is a sugar)

Salt can be written as Salt rock salt, sea salt, celery salt, garlic salt, onion salt, monosodium glutamate etc.

Fat, sugar and fibre will be the things most people will be looking out for on a food label. It may help to remember the 10:10:6 rule. The food is probably a good choice if it meets the following criteria:

Fat < 10g fat per 100g food
Sugar < 10g sugar per 100g food
Fibre more than 6g fibre per 100g food

Overall try to choose the lowest fat, sugar and salt levels possible and choose the highest fibre

[Hand out participant book 5] Here is your chapter for this week. There's more information in there on food labels which you can go over at home. The next time you are in the supermarket I would like you all to try to compare 2 similar types of food. For example a low fat cheese compared to the normal cheese.

Reading labels is a good habit to get into because it gives you the power to make informed choices.
Any questions?

Eating out – healthy choices

- **Use flip chart (prewritten)**

Script: Ok so we're changing topic now. We're going to talk about how to deal with eating out.
Did you all bring a menu along with you? If not I have some here you can use.

Let's go round the group, tell me what you normally order at your restaurant/take-away? And how often do you do so? [Go round the whole group]

So for those of you who only eat out/take-away 1 every month or so, you really don't have too much to worry about. You can enjoy the foods you like as long as you don't do it too often.

If you are eating out or getting a take-away more than once a month you need to start thinking how to deal with those situations so that you do not consume too many calories and ruin your hard work you've done for the rest of the week. You also may have to consider whether you want to limit the number of times you do get a take-away or eat out.

Eating out is meant to be an enjoyable occasion and you shouldn't have to stop or not enjoy yourself while you're there because you don't know what to have or because you feel guilty for what you eat.

There are three things to think about to help you make the best choices. They are written up here on the board:

1) Plan ahead

- Choose the restaurant carefully. Avoid all-you-can-eat restaurants
- Look at the menu before you go and make your decision before you get there and resolve to order what you planned. It is hard to make healthy choices when you are hungry and you have a whole menu to pick from.
- Eat fewer calories during the day so that you can eat at the restaurant and stay within your kcal target
- Plan to have a small snack before you go so that you are not hungry when you are ordering

2) Be assertive and ask for what you want

- Ask the restaurant for food substitutes
- Choose 1 or 2 courses instead of 3
- Ask if someone would like to share a main or dessert with you

3) Choose foods carefully

- Grilled, baked, tandoor will often have fewer calories than other meals
- Watch out for cheese or cream sauces
- Trim off visible fat from meat

Can you suggest some food substitutes that you can ask for at a restaurant or any tips on eating out? [Write the suggestions on the board. Write up some of the obvious examples if they have not been said
- could I have salad/boiled potatoes instead of chips/roast potatoes?
- choose a starter instead of a main dish
- try not to eat the bread that is served before the meal – don't dip it in oil
- ask for sauces and salad dressings to come separately so that you can judge how much you want to add
- please could you serve my potatoes/vegetables without butter or oil?
- could I have my fish grilled instead of fried?
- look for tomato based sauces rather than cream/cheese/coconut milk sauces
- ask for an extra portion of vegetables with your meal to satisfy your appetite
- Be aware of how much alcohol you are drinking – set yourself a limit before you arrive
- Choose sorbet for dessert

Let's go back to your menus. Tell me again what you would normally order and let the rest of the group suggest ways you could adapt to a healthier choice at that restaurant. **[Discuss as whole group]**

Social Pressures to eat
Use PB5 page 7-8

[Split participants into groups of 2 or 3]

Apart from socialising in restaurants there will always be occasions where it may be more difficult for you to stick to your healthy eating. It could be holidays, parties, at a friend's house or even just a hard and tiring day at work. Take a look at pages 7 and 8 in your PB5 on Pressures to Eat.<br><br>I would like you all individually to fill in the table on page 8, but discuss with your group to get other ideas and solutions.<br>**[Give them time to complete this and then ask some people to discuss their responses with the whole group]**
**Target setting**<br>• **Record in CRF**<br>• **Hand out PB5, ask them to write their targets in their books**
**Script:** So now we're going to set a target to add to the one's you are already doing.<br>I'd like to go round the group and ask each of you to suggest the target you would like to make for this week it can be based on something we've discussed today or something completely different?<br>**[Help them to formulate them into SMART targets]**
**Physical Activity reminder**
Before you go I would like to remind you of how important it is to keep being physically active. Including physical activity into your routine will make weight loss easier and help keep that weight off.<br><br>Did any of you meet up with each other outside this session to do some activity?<br>Is anyone willing to organise the next activity meet? All of you that are interested would need to exchange telephone numbers.
**Goodbye**<br>• **Remind them to bring their PBs to the next visit**<br>• **Encourage them to bring family members, particularly the cook of the household**<br>• **Encourage record keeping**<br>• **Remind them to continue with their previous targets**<br>• **Remind them of the importance of attending all visits**<br>• **Date of next appointment**

5.4.6 Clinic Visit 6 Review session

Overview

1. Welcome back
2. Take measurements: Weight, hip, waist. 24h recall.
3. Review last week's targets
4. Introduce PB6 and this week's topics
5. Discuss 6 month review
6. Diet history
7. Discuss future weight loss or weight maintenance
8. Review energy prescription and My Eating Plan
9. Planning for the next phase
10. Confidence and importance scales
11. Target setting

You will need:

- CRF
- Family consent forms
- Participant booklet 6
- Weighing scales, measuring tape
- My Eating Plan and set menu plans
- Weighing scales, measuring tape
- Food portions book and cutlery/crockery
- Food plate mat and BHF food cards

Appointment length: 1-1.5h

Welcome back

Give positive feedback that they have made it this far
How have you found the last few weeks?

Script: Hello again, it's nice to see you. You've made it through the first half of the intervention, you should be very proud of yourself, it shows huge commitment.

How have you found the last few weeks?

Take measurements:

- **Weight, waist and hip**
- **Take a 24h recall**
- **Record this in the CRF**
- **Give positive feedback or reassurance**

Script: Are you happy to take some measurements again?
[Follow SOPs for measurements and 24h recall and record in CRF]

Since the beginning of the intervention you have lost 6kg and your waist has reduced by 3cm – that's amazing. How do you feel about your progress?
Or
You are the same weight as when we started but it doesn't mean that you have had no health benefits. There are plenty of healthy lifestyle changes you have made that will be benefiting your health. How do you feel about your progress?

Review last week's targets

- **Record in CRF and PB6**
- **Give positive feedback for achievements**
- **Non-judgemental**
- **Try to uncover the barriers that prevented them from achieving**

• **Remind them that this is a long process, changing habits is difficult and you will be working with them for the rest of the year**
**Script:** What targets did you set yourself last week? Did you manage to achieve them in part/fully? Did you have any difficulties? Tell me a bit more about that. *Problem solve with the participant to address any barriers*.
**Introduce this week's topic**<br>• **Review past 6 months progress in weight, waist and hip and targets**<br>• **Diet history**<br>• **Future weight loss or weight maintenance?**<br>• **Review energy prescription and My Eating Plan**<br>• **Planning for the next phase**<br>• **Importance and confidence scales**<br>• **Target setting**
**Script:** This appointment is a review of the first half of your lifestyle intervention and planning for the next 6 months. Here is your participant book for this week
**Discuss 6 month review**<br>• **Relate weight , BMI and waist changes back to risk of getting T2D**<br>• **Highlight all the positive changes they have made to their lifestyle**
**Script:** As we said earlier you have lost 6kg in body weight which is an amazing achievement. Losing weight may be one of the hardest things you will ever do.<br>If we take a look at your waist circumference you have lost 3cm from around your waist and your BMI has dropped 2 points. Not only that, when we look back over the physical activity targets you set yourself, you are now far more physically active than before.<br>How do you feel about your progress?<br><br>If you remember the two programme goals were to achieve a 10% weight loss and to increase physical activity to a minimum of 150 minutes per week. This was to reduce your risk of getting T2D.<br><br>You have lost nearly 8% of your body weight at the moment. We know that even a 5-7% weight loss reduces your risk of T2D and you have more than achieved that. You are also doing half an hour of activity on four or five days a week which, even without the weight loss, is benefitting your health.
**Discuss future weight loss or weight maintenance**
**Script:** So you have reduced you risk already so far, however your BMI and waist is still in the at risk category. The next 6 months is very important because it is during this next phase that you begin to cement these lifestyle changes into your life. A very high proportion of people fall at this step and lose commitment to the changes they have made and fall back into old habits and slowly put weight back on.<br><br>During the next 6 months, what would you like to achieve regarding your body weight? Would you like to work towards losing more weight or would you like to work on maintaining your current weight?
**Review energy prescription and My Eating Plan**
**Script:** So you have decided to lose/maintain weight. I've recalculated your requirements based on your latest body weight, age and sex and your aims and you should be consuming 1200kcal per day to lose/maintain weight.<br><br>Here is another page for My Eating Plan. For a 1200kcal plan you can have 5 portions from the starchy group, 5 portions of fruit and veg, 2 portions of meat/alternative, 2 portions of dairy, 3 portions of fat and you have a little over 50kcal spare to eat as you wish.<br><br>You can see the portion lists are the same as before. Would you like me to explain how this plan works to you or are you familiar with it now? Do you have any questions?
**Diet and physical activity history**<br>• **Ask what they would eat on a typical day**<br>• **Which meals do they buy outside of the house**<br>• **Which meals do they prepare**

- Who is the main cook
- Who does the food shopping

- **Record this in the CRF**

Script: I would like to find out a bit more about what your typical diet and lifestyle is like at this point. It will help us to identify further changes that you may want to make or aspects that you are concerned about.

Can you talk to me about what a typical day looks like to you in terms of what you eat, when you eat and where you eat it? Perhaps start with a typical morning….
Do you do most of the cooking? If not who does and do you think we could encourage them to come along to these sessions with you?
Who does the food shopping?
Talk me through the physical activity you do on a daily/weekly basis.

Planning for the next 6 months

Script: As we discussed the next 6 months is very important. What we want to avoid is rebound weight gain which is very common and will put you back at the same risk of T2D as when we began.

The next 6 months of this intervention involves fewer contact points with me and longer gaps in between those contacts. How do you feel about this next phase? Is there anything in particular that concerns you or any topic that you'd like to discuss? What are the main barriers to you achieving your weight loss/maintenance target over the next 6 months?

Confidence and importance scales

- **Use PB6**
- **Record in CRF**

Script: Let's repeat the importance and confidence scales, they are the same as you have done before.
At this moment how important is it that you lose weight in the next 6 months?
At this moment, how confident are your that you will lose weight in the next 6 months?

Target setting

- **Ask them what target they would like to set, make it SMART**
- **Set a review date (when does the participant want to review it? It may be too far away to review it at the next clinic visit)**
- **Do they want to reward themselves if they achieve it? (make this specific)**
- **Record in CRF and PB6**

Script: Today shall we set a target that you will review alone, before our next meeting? We can still discuss it at our telephone call. Do you want to attach a reward to it?

Goodbye

- **Remind them to bring their PBs to the next visit**
- **Encourage them to bring family members, particularly the cook of the household**
- **Encourage record keeping**
- **Remind them to continue with their previous targets**
- **Remind them of the importance of attending all visits**
- **Date of next appointment**

5.4.7 Clinic Visit 7 Group session

Overview

1. **Take measurements and review targets**
2. **Introduce yourself and the content of the session to the group**
3. **Ice breaker**
4. **Cover topics:**
   - **Review achievements**
   - **Sharing advice**
   - **Open topic discussion**
   - **Bring recipes next session!**
5. **Target setting**

You will need:

- **CRFs for everyone**
- **Family consent forms**
- **Participant booklet 7**
- **Weighing scales, measuring tape**
- **Flip chart, marker pen, post-it notes and pens**

Appointment length: 1-1.5h

Take measurements and review targets:

- **As people arrive take weight, waist and hip in private**
- **Review target's**
- **Record this in the CRF**
- **Give positive feedback or reassurance**

Script: Hello again, are you happy to take some measurements again?
What targets did you set yourself last week? Did you manage to achieve them in part/fully? Did you have any difficulties? Tell me a bit more about that. *Problem solve with the participant to address any barriers*.
[Follow SOPs for measurements and record measurements and target review in CRF]

Introduce yourself and the content of the session to the group

- **Review achievements**
- **Share advice**
- **Open topic discussion**

Script: Hello everyone, thanks for coming. This is our 4th group session together.
We're going to review the achievements you've all made and share your best pieces of advice. Then we have some time to talk about anything you like, we could go back over some of the topics we've covered before or just have a question and answer session – it is up to you.
How did it go in the time since we saw you? What went well? What problems did you have? What could you do differently?

Ice breaker

- **Aim is to get the participant's to talk and feel involved from the beginning**
- **Help's the group to bond and feel relaxed**

Script: Once again, let's introduce ourselves again, I would like each of you to tell everybody your first name and this time say what you have most enjoyed about this intervention.
I'll begin, my name is [NAME] and I have enjoyed getting to know you all and seeing you all succeed in making healthy lifestyle changes. Next person…

Achievements

Script: Everyone take a post-it note and a pen. I would like you to spend the next few minutes writing 3 achievements you have made during this process. They could be big or small. Then I'd like you to stick them on the board at the front.

**[Give time for everyone to finish and then go round the group and ask everyone to read them out to the group]**
**Sharing advice**
**Script:** If you all had one tip or piece of advice that you would give to someone who was about to begin to change their lifestyle habits, what would it be?<br><br>Again write it on a new post it note and stick it on the board at the front.<br>**[Give time for everyone to finish and then go round the group and ask everyone to read them out to the group]**
**Open topic discussion**
**Script**: We now have some time to discuss anything you like.<br>Is there anything you would like me to go over again from the topics we've discussed already?<br>Are you having any particular problems or have any concerns you'd like to ask the group about?
**Recipe sharing**
**Script**: For our next group appointment, I would like you all to share some of your healthy recipes you have come up with during your time on the intervention and bring in the dish to share with the group.<br>So if you could, please write the recipe on a piece of paper and bring in the dish.
**Target setting**<br>• **Record in CRF**<br>• **Hand out PB7, ask them to write their targets in their books**
**Script**: So now we're going to set a target to add to the one's you have already started doing.<br>I'd like to go round the group and ask each of you to suggest the target you would like to make for this week it can be based on something we've discussed today or something completely different?<br>**[Help them to formulate them into SMART targets]**
**Physical Activity reminder**
Before you go I would like to remind you of how important it is to keep being physically active. Including physical activity into your routine will make weight loss easier and help keep that weight off.<br>Did any of you meet up with each other outside this session to do some activity?<br>Is anyone willing to organise the next activity meet?
**Goodbye**<br>• **Remind them to bring their PBs to the next visit**<br>• **Encourage them to bring family members, particularly the cook of the household**<br>• **Encourage record keeping**<br>• **Remind them to continue with their previous targets**<br>• **Remind them of the importance of attending all visits**<br>• **Date of next appointment**

5.4.8 Clinic Visit 8 Group session

Overview

1. **Take measurements and review targets**
2. **Introduce yourself and the content of the session to the group**
3. **Ice breaker**
4. **Cover topics:**
   - **Recipe sharing**
   - **Open topic discussion**
   - **THANK YOU and WELL DONE**
5. **Target setting**

You will need:

- **CRFs for everyone**
- **Family consent forms**
- **Participant booklet 8**
- **Weighing scales, measuring tape**
- **Paper and pens and blue-tack**

Appointment length: 1-1.5h

Take measurements and review targets:

- **As people arrive take weight, waist and hip in private**
- **Review target's**
- **Record this in the CRF**
- **Give positive feedback or reassurance**

Script: Hello again, are you happy to take some measurements again?
What targets did you set yourself last week? Did you manage to achieve them in part/fully? Did you have any difficulties? Tell me a bit more about that. *Problem solve with the participant to address any barriers*.
[Follow SOPs for measurements and record measurements and target review in CRF]

Introduce yourself and the content of the session to the group

- **Recipe sharing!**
- **Open topic discussion**

Script: Hello everyone, thanks for coming. This is our 5th and final group session together.
Put all your dishes on the table and stick your recipes up on the board/wall.

Before we start tasting, I would like each of you to tell everybody what dish you have made and how you have made it healthy.

There's some paper and pens on the table so if you want to copy any of the dishes to try at home feel free to take the recipes down.
[Half the session will be eating and talking amongst the group]

Open topic discussion

Script: We now have some time to discuss anything you like.
Is there anything you would like me to go over again from the topics we've discussed already?
Are you having any particular problems or have any concerns you'd like to ask the group about?

Target setting

- **Record in CRF**
- **Hand out PB8, ask them to write their targets in their books**

Script: So now we're going to set a target to add to the one's you have already started doing.
I'd like to go round the group and ask each of you to suggest the target you would like to make for this week it can be based on something we've discussed today or something completely different?
[Help them to formulate them into SMART targets]

Physical Activity reminder

Before you go I would like to remind you of how important it is to keep being physically active. Including physical activity into your routine will make weight loss easier and help keep that weight off.<br>Did any of you meet up with each other outside this session to do some activity?<br>Is anyone willing to organise the next activity meet?
**Goodbye**<br>• **Thank everybody for coming to the sessions and making them enjoyable**<br>• **Remind them to bring their PBs to the next visit**<br>• **Encourage them to bring family members, particularly the cook of the household**<br>• **Encourage record keeping**<br>• **Remind them to continue with their previous targets**<br>• **Remind them of the importance of attending all visits**<br>• **Date of next appointment**

5.4.9 Clinic visit 9 Review

Overview

1. Welcome back
2. Take measurements: Weight, hip, waist. 24h recall.
3. Review last week's targets
4. Introduce PB9 and this week's topics
5. Discuss 6 month review
6. Diet history
7. Discuss future weight loss or weight maintenance
8. Review energy prescription and My Eating Plan
9. Planning for the next phase
10. Confidence and importance scales
11. Target setting

You will need:

- CRF
- Family consent forms
- Participant booklet 9
- Weighing scales, measuring tape
- My Eating Plan and set menu plans
- Weighing scales, measuring tape
- Food portions book and cutlery/crockery
- Food plate mat and BHF food cards

Appointment length: 1-1.5h

Welcome back<br>Give positive feedback that they have made it this far<br>How have you found the last few weeks?
Script: Hello again, it's nice to see you. You've made it through the year-long intervention, you should be very proud of yourself, it shows huge commitment.<br><br>How have you found the last few weeks?
Take measurements:<br>• Weight, waist and hip<br>• Take a 24h recall<br>• Record this in the CRF<br>• Give positive feedback or reassurance
Script: Are you happy to take some measurements again?<br>[Follow SOPs for measurements and 24h recall and record in CRF]<br><br>Since the beginning of the intervention you have lost 9kg and your waist has reduced by 4cm – that's amazing. How do you feel about your progress?<br>Or<br>You are the same weight as when we started but it doesn't mean that you have had no health benefits. There are plenty of healthy lifestyle changes you have made that will be benefiting your health. How do you feel about your progress?
Review last week's targets<br>• Record in CRF and PB6<br>• Give positive feedback for achievements<br>• Non-judgemental<br>• Try to uncover the barriers that prevented them from achieving

- Remind them that this is a long process, changing habits is difficult and you will be working with them for the rest of the year

Script: What targets did you set yourself last week? Did you manage to achieve them in part/fully? Did you have any difficulties? Tell me a bit more about that. *Problem solve with the participant to address any barriers*.

Introduce this week's topic

- **Review past 6 months progress in weight, waist and hip and targets**
- **Diet history**
- **Future weight loss or weight maintenance?**
- **Review energy prescription and My Eating Plan**
- **Planning for the next phase**
- **Importance and confidence scales**
- **Target setting**

Script:
This appointment is a review of the second half of your lifestyle intervention and planning for the next phase.
Here is your participant book for this week

Discuss 6 month review

- **Relate weight , BMI and waist changes back to risk of getting T2D**
- **Highlight all the positive changes they have made to their lifestyle**

Script: As we said earlier you have lost 9kg in body weight which is an amazing achievement. Losing weight may be one of the hardest things you will ever do.
If we take a look at your waist circumference you have lost 4cm from around your waist and your BMI has dropped 3 points. Not only that, when we look back over the physical activity targets you set yourself, you are now far more physically active than before.
How do you feel about your progress?

If you remember the two programme goals were to achieve a 10% weight loss and to increase physical activity to a minimum of 150 minutes per week. This was to reduce your risk of getting T2D.

You have lost over 10% of your body weight now and you are regularly doing half an hour of activity on five days a week which, even without the weight loss, is benefitting your health.

Discuss future weight loss or weight maintenance

Script: So you have reduced you risk already so far, however your BMI and waist is still in the at risk category. The next phase is very important because it is during this next phase that you continue to cement these lifestyle changes into your life. A very high proportion of people fall at this step and lose commitment to the changes they have made and fall back into old habits and slowly put weight back on.

During the next 6 months, what would you like to achieve regarding your body weight? Would you like to work towards losing more weight or would you like to work on maintaining your current weight?

Review energy prescription and My Eating Plan

Script: So you have decided to lose/maintain weight. I've recalculated your requirements based on your latest body weight, age and sex and your aims and you should be consuming 1200kcal per day to lose/maintain weight.

Here is another page for My Eating Plan. For a 1200kcal plan you can have 5 portions from the starchy group, 5 portions of fruit and veg, 2 portions of meat/alternative, 2 portions of dairy, 3 portions of fat and you have a little over 50kcal spare to eat as you wish.

You can see the portion lists are the same as before. Would you like me to explain how this plan works to you or are you familiar with it now? Do you have any questions?

Diet and physical activity history

- **Ask what they would eat on a typical day**

- Which meals do they buy outside of the house
- Which meals do they prepare
- Who is the main cook
- Who does the food shopping

- Record this in the CRF

Script: I would like to find out a bit more about what your typical diet and lifestyle is like at this point. It will help us to identify further changes that you may want to make or aspects that you are concerned about.

Can you talk to me about what a typical day looks like to you in terms of what you eat, when you eat and where you eat it? Perhaps start with a typical morning….
Do you do most of the cooking? If not who does and do you think we could encourage them to come along to these sessions with you?
Who does the food shopping?
Talk me through the physical activity you do on a daily/weekly basis.

Planning for the next 6 months

Script: As we discussed the next 6 months is very important. What we want to avoid is rebound weight gain which is very common and will put you back at the same risk of T2D as when we began.

The next phase involves no more contact points with me. How do you feel about this next phase? Is there anything in particular that concerns you or any topic that you'd like to discuss? What are the main barriers to you achieving your weight loss/maintenance target over the next 6 months?

Confidence and importance scales

- Use PB9
- Record in CRF

Script: Let's repeat the importance and confidence scales, they are the same as you have done before.
At this moment how important is it that you lose weight in the next 6 months?
At this moment, how confident are your that you will lose weight in the next 6 months?

Target setting

- Ask them what target they would like to set, make it SMART
- Set a review date (when does the participant want to review it?)
- Do they want to set a reward if they achieve it? (make this specific)
- Record in CRF and PB9

Script: Today shall we set a target that you will review alone? Do you want to attach a reward to it?

Goodbye

- Encourage them to still seek support from family members
- Encourage record keeping
- Remind them to continue with their previous targets

5.4.10 Telephone call script

Overview

1. **Hello**
2. **Targets : Ask about each target set (exercise and diet)**
   - Congratulate any achievements
   - Problem solve with them to overcome barriers or adapt the target to be more feasible
3. **Meal Plan: Ask how they are finding this. Answer any questions.**
4. **Self monitoring: Have they been recording diet and exercise? Have they been weighing themselves?**
   - Encourage continuation
5. **Any other issues/questions**
6. **Next appointment confirmation**

You will need:

- **CRF**

Appointment length: 15 minutes

Script:

1. Hello, this is [YOUR NAME], you saw me last week in clinic. How are you?

2. How are you doing with your targets?
[Go through each one individually, get specific, measurable answers re SMART. Always ask about both diet and activity targets. Congratulate any achievements. Ask about the barriers that may be preventing them from reaching the targets and problem solve to overcome barriers or adapt the target to be more feasible]

3. How are you finding the meal plan?
[If they are using it, congratulate them, ask if they have any questions. If they are not using it discuss barriers to using it/problem solve/reiterate the benefits of it. Remember it is not essential that they use it, they may be losing weight regardless, however, if they are not losing weight perhaps revisit the meal plan or review their food diary at the next visit]

4. Have you been record keeping? Have you been weighing yourself?
[If yes, congratulate, ask what benefits they are finding from doing so. If not, reiterate benefits of doing so. As above, if they are not losing weight –ask them to do at least 7 day food diary to bring with them to the next appointment so you can review with them]

5. Is there anything you would like to ask or discuss?

6. OK. Look forward to seeing you on [TIME DATE]. Encourage a family member to attend.
Remember to bring:
- Participant book
- Record keeping book [Food diary is essential for week 2 visit]
- Menu [Week 3 only]

5.4.11 Group sessions

Role of the CHW at a group visit

- To time manage so that all the points of that group session are covered
- To engage every individual within the group, especially those who are particularly quiet
- To encourage the group to meet up independently for physical activity
- To steer group discussions back to the topic if they have veered off track
- To ensure targets are set and CRFs are competed

Preparation:

- Read the scripts above to familiarise yourself with the topics of the group sessions and to ensure you have all the supporting material and equipment required for that session.
- Have an attendance list so you know who you are expecting and can have all their CRFs ready.
- Decide if you need extra help. We recommend that you work with someone else to help take body weights and waist measurements and to ensure the CRFs are completed for all participants – especially the targets that are set for each individual at the end of each session.

Consider the choice of venue:

- Is the room big enough?
- Furniture required – enough seats, a low table for display, a partition curtain so you can take measurements in private (if no side room is available), weighing scales and tape measure.
- How accessible is the location for the participants you are targeting?
- How easy is the venue to reach by public transport?
- Are there adequate car parking facilities?
- Noisy rooms, whether due to the air conditioning systems or from external causes like building work, people passing by or traffic can be irritating and distracting.
- A room that is too hot or cold can distract learning. So make sure the ventilation is appropriate.
- Check the level of lighting is suitable.

On the day:

- Arrive at the venue in plenty of time, so that you can sort out any last minute details.
- Make sure you know where the fire exits, toilets and other facilities are located.
- Set up the room. Arrange the chairs in a “horse shoe” shape where participants are positioned in a semi-circle, with a low table in the centre (that can be used to display resources) and trainer sits at the open end. This will create an informal, inclusive atmosphere, where participants will find it easier to interact with each other. If you are using PowerPoint, make sure that the computer and projector are set up and working.

At the beginning of the session:

- Welcome the participants as they arrive and introduce yourself (they should all know you from the clinic visits but they may have brought along other family members)
- As they arrive, take them through to the private area to take their body weight and waist and hip (including consented family members). Record in the CRF and in their participant book.
- Once measurements have been taken for all the group and they are all seated you can begin the group session.
- Introduce yourself and give an overview of the session. Let the group know that the group sessions will be informal, interactive and include group discussion.
- Let the group know where the nearest facilities and the fire exit are.
- Ask them to turn off their mobile phones.
- Ice breaker: Ask everyone to introduce themselves and tell the group their favourite food (or another ice-breaker).
- Start on time and allow late comers to join.

At the end of the session:

- Before you finish – ensure each participant has made a goal for the coming session and they have made a note in their participant book. The CHW must record these targets in the CRFs
- Thank everyone for coming and remind them when the next session is
- Remind them that you will be contacting them before the next group session to check on their progress.
- Ask the participants to fill in a feedback form. Feedback forms are very useful to help the CHW learn what works and what doesn't work so well.

After the session:

- Tidy the room
- Collect all your equipment
- Check all CRF forms have been fully completed
- Gather the completed feedback forms and review them, discuss with other CHWs.

Standard operating procedures

SOP 1: Measuring Height

Ask the participant to remove their shoes (and turban if necessary).
Ask the participant to be positioned as follows:

1. Heels and back to the stadiometer
2. Feet together
3. Ask them to stand straight and ask them to take a breath in to elongate the torso
4. Ask them to look straight ahead and ensure their ear and nose is on the same horizontal plane. This is called the Frankfort plane
5. Record height in the CRF in meters to 2 decimal places. Eg. 1.68m

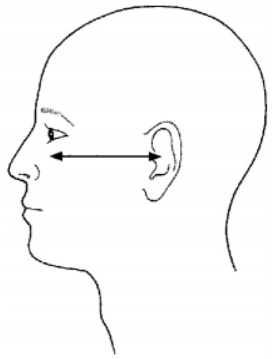

Figure S1: The Frankfort plane

SOP 2: Measuring Body Weight

Ask the participant to remove shoes (and turban if necessary) and outer clothing and to empty their pockets before stepping on the scales.
Record weight in the CRF in kilograms to 2 decimal places. Eg. 60.54kg

Weighing scales:

- Scales should be placed on a hard, level surface and the same set of scales should be used at each subsequent visit.
- If they have a spirit level bubble and adjustable feet, ensure the bubble is in the centre before every measurement
- Calibrate the scales on a monthly basis. Keep a log.

SOP 3: Measuring Waist Circumference

Ask the participant to be positioned as follows:

- Shirt or blouse should be raised.
- Stand up. Feet together
- 'relax and exhale', take measurement at the end of the exhale

Position the tape:

- Find the mid-point between the top of the hip (iliac crest) and the lowest rib
- Ask the participant to find these points if it is difficult to feel due to body fat
- Pass the tape around the waist, either by asking the participant to hold one end and the clinician walking around the participant, or by asking the participant to pass the tape around the waist.
- Make sure the tape remains on a lateral/horizontal plane all the way around the waist
- The tape should fit snugly, in contact with the skin but should not compress the skin
- Take the measurement twice, removing the tape between each measurement. If the measurements are different, measure a third time and take the average of the two closest values.
- Record waist circumference in the CRF in centimetres. Eg. 102cm

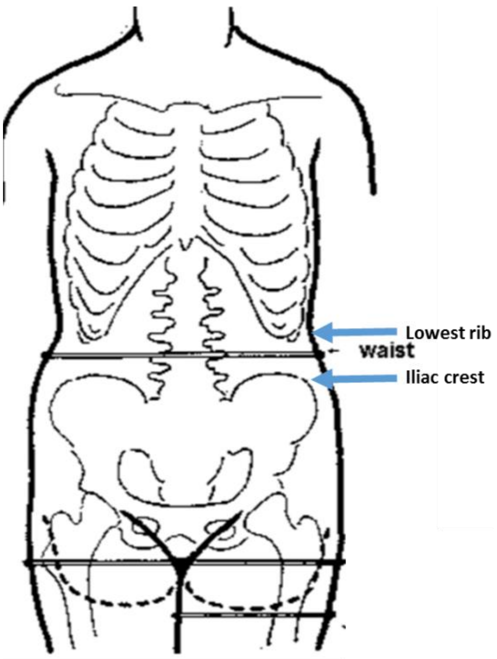

Figure S3: position of the tape measure should be at the mid-point between the lowest rib and the iliac crest

SOP 4: Measuring Hip Circumference

Ask the participant to be positioned as follows:

- Shirt or blouse should be raised. Trousers can remain on.
- Stand up. Feet together
- ‘relax and exhale’, take measurement at the end of the exhale

Position the tape:

- Find the widest point between the thighs and the waist.
- Pass the tape around the hips, either by asking the participant to hold one end and the clinician walking around the participant, or by asking the participant to pass the tape around the hips.
- Make sure the tape remains on a lateral/horizontal plane all the way around the hips – this is particularly important on the hips because of the buttocks. If you place the tape below or above the buttocks you can change the hip measurement drastically.
- The tape should fit snugly, in contact with the skin but should not compress the skin
- Take the measurement twice, removing the tape between each measurement. If the measurements are different, measure a third time and take the average of the two closest values.
- Record the hip circumference in the CRF in centimeters. Eg. 102cm

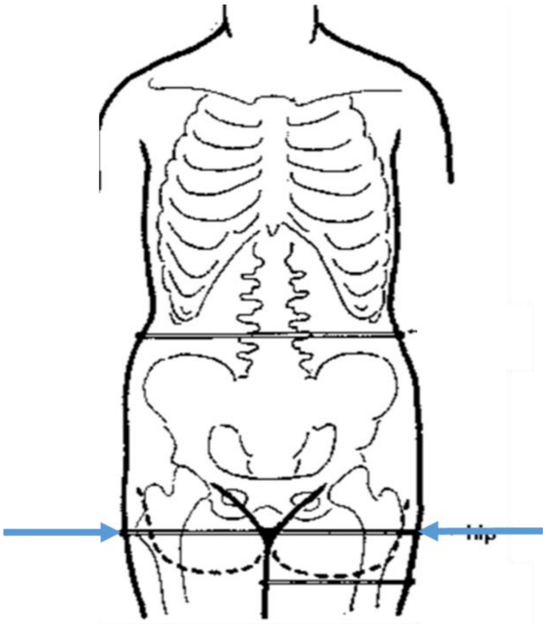

Figure S4: position of the tape measure at the widest point between the thighs and the waist

SOP 5: Multi-pass, 24hour dietary recall

A multi-pass, 24 hour dietary recall is a specific method of recording a participant's food intake over the previous 24 hours. It is conducted at clinic visit 1, 2, 6 and 9. Each recall takes 10-20 minutes.

There are 5 steps which are deliberately repetitious to ensure completeness of the recall. The order, content and the script must be followed exactly as is written:

**Pass 1:** Quick list	"First, we'll make a list of the foods you ate and drank yesterday, (insert DAY). Please tell me everything you had to eat and drink yesterday, (insert DAY) from midnight to midnight. Include everything you had at home and out of the home, including all snacks and drinks no matter how small.<br>I'll ask you for more details and portion sizes in a few minutes. At this time, simply list what you had."<br>Do not interrupt or prompt (unless there is something you don't understand), let the participant fill the silence and do not hurry them. When they have finished ask "Is there anything else that you can remember?"
**Pass 2:** Forgotten foods	"We'll now go through a list of commonly forgotten foods. Let me know if there is anything here that we have not got down on your list already.<br>Did you have any:<br>• **Drinks**: Coffee, tea, soft drinks, milk, alcohol<br>• **Sweet snacks:** Biscuits, cakes, sweets, chocolate, any other sweet snacks<br>• **Savoury snacks:** Crisps, peanuts or other savoury snacks<br>• **Chutney's**, **sauces, dressings, toppings: R**emind them of each meal/snack they had and ask about sauces etc for each one.<br>• **Anything else** that you have not already mentioned?"
**Pass 3:** Time & occasion	"Now we'll go back through each item and I'll ask you to give me the time at which you ate it and about the meal occasion (let the participant name the meal, ie lunch, breakfast, snack etc.)
**Pass 4:** Detail cycle	"Now we'll go back through each item and I'll ask you to give me more detail about the type of food, the amount you ate, how it was cooked and the brand name if bought"<br>Depending on the food, think about asking:<br>• **Type of food**: eg. Semi skimmed milk/full fat milk<br>• **Form purchased**: fresh/frozen/canned/dry<br>• **Amount:** Use unit size, packet size, household measures or food models to get the participant to estimate the amount they ate. Did they eat all of it themselves or leave/share some?<br>• **Method of preparation**: Boiled/ baked/fried/breaded.<br>• **Brand name**<br>• **Home-cooked recipes:** list ingredients and amount of each item, divide total recipe for the amount the participant ate.<br>• **Additions:** was anything added to food during preparation or when eating
**Pass 5:** Final probe	Review the list with the participant.<br>Interviewer to check all necessary detail is there.<br>Ask one final time "Is there anything else that you ate or drank yesterday that we have not got on the list?"

The CHW must record the answers in the CRF legibly and in detail.

General rules when conducting a multi-pass, 24 hour dietary recall

Be prepared: Read the manual and practice conducting the interview until you are comfortable with the content. Ensure that you have all necessary materials to estimate portion sizes, and that your materials are organized in an orderly way.

Explain the procedure carefully and let the respondents know that it may seem repetitious but it is important that it is done in that order. Let them know you will be patient while they try to recall the information. The participant must understand that you are interested in everything they ate or drank during the 24 hours of the previous day, from midnight to midnight.

Encourage participant to talk freely: An important part of your role as an interviewer is to get the respondent actively involved in the interview, to encourage him/her to talk comfortably and freely in response to your questions.

Discourage unrelated conversation: Occasionally a respondent may go off track and talk at length about things with little relevance to the recall. As an interviewer, your task is to keep the discussion focused on the interview. Be polite, acknowledge what they are saying and then ask the question again.

Ask all questions as worded: You must read each question completely and exactly as it is worded to ensure uniformity.

- At times respondents may ask you to define words or to explain some part of a question. Use your judgment to provide clarification without saying something that will bias the response.
- At times, you may feel that he/she has already answered a question before you get to it (Eg. time eaten or brand name). Do not skip over the question, either ask it as usual or confirm the response that you heard.

Always remain neutral. During the entire interview you must always maintain a completely neutral attitude. As an interviewer, you must never allow anything in your words or manner to express criticism, surprise, approval, or disapproval of the questions you ask or of the answers respondents give. You are there to ask for and record the respondent’s answers, not to influence or advise in any way.

Ending the interview Thank participants for providing the information and being patient with the questioning; mention that their detailed answers provide important information to the study. We want them to be willing to repeat the recall 4 times throughout the study so let them know we appreciate their effort and that it is very useful to the study.

Neutral probing

There will be situations where you need more information or clarity than was offered by the participant. This must be done in a neutral way by asking open questions that do not lead the participant to an expected or restricted answer. Eg:

Neutral Probe: "Did you add anything to your coffee?"
Non-neutral Probe: "So you probably added cream and sugar to your coffee?"

Expectant pause: The simplest way to convey to a respondent that you know he/she has begun to answer the question, but has more to say, is to be silent. The pause, often accompanied by eye contact or a nod of the head, allows the respondent time to gather his or her thoughts and continue.
Acknowledge that you are listening: By saying such things as "uh-huh" or "I see" or "yes," the interviewer indicates that the response has been heard, that it is interesting and that more is expected.

Probing to jog their memory: **If respondents seem unable to recall what they ate in the 24-hour period (or for a particular eating occasion), use a prompt. Eg:**
"Take your time to think about it"
"It may help to think about when you woke up yesterday and follow your steps from there"
"Perhaps it will help if you think about what you were doing at that time"

Be careful not to use probes that suggest specific meals or foods such as, "What did you have for breakfast?" or "Do you usually have orange juice first?"

Probing for specificity/more information: Don't ask "Do you mean ____ or _____?" Such a probe suggests only one or two possible answers, when the respondent may actually be thinking about other possibilities. Use neutral probes such as:

"Could you be more specific about that?"
"What type was that?"
"What was the brand name?"
"How was that meal prepared?" (If home recipe, ask for ingredients and cooking methods. If shop bought, ask for brand and cooking methods)

Probing for relevance: If a participant does not answer clearly, do not make assumptions about what they meant. Use a probe such as repeating the original question or asking "What would you like me to record?" Eg:

Question: How much coffee did you drink yesterday?
Answer: Well, not as much as usual.
Probe: OK. Looking at the mugs/cups, can you tell me how many you drank yesterday?

Portion sizes

How much a person ate of a food is the most difficult part for the respondent to estimate and the most difficult part for the data inputter to interpret. The role of the CHW is to gather information from the respondent about portion sizes as accurately as possible and to write it down as clearly as possible for the inputter to read. The more clarity you can provide the better.

You will have the following sets of tools to help the respondent accurately recall the portion size of each food they ate:

1. *Kitchen measures* - These are descriptions such as teaspoon, dessert spoon, tablespoon, cup and mug. (When describing a solid food in spoons, record whether heaped or flat). A 'handful' can be used to describe things like lettuce leaves, dried fruit, nuts, roasted channa and other snacks and berries.
2. *Packet sizes/Food units* – It is appropriate to write portion sizes as packet sizes for many foods, for example a 'regular tin of baked beans' or a 'small individual tin of baked beans' or 'one regular sized packet of crisps' or '1 regular yoghurt pot'. It is useful to find out the brand name of packaged food so the data inputter can look up the exact weight when they come to analyse the recall. A natural food unit is something like '1 medium apple' or '2 biscuits' or '1 slice of cheese' or '1 bacon rasher' etc.
3. *Example cutlery, crockery and glasses* – Each CHW will be provided with a set of various sizes of spoons, bowls and drinking vessels. All labelled with an ID number. You can show these to the participant and write down how many of which vessel is appropriate. Eg. '2 x spoons (ID: SP12) of dhal'
4. *Food atlas* – Each CHW will be provided with a food atlas. This is a picture book of portion sizes of many different foods. It is very useful and accurate to use with the participant. Each picture has an ID number. Eg 1 portion of curry (ID: FA123). It is important you become familiar with this book (and use tabs to label pages you use frequently, such as rice and curry and pasta) so that you can quickly find the correct page, otherwise it can be very time-consuming.

IMPORTANT NOTES:

Do not guess the weight/volume of food. Only write the weight/volume of food down if it is offered by the participant. In all other cases it is better that you write a description of the portion size (as above) and let the data inputter convert that into a weight of food. The data inputters follow a strict protocol and use software to convert different foods into weights which will be more accurate than the CHW can provide.
Record the amount eaten – not the amount served. A participant may be concentrating on describing the serving size to you that they forget that they only ate half of that food, for example.

Don't assume – If a participant says they had a packet of crisps, do not assume it is a regular packet size, they could mean a family/sharing pack. Probe for more information. Another example is asking what they mean by 'a sandwich'. Probe how many slices of bread etc.

Food descriptions

Provide clear information about the type of food so the data inputter knows exactly what was eaten.

Common foods for which you will usually need to gather more information:

1. Milk (and other dairy products) –skimmed, semi-skimmed or full-fat milk. Low-fat or full fat yoghurt. Single or double cream. Sweetened or unsweetened soya/almond milk.
2. Cheese – what type of cheese eg. Paneer/cheddar/brie etc. Low fat or full fat.
3. Meat – with or without skin/fat. Low fat mince or full fat. Wings, thighs or breast etc.
4. Fish – coated or not coated (batter or bread crumbs).
5. Bread – white/brown/wholemeal/granary etc. Medium/thick sliced. Bread roll or sliced etc.
6. Rice/pasta – white, brown, basmati, long-grain etc

Note: For dried foods (lentils, chickpeas, rice, pasta) ensure you describe the portion as dried or cooked because the additional weight of water alters the composition hugely.

Cooking methods

1. Deep-fried, shallow fried, baked, steamed, poached etc
2. How much oil/fat was added
3. Which type of oil/fat was used

Additions and sauces/dressings

Often the additions to foods may be high in sugar and or fat and so contribute significantly to the calorie intake. In one of the stages of the recall you will ask about this. Go back through each meal and food and ask for clarity about the exact composition and additions they may have added.

Common additions in foods can be:

1. Tea/coffee– Indian tea or English tea, the amount of milk/water/sugar used can vary greatly
2. Sandwiches/burgers – If someone said they had a tuna sandwich you will need to probe for additions such as mayonnaise/butter/margarine/salad/cheese/ etc
3. Chappatti – was there fat in the dough or was fat added after
4. Rice – plain boiled or additions such as oil
5. Salad dressing – this is often oil based and will contribute many calories so if someone says they ate salad you should probe further for each ingredient of the salad and whether there was a dressing on it.
6. Tomato ketchup and other sauces are commonly added to many foods such as chips, sandwiches etc
7. Porridge may be made up with milk or water or a mixture

Recipes

For home cooked foods, ask if the participant knows the recipe. Write a list of each of the ingredients and how much was eaten. If the participant cooked the meal for all the family, it may be easier for them to list the total quantity of each ingredient that they added to the recipe and then say, for example, that they ate a quarter of the whole recipe. This section can be quite confusing for the data inputter if you do not make clear what you are describing. This is an example of how to write a recipe:

Food	Time	Portion		Brand
Tea (made up with water)	*08:00*	*Mug*		
-semi skimmed milk		*average*		
- sugar		*1tsp*		
Tuna Sandwich contains:	*13:00*			
-wholemeal bread		*2 slices medium thick*		
-Margarine		*Thinly spread*		
-tuna		*½ a small tin*		
-mayonnaise		*1 tbsp*		
Tea (made up with water)	*15:00*	*Mug*		
-semi skimmed milk		*average*		
- sugar		*1tsp*		
Chocolate digestive biscuit	*15:00*	*2 biscuits*		*McVities*
Chicken curry recipe:	*18:00*	*Whole recipe:*	}	
-Chicken pieces		*400g*	*ate ¼*	
-rapeseed oil		*4 tbsp*	*of*	
-Tinned tomato		*2 tins*	*whole*	
-white onion		*2 large*	*recipe*	
-red pepper		*1 large red pepper*		
-Red chilli		*2 chilli*		

Some participants will not know the recipe at all, in which case you can just write that it was a home cooked chicken curry, perhaps they will be able to describe how much meat they ate and whether there were vegetables in it and how much curry they had (using spoons to describe or using food atlas picture). It is then up to the data inputters and their protocol as to how they enter the composition of a generic chicken curry. **It is not for the CHW to assume ingredients and composition.**

SOP 6: History of weight gain

On visit 1, you will begin to discuss the participant's history of weight gain. A careful and detailed assessment forms the basis of a good weight management programme.

The primary functions of the assessment stage are:

1. To listen to the participant's experience of their weight and to establish rapport
2. To elicit the participant's beliefs and understanding about obesity and to gain an understanding of the factors that have led to weight gain
3. To gather information that will help to characterise the health risks for the participant
4. To gain a picture of current lifestyle habits
5. To identify any potential difficulties or barriers to change
6. To have the opportunity to discuss the expectations from treatment and to agree a way forward.
7. Record appropriate notes in the CRF
8. Ask them to record their reasons for weight gain in participant book 1

SOP 7: Diet and physical activity history

This is done at visit 1. It is less structured than the 24h recall and has a different purpose.

The aim of the diet and physical activity history is for the CHW to gain an understanding of general patterns and eating behaviour of the participant and their physical activity patterns. It is recorded in note form in the CRF.

The diet history will consist of:

- General questions about a 'typical weekday' and a 'typical weekend day' eating
- Whether they get food from canteens/restaurants
- If they cook
- If they shop
- Are they vegetarian
- Also include physical activity questions, including what their work involves
- Make clear notes for yourself in the CRF
- This will help you both to identify when and where changes could be made

SOP 8: Estimating energy requirements

In order to know how many calories a person should be eating we first need to know their total energy expenditure (TEE). We can use the tables on the next page to find a participant's TEE, this is the amount of energy they expend in one day and it has been calculated based on their body weight, height and age and their physical activity level.

If the participant consumes the same amount of calories as their TEE (and does not alter their activity level) they will remain weight stable. Ie They are in energy balance because their intake of calories matches their output of calories.

The tables are based on Schofield equations which allow us to calculate a person's resting energy expenditure (REE; also known as basal metabolic rate or BMR) which is then multiplied by a number which represents their physical activity level (PAL; inactive through to heavy activity level) to give the TEE.

REE x PAL = TEE

REE = Resting Energy Expenditure. Obligatory energy expenditure required for performance of cellular and organ function. Ie it is the energy required to keep your body alive without doing physical activity. Body size and body composition are the main determinants of REE in a healthy adult, and these vary with sex and age.

PAL = Physical Activity Level. This aspect of energy expenditure is variable based on how much activity a person does. The PAL number is simply a factor by which you multiply the REE to give the TEE. You will have to make a judgment about which physical activity level applies best to each participant. You will do this based on the information you gained during the diet and physical activity history you took at visit 1.

Inactive (**PAL = 1.0):** Spends very little time (less than 2 hours) on their feet or involved in any physical activity
Light Activity (PAL = 1.2): Some daily activity (at work or tasks about the house or garden) with at least 2 hours on their feet).
Moderate Activity (PAL = 1.3): Assumes 6 hours on their feet or regular strenuous exercise.
Heavy Activity (PAL = 1.4): Those in heavy labouring jobs or serious athletes in training.

TEE = Total Energy Expenditure. This is the amount of energy a person, doing their normal activities, will expend in a day.

FEMALES	Wt (kg)	Wt (St lb)	Inactive	Light	Moderate	Heavy
18-30 years	60-70	9 6 lb –11 1 lb	1200	1500	1500	2000
	71-75	11 2 lb – 11 12 lb	1500	1800	2000	2300
	76-80	11 13 lb – 12 9 lb	1500	2000	2000	2300
	81-85	12 10 lb – 13 6 lb	1500	2000	2000	2500
	86-90	13 7 lb – 14 3 lb	1500	2000	2300	2500
	91-100	14 4 lb – 15 11 lb	1800	2300	2500	2500
	101-114	15 12 lb – 18 0 lb	2000	2500	2500	2500
	115-125	18 1 lb – 19 10 lb	2300	2500	2500	2500
	>125	> 19 10 lb	2500	2500	2500	2500
31-60 years	60-70	9 6 lb –11 1 lb	1200	1500	1500	1800
	71-75	11 2 lb – 11 12 lb	1200	1500	1800	2000
	76-80	11 13 lb – 12 9 lb	1200	1800	1800	2000
	81-95	12 10 lb – 13 6 lb	1500	1800	2000	2300
	96-114	15 1 lb – 18 0 lb	1500	2000	2300	2500
	115-130	18 1 lb – 20 7 lb	1800	2300	2500	2500
	131-150	20 8 lb – 23 9 lb	2000	2500	2500	2500
	151-170	23 10 lb –26 12 lb	2300	2500	2500	2500
	>170	> 26 12 lb	2500	2500	2500	2500
Over-60 years	60-65	9 6 lb –10 5 lb	1200	1200	1500	1500
	66-75	10 6 lb – 11 12 lb	1200	1500	1500	1800
	76-85	11 3 lb – 13 6 lb	1200	1500	1800	2000
	86-95	13 7 lb – 15 0 lb	1500	1800	2000	2000
	96-100	15 1 lb – 15 11 lb	1500	2000	2000	2000
	101-110	15 12 lb – 17 5 lb	1500	2000	2000	2500
	111-120	17 6 lb – 18 3 lb	1500	2000	2300	2500
	121-135	18 4 lb – 21 4 lb	1800	2300	2500	2500
	136-165	21 5 lb – 26 1 lb	2000	2500	2500	2500
	>165	> 26 1 lb	2500	2500	2500	2500

Table S8.1: Total energy expenditure table for females

MALES	Wt (kg)	Wt (St lb)	Inactive	Light	Moderate	Heavy
18-30 Years	60-65	9 6 lb –10 5 lb	1500	1800	2300	2800
	66-70	10 6 lb – 11 1 lb	1500	2000	2500	3000
	71-75	11 2 lb – 11 12 lb	1500	2000	2500	3000
	76-80	11 13 lb – 12 9 lb	1800	2300	2500	3000
	81-85	12 10 lb – 13 6 lb	1800	2300	2800	3000
	86-95	13 7 lb – 15 0 lb	2000	2500	3000	3000
	96-110	15 1 lb – 17 5 lb	2300	2800	3000	3000
	111-125	17 6 lb – 19 10 lb	2500	3000	3000	3000
	126-135	19 11 lb – 21 4 lb	2800	3000	3000	3000
	>135	> 21 4 lb	3000	3000	3000	3000
31-60 years	60-65	9 6 lb –10 5 lb	1500	1800	2300	2800
	66-70	10 6 lb – 11 1 lb	1500	2000	2300	2800
	71-75	11 2 lb – 11 12 lb	1500	2000	2500	3000
	76-80	11 13 lb – 12 9 lb	1800	2000	2500	3000
	81-85	12 10 lb – 13 6 lb	1800	2300	2500	3000
	86-90	13 7 lb – 14 3 lb	1800	2300	2800	3000
	91-110	14 4 lb – 17 5 lb	2000	2500	2800	3000
	111-120	17 6 lb – 18 13 lb	2300	2800	3000	3000
	121-150	19 0 lb – 23 9 lb	2500	3000	3000	3000
	151-160	23 10 lb – 25 2 lb	2800	3000	3000	3000
	>160	> 25 2 lb	3000	3000	3000	3000
>60 years	60-65	9 6 lb –10 5 lb	1200	1500	1800	2000
	66-70	10 6 lb – 11 1 lb	1200	1500	1800	2300
	71-75	11 2 lb – 11 12 lb	1200	1500	2000	2500
	76-80	11 13 lb – 12 9 lb	1500	1800	2000	2500
	81-85	12 10 lb – 13 6 lb	1500	1800	2300	2800
	86-95	13 7 lb – 15 0 lb	1500	2000	2300	2800
	96-100	15 1 lb – 15 11 lb	1800	2000	2500	3000
	101-110	15 12 lb – 17 5 lb	1800	2300	2800	3000
	111-125	17 6 lb – 19 10 lb	2000	2500	3000	3000
	126-135	19 11 lb – 21 4 lb	2300	2800	3000	3000
	136-165	21 5 lb – 26 1 lb	2500	3000	3000	3000

Table S8.2: Total energy expenditure table for males

If a participant would like to reduce body weight, rather than remain weight stable they will have to consume fewer calories than their TEE. We will recommend they consume 80% of their TEE which results in a steady, healthy rate of weight loss.

To find 80% of the TEE you simply have to multiply the TEE you find in the tables above by 0.8.

Example: 40 year old woman who weighs 85kg with a light activity level, who wishes to begin a 20% energy reduction weight loss plan

Step 1: Find her TEE in the tables

TEE = 1800kcal

Step 2: Multiply the TEE by 0.8

1800 X 0.8 = 1440kcal/day

This calorie prescription of 1440kcal each day should result in a steady, healthy rate of weight loss for the participant.

How much weight will they lose?

Another calculation you will need to do is to calculate how much weight loss this calorie intake should achieve per week. This is important because the participant needs to know whether they are on track.

Step 1: Subtract the new calorie prescription from the original TEE

1800 – 1440 = 360kcal

Step 2: Multiply by 7 (because we want to know the weight loss in a week, not 1 day)

360kcal X 7 = 2520kcal

Step 3: Divide by 7200 (this is just a number we know will convert the calorie deficit into weight (kg) of fat loss)

2520kcal / 7200 = 0.35kg weight loss per week

REMEMBER
As body weight will be changing, energy prescription may have to be recalculated if weight loss begins to plateau.

SOP 9: Food Group Portions Chart

Once you have calculated the energy prescription required to achieve the goal of the participant (ie weight maintenance or weight loss), you will then explain how the eating plan works.

The reason for using food group portions rather than just calories, is two-fold:

1) Many people do not want to count calories or would find it difficult to count calories. It is especially difficult for foods without a nutritional label.
2) By allocating food from each of the foods groups, we are ensuring that it is a healthy diet that will provide all the nutrients they need.

	Number of servings of each food group per day: (kcal per serving given in brackets)						
Energy prescription (kcal/day)	Starch (80kcal)	Fruit (60kcal)	Vegetables (15kcal)	Meat/ Alternatives (140kcal)	Dairy (90kcal)	Fat (36kcal)	Snack Allowance (in kcal)
**1000**	3	2	3	2	2	3	0
**1200**	5	2	3	2	2	3	67
**1500**	7	3	3	2	2	3	147
**1800**	8	4	4	2	2	4	220
**2000**	8	4	4	3	3	5	190
**2300**	11	4	5	3	3	6	199
**2500**	12	5	5	3	3	6	259
**2800**	14	6	5	3	3	7	303
**3000**	16	6	5	3	3	7	343

Table S9: Food portions table

REMEMBER
This is done in visit 2 and at any point at which the calorie prescription has been revised

SOP 10: Taking Informed Consent

Informed Consent is the process by which a subject voluntarily confirms his/her willingness to participate in a study, having been informed of the full details of the project. Informed consent is documented by means of a written, signed and dated informed consent form.

The index case for this study will have been consented by the research nurse before their first visit with the CHW. However, the CHW will be the first point of contact for family members who accompany the index case. Family members may attend all sessions without signing a consent form (with the index case's approval) and without having any measurements or personal details recorded for the purposes of the study. However, if a family member is willing to have their details and weight, waist and hip measurements recorded during the study, informed consent is required before any measurements are taken.

Each CHW will receive appropriate training to be able to take informed consent.

Only family members, aged 18 and over, non-diabetic, residing in the same household as the index case and with the capacity to give informed consent may be recruited to the study as 'family members'.

SOP: INFORMED CONSENT FOR FAMILY MEMBERS:

All potential 'family members' who are interested in taking part in the study should be given a verbal explanation of the study and a participant information sheet for family members. Only when the family member has had adequate time to read the information sheet, has had all questions regarding their participation answered satisfactorily, should they be asked to sign the written informed consent form.

When describing the study the person seeking consent should explain:
I. What the purpose of the study is and any background information that may be relevant.
II. Why the subject has been approached and that confidentiality will be maintained.
III. Details of the study design, duration and number of study visits.
VI. All procedures, such as waist, weight and hip measurements, that are required and any personal details that may be recorded.
VII. The potential benefits and risks of participation in the study.
X. That the subject enters the study voluntarily and can withdraw at any time.

Three copies of the signed consent form should be taken – one given to the family member, one kept in their study file and one in the trial master file.

SOP 11: How to conduct GPAQ

**Physical Activity**		
Next I am going to ask you about the time you spend doing different types of physical activity in a typical week. Please answer these questions even if you do not consider yourself to be a physically active person.<br>Think first about the time you spend doing work. Think of work as the things that you have to do such as paid or unpaid work, study/training, household chores, harvesting food/crops, fishing or hunting for food, seeking employment. [Insert other examples if needed]. In answering the following questions 'vigorous-intensity activities' are activities that require hard physical effort and cause large increases in breathing or heart rate, 'moderate-intensity activities' are activities that require moderate physical effort and cause small increases in breathing or heart rate.<br>Read this opening statement out loud. It should not be omitted. The respondent will have to think first about the time he/she spends doing work (paid or unpaid work, household chores, harvesting food, fishing or hunting for food, seeking employment [Insert other examples if needed]), then about the time he/she travels from place to place, and finally about the time spent in vigorous as well as moderate physical activity during leisure time.<br>Remind the respondent when he/she answers the following questions that 'vigorous-intensity activities' are activities that require hard physical effort and cause large increases in breathing or heart rate. 'Moderate-intensity activities' are activities that require moderate physical effort and cause small increases in breathing or heart rate. Don't forget to use the showcard which will help the respondent when answering to the questions.		
Question	Response	Code
**Work**		
Does your work involve vigorous-intensity activity that causes large increases in breathing or heart rate like [carrying or lifting heavy loads, digging or construction work] for at least 10 minutes continuously?<br>[INSERT EXAMPLES] (USE SHOWCARD)<br>Ask the participant to think about vigorous-intensity activities at work only. Activities are regarded as vigorous intensity if they cause large increases in breathing and/or heart rate.	Yes 1<br>No 2 If No, go to P 4	P1
In a typical week, on how many days do you do vigorous-intensity activities as part of your work?<br>"Typical week" means a week when the participant is engaged in his/her usual activities. Valid responses range from 1-7.	Number of days └─┘	P2
How much time do you spend doing vigorous-intensity activities at work on a typical day?<br>Ask the participant to think of a typical day he/she can recall easily in which he/she engaged in vigorous-intensity activities at work. The participant should only consider those activities undertaken continuously for 10 minutes or more. Probe very high responses (over 4 hrs) to verify.	Hours:minutes<br>└─┴─┘ : └─┴─┘<br>hrs mins	P3 (a-b)

Does your work involve moderate-intensity activity, that causes small increases in breathing or heart rate such as brisk walking [or carrying light loads] for at least 10 minutes continuously?<br>[INSERT EXAMPLES] (USE SHOWCARD)<br>Ask the participant to think about moderate-intensity activities at work only. Activities are regarded as moderate intensity if they cause small increases in breathing and/or heart rate.	Yes 1<br>No 2 If No, go to P 7	P4
In a typical week, on how many days do you do moderate-intensity activities as part of your work?<br>"Typical week" means a week when the participant is engaged in his/her usual activities. Valid responses range from 1-7.	Number of days<br>└┘	P5
How much time do you spend doing moderate-intensity activities at work on a typical day?<br>Ask the participant to think of a typical day he/she can recall easily in which he/she engaged in moderate-intensity activities at work. The participant should only consider those activities undertaken continuously for 10 minutes or more. Probe very high responses (over 4 hrs) to verify.	Hours:minutes<br>└┴┘ : └┴┘<br>hrs mins	P6 (a-b)
**Travel to and from places**		
The next questions exclude the physical activities at work that you have already mentioned.<br>Now I would like to ask you about the usual way you travel to and from places. For example, to work, for shopping, to market, to place of worship. [Insert other examples if needed]<br>The introductory statement to the following questions on transport-related physical activity is very important. It asks and helps the participant to now think about how they travel around getting from place-to-place. This statement should not be omitted.		
Do you walk or use a bicycle (pedal cycle) for at least 10 minutes continuously to get to and from places?<br>Select the appropriate response.	Yes 1<br>No 2 If No, go to P 10	P7
How much time do you spend walking or bicycling for travel on a typical day?<br>Ask the participant to think of a typical day he/she can recall easily in which he/she engaged in transport-related activities. The participant should only consider those activities undertaken continuously for 10 minutes or more. Probe very high responses (over 4 hrs) to verify.	Hours:minutes<br>└┴┘ : └┴┘<br>hrs mins	P9(a-b)
**Recreational activities**		
The next questions exclude the work and transport activities that you have already mentioned.<br>Now I would like to ask you about sports, fitness and recreational activities (leisure) [Insert relevant terms].<br>This introductory statement directs the participant to think about recreational activities. This can also be called discretionary or leisure time. It includes sports and exercise but is not limited to participation in competitions. Activities reported should be done regularly and not just occasionally. It is important to focus on only recreational activities and not to include any activities already mentioned. This statement should not be omitted.		

Do you do any vigorous-intensity sports, fitness or recreational (leisure) activities that cause large increases in breathing or heart rate like [running or football] for at least 10 minutes continuously?<br>[INSERT EXAMPLES] (USE SHOWCARD)<br>Ask the participant to think about recreational vigorous-intensity activities only. Activities are regarded as vigorous intensity if they cause large increases in breathing and/or heart rate.	Yes 1<br><br>No 2 If No, go to P 13	P10
In a typical week, on how many days do you do vigorous-intensity sports, fitness or recreational (leisure) activities?<br>"Typical week" means a week when the participant is engaged in his/her usual activities. Valid responses range from 1-7.	Number of days └─┘	P11
How much time do you spend doing vigorous-intensity sports, fitness or recreational activities on a typical day?<br>Ask the participant to think of a typical day he/she can recall easily in which he/she engaged in recreational vigorous-intensity activities. The participant should only consider those activities undertaken continuously for 10 minutes or more. Probe very high responses (over 4 hrs) to verify.	Hours:minutes<br>└─┴─┘ : └─┴─┘<br>hrs mins	P12 (a-b)
Do you do any moderate-intensity sports, fitness or recreational (leisure) activities that cause a small increase in breathing or heart rate such as brisk walking, [cycling, swimming, volleyball] for at least 10 minutes continuously?<br>[INSERT EXAMPLES] (USE SHOWCARD)<br>Ask the participant to think about recreational moderate-intensity activities only. Activities are regarded as moderate intensity if they cause small increases in breathing and/or heart rate.	Yes 1<br><br>No 2 If No, go to P16	P13
In a typical week, on how many days do you do moderate-intensity sports, fitness or recreational (leisure) activities?<br>"Typical week" means a week when the participant is engaged in his/her usual activities. Valid responses range from 1-7.	Number of days └─┘	P14
How much time do you spend doing moderate-intensity sports, fitness or recreational (leisure) activities on a typical day?<br>Ask the participant to think of a typical day he/she can recall easily in which he/she engaged in recreational moderate-intensity activities. The participant should only consider those activities undertaken continuously for 10 minutes or more. Probe very high responses (over 4 hrs) to verify.	Hours:minutes<br>└─┴─┘ : └─┴─┘<br>hrs mins	P15 (a-b)
**Sedentary behaviour**		
The following question is about sitting or reclining at work, at home, getting to and from places, or with friends including time spent sitting at a desk, sitting with friends, traveling in car, bus, train, reading, playing cards or watching television, but do not include time spent sleeping. [INSERT EXAMPLES] (USE SHOWCARD)		
How much time do you usually spend sitting or reclining on a typical day?<br>Ask the participant to consider total time spent sitting at work, in an office, reading, watching television, using a computer, doing hand craft like knitting, resting etc. The participant should not include time spent sleeping.	Hours:minutes<br>└─┴─┘ : └─┴─┘<br>hrs mins	P16 (a-b)

Appendix

Appendix 1: Inclusion and Exclusion Criteria for Family Members

Inclusion criteria:

- South Asian, Male or Female , aged >18 years
- Living in the same household as an index case receiving intensive lifestyle modification

Exclusion criteria:

- Known type 1 or 2 diabetes
- Serious illness
- Lack of capacity to consent

Appendix 2: Participant Information Sheet

Add your Institution's Participant Information Sheet. Make sure to use the correct dated and document version.

Appendix 3: Participant Consent Form

Add your Institution's Participant Consent Form. Make sure to use the correct dated and document version.

Appendix 4: Information Sheet for Family Members

Add your Institution's Information Sheet for Family members.
Make sure to use the correct dated and document version.

Appendix 5: Consent form for Family Members

Add your Institution's Consent Form for Family members. Make sure to use the correct dated and document version.

Appendix 6: Frequently asked questions from participants about nutrition

1. I thought carbohydrates were fattening?
We get energy from carbohydrates, protein fat and alcohol in our diet. Any extra (unneeded) energy we take in will be converted to fat no matter what source. Sometimes people think that carbohydrates are fattening, however, the same amount (in weight) of carbohydrate contains less than half the calories of fat. Studies have also shown that carbohydrates are better at satisfying our hunger. The trick is not to add lots of fat when you are cooking or preparing starchy foods like potatoes, rice and chapattis.

2. What is the difference between brown bread and wholegrain bread?
Note: Wholemeal, wholegrain and wholewheat are essentially the same thing.
Brown bread is better than white bread. Wholegrain is better than both brown and white bread.
Whole grain means that all of the grain is used in the flour, this means it contains much more nutrients and fibre than white flour, which has had most of the nutrients and all the fibre removed. Brown bread is often made from a mixture of white flour and wholegrain flour with added ingredients for colour. Brown bread contains fewer nutrients and fibre than wholegrain bread but more than white bread.

3. Should I cut out all the sugar in my diet to prevent the onset of T2D?
Eating sugar does not cause diabetes. Maintaining a healthy weight and eating a healthy balanced diet, low in fat and rich in fibre and fruit and vegetables, as well as being physically active is the best way of reducing your risk. Even if you're not overweight, maintain a healthy weight through eating well and being active is an important part of managing blood sugar levels and avoiding other health complications.

4. I have high cholesterol. Should I avoid eating eggs?
No. Keeping saturated fat low has been shown to be better at reducing cholesterol than restricting eggs. Eggs can be included in a healthy balanced diet, but remember that it's a good idea to eat as varied a diet as possible and use healthier cooking methods when you do have eggs. Boil or poach them rather than frying.

5. Are reduced fat options always the best on my weight loss plan?
Don't assume that "low fat" or "fat-free" versions of products are healthy or better choices - many of these foods are very high in sugar. Always check the label.

6. Which is the best oil to use for cooking?
Any oil that you use should contain high amounts of monounsaturated and polyunsaturated fats with minimal saturated fats. Different fats and oils have different uses. Each performs best within a certain range of temperatures. Heating oil can change its characteristics. Some oils that are healthy at room temperature can become unhealthy when heated above certain temperatures. When choosing cooking oil it is important to consider its smoking point. This is the temperature at which the fat begins

to smoke and smell. Processing, re-use, age and improper storage can all lower the smoking point. When oil smokes, it loses some of its health-promoting properties, so for the healthiest approach discard any oil that has gone beyond its smoke point.
Oils suitable for medium temperature frying, e.g. curry sauces, stir-frying, pan –fry

- Rapeseed oil
- Olive oil
- Sunflower oil
- Sesame seed oil
- Corn oil

Unrefined oils, such as virgin olive oil should be restricted to using in salad dressings.

7. Why is coconut oil so popular?
A very clever marketing programme has seen coconut oil become a popular choice in health food stores and supermarkets. There are lots of stories about its health benefits but the evidence is sparse and scientists are far from convinced. Coconut oil is highly saturated-the highest of any oil. The fats in coconut oil can significantly raise bad LDL cholesterol, so it's best to avoid coconut oil. If you do use it in cooking, use occasionally only and try to use the lighter versions.

8. Where do omega 3 fats come from?
Fish, and especially oily fish, are good sources omega 3 (Also called DHA and EPA). Oily fish has the highest levels. These include mackerel, pilchards, salmon, sardines, trout, and tuna (fresh and frozen). Canned fish does count but some brands of tuna may have the omega 3 removed during processing, so always try to check the label. White fish such as cod, haddock and plaice contains some omega-3 too but at much lower levels than oily fish.

9. I am a vegetarian, where can I get omega-3s from?
People that don't eat fish can get omega-3 from the following foods: nuts and seeds e.g. walnuts and pumpkin seeds; vegetable oils e.g. rapeseed and linseed; soya and soya products e.g. beans, milk and tofu; and green leafy vegetables.

10. What about omega 3 supplements instead of omega rich foods?
It is best to try to get omega-3 from foods but the following advice may be helpful if you find this difficult and wish to take a supplement:

- Look for omega 3 oil rather than fish liver oil
- Check the vitamin A content- The Scientific Advisory Committee on Nutrition (SACN) advises that if you take supplements containing vitamin A, you should not have more than a total of 1.5mg (1500ug) a day from food and supplements combined.
- Check labels for DHA and EPA content-stick to the daily amount provided by eating one to two portions of fish per week (about 450mg EPA and DHA per daily adult dose)
- Do not take supplements containing vitamin A if you are pregnant or planning a baby
