# Supplementary material for: The iHealth-T2D study, prevention of type 2 diabetes amongst South Asians with central obesity and prediabetes: study protocol for a randomised controlled trial
Source: Trials. 2021 Dec 18;22:928. doi: 10.1186/s13063-021-05803-7 (PMC8684177; doi:10.1186/s13063-021-05803-7)
Supplement: Supplementary file 1 — Additional file 1: S1. CHW Handbook. Training manual summarising the content and delivery of the lifestyle intervention. Translated versions available through study website. S2. CHW Workbook. Case report form / intervention delivery workbook for the lifestyle intervention. Translated versions available through study website. S3. Participant Handbook for Lifestyle intervention. Written materials provided to the participant to support the CHW delivered lifestyle intervention. Translated versions available through study website. S4. Participant Handbook for Usual care. Translated versions available through study website. Supplementary Table 1: The a-priori power calculations under alternate assumptions with a sample size of 3,600 participants. [file 13063_2021_5803_MOESM1_ESM.zip › Addcorr2.zip!/S2 Intensive care CRF.pdf]

Lifestyle Intervention Clinic Report Form

Participant name:		Initials:			
Participant ID:		DOB:	DD/MM/YYYY	Sex:	M/F
Does the participant cook?	Y/N	Is the participant the main cook of their household?			Y/N

Planned appointments

Episode	Visit	Week	Planned visit date	Actual visit date	Attended	CHW ID
1	Clinic visit 1 (121)	1	DD/MM/YY	DD/MM/YY	Y/N	
2	Phonecall	2	DD/MM/YY	DD/MM/YY	Y/N	
3	Clinic visit 2 (121)	3	DD/MM/YY	DD/MM/YY	Y/N	
4	Phonecall	5	DD/MM/YY	DD/MM/YY	Y/N	
5	Clinic visit 3 (Group)	7	DD/MM/YY	DD/MM/YY	Y/N	
6	Phonecall	9	DD/MM/YY	DD/MM/YY	Y/N	
7	Clinic visit 4 (Group)	11	DD/MM/YY	DD/MM/YY	Y/N	
8	Phonecall	13	DD/MM/YY	DD/MM/YY	Y/N	
9	Clinic visit 5 (Group)	15	DD/MM/YY	DD/MM/YY	Y/N	
10	Phonecall	17	DD/MM/YY	DD/MM/YY	Y/N	
11	Clinic visit 6 (121)	19	DD/MM/YY	DD/MM/YY	Y/N	
12	Phonecall	21	DD/MM/YY	DD/MM/YY	Y/N	
13	Phonecall	23	DD/MM/YY	DD/MM/YY	Y/N	
14	Clinic visit 7 (Group)	25	DD/MM/YY	DD/MM/YY	Y/N	
15	Phonecall	28	DD/MM/YY	DD/MM/YY	Y/N	
16	Phonecall	31	DD/MM/YY	DD/MM/YY	Y/N	
17	Phonecall	34	DD/MM/YY	DD/MM/YY	Y/N	
18	Clinic visit 8 (Group)	37	DD/MM/YY	DD/MM/YY	Y/N	
19	Phonecall	40	DD/MM/YY	DD/MM/YY	Y/N	
20	Phonecall	43	DD/MM/YY	DD/MM/YY	Y/N	
21	Phonecall	46	DD/MM/YY	DD/MM/YY	Y/N	
22	Clinic visit 9 (121)	49	DD/MM/YY	DD/MM/YY	Y/N	

Clinic visit notes: pages 4-15
Phone call notes: pages 16-18

**Participant ID**	
**Participant initials**	

iHealth-T2D

Participant progress chart: Clinic visits 1-5

	**Height (m):**	**BMI (kg/m²)**	**Weight (kg)**	**Waist (cm)**	**Exercise (mins/week)**
**Start**					
**Target**					>150

**Clinic visit**	**Visit 1**	**Visit 2**	**Visit 3**	**Visit 4**	**Visit 5**
Visit type	**121**	**121**	**Group**	**Group**	**Group**
Date	DD/MM/YY	DD/MM/YY	DD/MM/YY	DD/MM/YY	DD/MM/YY
CHW ID					

**Participant**					
Weight (kg)					
Waist (cm)					
Hip (cm)					
Importance scale (1-10)					
Confidence scale (1-10)					
**Lifestyle data collection**					
24 hour recall completed	Y/N (p20-21)	Y/N (p22-23)			
7 day food diary collected		Y/N			
**Treatment**					
Targets set / reviewed	Y/N (p4)	Y/N (p6)	Y/N (p7)	Y/N (p8)	Y/N (p9)
Lose / maintain weight	Lose / Maintain	Lose / Maintain	Lose / Maintain	Lose / Maintain	Lose / Maintain
Calorie prescription (kcal)					
Menu plan	Standard / Personal	Standard / Personal	Standard / Personal	Standard / Personal	Standard / Personal
**Materials given out**					
Participant booklet	Y/N	Y/N	Y/N	Y/N	Y/N
Tape measure	Y/N				
7 day food diary	Y/N				

**Relatives**					
Family members attended	Y/N	Y/N	Y/N	Y/N	Y/N
Family data recorded	Y/N (p30)	Y/N (p30)	Y/N (p30)	Y/N (p30)	Y/N (p30)

Participant ID	
Participant initials	

iHealth-T2D

Participant progress chart: Clinic visits 6-9

Clinic visit	Visit 6	Visit 7	Visit 8	Visit 9
Visit type	121	Group	121	121
Date	DD/MM/YY	DD/MM/YY	DD/MM/YY	DD/MM/YY
CHW ID				

Participant				
Weight (kg)				
Waist (cm)				
Hip (cm)				
Importance scale (1-10)				
Confidence scale (1-10)				
**Lifestyle data collection**				
24 hour recall completed	Y/N (p24-25)			Y/N (p26-27)
GPAQ completed	Y/N (p28-29)			
**Treatment**				
Targets set / reviewed	Y/N (p10)	Y/N (p12)	Y/N (p13)	Y/N (p14)
Lose / maintain weight	Lose / Maintain	Lose / Maintain	Lose / Maintain	Lose / Maintain
Calorie prescription (kcal)				
Menu plan	Standard / Personal	Standard / Personal	Standard / Personal	Standard / Personal
**Materials given out**				
Participant booklet	Y/N	Y/N	Y/N	Y/N

Relatives				
Family members attended	Y/N	Y/N	Y/N	Y/N
Family data recorded	Y/N (p30)	Y/N (p30)	Y/N (p30)	Y/N (p30)

**Participant ID**	
**Participant initials**	

iHealth-T2D

Targets and Notes: Clinic Visit 1

**Administrative information**			
Appointment Date		Appointment time	
Data sheet completed (p2)	Y/N	CHW name	

**Diet history**
Typical day: Diet, exercise, work, cooking, shopping:

Participant ID	
Participant initials	

iHealth-T2D

Target setting	
Set Targets (Set at clinic visit 1)	Targets met? (Review at clinic visit 2)

Notes

Participant ID	
Participant initials	

iHealth-T2D

Targets and Notes: Clinic Visit 2

Administrative information			
Appointment Date		Appointment time	
Data sheet completed (p2)	Y/N	CHW name	

Target setting	
Set Targets (Set at clinic visit 2)	Targets met? (Review at clinic visit 3)

Notes

Participant ID	
Participant initials	

iHealth-T2D

Targets and Notes: Clinic Visit 3

**Administrative information**			
Appointment Date		Appointment time	
Data sheet completed (p2)	Y/N	CHW name	

**Target setting**	
Set Targets (Set at clinic visit 3)	Targets met? (Review at clinic visit 4)

**Notes**

**Participant ID**	
**Participant initials**	

iHealth-T2D

Targets and Notes: Clinic Visit 4

**Administrative information**			
Appointment Date		Appointment time	
Data sheet completed (p2)	Y/N	CHW name	

**Target setting**	
Set Targets (Set at clinic visit 4)	Targets met? (Review at clinic visit 5)

**Notes**

Participant ID	
Participant initials	

Targets and Notes: Clinic Visit 5

Administrative information			
Appointment Date		Appointment time	
Data sheet completed (p2)	Y/N	CHW name	

Target setting	
Set Targets (Set at clinic visit 5)	Targets met? (Review at clinic visit 6)

Notes

**Participant ID**	
**Participant initials**	

Targets and Notes: Clinic Visit 6

**Administrative information**			
Appointment Date		Appointment time	
Data sheet completed (p3)	Y/N	CHW name	

**Diet history**
Typical day: Diet, exercise, work, cooking, shopping:

Participant ID	
Participant initials	

iHealth-T2D

Target setting	
Set Targets (Set at clinic visit 6)	Targets met? (Review at clinic visit 7)

Notes

Participant ID	
Participant initials	

iHealth-T2D

Targets and Notes: Clinic Visit 7			
**Administrative information**			
Appointment Date		Appointment time	
Data sheet completed (p3)	Y/N	CHW name	
**Target setting**			
Set Targets (Set at clinic visit 7)		Targets met? (Review at clinic visit 8)	
**Notes**			

Participant ID	
Participant initials	

iHealth-T2D

Targets and Notes: Clinic Visit 8

Administrative information			
Appointment Date		Appointment time	
Data sheet completed (p3)	Y/N	CHW name	

Target setting	
Set Targets (Set at clinic visit 8)	Targets met? (Review at clinic visit 9)

Notes

**Participant ID**	
**Participant initials**	

iHealth-T2D

Targets and Notes: Clinic Visit 9

**Administrative information**			
Appointment Date		Appointment time	
Data sheet completed (p3)	Y/N	CHW name	
**Diet history**			
Typical day: Diet, exercise, work, cooking, shopping:			

Target setting
Set long term targets (Set at clinic visit 9)
**Notes**

Participant ID	
Participant initials	

iHealth-T2D

Phone Call Notes:

Date	Episode	Notes

Participant ID	
Participant initials	

iHealth-T2D

Phone Call Notes:

Date	Episode	Notes

Phone Call Notes:

Date	Episode	Notes

Participant ID	
Participant initials	

24 Hour Dietary Recall - Quick Script Instructions

1. Quick list

"First, we'll make a list of the foods you ate and drank yesterday, (insert DAY). Please tell me everything you had to eat and drink yesterday, (insert DAY) from midnight to midnight. Include everything you had at home and out of the home, including all snacks and drinks no matter how small.
I'll ask you for more details and portion sizes in a few minutes. At this time, simply list what you had."
Do not interrupt or prompt (unless there is something you don't understand), let the participant fill the silence and do not hurry them. When they have finished ask "Is there anything else that you can remember?"

2. Forgotten Foods

"We'll now go through a list of commonly forgotten foods. Let me know if there is anything here that we have not got down on your list already.
Did you have any:

- **Drinks**: Coffee, tea, soft drinks, milk, alcohol
- **Sweet snacks:** Biscuits, cakes, sweets, chocolate, any other sweet snacks
- **Savoury snacks:** Crisps, peanuts or other savoury snacks
- **Sauces, dressings, toppings:** Remind them of each meal/snack they had and ask about sauces etc for each one.
- **Anything else** that you have not already mentioned?"

3. Time and Occasion

"Now we'll go back through each item and I'll ask you to give me the time at which you ate it and about the meal occasion (let the participant name the meal, ie lunch, breakfast, snack etc.)

4. Detail cycle

"Now we'll go back through each item and I'll ask you to give me more detail about the type of food, the amount you ate, how it was cooked or the brand name if bought"
Depending on the food, think about asking:

- **Type of food**: eg. type of milk –Semi skimmed/full fat
- **Form purchased**: fresh/frozen/canned/dry
- **Amount:** Use unit size, packet size, household measures or food models to get the participant to estimate the amount they ate. Did they eat all of it themselves or leave/share some?
- **Method of preparation**: Boiled/ baked/fried/breaded.
- **Brand name**
- **Home-cooked recipes:** what are the ingredients and amount of each item, divide total recipe for the amount the participant ate.
- **Additions:** was anything added to food during preparation or when eating like ketchup, cream, sugar

5. Final probe

Review the list with the participant.
Interviewer to check all necessary detail is there.
Ask one final time "Is there anything else that you ate or drank yesterday that we have not got on the list?"

**Participant ID**	
**Participant initials**	

iHealth-T2D

24 hour dietary recall: Clinic Visit 1

**Administrative information**			
Appointment Date		Appointment time	
Data sheet completed (p3)	Y/N	CHW name	
**24 hour food recall information**			

Food /drink description	***Time***	***Meal***	***Portion size***	***Brand, home-made, take-away, restaurant***

Participant ID	
Participant initials	

24 hour dietary recall: Clinic Visit 1 (continued)

Food /drink description	*Time*	*Meal*	*Portion size*	*Brand, home-made, take-away, restaurant*

Participant ID	
Participant initials	

iHealth-T2D

24 hour dietary recall: Clinic Visit 2

**Administrative information**			
Appointment Date		Appointment time	
Data sheet completed (p3)	Y/N	CHW name	

24 hour food recall information

Food /drink description	***Time***	***Meal***	***Portion size***	***Brand, home-made, take-away, restaurant***

**Participant ID**	
**Participant initials**	

24 hour dietary recall: Clinic Visit 2 (continued)

Food /drink description	*Time*	*Meal*	*Portion size*	*Brand, home-made, take-away, restaurant*

Participant ID	
Participant initials	

iHealth-T2D

24 hour dietary recall: Clinic Visit 6

Administrative information			
Appointment Date		Appointment time	
Data sheet completed (p3)	Y/N	CHW name	

24 hour food recall information

Food /drink description	*Time*	*Meal*	*Portion size*	*Brand, home-made, take-away, restaurant*

Participant ID	
Participant initials	

24 hour dietary recall: Clinic Visit 6 (continued)

Food /drink description	*Time*	*Meal*	*Portion size*	*Brand, home-made, take-away, restaurant*

Participant ID	
Participant initials	

24 hour dietary recall: Clinic Visit 9

Administrative information			
Appointment Date		Appointment time	
Data sheet completed (p3)	Y/N	CHW name	

24 hour food recall information

Food /drink description	*Time*	*Meal*	*Portion size*	*Brand, home-made, take-away, restaurant*

Participant ID	
Participant initials	

iHealth-T2D

24 hour dietary recall: Clinic Visit 9 (continued)

Food /drink description	*Time*	*Meal*	*Portion size*	*Brand, home-made, take-away, restaurant*

Participant ID	
Participant initials	

iHealth-T2D

Physical Activity Record (GPAQ): Clinic Visit 6

Administrative information			
Appointment Date		Appointment time	
Data sheet completed (p3)	Y/N	CHW name	

Physical Activity Questions

I am going to ask you about the time you spend doing different types of physical activity in a typical week. Please answer these questions even if you do not consider yourself to be a physically active person.

Think first about the time you spend doing work. Think of work as the things that you have to do such as paid or unpaid work, study/training, household chores, harvesting food/crops, fishing or hunting for food, seeking employment. In answering the questions 'vigorous-intensity activities' are activities that require hard physical effort and cause large increases in breathing or heart rate, 'moderate-intensity activities' are activities that require moderate physical effort and cause small increases in breathing or heart rate.

Work

**Q1**	Does your work involve vigorous-intensity activity that causes large increases in breathing or heart rate like carrying or lifting heavy loads, digging or construction work for at least 10 minutes continuously?	Yes / No<br>If No → go to Q4	
**Q2**	In a typical week, on how many days do you do vigorous- intensity activities as part of your work?	Days	
**Q3**	How much time do you spend doing vigorous-intensity activities at work on a typical day?	Hours	Mins
**Q4**	Does your work involve moderate-intensity activity, that causes small increases in breathing or heart rate such as brisk walking [or carrying light loads] for at least 10 minutes continuously?	Yes / No<br>If No → go to Q7	
**Q5**	In a typical week, on how many days do you do moderate- intensity activities as part of your work?	Days	
**Q6**	How much time do you spend doing moderate-intensity activities at work on a typical day?	Hours	Mins

Travel to and from places

The next questions exclude the physical activities at work that you have already mentioned. Now I would like to ask you about the usual way you travel to and from places. For example to work, for shopping, to market, to place of worship.

**Q7**	Do you walk or use a bicycle (pedal cycle) for at least 10 minutes continuously to get to and from places?	Yes / No<br>If No → go to Q10	
**Q8**	In a typical week, on how many days do you walk or bicycle for at least 10 minutes continuously to get to and from places?	Days	
**Q9**	How much time do you spend walking or bicycling for travel on a typical day?	Hours	Mins

Participant ID	
Participant initials	

iHealth-T2D

Recreational activities		
The next questions exclude the work and transport activities that you have already mentioned. Now I would like to ask you about sports, fitness and recreational activities (leisure), [Insert relevant terms].		
**Q10**	Do you do any vigorous-intensity sports, fitness or recreational (leisure) activities that cause large increases in breathing or heart rate like [running or football] for at least 10 minutes continuously?	Yes / No<br>If No → go to Q13
**Q11**	In a typical week, on how many days do you do vigorous- intensity sports, fitness or recreational (leisure) activities?	Days
**Q12**	How much time do you spend doing vigorous-intensity sports, fitness or recreational activities on a typical day?	Hours | Mins
**Q13**	Do you do any moderate-intensity sports, fitness or recreational (leisure) activities that cause a small increase in breathing or heart rate such as brisk walking, [cycling, swimming, volleyball] for at least 10 minutes continuously?	Yes / No<br>If No → go to Q16
**Q14**	In a typical week, on how many days do you do moderate- intensity sports, fitness or recreational (leisure) activities?	Days
**Q15**	How much time do you spend doing moderate-intensity sports, fitness or recreational (leisure) activities on a typical day?	Hours | Mins
**Sedentary behaviour**		
The following question is about sitting or reclining at work, at home, getting to and from places, or with friends including time spent sitting at a desk, sitting with friends, traveling in car, bus, train, reading, playing cards or watching television, but do not include time spent sleeping		
**Q16**	How much time do you usually spend sitting or reclining on a typical day?	Hours | Mins

Participant ID	
Participant initials	

iHealth-T2D

Relative Data: Use ONE sheet per consenting relative

Relative name:					
Relative ID:	"Participant ID" - 1	DOB:	DD/MM/YYYY	Sex:	M/F
Relationship to participant:	The participant is my:				
Does the relative cook?	Y/N	Is the relative the main cook of the household?	Y/N		

	Height (m):	BMI (kg/m^2)	Weight (kg)	Waist (cm)	Exercise (mins/week)
Start					
Target					>150

Visit	Attended?	Weight (kg)	Waist (cm)	Hip (cm)	Lose / Maintain weight	Calorie prescription (kcal)
Clinic visit 1	Y/N				Lose / Maintain	
Clinic visit 2	Y/N				Lose / Maintain	
Clinic visit 3	Y/N				Lose / Maintain	
Clinic visit 4	Y/N				Lose / Maintain	
Clinic visit 5	Y/N				Lose / Maintain	
Clinic visit 6	Y/N				Lose / Maintain	
Clinic visit 7	Y/N				Lose / Maintain	
Clinic visit 8	Y/N				Lose / Maintain	
Clinic visit 9	Y/N				Lose / Maintain	

Participant ID	
Participant initials	

iHealth-T2D

Relative Data: Use ONE sheet per consenting relative

Relative name:					
Relative ID:	"Participant ID" - 2	DOB:	DD/MM/YYYY	Sex:	M/F
Relationship to participant:	The participant is my:				
Does the relative cook?	Y/N	Is the relative the main cook of the household?	Y/N		

	Height (m):	BMI (kg/m²)	Weight (kg)	Waist (cm)	Exercise (mins/week)
Start					
Target					>150

Visit	Attended?	Weight (kg)	Waist (cm)	Hip (cm)	Lose / Maintain weight	Calorie prescription (kcal)
Clinic visit 1	Y/N				Lose / Maintain	
Clinic visit 2	Y/N				Lose / Maintain	
Clinic visit 3	Y/N				Lose / Maintain	
Clinic visit 4	Y/N				Lose / Maintain	
Clinic visit 5	Y/N				Lose / Maintain	
Clinic visit 6	Y/N				Lose / Maintain	
Clinic visit 7	Y/N				Lose / Maintain	
Clinic visit 8	Y/N				Lose / Maintain	
Clinic visit 9	Y/N				Lose / Maintain	

Participant ID	
Participant initials	

iHealth-T2D

Relative Data: Use ONE sheet per consenting relative

Relative name:					
Relative ID:	"Participant ID" - 3	DOB:	DD/MM/YYYY	Sex:	M/F
Relationship to participant:	The participant is my:				

Does the relative cook?	Y/N	Is the relative the main cook of the household?	Y/N

	Height (m):	BMI (kg/m^2)	Weight (kg)	Waist (cm)	Exercise (mins/week)
Start					
Target					>150

Visit	Attended?	Weight (kg)	Waist (cm)	Hip (cm)	Lose / Maintain weight	Calorie prescription (kcal)
Clinic visit 1	Y/N				Lose / Maintain	
Clinic visit 2	Y/N				Lose / Maintain	
Clinic visit 3	Y/N				Lose / Maintain	
Clinic visit 4	Y/N				Lose / Maintain	
Clinic visit 5	Y/N				Lose / Maintain	
Clinic visit 6	Y/N				Lose / Maintain	
Clinic visit 7	Y/N				Lose / Maintain	
Clinic visit 8	Y/N				Lose / Maintain	
Clinic visit 9	Y/N				Lose / Maintain	

**Participant ID**	
**Participant initials**	

iHealth-T2D

Relative Data: Use ONE sheet per consenting relative

**Relative name:**					
**Relative ID:**	"Participant ID" **- 4**	**DOB:**	DD/MM/YYYY	**Sex:**	M/F
**Relationship to participant:**	The participant is my:				
**Does the relative cook?**	Y/N	**Is the relative the main cook of the household?**	Y/N		

	Height (m):	BMI (kg/m^2)	Weight (kg)	Waist (cm)	Exercise (mins/week)
**Start**					
**Target**					>150

Visit	Attended?	Weight (kg)	Waist (cm)	Hip (cm)	Lose / Maintain weight	Calorie prescription (kcal)
**Clinic visit 1**	Y/N				Lose / Maintain	
**Clinic visit 2**	Y/N				Lose / Maintain	
**Clinic visit 3**	Y/N				Lose / Maintain	
**Clinic visit 4**	Y/N				Lose / Maintain	
**Clinic visit 5**	Y/N				Lose / Maintain	
**Clinic visit 6**	Y/N				Lose / Maintain	
**Clinic visit 7**	Y/N				Lose / Maintain	
**Clinic visit 8**	Y/N				Lose / Maintain	
**Clinic visit 9**	Y/N				Lose / Maintain	

Participant ID	
Participant initials	

iHealth-T2D

Relative Data: Use ONE sheet per consenting relative

Relative name:					
Relative ID:	"Participant ID" - 5	DOB:	DD/MM/YYYY	Sex:	M/F
Relationship to participant:	The participant is my:				

Does the relative cook?	Y/N	Is the relative the main cook of the household?	Y/N

	Height (m):	BMI (kg/m^2)	Weight (kg)	Waist (cm)	Exercise (mins/week)
Start					
Target					>150

Visit	Attended?	Weight (kg)	Waist (cm)	Hip (cm)	Lose / Maintain weight	Calorie prescription (kcal)
Clinic visit 1	Y/N				Lose / Maintain	
Clinic visit 2	Y/N				Lose / Maintain	
Clinic visit 3	Y/N				Lose / Maintain	
Clinic visit 4	Y/N				Lose / Maintain	
Clinic visit 5	Y/N				Lose / Maintain	
Clinic visit 6	Y/N				Lose / Maintain	
Clinic visit 7	Y/N				Lose / Maintain	
Clinic visit 8	Y/N				Lose / Maintain	
Clinic visit 9	Y/N				Lose / Maintain	

ADD EXTRA RELATIVE SHEETS AS NECESSARY
