# Supplementary material for: The iHealth-T2D study, prevention of type 2 diabetes amongst South Asians with central obesity and prediabetes: study protocol for a randomised controlled trial
Source: Trials. 2021 Dec 18;22:928. doi: 10.1186/s13063-021-05803-7 (PMC8684177; doi:10.1186/s13063-021-05803-7)
Supplement: Supplementary file 1 — Additional file 1: S1. CHW Handbook. Training manual summarising the content and delivery of the lifestyle intervention. Translated versions available through study website. S2. CHW Workbook. Case report form / intervention delivery workbook for the lifestyle intervention. Translated versions available through study website. S3. Participant Handbook for Lifestyle intervention. Written materials provided to the participant to support the CHW delivered lifestyle intervention. Translated versions available through study website. S4. Participant Handbook for Usual care. Translated versions available through study website. Supplementary Table 1: The a-priori power calculations under alternate assumptions with a sample size of 3,600 participants. [file 13063_2021_5803_MOESM1_ESM.zip › Addcorr2.zip!/S3 Participant booklet.zip!/21526 Clinic visit 1 V3 England_7.pdf]

i HEALTH
T 2 D

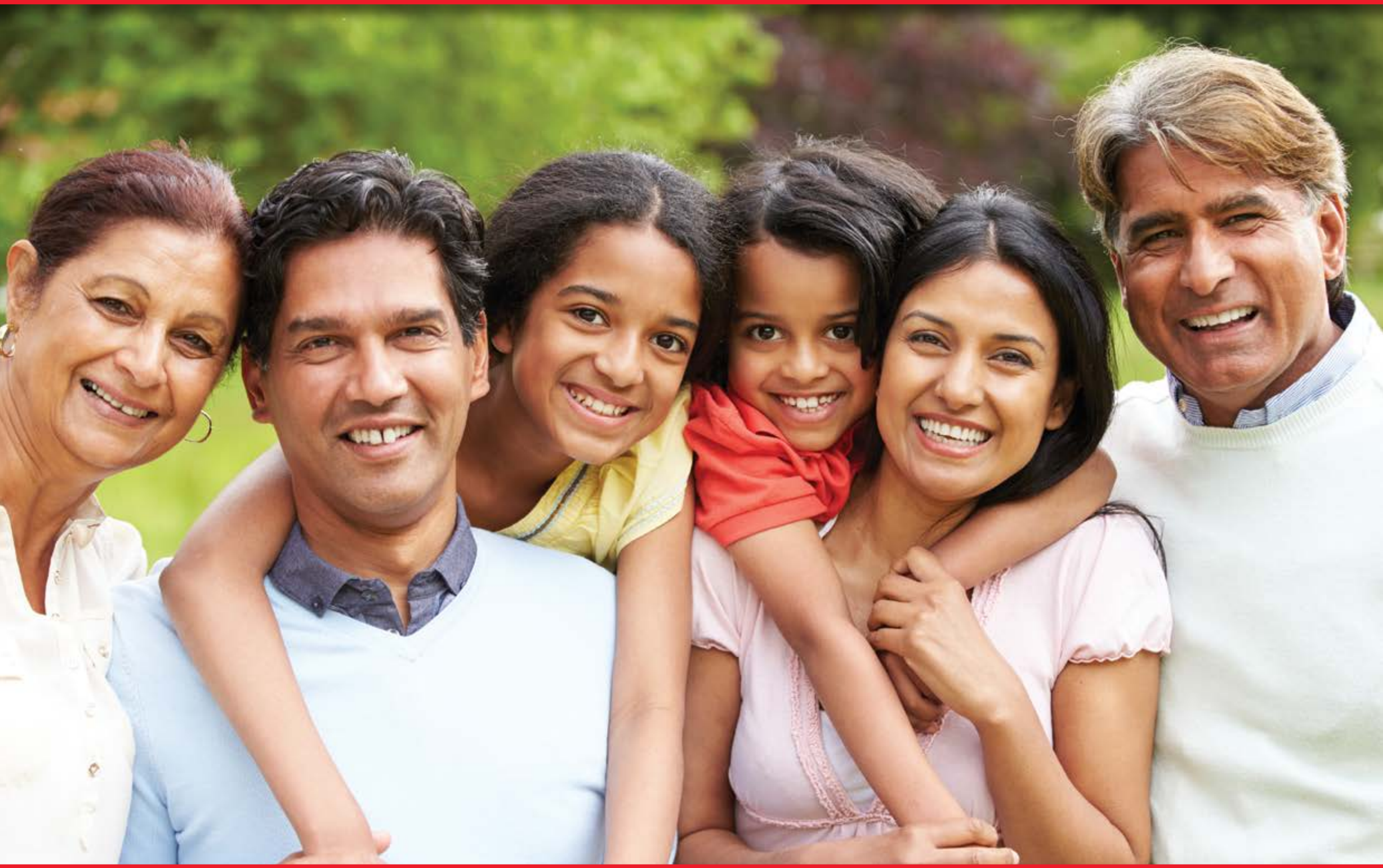

Participant booklet

Lifestyle intervention to prevent Type-2 Diabetes amongst South Asians with central obesity and prediabetes

Horizon 2020
European Union Funding
for Research & Innovation

Name:	
CHW Name:	
Study ID:	
Contact Tel:	

Episode	Visit	Week	My appointment dates	My appointment times
1	Visit 1	1		
2	Phonecall	2		
3	Visit 2	3		
4	Phonecall	5		
5	Visit 3	7		
6	Phonecall	9		
7	Visit 4	11		
8	Phonecall	13		
9	Visit 5	15		
10	Phonecall	17		
11	Visit 6	19		
12	Phonecall	21		
13	Phonecall	23		
14	Visit 7	25		
15	Phonecall	28		
16	Phonecall	31		
17	Phonecall	34		
18	Visit 8	37		
19	Phonecall	40		
20	Phonecall	43		
21	Phonecall	46		
22	Visit 9	49		

Welcome!

The lifestyle intervention programme you have joined lasts 12 months and includes a total of 22 sessions with your Health Educator (a community health worker, CHW). Nine of these sessions will be face-to-face clinic appointments, the others will be telephone sessions.

During this time you and your CHW will discuss many aspects of your eating behaviour and physical activity and together, develop ways in which you can improve your diet and lifestyle. You will set your own personal goals at every visit and your CHW is here to help you achieve these goals at every step throughout the year.

Other family members are strongly encouraged to attend the clinic appointments with you, particularly if they are overweight or the main cook of the family unit.

At the end of the year you and your family will have the knowledge and skills to lead a healthy lifestyle for the rest of your lives.

My progress

	Date	Body weight (kg)	Weight loss since last visit (kg)	Waist circumference (cm)
Clinic visit 1				
Clinic visit 2				
Clinic visit 3				
Clinic visit 4				
Clinic visit 5				
Clinic visit 6				
Clinic visit 7				
Clinic visit 8				
Clinic visit 9				

Lifestyle intervention overview

This outlines what will be covered at each visit. It is important that you attend all appointments. In between clinic appointments, there will be telephone reviews where you can discuss any queries or problems, and go through goals set in the previous appointment.

Clinic visit 1: Personal session

- Why I am here
- Programme goals
- Weight loss and physical activity goals
- Record keeping
- Lapses and relapses
- Physical activity
- Set a personal target for this week

Clinic visit 2: Personal session

- Review your experience since the last meeting
- Looking at your food & activity diary
- The plate model
- The 5 food groups
- Daily diet plan
- Set your next personal target

Clinic visit 3, 4 and 5: Group sessions

Visit 3

- Review your experience since the last meeting
- Focus on fat
- Set your next personal target

Visit 4

- Review your experience since the last meeting
- Carbohydrate, fibre and sugar
- Trigger foods and trigger situations
- Eating behaviour cycle
- Set your next personal target

Visit 5

- Review your experience since the last meeting
- Food labels and shopping
- Eating out/holidays/special events
- Dealing with pressures to eat
- Set your next personal target

Clinic visit 6: Personal session

At the 6th appointment, there will be a review of your lifestyle achievements so far and a discussion around whether you would like to continue trying to lose weight or maintain the weight that has already been lost and how to achieve that.

Clinic visit 7 and 8: Group sessions

Group sessions 7 and 8 provide an opportunity to ask questions and discuss with the CHW and the group any aspect of your lifestyle plan that you are concerned or pleased about. It's a chance to learn from each other and to share experiences, problem solving, recipes and tips.

Final Clinic visit 9: Personal session

At the 9th appointment, there will be a review of your lifestyle achievements.

Telephone calls: Personal session

You will have regular telephone calls with your CHW in between all your visits. They are a chance to ask for help or guidance or to discuss any issues or triumphs that you wish.

Visit 1

Why am I here?

You were invited to take part in this programme because you are overweight or you are pre-diabetic or both. Both of these things mean that you are at higher risk of developing complications associated with excess body weight or high blood sugar.

Within 5 years, half of people with pre-diabetes will develop type 2 diabetes

The good news is that the vast majority of cases of type 2 diabetes can be prevented through making lifestyle changes.

Maintaining a healthy weight and eating a healthy balanced diet, low in fat and rich in fibre and fruit and vegetables, as well as being physically active is the best way of reducing your risk. Even if you're not overweight, maintaining a healthy weight through eating well and being active is an important part of managing blood sugar levels and avoiding other health complications.

Type 2 diabetes can be prevented by adopting a healthy lifestyle

Complications with being overweight and getting type 2 diabetes

Being overweight can cause problems, from difficulties with daily activities to serious health conditions, like type 2 diabetes. Diabetes is a lifelong condition that causes a person's blood sugar level to become too high. High blood sugar levels can damage blood vessels, nerves and organs.

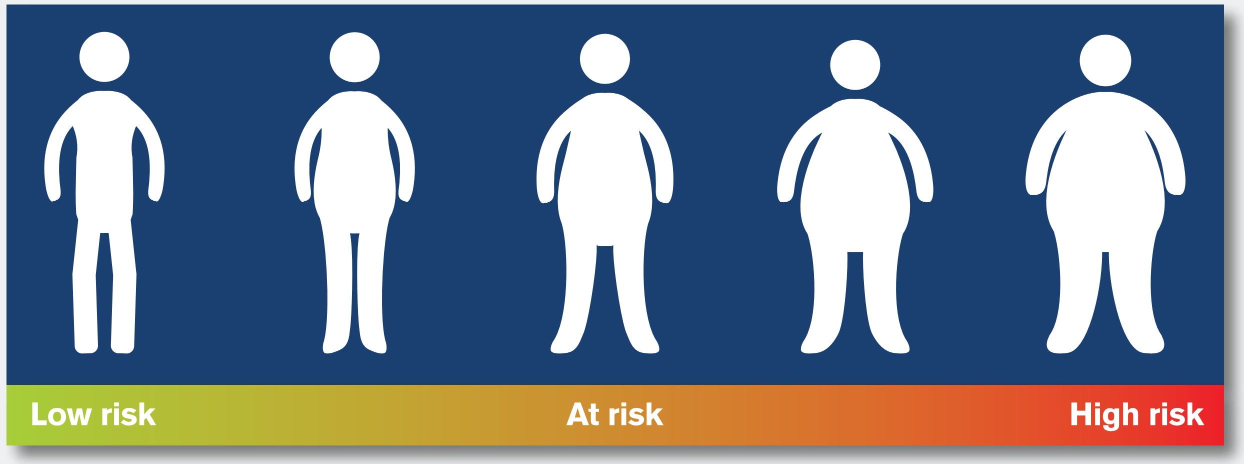

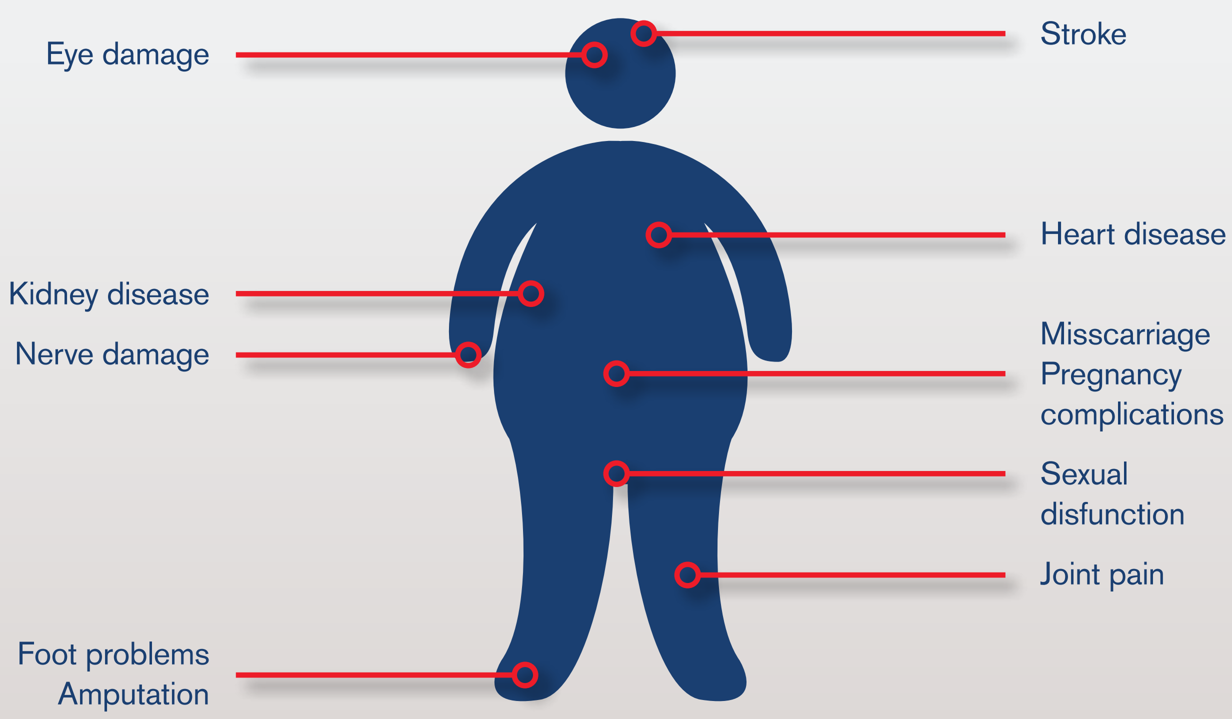

Programme Goals

Weight loss target

10% body weight

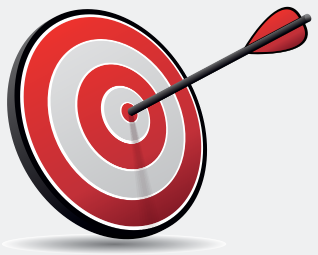

Physical activity target

30 minutes each day

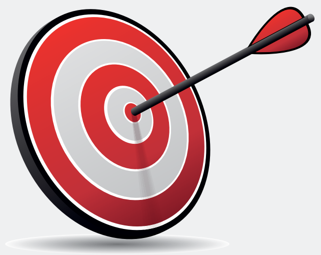

Realistic weight loss

When starting a weight loss programme many people have an 'ideal weight' in mind. This may not be necessary or achievable now. Scientists have shown that losing as little as 10% of your current body weight can vastly improve your health. Even a 5-7% weight loss has been shown to have benefits.

Benefits of 10 % Weight Loss

Reduces risk of heart disease, diabetes and cancer. Improve the chance you will live a longer and healthier life.

↓ blood pressure

↓ cholesterol

↓ triglycerides (fat in blood)

↓ glucose (sugar in blood)

**My current weight is**	
**10% of this weight is**	
**My target weight is**	

Think about what a 10% weight loss would mean to you. You may not think it is enough, or you may think it is plenty, however, a 10% weight loss aim is a good starting point

Another measure of excess fat is waist circumference, and can be used as an additional measure in people who are overweight.

	Ideal	Seriously at Risk
**South Asian Men**	Less than 90 cm (35.5 in)	More than 90 cm (35.5 in)
**South Asian Women**	Less than 80 cm (31 in)	More than80 cm (31 in)

Physical activity

Even without weight loss, increasing your level of physical activity will have health benefits. It doesn't have to be intense exercise if you cannot manage that at the moment. Doing a minimum of 30 minutes of moderate intensity activity, such as brisk walking or housework, each day will help. As long as you are slightly hot and your breathing rate increases – it all counts as exercise!

My reasons for weight gain

Losing weight can be very difficult; Understanding the reasons why you have gained weight can provide insight into how best to tackle losing weight for you. Think about some of these reasons and note them down below.

E.g.	***'I have a healthy diet, but I snack too much';*** ***'My wife does the cooking and uses too much oil'***
**1**	
**2**	
**3**	
**4**	

Importance of weight loss

How **important** is it for you to lose weight in the next 6 months? (please mark from 1-10)

1	2	3	4	5	6	7	8	9	10
Not important at all		Fairly important			important				Very important

Why are you at …….. and not at …….?

What would it take to move you up a couple of points?

Confidence

How **confident** are you that you can lose weight to in the next 6 months? (please mark from 1-10)

1	2	3	4	5	6	7	8	9	10
Not confident at all		Fairly confident			confident				Very confident

Why are you at …….. and not at …….?

What would it take to move you up a couple of points?

What would be a good first step?

What obstacles do you foresee and how might you deal with them?

My motivation

Try to identify 3 Reasons why you want to try and lose some weight.

Reason 1

Reason 2

Reason 3

Remind yourself of these when you are finding it difficult.

Record keeping

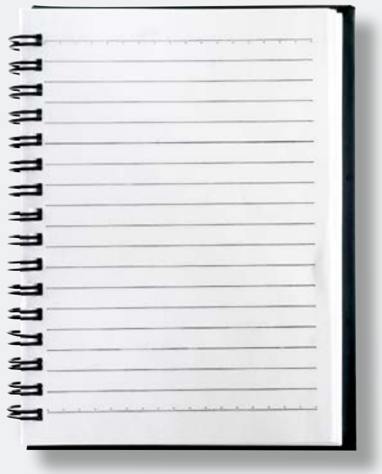

This is a very important part of your weight loss programme. Studies have shown that people who keep records of what they are eating are more likely to succeed at losing weight. Keeping records makes you more aware of what, why and when you are eating.

Try to record what you eat every day for the next 6 months. You may find this very difficult at first, but once you get into the habit of writing down what you eat and when you eat it, it will become very easy.

Record everything that you eat

It doesn't matter how big or how small the item is, write it down. The more descriptive you are the better. Record how many grapes you picked at while waiting for your toast, how many spoons of sugar you put in your tea, and how many grams/ounces of pasta, meat, or chicken you had for dinner.

Record your mood and physical activity

Also record your mood when eating, to see if this affects what you eat. You may find that you eat more when you are happy or sad, angry or anxious. It is very helpful to know what triggers a change in your eating habits.

When you do physical activity during the day write it down. It will be encouraging to see how you are managing to make changes to your lifestyle.

Carry your food diary everywhere you go and record immediately after eating

Get a small note book that you can carry with you so that you always have the opportunity to write down exactly what you eat, when you eat it. It will soon become something that you don't leave the house without.

Try not to wait until the end of the day or even the next day to record what you have consumed. It is very easy to forget the handful of crisps you had when out drinking in the pub or what you had while preparing the evening meal. By writing things down immediately that you eat them, we hope you will focus on what you really want to eat, as opposed to foods you eat just for the sake of it.

Please bring at least one week (7 days) food diary with you when you come for visit 2

This is so you and your CHW can begin to understand your eating habits and discuss aspects that you may wish to change about your diet. Your CHW can help you to come up with ideas of ways in which you can make changes to your diet. It may be that there are one or two weeks when you don't lose weight. Looking over your food record with the community health worker may help you work out why this may have happened.

Lapses and relapses

What is a lapse?

- A lapse is a temporary (one-off) slip back from your eating or activity plan.
- You should expect to have lapses from time to time, after all you are only human.
- One lapse is not going to undo all your hard work. What you do following the lapse is the most important thing.

What causes a lapse?

- Lapses do not come out of the blue; they are usually triggered by something.
- Think about the times you have not kept to your eating and activity goals since attending this programme.
  - Is it when you have certain feelings, like loneliness or frustration?
  - Is it when you have to deal with a particular person or event?
  - Is it when somebody offers you food?
  - Is it when you are eating with friends or when you eating alone?
- If you are aware of these 'triggers' and 'risky situations' you can do something about them.

How to recover after a lapse?

- Stop – try to avoid the situation.
- Stay Calm – don't panic. One lapse does not mean failure, say to yourself 'its not a disaster, one slip does not mean the end of the world.
- Think – what was going on before the lapse? What were the triggers? Analyse the situation using an eating or behavioural chain.
- Regain control – do something else, dispose of the problem food if any remains.
- Do not starve yourself – eat your next meal as usual. Depriving yourself can set you up to binge eat.
- Learn and Plan – what can be done in the same situation to prevent another lapse?
- Be Positive – remind yourself of how much you have achieved so far.

Remember
A lapse isn't a failure. Expect them to happen
Learn to limit how often they happen by recognising your triggers
It's what you do after a lapse that is important!

Benefits of a More Active Lifestyle

Living an active life should be considered as the normal state of existence. There is a lot of evidence to show that the more active a person is, the more protection they receive from many diseases. The following list shows further benefits of being more active:

- **Burns calories** stored in fat, helping weight loss.
- **Preserves the body's muscle and bone**. Being active will maximise fat loss, and minimise the loss of healthy muscle. This will help improve your body shape. Being active also makes your bones stronger reducing your risk of fragile bones (osteoporosis).
- **Increases your metabolic rate**. Eating less and losing weight slows down your metabolism slightly. Improving your levels of physical activity can help offset this drop in metabolic rate.
- **Helps appetite control** by improving your sensitivity to what your body needs.
- **Improves your health**. Physical activity can lower blood pressure, improve your cholesterol profile and improve the control of diabetes.
- **Improve confidence and well being**. Getting fit will make you feel better. Many people do physical activity to relieve stress. If you are one of those people who eat to relieve stress, physical activity can act in the same way but will use up, rather than add calories.
- **Predicts long term success**. Being physically active is the factor that best predicts who will lose weight and keep it off for good.

What Type of Activity Should I do?

Choosing an activity you enjoy is most important and any activities that increase your heart rate and make you warm and slightly out of breath are suitable. Aim for 30 minutes each day.

- Using stairs instead of the lift or escalators
- Getting off the bus a few stops earlier
- DIY
- Gardening
- Housework
- Walking the dog
- Walking to the local shops
- Walking the children to school
- Walking to work

Reduce periods of sitting to less than 30-minutes at a time

- Golf
- Football
- Tennis
- Bowls
- Snooker
- Tenpin Bowling
- Hiking
- Netball
- Brisk Walking
- Swimming
- Aerobics
- Cycling
- Rowing
- Bhangra
- Dancing

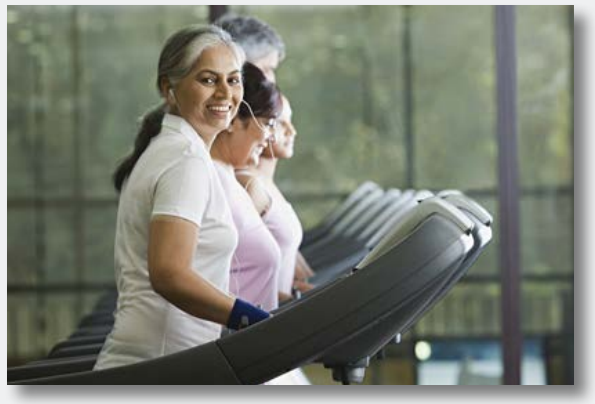

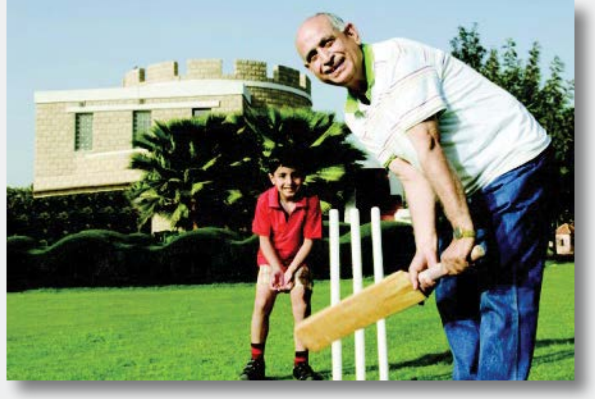

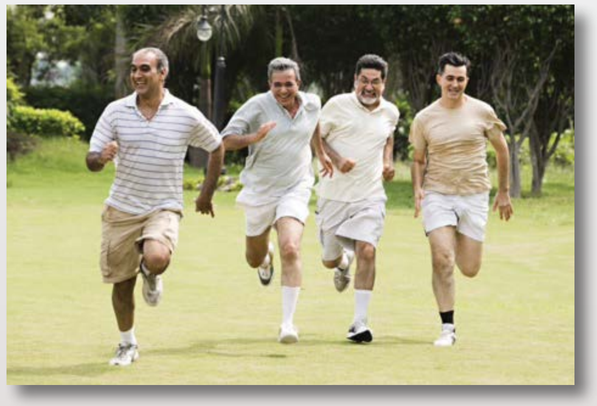

Remember, every little bit helps

Every calorie we burn makes a difference. For example, if you are only able to manage an EXTRA four, 3 minute walks each day, you would lose an extra ½ kg (1 lb) in weight each month. Over 12 months this would add up to 6 kg (12lbs).

An Example of a Walking Programme

This example is for someone who has not been physically active for a long period of time. It emphasises the importance of gradually increasing the intensity and duration of physical activity. Start off with physical activity that is within your physical ability e.g. 10 minutes of walking at a comfortable pace.

Week 1: 3 sessions of 10 minute walks at a comfortable pace

Week 2: 3 sessions of 12 minute walks at a comfortable pace

Week 3: 3 sessions of 14 minute walks at a comfortable pace

Weeks 4-11: Build this up to 30 minutes of walking by the 11th week

Week 12: Once you are up to 30 minutes of walking, start increasing your walking speed using a watch with a minute hand, walk for 5 minutes not too fast as a warm up.

Follow this by walking at a brisk pace for 5 minutes and then 5 minutes at a slower pace to allow your heart and breathing rate to return to normal. The fast walking speed should just leave you slightly out of breath.

Repeat this sequence for 30 minutes. This type of fast/slow walking will work your musculoskeletal system differently and strengthen your heart and lungs so that you can walk at a faster speed non-stop for 30 minutes.

Energy used during 30 minutes of activity

Activity		Calories burned during 30min of activity	
		For a person weighing 70kg	For a person weighing 100kg
**Sedentary activity**	• Sitting	45	63
	• Standing	54	75
**Household or occupational activity**	• Cleaning	132	183
	• Cooking	96	132
	• Shopping	132	183
	• Scrubbing	231	321
	• Digging	309	426
	• Painting	165	225
	• Gardening (mowing)	228	330
	• Gardening (raking)	114	159
**Recreational or leisure exercise**	• Walking (leisurely)	171	234
	• Walking (briskly)	255	353
	• Social dancing	108	150
	• Cycling	135	189
	• Running (11.5min/mile)	276	399
	• Swimming (crawl)	273	375
	• Weight training	246	342
	• Badminton	207	285
	• Golf	180	249
	• Table tennis	144	201
	• Tennis	231	321

Remember
It is not easy to burn off calories from extra food eaten. For example a chocolate bar containing 350 calories can be eaten in the space of a few minutes. However, you would need to walk briskly for 1-1 ½ hours to burn off the calories contained in this snack. It is therefore very important to combine exercise with healthy eating to bring about weight loss.

Visit 1: Personal Target Setting

Date	Weight	Waist Circumference

Targets Set
1
2
3
4
5
6

You may find it useful to ask yourself the following questions to help you achieve your targets:

How will I go about achieving this target? How will I know if I have met this target? Is it realistic for me to achieve this target in the amount of time I have available?

Targets Met
1
2
3
4
5
6

Notes

Notes

Horizon 2020
European Union Funding
for Research & Innovation

Designed by ESP Bioscience 2016
http://www.espstc.com

iHealth-T2D: Visit 1 – UK – English – version 1
