# Supplementary material for: The iHealth-T2D study, prevention of type 2 diabetes amongst South Asians with central obesity and prediabetes: study protocol for a randomised controlled trial
Source: Trials. 2021 Dec 18;22:928. doi: 10.1186/s13063-021-05803-7 (PMC8684177; doi:10.1186/s13063-021-05803-7)
Supplement: Supplementary file 1 — Additional file 1: S1. CHW Handbook. Training manual summarising the content and delivery of the lifestyle intervention. Translated versions available through study website. S2. CHW Workbook. Case report form / intervention delivery workbook for the lifestyle intervention. Translated versions available through study website. S3. Participant Handbook for Lifestyle intervention. Written materials provided to the participant to support the CHW delivered lifestyle intervention. Translated versions available through study website. S4. Participant Handbook for Usual care. Translated versions available through study website. Supplementary Table 1: The a-priori power calculations under alternate assumptions with a sample size of 3,600 participants. [file 13063_2021_5803_MOESM1_ESM.zip › Addcorr2.zip!/S4 Usual-Care-booklet-UK-English.pdf]

Working towards a healthy lifestyle: Preventing Diabetes

You were invited to take part in this study because you are overweight or you are pre-diabetic or both. Both of these things mean that you are at higher risk of developing complications associated with excess body weight or high blood sugar.

Within 5 years, half of people with pre-diabetes will develop type 2 diabetes

The good news is that the vast majority of cases of type 2 diabetes can be prevented through making lifestyle changes.

Maintaining a healthy weight and eating a healthy balanced diet, low in fat and rich in fibre and fruit and vegetables, as well as being physically active is the best way of reducing your risk. Even if you're not overweight, maintaining a healthy weight through eating well and being active is an important part of managing blood sugar levels and avoiding other health complications.

Type 2 diabetes can be prevented by adopting a healthy lifestyle

Complications with being overweight and getting type 2 diabetes

Being overweight can cause problems, from difficulties with daily activities to serious health conditions, like type 2 diabetes. Diabetes is a lifelong condition that causes a person's blood sugar level to become too high. High blood sugar levels can damage blood vessels, nerves and organs.

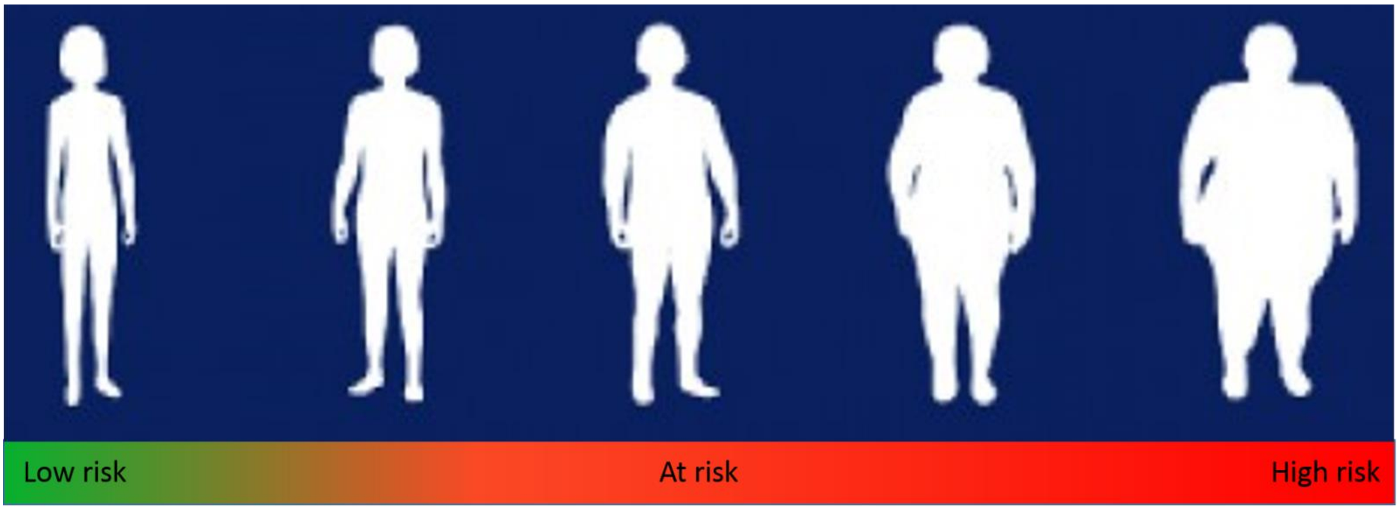

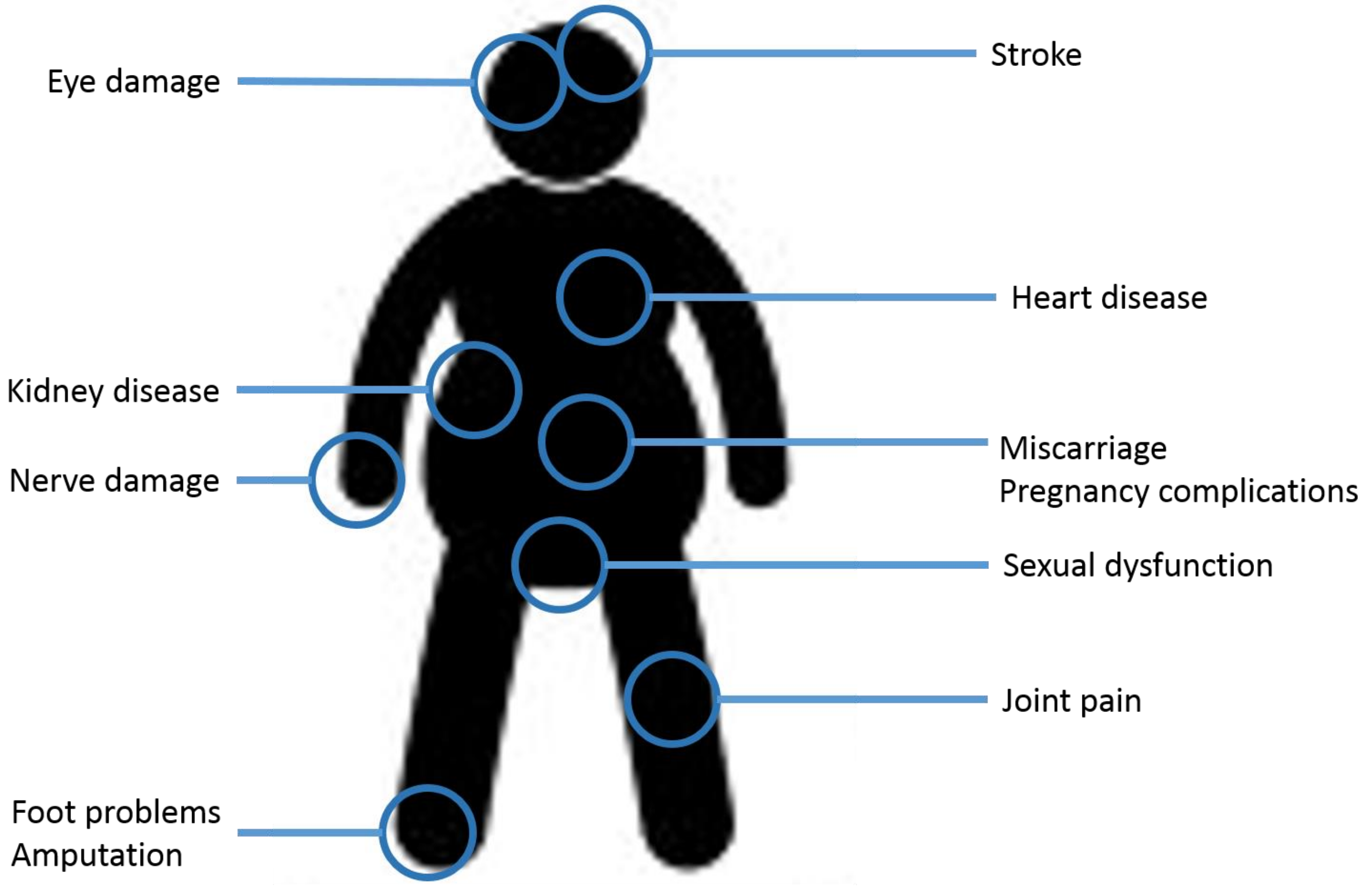

Healthy Lifestyle Goals

Weight loss target

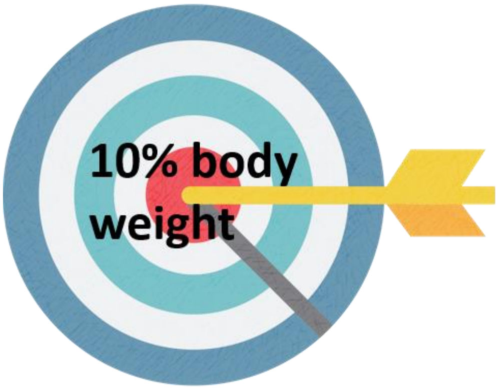

Physical activity target

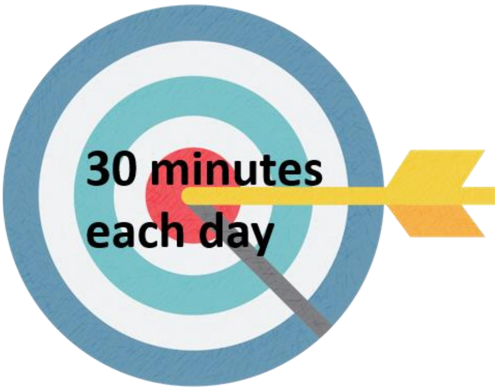

Realistic weight loss

When starting to lose weight many people have an 'ideal weight' in mind. This may not be necessary or achievable immediately. Scientists have shown that losing as little as 10% of your current body weight can vastly improve your health. Even a 5-7% weight loss has been shown to have benefits.

Benefits of 10 % Weight Loss

Reduces risk of heart disease, diabetes and cancer
Improve the chance you will live a longer and healthier life

↓ blood pressure	↓ triglycerides (fat in blood)
↓ cholesterol	↓ glucose (sugar in blood)

**My current weight is:**	
**10% of this weight is:**	
**My target weight is:**	

Think about what a 10% weight loss would mean to you. You may not think it is enough, or you may think it is plenty, however, a 10% weight loss aim is a good starting point.

Another measure of excess fat is waist circumference, and can be used as an additional measure in people who are overweight.

	**Ideal:**	**Seriously at Risk:**
**South Asian Men**	Less than 90 cm (35.5 in)	More than 90 cm (35.5 in)
**South Asian Women**	Less than 80 cm (31 in)	More than80 cm (31 in)

Physical activity

Even without weight loss, increasing your level of physical activity will have health benefits. It doesn't have to be intense exercise if you cannot manage that at the moment. Doing a minimum of 30 minutes of moderate intensity activity, such as brisk walking or housework, each day will help. As long as you are slightly hot and your breathing rate increases – it all counts as exercise!

Energy balance

The only way to lose weight and keep it off is to change your eating and physical activity habits permanently. Your body weight represents the balance between all the energy (calories) taken in from the food and drink you eat, and all the energy (calories) used up during your daily activities.

To maintain your weight, the amount of energy you take in from food and drink has to equal the amount of energy you use up in your daily activities.

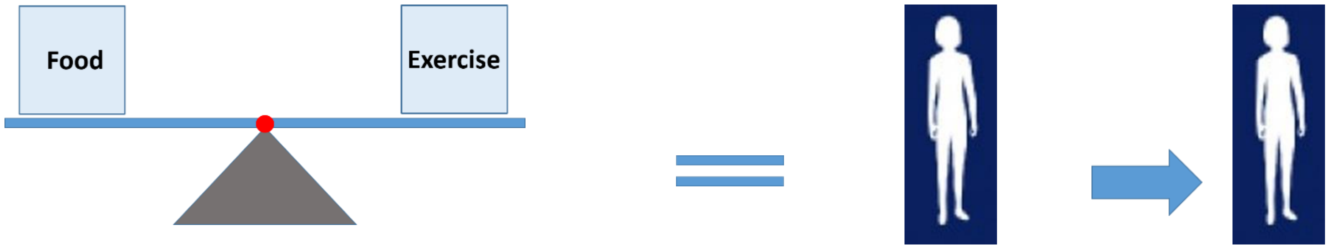

To put on weight, means that over time, the energy you have taken in has been more than the energy you have used up. The difference is usually small, typically only 50-100 extra calories daily.

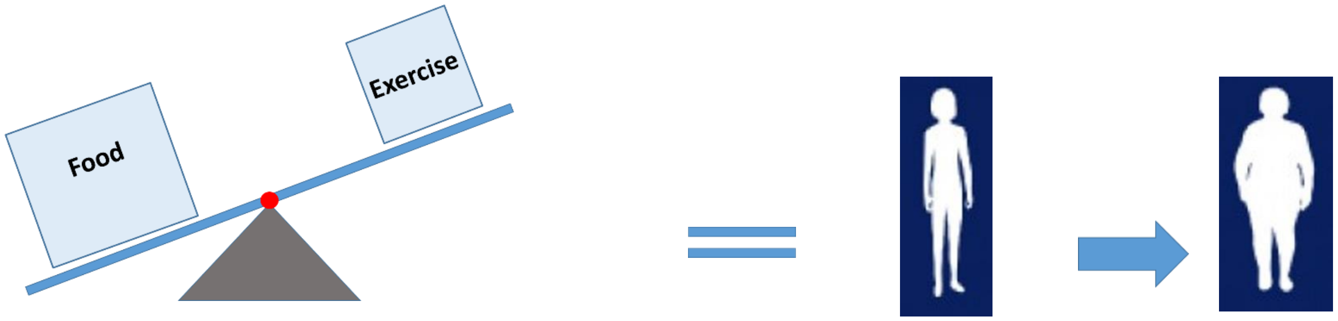

To lose weight, you need to eat fewer calories and/or do more physical activity. Your eating plan has been personally designed for you. By following this plan you should hope to lose at least ½ kg or 1lb per week.

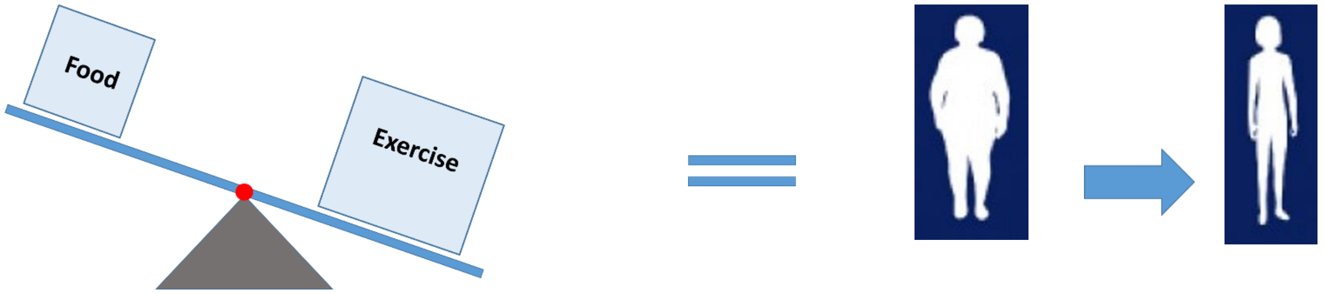

My reasons for weight gain

Losing weight can be very difficult; Understanding the reasons why you have gained weight can provide insight into how best to tackle losing weight for you. Think about some of these reasons and note them down below.

E.g.	***'I have a healthy diet, but I snack too much';*** ***'My wife does the cooking and uses too much oil'***
1	
2	
3	
4	

My motivation

Try to identify 3 Reasons why you want to try and lose some weight.

Reason 1

Reason 2

Reason 3

Remind yourself of these when you are finding it difficult.

Record keeping

This is a very important part of trying to lose weight. Studies have shown that people who keep records of what they are eating are more likely to succeed at losing weight. Keeping records makes you more aware of what, why and when you are eating.

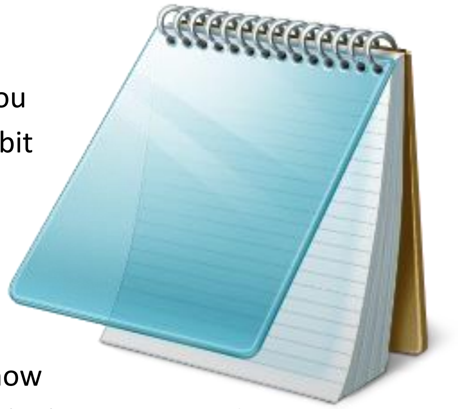

Try to record what you eat every day for the next 6 months. You may find this very difficult at first, but once you get into the habit of writing down what you eat and when you eat it, it will become very easy.

Record everything that you eat: It doesn't matter how big or how small the item is, write it down. The more descriptive you are the better. Record how many grapes you picked at while waiting for your toast, how many spoons of sugar you put in your tea, and how many grams/ounces of pasta, meat, or chicken you had for dinner.

Record your mood and physical activity: Record your mood when eating, to see if this affects what you eat. You may find that you eat more when you are happy or sad, angry or anxious. It is very helpful to know what triggers a change in your eating habits.

When you do physical activity during the day write it down. It will be encouraging to see how you are managing to make changes to your lifestyle.

Carry your food diary everywhere you go and record immediately after eating: Get a small note book that you can carry with you so that you always have the opportunity to write down exactly what you eat, when you eat it. It will soon become something that you don't leave the house without.

Try not to wait until the end of the day or even the next day to record what you have consumed. It is very easy to forget the handful of crisps you had when out drinking in the pub or what you had while preparing the evening meal. By writing things down immediately that you eat them, we hope you will focus on what you really want to eat, as opposed to foods you eat just for the sake of it.

Benefits of a More Active Lifestyle

Living an active life should be considered as the normal state of existence. There is a lot of evidence to show that the more active a person is, the more protection they receive from many diseases. The following list shows further benefits of being more active:

- **Burns calories** stored in fat, helping weight loss.

- **Preserves the body's muscle and bone.** Being active will maximise fat loss, and minimise the loss of healthy muscle. This will help improve your body shape. Being active also makes your bones stronger reducing your risk of fragile bones (osteoporosis).

- **Increases your metabolic rate.** Eating less and losing weight slows down your metabolism slightly. Improving your levels of physical activity can help offset this drop in metabolic rate.

- **Helps appetite control** by improving your sensitivity to what your body needs.

- **Improves your health.** Physical activity can lower blood pressure, improve your cholesterol profile and improve the control of diabetes.

- **Improve confidence and well being.** Getting fit will make you feel better. Many people do physical activity to relieve stress. If you are one of those people who eat to relieve stress, physical activity can act in the same way but will use up, rather than add calories.

- **Predicts long term success.** Being physically active is the factor that best predicts who will lose weight and keep it off for good.

What Type of Activity Should I do?

Choosing an activity you enjoy is most important and any activities that increase your heart rate and make you warm and slightly out of breath are suitable. Aim for 30 minutes each day.

- **Using stairs instead of the lift or escalators**
- **Getting off the bus a few stops earlier**
- **DIY**
- **Gardening**

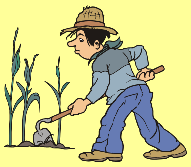

- **Housework**
- **Walking the dog**
- **Walking to the local shops**
- **Walking the children to school**
- **Walking to work**

Reduce periods of sitting to less than 30-minutes at a time

- **Golf**
- **Football**
- **Tennis**
- **Bowls**
- **Snooker**
- **Tenpin Bowling**
- **Hiking**
- **Netball**

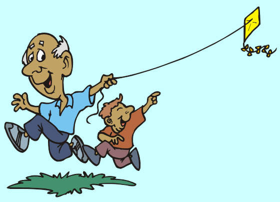

- **Brisk Walking**
- **Swimming**
- **Aerobics**
- **Cycling**
- **Rowing**
- **Bhangra**
- **Dancing**

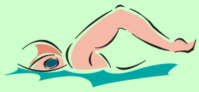

Remember, every little bit helps

Every calorie we burn makes a difference. For example, if you are only able to manage an EXTRA four, 3 minute walks each day, you would lose an extra ½ kg (1 lb) in weight each month. Over 12 months this would add up to 6 kg (12lbs).

An Example of a Walking Programme

This example is for someone who has not been physically active for a long period of time. It emphasises the importance of gradually increasing the intensity and duration of physical activity. Start off with physical activity that is within your physical ability e.g. 10 minutes of walking at a comfortable pace.

**Week 1:**	3 sessions of 10 minute walks at a comfortable pace
**Week 2:**	3 sessions of 12 minute walks at a comfortable pace
**Week 3:**	3 sessions of 14 minute walks at a comfortable pace
**Weeks 4-11:**	Build this up to 30 minutes of walking by the 11th week
**Week 12:**	Once you are up to 30 minutes of walking, start increasing your walking speed using a watch with a minute hand, walk for 5 minutes not too fast as a warm up.<br><br>Follow this by walking at a brisk pace for 5 minutes and then 5 minutes at a slower pace to allow your heart and breathing rate to return to normal. The fast walking speed should just leave you slightly out of breath.<br><br>Repeat this sequence for 30 minutes. This type of fast/slow walking will work your musculoskeletal system differently and strengthen your heart and lungs so that you can walk at a faster speed non-stop for 30 minutes.

Energy used during 30 minutes of activity

Activity		Calories burned during 30min of activity	
		For a person weighing 70kg	For a person weighing 100kg
**Sedentary activity**	• Sitting<br>• Standing	45<br>54	63<br>75
**Household or occupational activity**	• Cleaning<br>• Cooking<br>• Shopping<br>• Scrubbing<br>• Digging<br>• Painting<br>• Gardening (mowing)<br>• Gardening (raking)	132<br>96<br>132<br>231<br>309<br>165<br>228<br>114	183<br>132<br>183<br>321<br>426<br>225<br>330<br>159
**Recreational or leisure exercise**	• Walking (leisurely)<br>• Walking (briskly)<br>• Social dancing<br>• Cycling<br>• Running (11.5min/mile)<br>• Swimming (crawl)<br>• Weight training<br>• Badminton<br>• Golf<br>• Table tennis<br>• Tennis	171<br>255<br>108<br>135<br>276<br>273<br>246<br>207<br>180<br>144<br>231	234<br>353<br>150<br>189<br>399<br>375<br>342<br>285<br>249<br>201<br>321

Remember

It is not easy to burn off calories from extra food eaten.

For example a chocolate bar containing 350 calories can be eaten in the space of a few minutes. However, you would need to walk briskly for 1-1 ½ hours to burn off the calories contained in this snack. It is therefore very important to combine exercise with healthy eating to bring about weight loss.

Healthy eating

Reduce the amount of fat you eat: Fat comes in many forms such as butter, lard, ghee and all oils and contributes more calories (energy) than any other food component. Fried foods and foods with pastry contain a lot of fat. All fats contribute the same amount of calories and so will have the same effect on your body weight, however plant and vegetable fats (mono- and poly-unsaturated fats) are better for your health than animal fats (saturated fat).

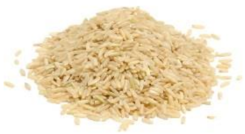

Fill up with fibre: Eating fibre can help you to feel fuller for longer and so can help with a weight reducing diet. Fibre is found in vegetables and fruit and wholegrain starchy foods such as brown rice, brown pasta, and wholemeal flours to make bread and chapatti.

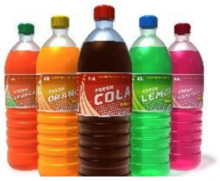

Be aware of added sugar: Try to limit the amount of added sugar you consume. Large amounts of sugar are found in many fizzy drinks and desserts. Anything that tastes sweet will probably contain sugar and should be limited in your diet.

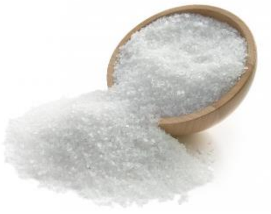

Reduce the amount of salt you eat: Although salt will not affect your body weight it will lead to an increase in your blood pressure and so can contribute to your risk of cardiovascular disease.

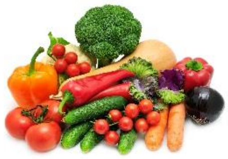

Eat more vegetables and fruit: Vegetables and fruit are low in calories, contain very little fat and salt and contain a lot of fibre. The bulk of your diet should come from vegetables and fruit.

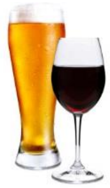

Alcohol: Alcohol contributes to your calorie intake. Ensure you know how many calories the drinks you consume contain. Beer, cider, wine and spirits mixed with sugary drinks all contain a lot of calories.

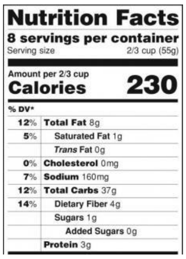

Learn to read food labels: Food labels can be really useful when you are choosing which foods to eat. They will tell you how many calories and how much fat, fibre, sugar and salt is contained within the food. Once you understand food labels you make informed choices about which foods are better for you.

Portion sizes: Don't overload your plate. Keep portion sizes sensible. Reduce the portion size of curry that contains fat and rice and chapatti and eat more fruit, salad and vegetables (with little oil).

Food Plates

The best way to reduce body weight and to increase the amount of fibre you eat is to eat more vegetables and fruit and less meat and alternatives and starchy foods. Try to make your plate look like the bottom image where over half the plate is vegetables.

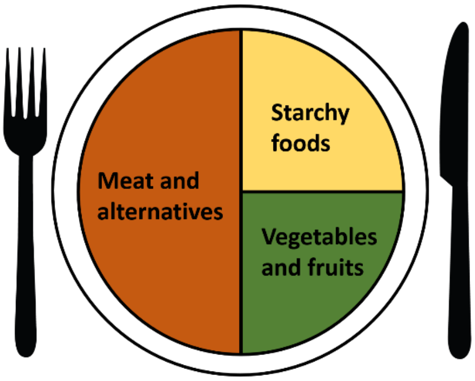

TRADITIONAL MEAL

Unhealthy meal proportions

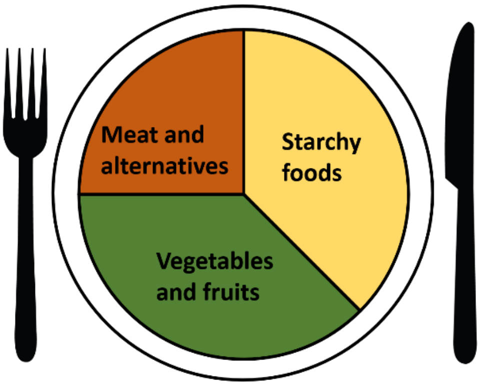

HEALTHY 'BALANCED' MEAL

Changing the proportions of food in this way leads to a healthier meal

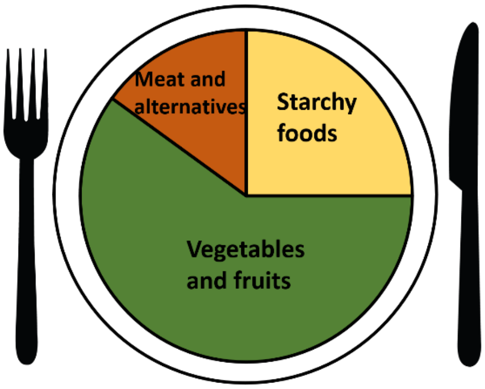

WEIGHT REDUCING MEAL

Changing the proportions in this way will reduce energy intake, help you lose weight, but allow you to eat the same amount of food

Tips:

- Cook more vegetables than you would normally prepare (cooked in no/minimal oil).
- Put vegetables on your plate first so that they take up half the plate.
- Followed by starch i.e. potatoes/rice.
- Followed by fish/meat/cheese portion last, therefore reducing your intake of these products

FOOD PLATE

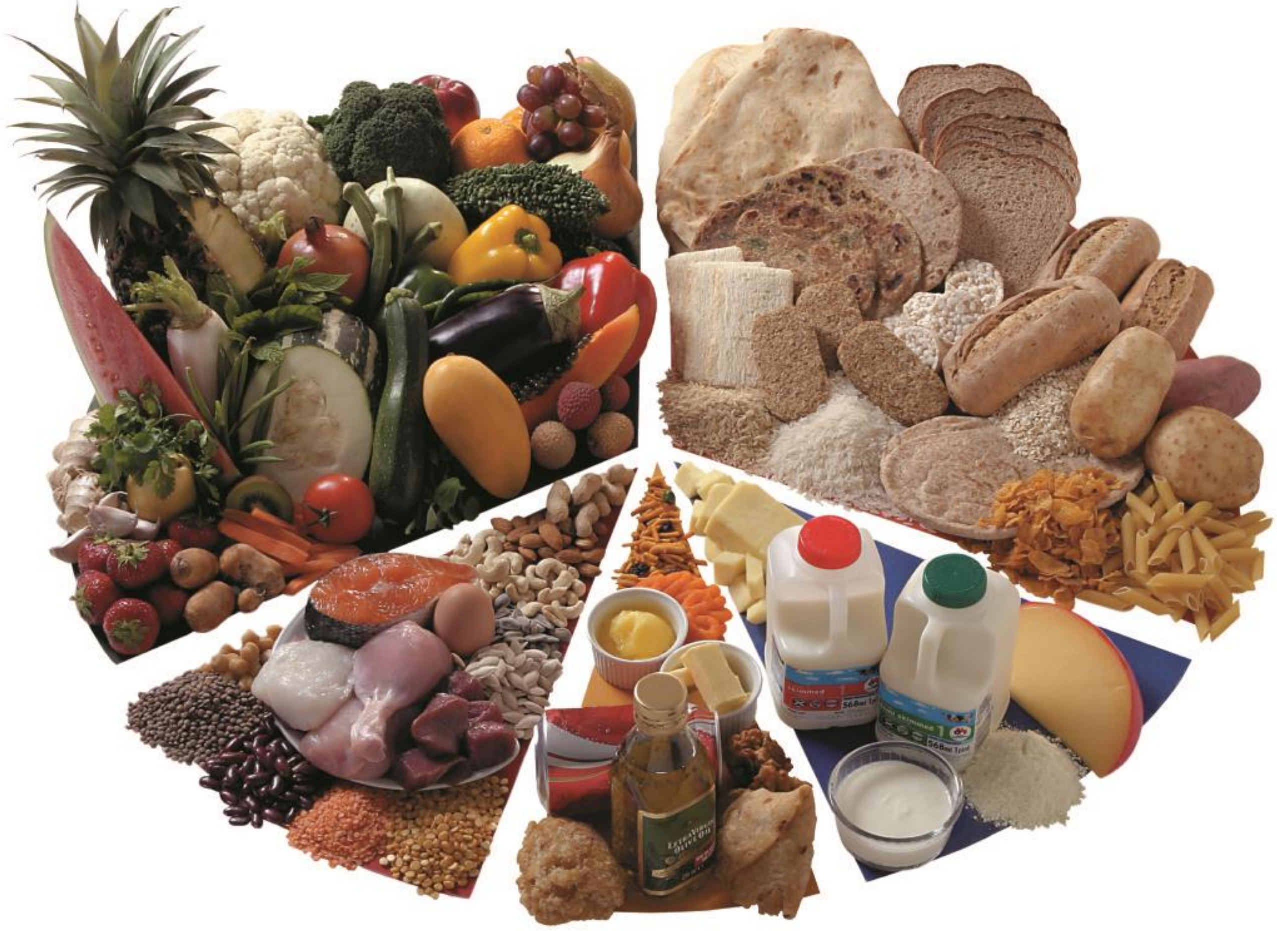

Cooking Tips

Cooking meat, poultry or fish

- Trim all fat off meat before cooking.
- Remove skin from poultry.
- Brown meat in a non-stick pan with a little oil.
- Try grilling, poaching, microwaving, baking or dry frying in a non-stick pan without oil.
- Prepare meat or chicken casseroles/soup a day ahead, refrigerate and skim off the surface fat.
- Use pearl barley, lentils, split peas and beans to thicken meat or chicken curry.

If you use oil in cooking

- Limit to 1 tsp. per person up to a maximum of 1 tbsp for a whole meal.
- Use mono or polyunsaturated oils such as olive, rapeseed, corn, soya, and sunflower oils.
- Add water or tinned tomatoes to prevent food sticking to the pan. Double the amount of onions you use for sauces to increase moisture content.
- There is no need to add oil if making meat or mince, just brown the meat and add the spices, tomatoes and onions as normal

If you use cream in cooking

- For savoury dishes, try adding low fat natural yoghurt after cooking, instead of cream during cooking.
- Try using low fat milk as a basis for a white sauce and thicken with cornflour. Flavour with herbs or a small amount of strong flavoured cheese.

If you like to grill

- Marinate foods in lemon/lime juice, tomato, soy, fish or oyster sauce.
- Seasonings such as herbs, garlic, ginger, mustard and other spices may be added before grilling to add tenderness and flavour.
- Foods cook more evenly if at room temperature before cooking.

If you like to roast

- Try dry roasting by spraying with an oil spray or use a small amount of mono or polyunsaturated
- Season meats with garlic, herbs, spices and herbs for fuller flavour.
- Cook meats in a baking dish, on a rack over water to retain moisture.
- Wrap foods and flavourings in foil try baking instead.

If you like desserts

- Try low fat ice cream, custard and other milky puddings such as kheer made with low fat milk and artificial sweetener, sugar free jelly, low fat yoghurt.
- Stew fruits using artificial sweetener and serve with natural yoghurt.

Focus on fat

Why eat low fat foods?

One gram of fat has more than twice the calories of one gram of carbohydrate. So a high fat food will almost always have more calories in it than a high carbohydrate food of a similar size or weight.

People tend to eat a constant amount of food and mainly judge food portion sizes by eye. So by choosing lower fat foods, you can eat a similar amount of food but still consume fewer calories.

High fat diets are more likely to lead to weight gain than high carbohydrate diets. Eating more starchy high fiber foods, fruits and vegetables and less fat can help with weight loss.

Eating a diet low in fat and high in starchy high fibre foods, fruits and vegetables is the best way to get the balance right.

1g fat = 9 kcal
1g carbohydrate = 4 kcal
1g protein = 4 kcal
1g alcohol = 7 kcal

Finding the fat in food

The food labels and ingredients lists on food products can help you work out how much fat there is in different food products. Have a look at the Clinic visit 5 section of this booklet called '*Confused About Food Labels?'* for more information.

Some typical examples of foods with a lot of fat in them are: pies, pastries, biscuits, cakes, crisps and savoury sauces. These are called hidden fats.

Some fats are healthier than others

All types of fat contain the same amount of calories so switching from one type to another will not help you lose weight. Only *reducing* the amount of fat you eat can do that.

However, limiting the amount of saturated fat (animal fat) you eat is better for you.

Replace saturated fats such as ghee and butter with small amounts of healthier fats such as pure vegetable oil (rapeseed), olive oil, sunflower oil, corn oil

FAT POSTER HERE

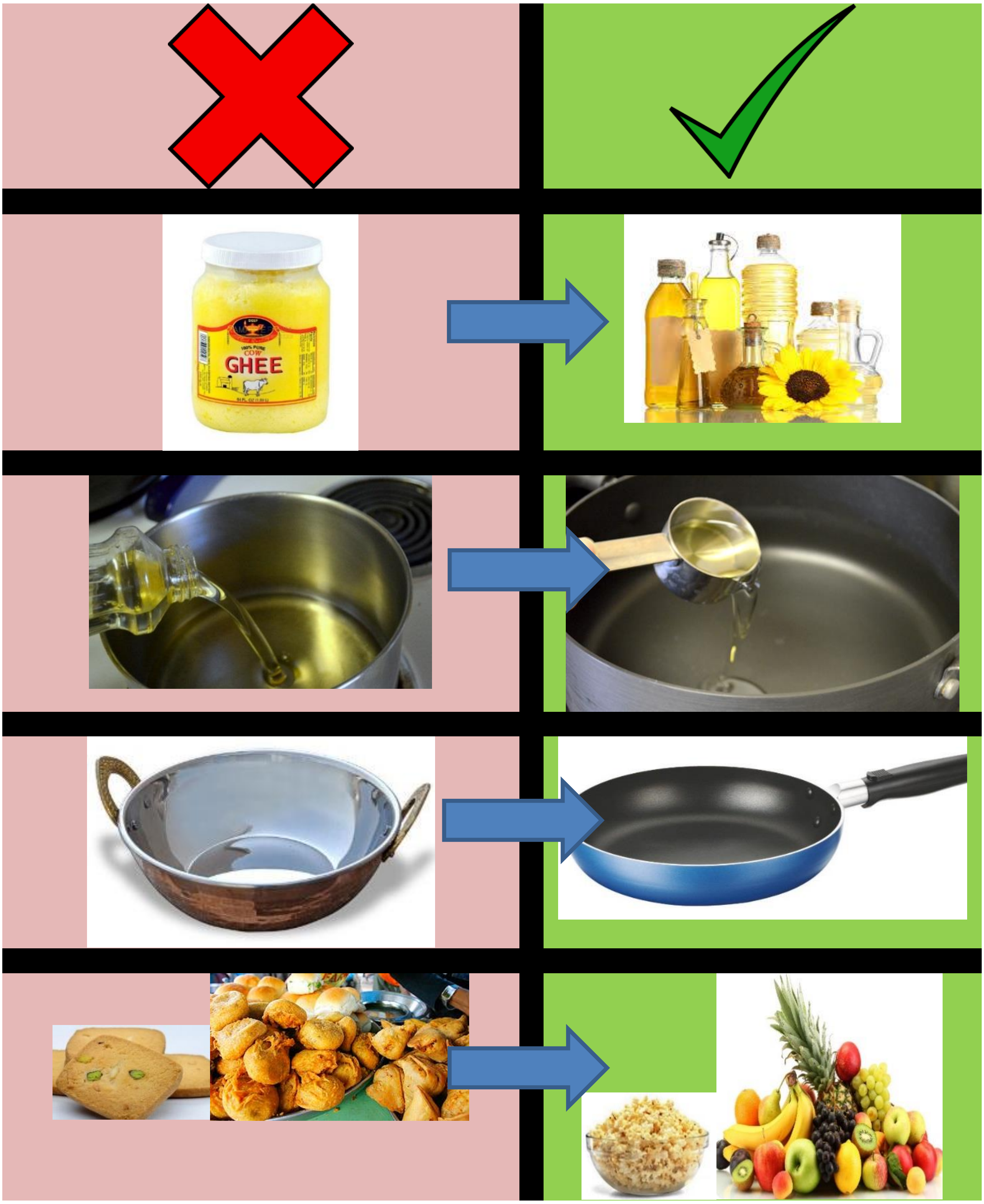

Hidden fats

Look at how much fat can be hidden in some foods. To cut down your fat, choose some of the lower fat alternatives.

If you choose this HIGH FAT product			SWITCH to this LOW FAT alternative		
**Food**	**Amount**	**Contains this amount of fat (g)**	**Food**	**Amount**	**Contains this amount of fat (g)**
**Scotch egg**	1	**65**	**Baked beans on toast**		**0**
**Sausage roll**	1 large	**60**	**Jacket potato (no spread)**<br>**+cottage cheese**	1<br>90g (3 oz)	**0**<br>**5**
**Cheddar Cheese**	60g (2 oz)	**30**	**Edam**	60g (2 oz)	**20**
**Cream Cheese**	150g (5 oz)	**120**	**Low fat soft cheese**	150g (5 oz)	**20**
**Double Cream**	150g (small ctn)	**100**	**Natural yoghurt**	150g (small ctn)	**0**
**Chocolate bar (milk)**	60g (2 oz)	**20**	**Muesli/ Crunchy bar**	1	**10**
**Salted peanuts**	60g (2 oz)	**40**	**Raisins**	60g (2 oz)	**0**
**American muffin**	1	**20**	**English muffin with reduced sugar jam**	1	**2**
**Pork chop with fat (grilled)**	150g (5 oz)	**50**	**Pork chop no fat (grilled)**	150g (5 oz)	**20**
**Fish in batter**	120g (4 oz)	**20**	**Plain Fish (grilled/baked)**	120g (4 oz)	**0**
**Sausages, pork (fried)**	2	**40**	**Fish fingers (grilled)**	4	**10**
**Egg (fried)**	1	**20**	**Egg (poached)**	1	**10**
**Chicken with skin (roasted)**	90g (3 oz)	**20**	**Chicken no skin (roasted)**	90g (3 oz)	**5**
**Chips (thin)**	Average portion (100g)	**20**	**Oven chips**	Average portion (100g)	**5**
**Luncheon meat**	60g (2 oz)	**20**	**Ham (lean)**	60g (2 oz)	**5**
**Meat pie**	Portion	**40**	**Beef & vegetable stew**	Portion	**10**
**Fried breakfast**	1 egg,<br>2 rashers bacon,<br>1 tomato,<br>1 slice fried bread	**60**	**Grilled breakfast**	1 poached egg,<br>1 grilled rasher bacon,<br>1 tomato +<br>1 slice toast	**25**

Traditional foods

Losing weight isn't about giving up your traditional dishes. It's about making small, healthy changes. Use the guide below to make your choices healthier.

	Traditional	Swap for this
**Starches**	Paratha,puri,kachori<br>Pulao,Biriyani<br>Fried potatoes<br>Potato curry	Chapatti,pitta bread,idli<br>Boiled basmati rice<br>Potatoes-prepared with minimal oil<br>Mixed vegetable curry
**Fruits**	Canned fruit in syrup<br>Tinned mango pulp<br>Dried fruit	All fresh fruits<br>Fruit salad with fresh mango pulp<br>Small amounts of dried fruits
**Vegetables**	Creamed or fried vegetables<br>Oily pickles	All vegetables cooked with minimal oil<br>More vegetables instead of potatoes in the veg curry<br>Dry pickle
**Meat and alternatives**	Dahl made with ghee<br>Paneer/Cheese<br>Butter chicken<br>Fried kebabs<br>Lamb curry<br>Goat/mutton curry<br>Fried fish	Dahl made with small amounts of unsaturated oil<br>Tofu/Low fat cheese<br>Grilled chicken tikka or tandoori chicken<br>Lean saag lamb(lean cut bulked with spinach)<br>Baked/grilled masala fish
**Dairy**	Whole milk and its products<br>Raita with boondhi<br>Kheer<br>Kulfi	Semi-skimmed/skimmed milk<br>Low fat raita with cucumber<br>Kheer made with skimmed milk and less sugar<br>Shrikhand made with low fat Greek style yoghurt
**Fats**	Butter,ghee,cream,<br>Coconut oil,coconut milk	Small amounts of pure vegetable, rapeseed, olive, sunflower, corn oil
**Snacks**	Bhel puri<br>Chevda, sev,gathia,dal moth<br>Crisps<br>Potato vada,pakoras,samosas<br>Fried moong<br>Dal vada<br>Fried papad<br>Fried salted nuts<br>Sweet or mango lassi<br>Sweet biscuits, cakes, mithai	Plain puffed rice (mamra) with spices<br>Chevda made with 1 part chevda mix and 3 parts puffed rice and spices<br>Dry roasted chick peas<br>Roasted corn on the cob<br>Boiled potato chaat<br>Samosa baked in the oven<br>Baked crisps<br>Popcorn (unsalted)<br>Microwaved or grilled papad<br>Handful of mixed unsalted nuts<br>Fresh sprouted moong beans<br>Dhokla<br>Handvo<br>Fruit –all types<br>Plain lassi made from low fat yoghurt<br>Fruit chaat<br>Oatcakes, rye based crackers with tomato salsa or cottage cheese

Tips to reduce fat

- Avoid frying foods when possible. Microwave, steam, poach, bake, boil or grill instead
- If you use oil, measure what you're adding with a spoon instead of pouring it straight from the container. Oil spray bottles enable you to use a fraction of the oil you would normally use.
- Add water or lemon juice if the onions stick to the bottom of the pan. Invest in a good quality non-stick frying pan if you make turka/vagar.
- Skim off any oil that settles on top of the cooked curry
- Use smaller portions of meat and add extra vegetables, potatoes and pulses such as peas, beans or lentils.
- Remove the skin from poultry and trim any visible fat from meat before cooking. Red meat such as lamb, beef, mutton and goat meat can be high in saturated fat, so use lean cuts only.
- Brown meat in a non-stick pan, drain excess fat and then add the turka if necessary.
- Use low fat products where possible.
- Choose a low fat spread instead of butter or margarine.
- Use semi-skimmed or skimmed milk instead of full fat and use low fat yoghurts instead of the full fat versions. (You can still make your own yoghurt using semi skimmed milk. For a creamier taste add a tablespoon of dried skimmed milk powder to every pint of milk)
- Switch to lower fat cheese and limit your intake of hard cheese as it is higher in fat. If you grate hard cheese rather than slice it you tend to put less on your plate.
- Avoid adding ghee or oil to your chapatti dough and spreading fat on cooked chapattis
- Pizzas, samosas, pakoras and other fast foods are high in fat. Only eat them occasionally as with all types of mithai and savoury Indian snacks
- Crisps, chocolate, biscuits and rich cakes are high energy and high fat foods. Try and find alternative snacks such as low fat yoghurt, crackers, rice cakes, fruit and raw vegetables.
- Avoid fish deep-fried in batter
- Use lemon juice, vinegar, herbs, spices, and tomato juice or low fat yoghurt instead of salad cream or mayonnaise
- If you are eating out try and choose foods that are not fried and that are not covered in dressing. Restaurant and convenience foods tend to be high in salt and fat
- Avoid spreads such as chocolate spread and peanut butter. Try and use jams or marmalade instead. These are a good source of energy with a low fat content.

Zero oil way of cooking curries

- Heat a non-stick heavy pan (kadhai)
- Dry roast jeera (cumin seeds) until it crackles and turns brown
- Add ground onion in the pan and keep roasting. When it starts sticking, add a small amount of water and keep stirring
- Add ginger and garlic paste (as per your recipe)
- Roast the onion, garlic, ginger until it turns brown. Do not add more water as it will dilute the taste
- Then add the required amounts of tinned tomatoes
- Stir and cook for a few minutes and add the turmeric and other spices according to taste
- Your zero oil masala is now ready!

Carbohydrates: Starchy foods, fibre and sugar

Carbohydrate containing foods give us energy which we need to get through the day. Both starch and sugar are digestible carbohydrates but starchy foods are more slowly absorbed than sugars and so they are better for us.

Most of the carbohydrate you eat should come from starchy foods.

Starchy foods are made from cereals and potato Eg. Bread, chapatti, rice, noodles, pasta and potatoes

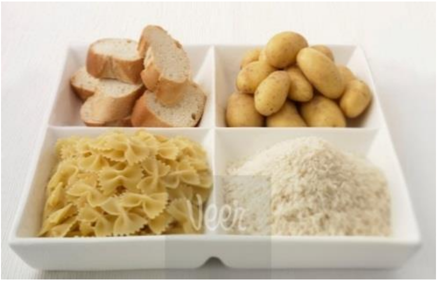

Fibre is carbohydrate that we cannot digest fully, so it travels all the way through our digestive tract intact and is good for bowel and heart health as well as adding bulk to foods and helps to make us feel full and satisfied after a meal. Fibre comes from the cell walls of plants (cereals and fruit and vegetables) but is often removed during food processing. White pasta, white rice and white bread flour have had most of the fibre removed.

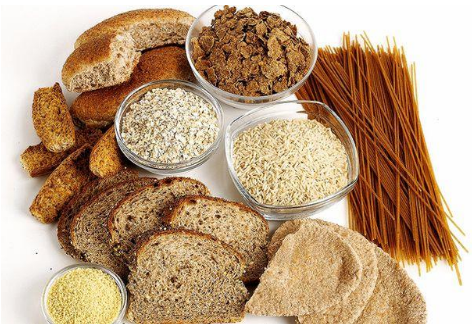

Brown/wild rice, brown/wholewheat pasta, brown/wholemeal flour and bread and oats, beans and lentils are high in fibre.

Eat these as a healthier alternatives to the 'white' starchy foods as much as possible.

Fruits and vegetables, nuts and seeds contain fibre too

- **Eat plenty of fruits and vegetables**
- **Eat small amounts of nuts and seeds (remember these are quite high in fat)**

Hidden Sugar poster here

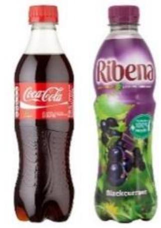<br>500ml bottle<br>Coca-cola or Ribena	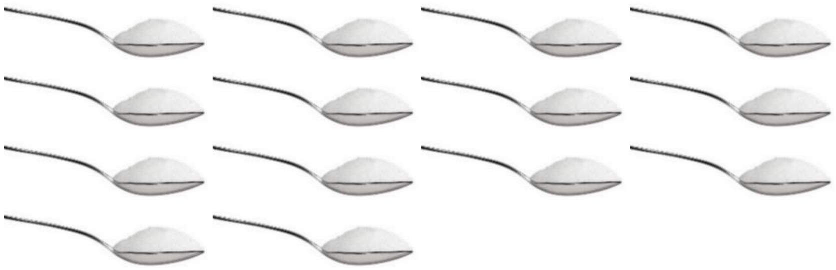
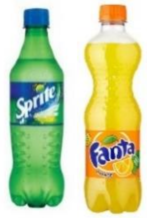<br>500ml bottle<br>Sprite or Fanta	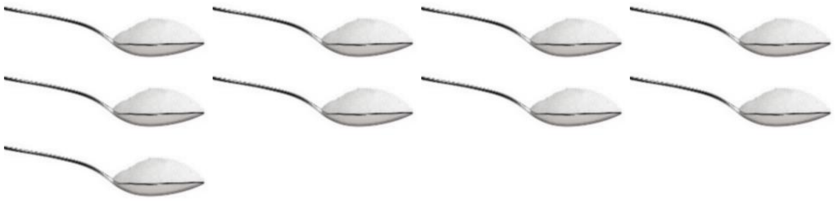
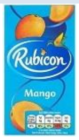<br>288ml carton<br>Rubicon Mango Juice Drink	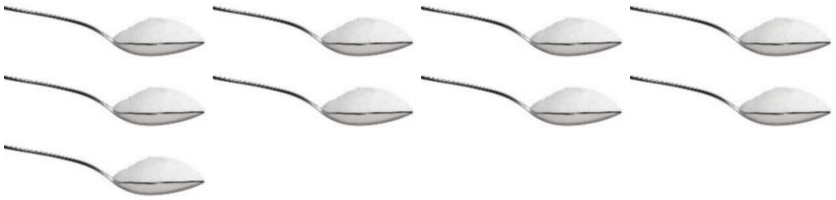
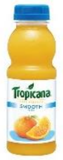<br>300ml bottle<br>Pure orange juice	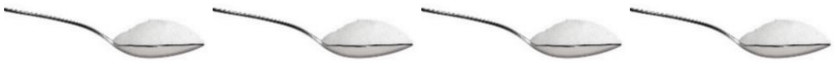
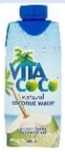<br>330ml carton<br>100% natural coconut water	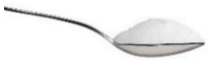
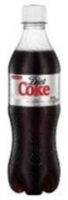<br>500ml bottle<br>Diet Coke	

Alcohol

Alcohol contains a lot of calories and increases your appetite which can encourage weight gain. If you are trying to lose weight you will need to limit the amount you drink.

For most people enjoying a small amount of alcohol regularly can be healthy but drinking too much can be harmful and increase the risk of long-term damage to your health. Many people underestimate how much alcohol they drink, it is important to know how many units you are having and keep to a healthy intake.

The UK recommendations suggest that the upper limit is 14 units/week for both men and women. However, it is very unlikely you will be able to maintain a healthy weight reducing diet if you are consuming this much alcohol.

See how many calories are in some commonly consumed drinks (1 unit measures):

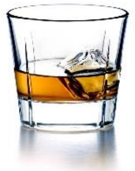

A single measure of whiskey (25ml)
55kcal

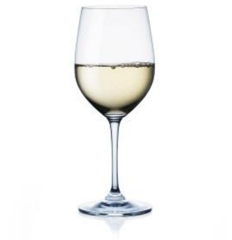

A small glass of white wine (125ml)
110kcal

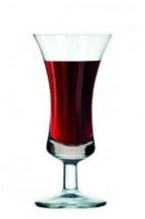

A measure of port (50ml)
80kcal

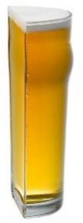

Half a pint of beer (284ml)
120kcal

Remember

- Drinks poured at home are often more generous than pub measures so don't underestimate the amount you drink.
- Alcohol is high in calories, so if you are trying to lose weight, limit your intakes and use low calorie mixer drinks.
- Benefits can come from drinking small amounts of alcohol, but drinking large amounts even occasionally can seriously damage your health.

Confused About Food Labels?

Being able to understand food labels can help you make more informed choices about packaged foods.

For a quick glance see front of pack labelling (Traffic light labelling)

Most supermarkets and food manufacturers now highlight the energy, fat, saturated fat, sugars and salt content per portion on the front of the packaging, alongside the contribution of this food (%) to the reference intake (RI) of an average adult. Be aware that the manufacturer's idea of a portion may be different from yours.

Some front-of-pack nutrition labels use red, amber and green colour coding.

- **red means high (Eat occasionally)**
- **amber means medium (Eat some of the time)**
- **green means low (Healthiest choice)**

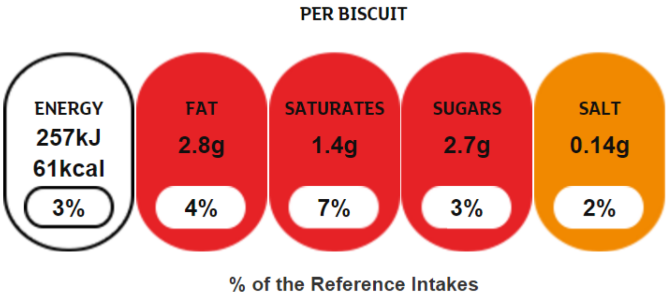

Typical Values Per 100g: Energy 2052 kJ/490 kcal

RI= Reference Intakes of an average adult (8400kJ/2000kcal)

All measures per 100g	LOW a healthier choice	MEDIUM most of the time	HIGH eat occasionally
Sugars	5g or less	5.1g - 15g	More than 15g
Fat	3g or less	3.1g - 20g	More than 20g
Saturates	1.5g or less	1.6g - 5g	More than 5g
Salt	0.3g or less	0.31g - 1.5g	More than 1.5g

What are reference intakes (RI)?

Reference Intakes (RI) have been set by European law. The values are maximum amounts that should be eaten in one day by an average adult. They are a good guide but they are not individual recommendations and your needs may be different to the RI, depending on your age, gender and how physically active you are.

For more detail see back of pack labelling (Nutrition table)

Typical Values	Per 100g	Per biscuit	% adult RI per biscuit	RI for an average adult
**Energy**	2052kJ	257kJ	-	8400kJ
	490kcal	**61kcal**	3%	2000kcal
**Fat**	23g	**2.8g**	4%	70g
**of which saturates**	11g	**1.4g**	7%	20g
**Carbohydrate**	63g	**7.8g**	3%	
**Of which sugars**	22g	**2.7g**	3%	90g
**Fibre**	3.8g	**<0.5g**	-	
**Protein**	6.8g	**0.9g**	2%	
**Salt**	1.1g	**0.14g**	2%	6g

RI= Reference Intakes of an average adult

This is telling you that each biscuit contains 61kcal

This is telling you that each biscuit contains 2.8g of fat, and that this contributes 4% of the average adult's maximum reference intake (RI)

Shopping

You may find shopping for food a difficult time. There are lots of temptations.

Choose seasonal fruits and vegetables or those on special offer if you find them expensive. Remember tinned or frozen foods can be just as healthy as fresh fruit and vegetables.

Plan for the week ahead. Write a list before you go and don't buy anything that isn't on the list.

Where possible, avoid the cakes, biscuits and snack aisles or other foods you know you will find tempting.

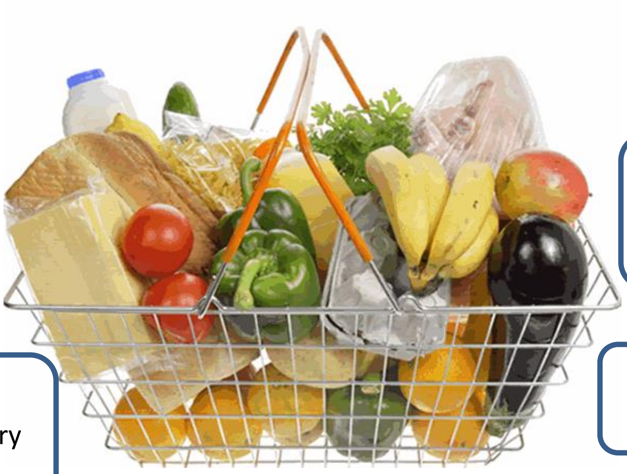

Bigger packets may not be a bargain in the long run, if they mean you eat more.

Don't shop when you are hungry, stressed, tired, angry or depressed.

Go shopping with family or a friend if you think it would help.

Holidays and special occasions

Holidays and special occasions are a time to enjoy. However if they occur regularly, or your holiday lasts a number of days and you are not mindful, you will find that a lot of the weight you have worked so hard to lose over previous months can be re-gained very quickly. This need not be the case.

Serving sizes: You may be sampling new foods. Remember to keep portion sizes small, especially if you are trying lots of different dishes. Listen to when your body is getting full, and try to leave some food on the plate.

Indulgences: Holidays and celebrations would not be same if we cannot indulge in our favourite treats. Allow yourself one per day on holiday or one per occasion, so you do not restrict your enjoyment.

Interaction: For many, the holidays and celebrations are 'food, food and more food.' Aim to shift the focus to catching up with friends and family, observing new things, meeting new people, or trying a new activity.

Exercise: Don't let the holidays be a time to abandon your exercise plan. Take advantage of your new fitness level and the extra time you have available by going on walks, swimming or playing games.

Alcohol: Remember that alcohol is high in calories and reduces your self control.

Eating out

'Eating out' includes eating at the cafeteria at work, at a restaurant or café or a takeaway. If you eat out regularly (once a week or more) the choices you make are important and will affect your ability to lose weight. If you rarely eat out (once a month or less) ENJOY your favourite foods.

Tips: ❶Choosing a starter portion as a main course means you can enjoy more courses without increasing your calories too much ❷Choose broth type soups rather than 'cream of' soups or higher fat starters ❸ Choose fruit/sorbets for dessert rather than richer cakes and puddings ❹ Choose tomato based sauces rather than cream or butter based sauces ❺ Ask for your dressing on the side so you can control how much you have ❻ At buffets or BBQs take smaller portions of high fat foods, fill up on the healthy options available ❼ Restrict the amount of alcohol you drink; it has calories and lowers your self-control.

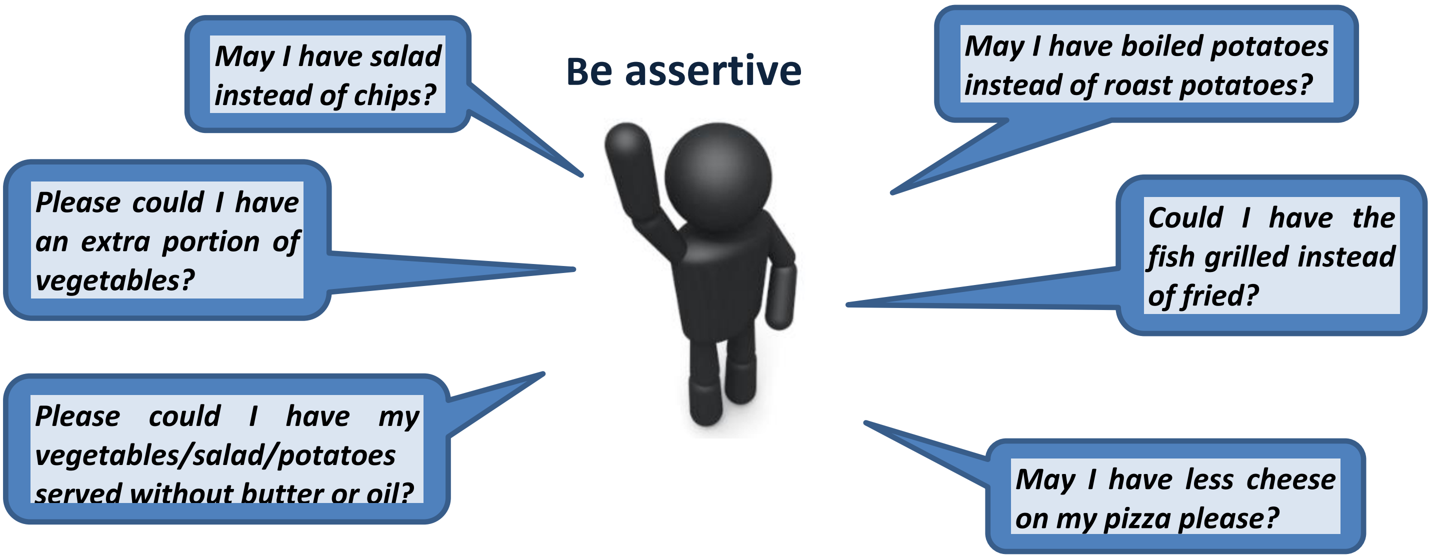

Eating out exercise:

What are your favourite takeaways and places to eat out?

How can you improve your choices?

Collect some menus from restaurants or take-away venues you use. Try and pick out the healthier options and be prepared to discuss them on your next phone call.

Dealing with pressures to eat

There will always be occasions when it may be more difficult for you to stick to your healthy eating plan. It could be holidays, parties, or even a hard day at work.

The problem solving approach

These situations are challenges and it can be helpful to take a '*problem solving approach'* to these situations.

The problem solving approach has 3 elements:

1. Stop and think about the problem
2. Look at your options
3. Resolve to respond positively and assertively

Example:

You come home from work and you find that your partner has left a plate of chocolate biscuits out in the kitchen. You feel annoyed and upset at his/her thoughtlessness. You feel tempted to eat them…

**❶ Stop and think about the problem**	When you take a step back and think, you realise you have several options
**❷ Look at your options**	You could:<br>▪ Eat the biscuits<br>▪ Throw the biscuits out<br>▪ Put the biscuits in the container<br>▪ Go and talk to your partner<br>Think carefully how each of these responses would make you feel.
**❸ Resolve to respond positively and assertively**	This means an honest and appropriate expression of your feelings while acknowledging other people's right to fairness and respect. For instance, in the example given above, you could:<br>▪ Tell your partner that it is easier for you, if he/she puts the biscuits away.<br>▪ Explain to your partner that you would find it easier to keep to the eating plan if there were no biscuits in the house and that you are going to stop buying chocolate biscuits altogether.

Pressure to eat from other people

You will come across times when other people encourage you to eat. It's a normal behaviour to share or offer food.

They may think they're being helpful, or they're uncomfortable eating alone in front of you or even that they want to test your determination.

Try to be prepared for situations where you will be offered food and have in mind how you will respond.

No thanks, I've just eaten

No thanks, I'm not hungry at the moment

Think about some of your own experiences in dealing with pressures to eat and how you have responded in the past. How might you deal with these situations now?

	How I may have responded in the past.	How I could I respond in future?
**Example 1** Tempting food at a buffet	*Starved myself all day, so felt I deserved to eat anything I liked – over ate and felt very full afterwards. I felt that I had sabotaged my diet.*	*Eat normally before going out. Survey choice of food available at buffet, go for healthier choices, and move away from the table.*
**Example 2** A friend eats in front of you		
**Example 3**		
**Example 4**		

Lapses and relapses

What is a lapse?

- A lapse is a temporary (one-off) slip back from your eating or activity plan.
- You should expect to have lapses from time to time, after all you are only human.
- One lapse is not going to undo all your hard work. What you do *following* the lapse is the most important thing.

What causes a lapse?

- Lapses do not come out of the blue; they are usually triggered by something.
- Think about the times you have not kept to your eating and activity goals.
  - Is it when you have certain feelings, like loneliness or frustration?
  - Is it when you have to deal with a particular person or event?
  - Is it when somebody offers you food?
  - Is it when you are eating with friends or when you eating alone?
- If you are aware of these 'triggers' and 'risky situations' you can do something about them.

How to recover after a lapse?

- Stop – try to avoid the situation.
- Stay Calm – don't panic. One lapse does not mean failure, say to yourself 'its not a disaster, one slip does not mean the end of the world.
- Think – what was going on before the lapse? What were the triggers? Analyse the situation using an eating or behavioural chain.
- Regain control – do something else, dispose of the problem food if any remains.
- Do not starve yourself – eat your next meal as usual. Depriving yourself can set you up to binge eat.
- Learn and Plan – what can be done in the same situation to prevent another lapse?
- Be Positive – remind yourself of how much you have achieved so far.

Remember

A lapse isn't a failure

Expect them to happen

Learn to limit how often they happen by recognising your triggers

It's what you do after a lapse that is important!

Thought patterns

All our actions begin with a thought about the action. Losing weight involves many new thoughts as we try to establish new healthier habits.

Sometimes people give themselves too hard a time about trying to lose weight and set impossible targets and deadlines. This can lead to you feeling unhappy with yourself and your abilities to achieve change of any kind. You fall into the trap of expecting too much too soon. This type of thought process is called an ‘attitude trap’.

Unhelpful Thought	Helpful Thought
**Black and white thinking:** “Either I stick to my eating plan completely or I have blown it altogether and may as well give up”	“I will try and stick to my eating plan as best I can, but expect the occasional slip”.
**Catastrophising:** “Oh no. I’ve blown it again. I had a second helping so all my hard work this week has been a waste of time”	“I have had a temporary lapse. The most important thing is that I try and get back on track, then this will not affect my overall progress”
**Over-generalisation:** “I ate that big bag of crisps. I never have any willpower”	“I ate that bag of crisps. I find them very hard to resist. Next time I will include crisps in my weekly eating plan”
**Minimising the good things:** “I’ve had a good day today, but it won’t last. I know what I am like”	“I’ve had a good day today. I am really happy with my progress”
**Magnifying the bad things:** “I ate that pudding. That’s so typical of me”	“I ate that pudding. This is my first lapse all week so overall I have done well”

Recognising your negative thought processes is the first step in learning how to change them for the better. Here are some words which are common pitfalls when trying to make goals or targets. Think about your unhelpful thoughts and how you could change them into being more helpful ones.

UNHELPFUL THOUGHT	HELPFUL THOUGHT
**I must not…**	**I will try and avoid…**
**I must…**	**My goal is to…**
**I will always…**	**I will try to…**
**I will never…**	**I will try not too…**

This slight alteration in the way you think can seem very obvious but it will really help when you look back on what you have been able to achieve.

Triggers

For many people there are particular foods or situations which cause them to lose control over their eating. It can be helpful to develop a strategy to deal with them.

Trigger Foods

Trigger foods are foods that you may find very difficult to resist. If you experience any of the following when eating a food it is probably a trigger food:

- You eat the food whenever you see it
- You find it hard to stop eating it
- You eat the food even when you are not hungry
- You eat the food instead of a meal

Coping Strategies

It may be better to try and avoid eating these foods. They can be reintroduced back into your diet once you feel that you are better able to control your eating behaviour. Some people find that cutting the food out altogether makes them crave it much more. If this happens to you it may be possible to still include it in your diet, but in a much more controlled way. For example:

- Try buying single or two-packed chocolates and biscuits and single packets of crisps.
- Before you start to eat your trigger food stop and think about how you will feel afterwards.

Trigger foods exercise:

Try to think if there are any foods you find irresistible and list them below:
What coping strategies have been discussed?

Trigger Situations

Sometimes it is not what you eat, but when and how you eat it that causes you to lose control. Try to identify your trigger situations.

Here are some common ones:

- I eat very little all day, but snack all evening.
- I often eat while watching TV.
- I often eat standing up.
- I eat while preparing food.
- I eat immediately when I come home from work.
- If I wake up at night, I eat something to help me get back to sleep.
- I often eat out with friends after work.
- I eat on the way home from the supermarket.
- If I open a packet of biscuits or chocolates I usually eat them all.
- I often eat while I walk home from the pub.

Identifying trigger situations is a big step towards coping with them.

Trigger situations exercise:

Try to think of times when you are triggered from sticking with your plans (eating or activity) and list them below:
What coping strategies have been discussed?

Eating behavior exercise

Psychologists recognise eating as a chain of events which generally starts with buying or being presented with a food and ends with eating the food. In between exists a chain of events which can best be explained through the example below:

Cathy is trying very hard to lose weight. Cathy ate half a packet of biscuits, felt guilty and then ate even more. Her eating was a chain of events with many links before and after eating. We can look at the chain that started when Cathy bought the biscuits.

There are 12 links in this chain:

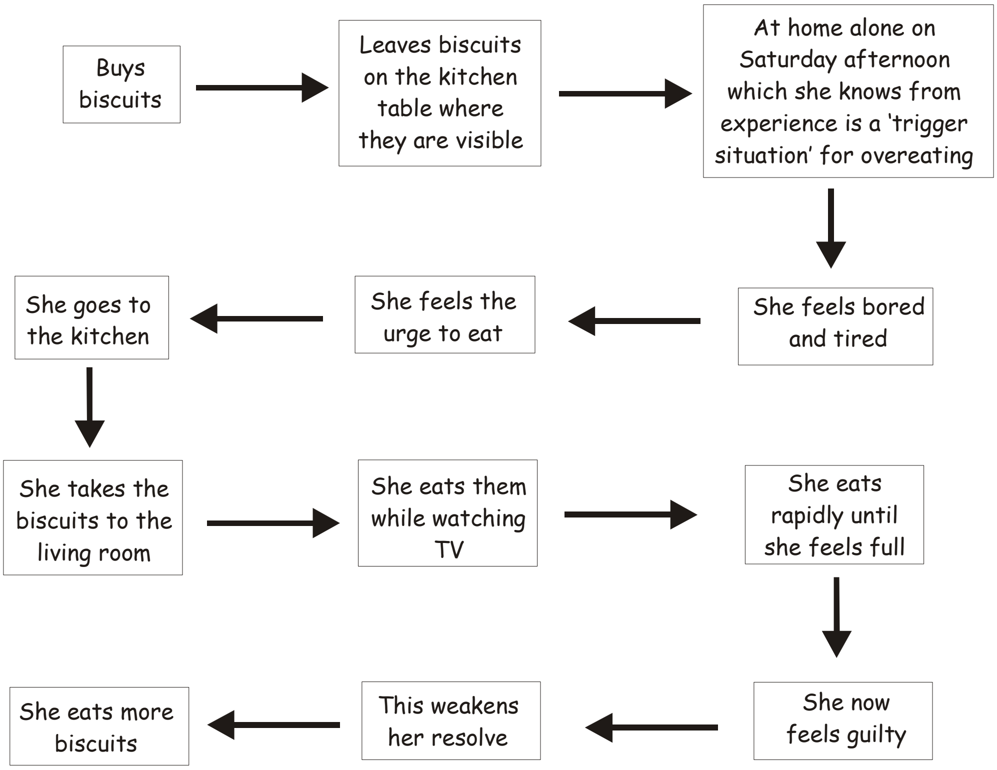

Can you see from this example there are many opportunities for Cathy to remove herself from her 'trigger situations' and her trigger food?

Here are some of the ways Cathy could have 'broken' the links in the chain which led her to eating her biscuits, and gained more control over her eating habits.

Link	Breaking the link
1. Buy biscuits	• Shop from a list and on a full stomach.<br>• Avoid the biscuit aisle.<br>• Buy some healthy alternatives to biscuits.<br>• Buy something that requires preparation.<br>• Shop with a partner or friend.
2. Biscuits on the table	• Store in opaque container.<br>• Freeze biscuits.<br>• Store out of sight and reach.
3. Home at a high risk time	• Do something you enjoy e.g. go for a walk.<br>• Get out of the house.
4. Tired and bored	• Do some exercise.<br>• Relax in a hot bath.<br>• Read a book/magazine.<br>• Household chores.
5. Urge to eat	• Learn to separate hunger from cravings.<br>• Rate your hunger on a scale of 1-10, if you rate it less then 7 then resolve not to eat.
6. Going into the kitchen	• Steer clear of the kitchen.<br>• Make sure a low calorie food is available.
7. Taking biscuits into the living room	• Resolve to eat in one room only.
8. Eating while watching TV	• Do nothing else while eating.
9. Eating rapidly until full	• Serve one biscuit at a time.<br>• Pause during eating.<br>• Chew your food well.<br>• Think about everything you eat.
10. Feeling guilty/a failure	• Think positively about achievements.<br>• Plan how to limit the damage.<br>• Review realistic goals.
11. Restraint weakens	• Resolve to increase control
12. More eating	• Examine chain.<br>• Watch attitude traps.

Think back and pick a time when you ate something that you did not really want to eat. Use the behaviour chain below to examine that situation.

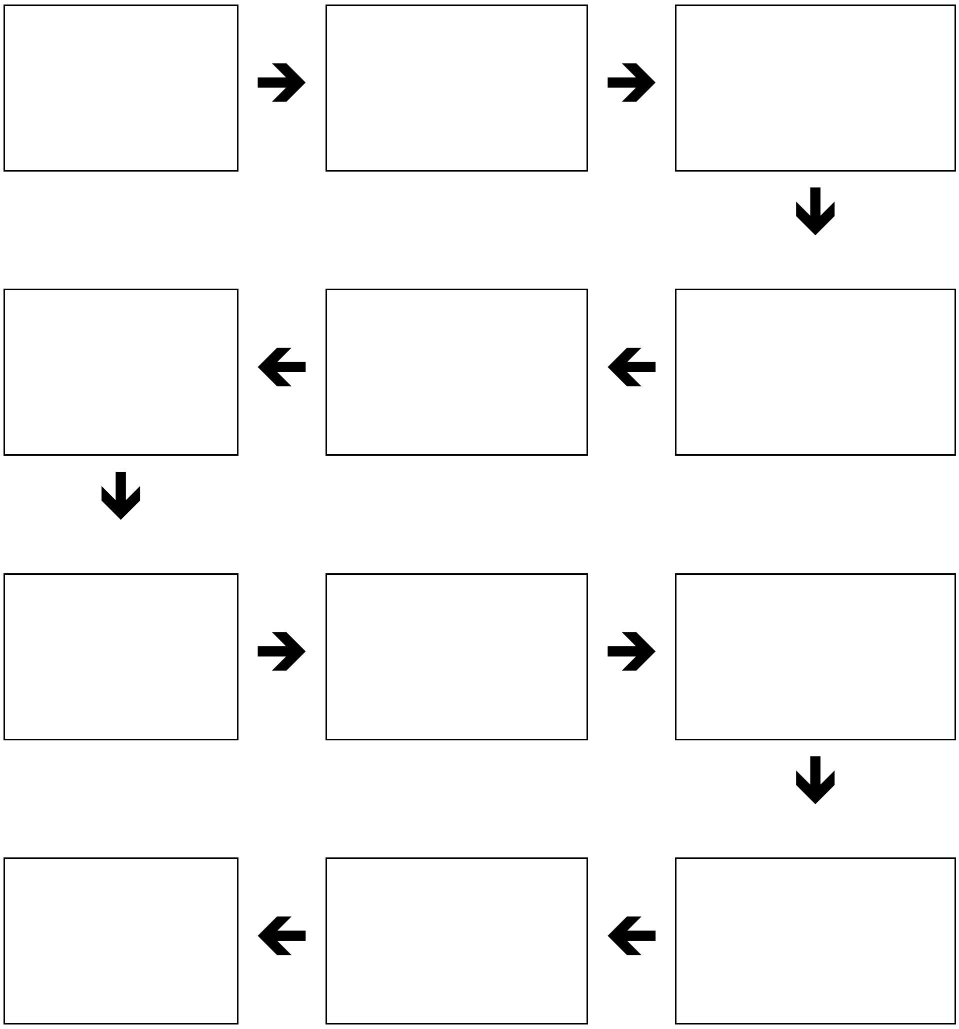

**Link**	**My strategies to breaking the link**
1.	
2.	
3.	
4.	
5.	
6.	
7.	
8.	
9.	
10.	
11.	
12.	
