# Supplementary material for: The iHealth-T2D study, prevention of type 2 diabetes amongst South Asians with central obesity and prediabetes: study protocol for a randomised controlled trial
Source: Trials. 2021 Dec 18;22:928. doi: 10.1186/s13063-021-05803-7 (PMC8684177; doi:10.1186/s13063-021-05803-7)
Supplement: Supplementary file 1 — Additional file 1: S1. CHW Handbook. Training manual summarising the content and delivery of the lifestyle intervention. Translated versions available through study website. S2. CHW Workbook. Case report form / intervention delivery workbook for the lifestyle intervention. Translated versions available through study website. S3. Participant Handbook for Lifestyle intervention. Written materials provided to the participant to support the CHW delivered lifestyle intervention. Translated versions available through study website. S4. Participant Handbook for Usual care. Translated versions available through study website. Supplementary Table 1: The a-priori power calculations under alternate assumptions with a sample size of 3,600 participants. [file 13063_2021_5803_MOESM1_ESM.zip › Addcorr2.zip!/S3 Participant booklet.zip!/21526 Clinic visit 2 V3 England_7.pdf]

i HEALTH T 2 D

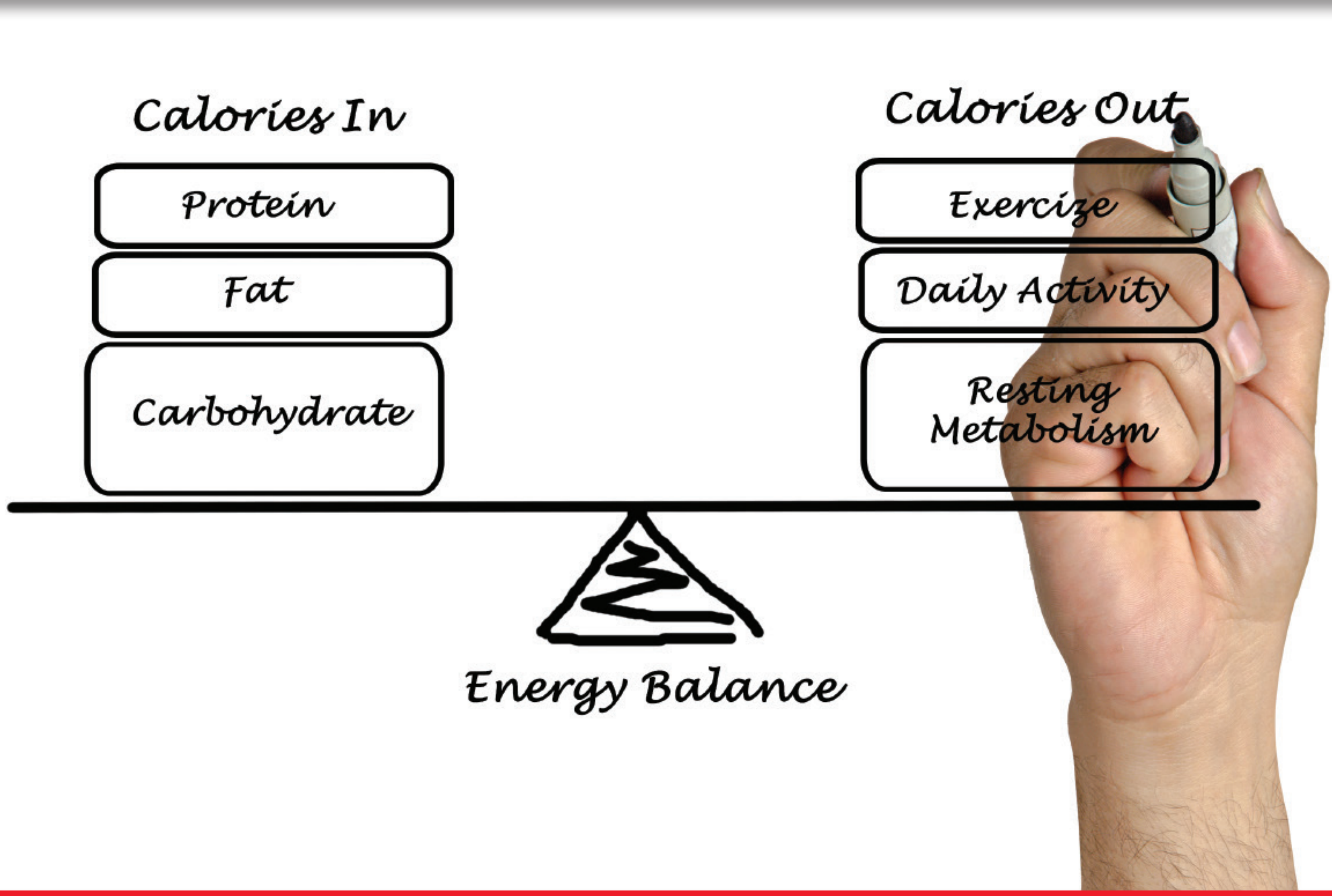

Visit 2

Energy balance

Horizon 2020
European Union Funding
for Research & Innovation

Energy balance

The only way to lose weight and keep it off is to change your eating and physical activity habits permanently. Your body weight represents the balance between all the energy (calories) taken in from the food and drink you eat, and all the energy (calories) used up during your daily activities.

To maintain your weight, the amount of energy you take in from food and drink has to equal the amount of energy you use up in your daily activities.

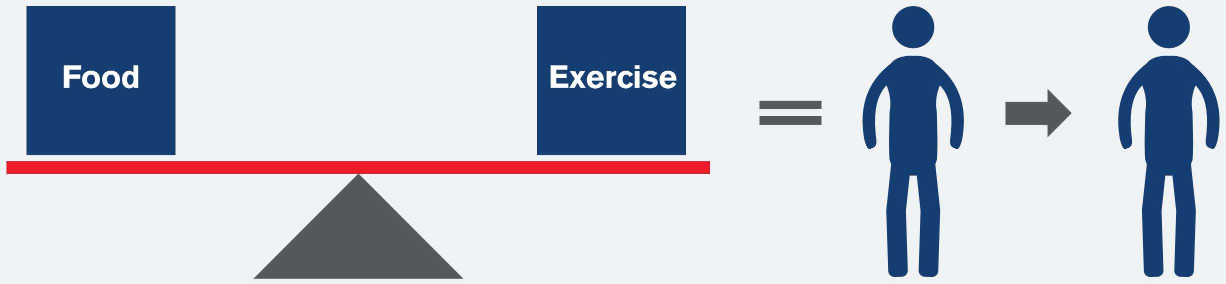

To put on weight, means that over time, the energy you have taken in has been more than the energy you have used up. The difference is usually small, typically only 50-100 extra calories daily.

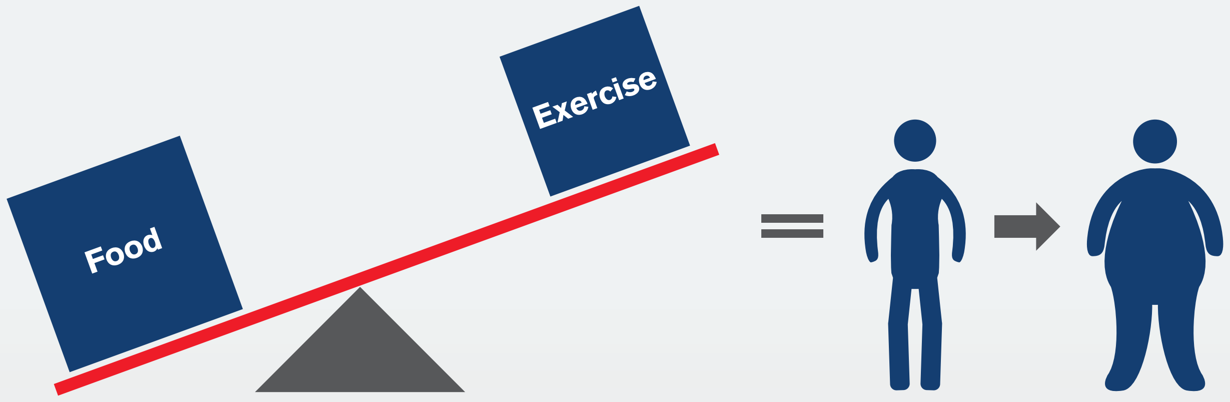

To lose weight, you need to eat fewer calories and/or do more physical activity. Your eating plan has been personally designed for you. By following this plan you should hope to lose at least ½ kg or 1lb per week.

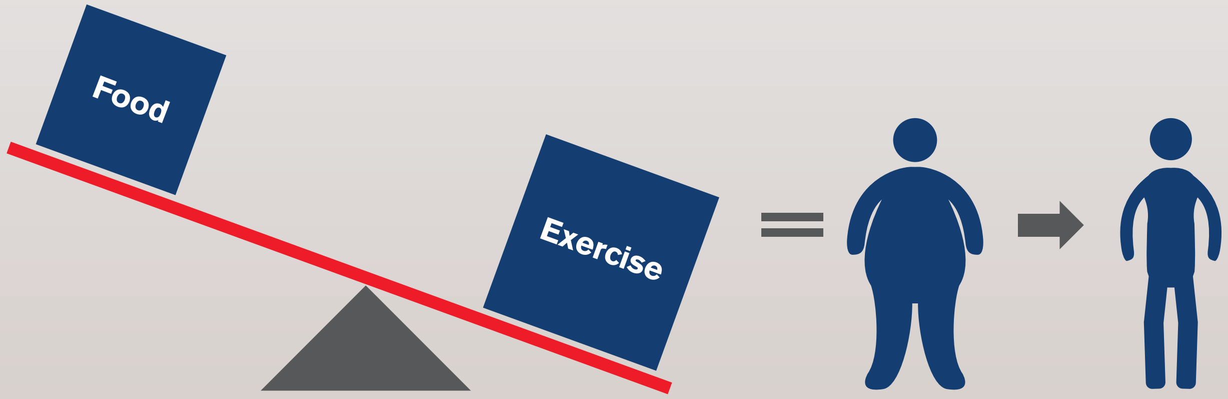

Food Plates

The best way to reduce body weight and to increase the amount of fibre you eat is to eat more vegetables and fruit and less meat and alternatives and starchy foods. Try to make your plate look like the bottom image where over half the plate is vegetables.

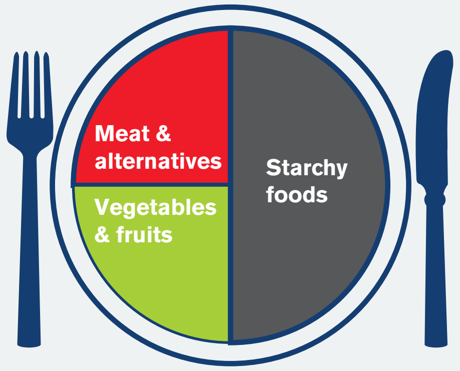

TRADITIONAL MEAL

Unhealthy meal proportions

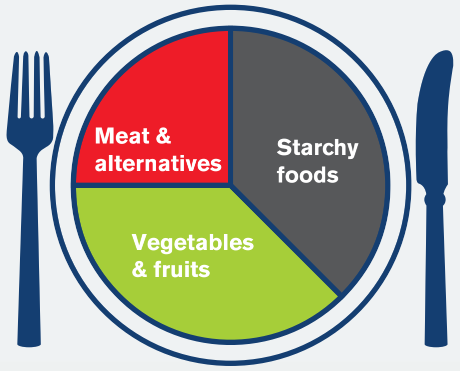

HEALTHY 'BALANCED' MEAL

Changing the proportions of food in this way leads to a healthier meal

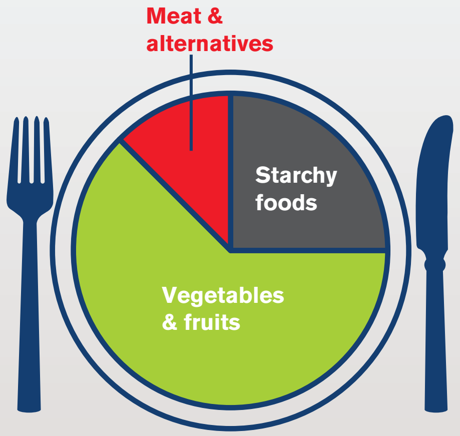

WEIGHT REDUCING MEAL

Changing the proportions in this way will reduce energy intake, help you lose weight, but allow you to eat the same amount of food

Tips

- Cook more vegetables than you would normally prepare (cooked in no/minimal oil).
- Put vegetables on your plate first so that they take up half the plate.
- Followed by starch i.e. potatoes/rice.
- Followed by fish/meat/cheese portion last, therefore reducing your intake of these products

Food plate poster

This eatwell plate shows the types and proportions of different foods people need for a healthy balanced diet.

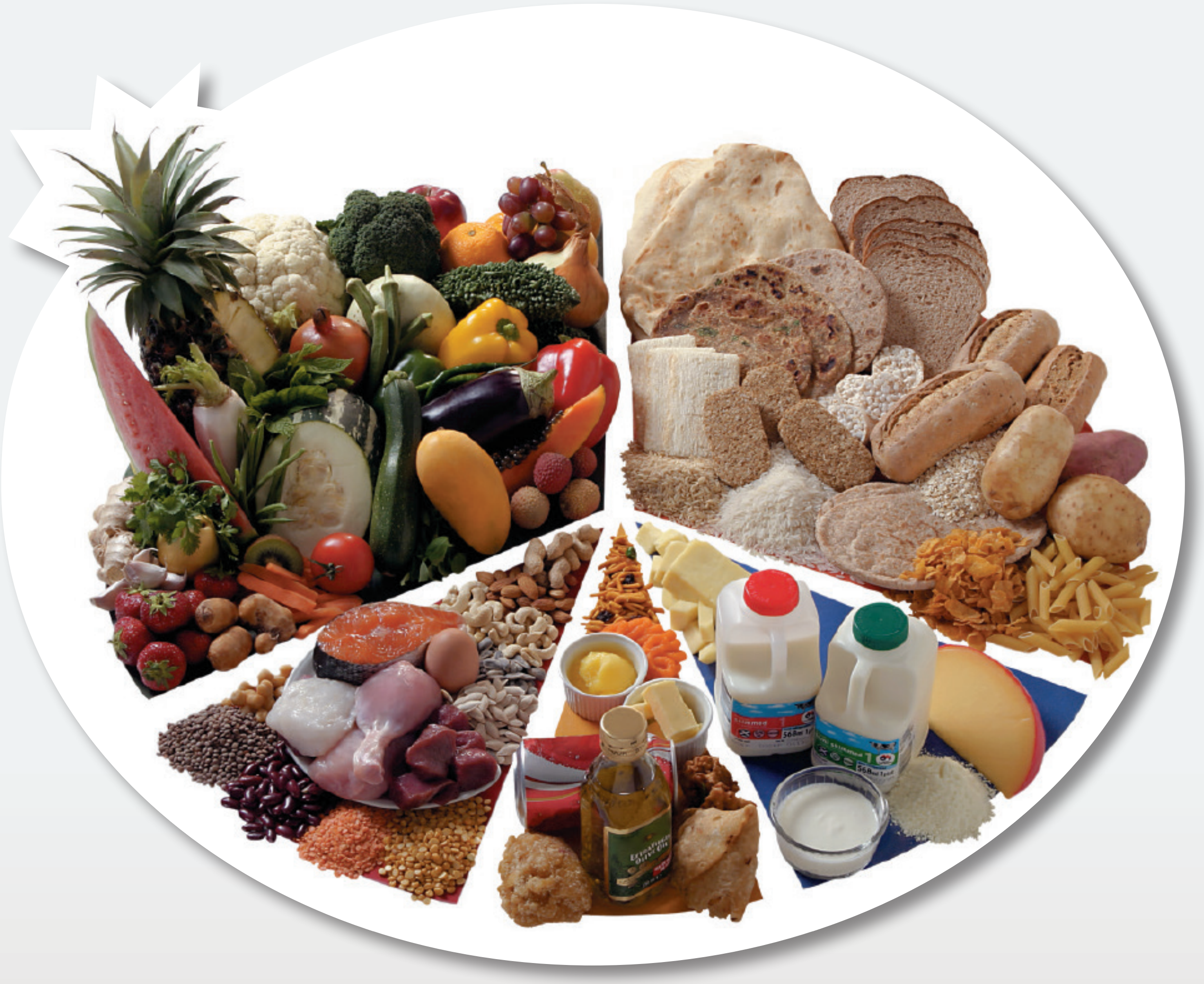

Healthy eating

In the following visits you attend you will discuss healthy eating in more detail. As an overview here are the main messages:

Reduce the amount of fat you eat

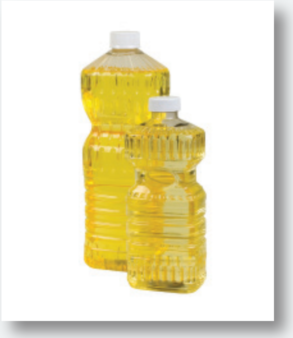

Fat comes in many forms such as butter, lard, ghee and all oils and contributes more calories (energy) than any other food component. Fried foods and foods with pastry contain a lot of fat. All fats contribute the same amount of calories and so will have the same effect on your body weight, however plant and vegetable fats (mono- and poly-unsaturated fats) are better for your health than animal fats (saturated fat).

Fill up with fibre

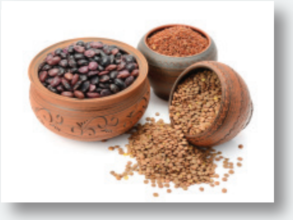

Eating fibre can help you to feel fuller for longer and so can help with a weight reducing diet. Fibre is found in vegetables and fruit and wholegrain starchy foods such as brown rice, brown pasta, and wholemeal flours to make bread and chapatti.

Be aware of added sugar

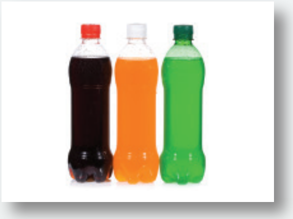

Try to limit the amount of added sugar you consume. Large amounts of sugar are found in many fizzy drinks and desserts. Anything that tastes sweet will probably contain sugar and should be limited in your diet.

Reduce the amount of salt you eat

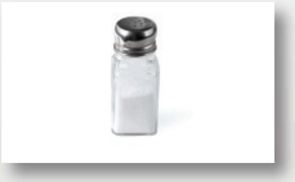

Although salt will not affect your body weight it will lead to an increase in your blood pressure and so can contribute to your risk of cardiovascular disease.

Eat more vegetables and fruit

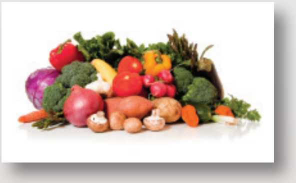

Vegetables and fruit are low in calories, contain very little fat and salt and contain a lot of fibre. The bulk of your diet should come from vegetables and fruit.

Alcohol

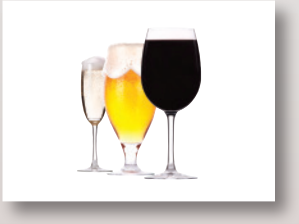

Alcohol contributes to your calorie intake. Ensure you know how many calories the drinks you consume contain. Beer, cider, wine and spirits mixed with sugary drinks all contain a lot of calories.

Learn to read food labels

Food labels can be really useful when you are choosing which foods to eat. They will tell you how many calories and how much fat, fibre, sugar and salt is contained within the food. Once you understand food labels you make informed choices about which foods are better for you.

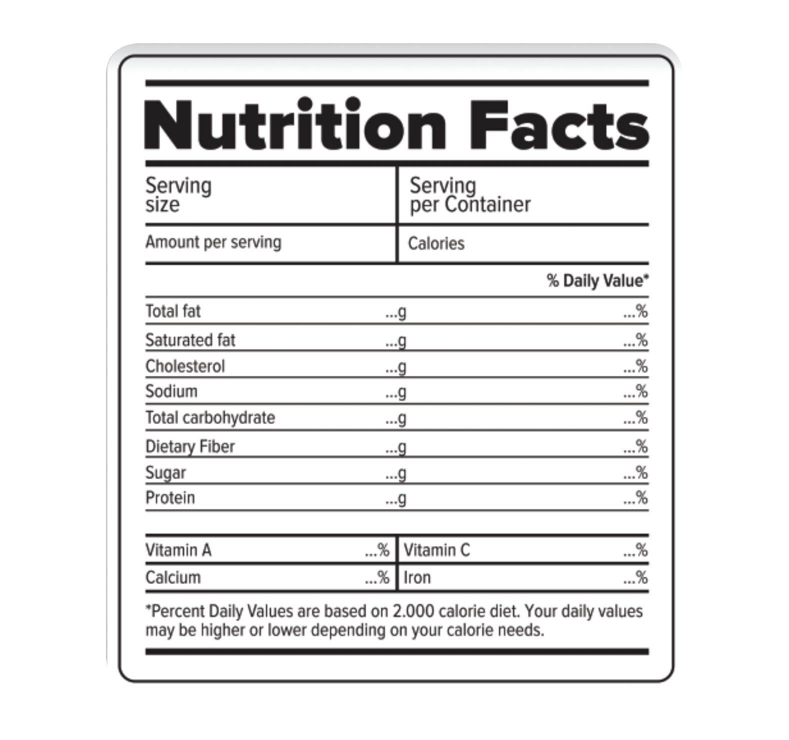

Portion sizes

Don't overload your plate. Keep portion sizes sensible. Reduce the portion size of curry that contains fat and rice and chapatti and eat more fruit, salad and vegetables (with little oil).

Cooking Tips

Cooking meat, poultry or fish

- Trim all fat off meat before cooking.
- Remove skin from poultry.
- Brown meat in a non-stick pan with a little oil.
- Try grilling, poaching, microwaving, baking or dry frying in a non-stick pan without oil.
- Prepare meat or chicken casseroles/soup a day ahead, refrigerate and skim off the surface fat.
- Use pearl barley, lentils, split peas and beans to thicken meat or chicken curry.

If you use oil in cooking

- Limit to 1 tsp. per person up to a maximum of 1 tbsp for a whole meal.
- Use mono or polyunsaturated oils such as olive, rapeseed, corn, soya, and sunflower oils.
- Add water or tinned tomatoes to prevent food sticking to the pan. Double the amount of onions you use for sauces to increase moisture content.
- There is no need to add oil if making meat or mince, just brown the meat and add the spices, tomatoes and onions as normal

If you use cream in cooking

- For savoury dishes, try adding low fat natural yoghurt after cooking, instead of cream during cooking.
- Try using low fat milk as a basis for a white sauce and thicken with cornflour. Flavour with herbs or a small amount of strong flavoured cheese.

If you like to grill

- Marinate foods in lemon/lime juice, tomato, soy, fish or oyster sauce.
- Seasonings such as herbs, garlic, ginger, mustard and other spices may be added before grilling to add tenderness and flavour.
- Foods cook more evenly if at room temperature before cooking.

If you like to roast

- Try dry roasting by spraying with an oil spray or use a small amount of mono or polyunsaturated
- Season meats with garlic, herbs, spices and herbs for fuller flavour.
- Cook meats in a baking dish, on a rack over water to retain moisture.
- Wrap foods and flavourings in foil try baking instead.

If you like desserts

- Try low fat ice cream, custard and other milky puddings such as kheer made with low fat milk and artificial sweetener, sugar free jelly, low fat yoghurt.
- Stew fruits using artificial sweetener and serve with natural yoghurt.

Alcohol

Alcohol contains a lot of calories and increases your appetite which can encourage weight gain. If you are trying to lose weight you will need to limit the amount you drink.

For most people enjoying a small amount of alcohol regularly can be healthy but drinking too much can be harmful and increase the risk of long-term damage to your health. Many people underestimate how much alcohol they drink, it is important to know how many units you are having and keep to a healthy intake.

The UK recommendations suggest that the upper limit is 14 units/week for both men and women. However, it is very unlikely you will be able to maintain a healthy weight reducing diet if you are consuming this much alcohol.

See how many calories are in some commonly consumed drinks (1 unit measures):

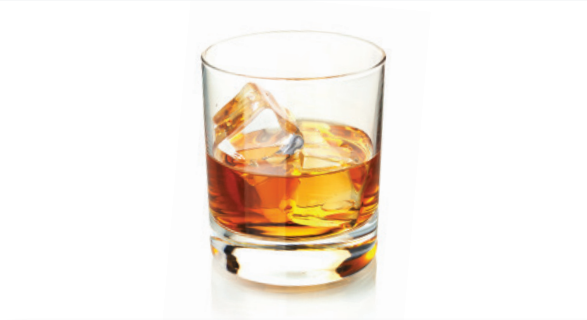

A single measure of whiskey (25ml)
55kcal

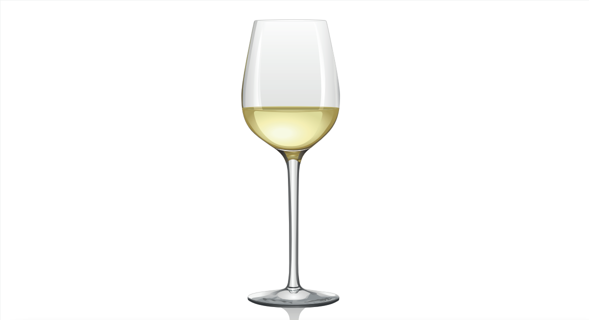

A small glass of white wine (125ml)
110kcal

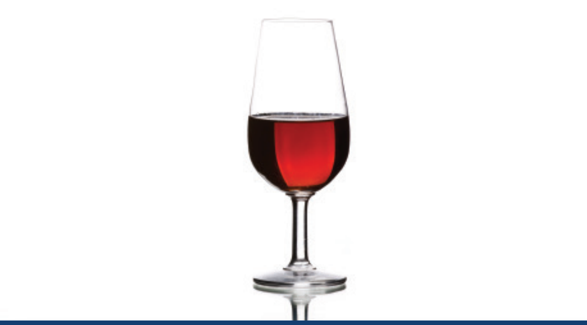

A measure of port (50ml)
80kcal

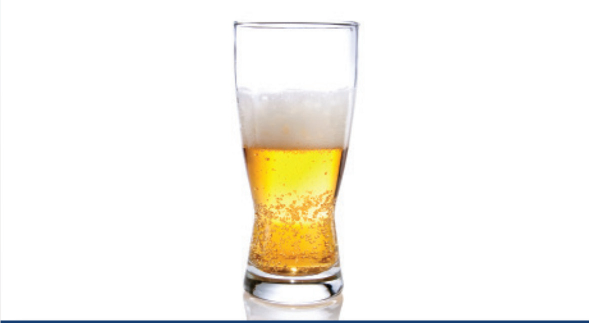

Half a pint of beer (284ml)
120kcal

Remember

- Drinks poured at home are often more generous than pub measures so don't underestimate the amount you drink.
- Alcohol is high in calories, so if you are trying to lose weight, limit your intakes and use low calorie mixer drinks.
- Benefits can come from drinking small amounts of alcohol, but drinking large amounts even occasionally can seriously damage your health.

Importance of weight loss

How **important** is it for you to lose weight in the next 6 months? (please mark from 1-10)

1	2	3	4	5	6	7	8	9	10
Not important at all		Fairly important			important				Very important

Confidence

How **confident** are you that you can lose weight to in the next 6 months? (please mark from 1-10)

1	2	3	4	5	6	7	8	9	10
Not confident at all		Fairly confident			confident				Very confident

Visit 2: Personal Target Setting

Date	Weight	Waist Circumference

Targets Set
1
2
3
4
5
6

You may find it useful to ask yourself the following questions to help you achieve your targets:

How will I go about achieving this target? How will I know if I have met this target? Is it realistic for me to achieve this target in the amount of time I have available?

Targets Met
1
2
3
4
5
6

Notes

Notes

i HEALTH T 2 D

Horizon 2020
European Union Funding
for Research & Innovation

Designed by ESP Bioscience 2016
http://www.espstc.com
