# Supplementary material for: The iHealth-T2D study, prevention of type 2 diabetes amongst South Asians with central obesity and prediabetes: study protocol for a randomised controlled trial
Source: Trials. 2021 Dec 18;22:928. doi: 10.1186/s13063-021-05803-7 (PMC8684177; doi:10.1186/s13063-021-05803-7)
Supplement: Supplementary file 1 — Additional file 1: S1. CHW Handbook. Training manual summarising the content and delivery of the lifestyle intervention. Translated versions available through study website. S2. CHW Workbook. Case report form / intervention delivery workbook for the lifestyle intervention. Translated versions available through study website. S3. Participant Handbook for Lifestyle intervention. Written materials provided to the participant to support the CHW delivered lifestyle intervention. Translated versions available through study website. S4. Participant Handbook for Usual care. Translated versions available through study website. Supplementary Table 1: The a-priori power calculations under alternate assumptions with a sample size of 3,600 participants. [file 13063_2021_5803_MOESM1_ESM.zip › Addcorr2.zip!/S3 Participant booklet.zip!/21526 Clinic visit 3 V3 England_6.pdf]

iHEALTH T2D

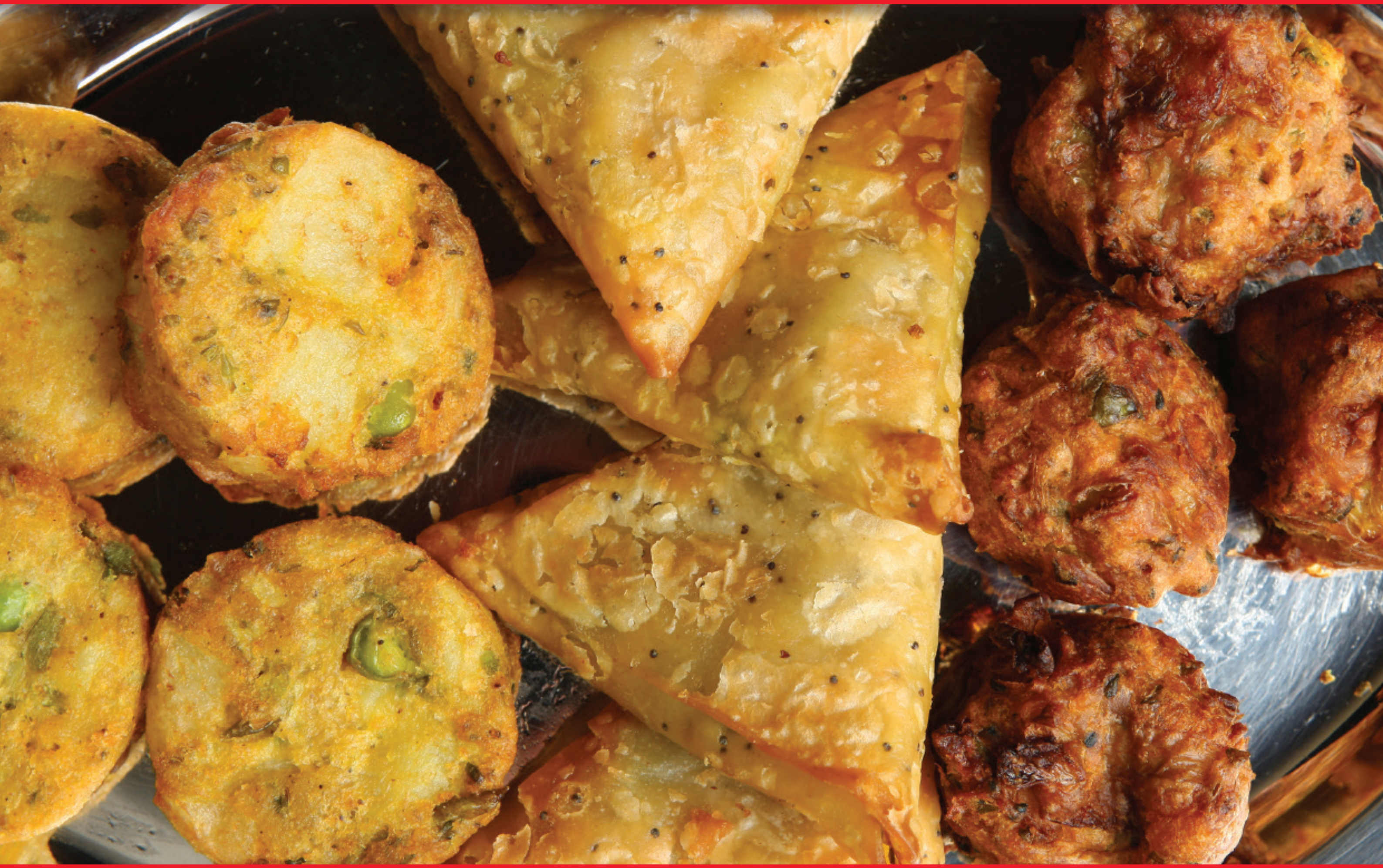

Visit 3

Focus on fat

Horizon 2020
European Union Funding
for Research & Innovation

Why eat low fat foods?

One gram of fat has more than twice the calories of one gram of carbohydrate. So a high fat food will almost always have more calories in it than a high carbohydrate food of a similar size or weight.

People tend to eat a constant amount of food and mainly judge food portion sizes by eye. So by choosing lower fat foods, you can eat a similar amount of food but still consume fewer calories.

High fat diets are more likely to lead to weight gain than high carbohydrate diets. Eating more starchy high fiber foods, fruits and vegetables and less fat can help with weight loss.

Eating a diet low in fat and high in starchy high fibre foods, fruits and vegetables is the best way to get the balance right.

1g fat = 9 kcal

1g carbohydrate = 4 kcal

1g protein = 4 kcal

1g alcohol = 7 kcal

Finding the fat in food

The food labels and ingredients lists on food products can help you work out how much fat there is in different food products. Have a look at the Clinic visit 5 section of this booklet called *'Confused About Food Labels?'* for more information.

Some typical examples of foods with a lot of fat in them are: pies, pastries, biscuits, cakes, crisps and savoury sauces. These are called hidden fats.

Some fats are healthier than others

All types of fat contain the same amount of calories so switching from one type to another will not help you lose weight. Only reducing the amount of fat you eat can do that.

However, limiting the amount of saturated fat (animal fat) you eat is better for you.

Replace saturated fats such as ghee and butter with small amounts of healthier fats such as pure vegetable oil (rapeseed), olive oil, sunflower oil, corn oil

However, limiting the amount of saturated fat (animal fat) you eat is better for you.

Fat poster

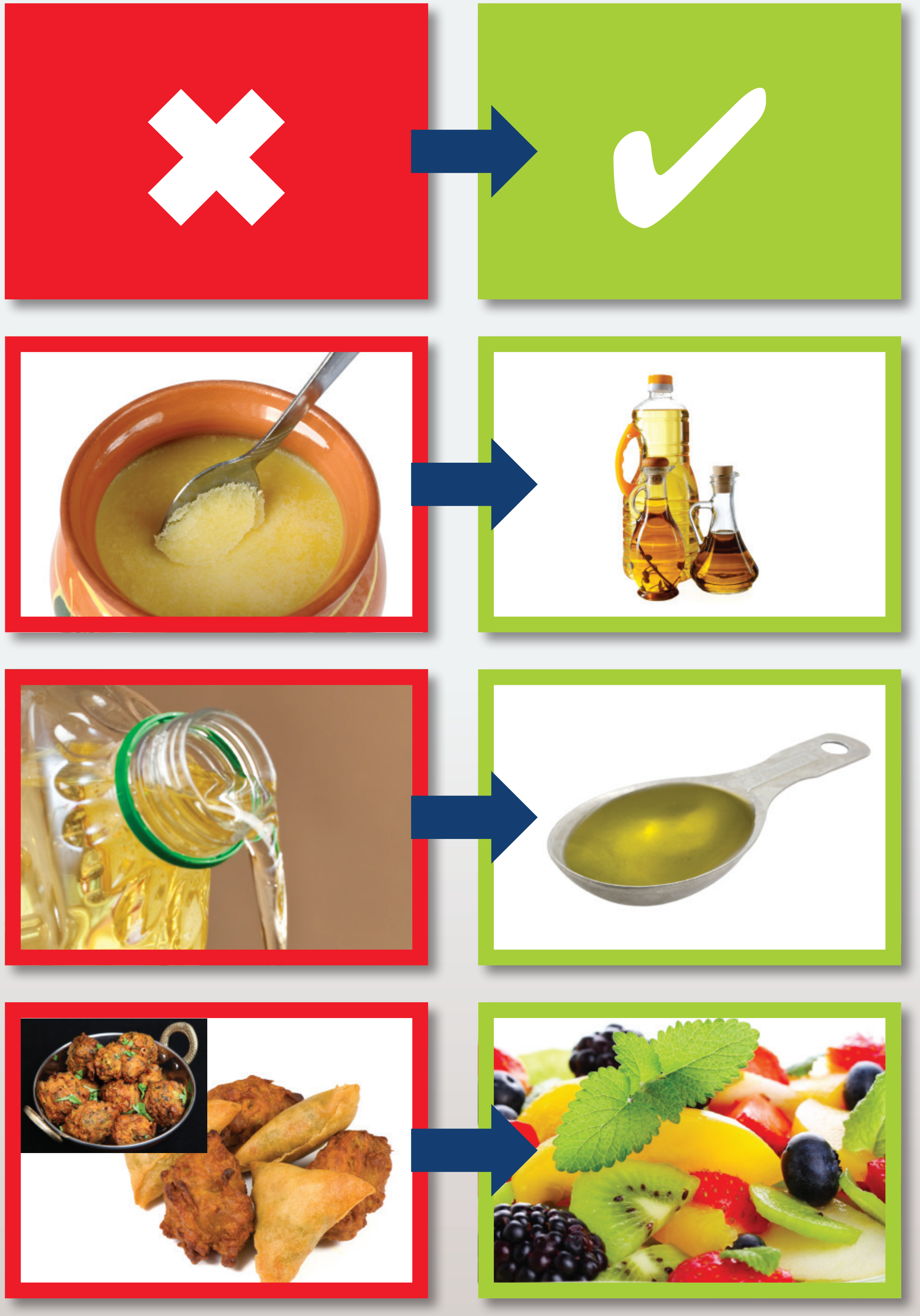

Hidden fats

Look at how much fat can be hidden in some foods. To cut down your fat, choose some of the lower fat alternatives

If you choose this HIGH FAT product			SWITCH to this LOW FAT alternative		
**Food**	**Amount**	**Contains this amount of fat (g)**	**Food**	**Amount**	**Contains this amount of fat (g)**
Scotch egg	1	65	Baked beans on toast		0
Sausage roll	1 large	60	Jacket potato (no spread) +cottage cheese	1<br>90g (3 oz)	0<br>5
Cheddar Cheese	60g (2 oz)	30	Edam	60g (2 oz)	20
Cream Cheese	150g (5 oz)	120	Low fat soft cheese	150g (5 oz)	20
Double Cream	150g (small ctn)	100	Natural yoghurt	150g (small ctn)	0
Chocolate bar (milk)	60g (2 oz)	20	Muesli/ Crunchy bar	1	10
Salted peanuts	60g (2 oz)	40	Raisins	60g (2 oz)	0
American muffin	1	20	English muffin with reduced sugar jam	1	2
Pork chop with fat (grilled)	150g (5 oz)	50	Pork chop no fat (grilled)	150g (5 oz)	20
Fish in batter	120g (4 oz)	20	Plain Fish (grilled/baked)	120g (4 oz)	0
Sausages, pork (fried)	2	40	Fish fingers (grilled)	4	10
Egg (fried)	1	20	Egg (poached)	1	10
Chicken with skin (roasted)	90g (3 oz)	20	Chicken no skin (roasted)	90g (3 oz)	5
Chips (thin)	Average portion (100g)	20	Oven chips	Average portion (100g)	5
Luncheon meat	60g (2 oz)	20	Ham (lean)	60g (2 oz)	5
Meat pie	Portion	40	Beef & vegetable stew	Portion	10
Fried breakfast	1 egg, 2 rashers bacon, 1 tomato, 1 slice fried bread	60	Grilled breakfast	1 poached egg, 1 grilled rasher bacon, 1 tomato + 1 slice toast	25

Traditional foods

Losing weight isn't about giving up your traditional dishes. It's about making small, healthy changes. Use the guide below to make your choices healthier.

	**Traditional**	**Swap for this**
**Starches**	Paratha, puri, kachori<br>Pulao, Biriyani<br>Fried potatoes<br>Potato curry	Chapatti, pitta bread, idli<br>Boiled basmati rice<br>Potatoes-prepared with minimal oil<br>Mixed vegetable curry
**Fruits**	Canned fruit in syrup<br>Tinned mango pulp<br>Dried fruit	All fresh fruits<br>Fruit salad with fresh mango pulp<br>Small amounts of dried fruits
**Vegetables**	Creamed or fried vegetables<br>Oily pickles	All vegetables cooked with minimal oil<br>More vegetables instead of potatoes in the veg curry<br>Dry pickle
**Meat and alternatives**	Dahl made with ghee<br>Paneer/Cheese<br>Butter chicken<br>Fried kebabs<br>Lamb curry<br>Goat/mutton curry<br>Fried fish	Dahl made with small amounts of unsaturated oil<br>Tofu/Low fat cheese<br>Grilled chicken tikka or tandoori chicken<br>Lean saag lamb (lean cut bulked with spinach)<br>Baked/grilled masala fish
**Dairy**	Whole milk and its products<br>Raita with boondhi<br>Kheer<br>Kulfi	Semi-skimmed/skimmed milk<br>Low fat raita with cucumber<br>Kheer made with skimmed milk and less sugar<br>Shrikhand made with low fat Greek style yoghurt
**Fats**	Butter, ghee, cream,<br>Coconut oil, coconut milk	Small amounts of pure vegetable, rapeseed, olive, sunflower, corn oil
**Snacks**	Bhel puri<br>Chevda, sev, gathia, dal moth<br>Crisps<br>Potato vada, pakoras, samosas<br>Fried moong<br>Dal vada<br>Fried papad<br>Fried salted nuts<br>Sweet or mango lassi<br>Sweet biscuits, cakes, mithai	Plain puffed rice (mamra) with spices<br>Chevda made with 1 part chevda mix and 3 parts puffed rice and spices<br>Dry roasted chick peas<br>Roasted corn on the cob<br>Boiled potato chaat<br>Samosa baked in the oven<br>Baked crisps<br>Popcorn (unsalted)<br>Microwaved or grilled papad<br>Handful of mixed unsalted nuts<br>Fresh sprouted moong beans<br>Dhokla<br>Handvo<br>Fruit – all types<br>Plain lassi made from low fat yoghurt<br>Fruit chaat<br>Oatcakes, rye based crackers with tomato salsa or cottage cheese

Tips to reduce fat

- Avoid frying foods when possible. Microwave, steam, poach, bake, boil or grill instead
- If you use oil, measure what you're adding with a spoon instead of pouring it straight from the container. Oil spray bottles enable you to use a fraction of the oil you would normally use.
- Add water or lemon juice if the onions stick to the bottom of the pan. Invest in a good quality non-stick frying pan if you make turka/vagar.
- Skim off any oil that settles on top of the cooked curry
- Use smaller portions of meat and add extra vegetables, potatoes and pulses such as peas, beans or lentils.
- Remove the skin from poultry and trim any visible fat from meat before cooking. Red meat such as lamb, beef, mutton and goat meat can be high in saturated fat, so use lean cuts only.
- Brown meat in a non-stick pan, drain excess fat and then add the turka if necessary.
- Use low fat products where possible.
- Choose a low fat spread instead of butter or margarine.
- Use semi-skimmed or skimmed milk instead of full fat and use low fat yoghurts instead of the full fat versions. (You can still make your own yoghurt using semi skimmed milk. For a creamier taste add a tablespoon of dried skimmed milk powder to every pint of milk)
- Switch to lower fat cheese and limit your intake of hard cheese as it is higher in fat. If you grate hard cheese rather than slice it you tend to put less on your plate.
- Avoid adding ghee or oil to your chapatti dough and spreading fat on cooked chapattis
- Pizzas, samosas, pakoras and other fast foods are high in fat. Only eat them occasionally as with all types of mithai and savoury Indian snacks
- Crisps, chocolate, biscuits and rich cakes are high energy and high fat foods. Try and find alternative snacks such as low fat yoghurt, crackers, rice cakes, fruit and raw vegetables.
- Avoid fish deep-fried in batter
- Use lemon juice, vinegar, herbs, spices, and tomato juice or low fat yoghurt instead of salad cream or mayonnaise
- If you are eating out try and choose foods that are not fried and that are not covered in dressing. Restaurant and convenience foods tend to be high in salt and fat
- Avoid spreads such as chocolate spread and peanut butter. Try and use jams or marmalade instead. These are a good source of energy with a low fat content.

Zero oil way of cooking curries

- Heat a non-stick heavy pan (kadhai)
- Dry roast jeera (cumin seeds) until it crackles and turns brown
- Add ground onion in the pan and keep roasting. When it starts sticking, add a small amount of water and keep stirring
- Add ginger and garlic paste (as per your recipe)
- Roast the onion, garlic, ginger until it turns brown. Do not add more water as it will dilute the taste
- Then add the required amounts of tinned tomatoes
- Stir and cook for a few minutes and add the turmeric and other spices according to taste
- Your zero oil masala is now ready!

Visit 3: Personal Target Setting

Date	Weight	Waist Circumference

Targets Set
1
2
3
4
5
6

You may find it useful to ask yourself the following questions to help you achieve your targets:

How will I go about achieving this target? How will I know if I have met this target? Is it realistic for me to achieve this target in the amount of time I have available?

Targets Met
1
2
3
4
5
6

i HEALTH T 2 D

Horizon 2020
European Union Funding
for Research & Innovation

Designed by ESP Bioscience 2016
http://www.espstc.com
iHealth-T2D: Visit 3 – UK – English – version 1
