# Supplementary material for: The iHealth-T2D study, prevention of type 2 diabetes amongst South Asians with central obesity and prediabetes: study protocol for a randomised controlled trial
Source: Trials. 2021 Dec 18;22:928. doi: 10.1186/s13063-021-05803-7 (PMC8684177; doi:10.1186/s13063-021-05803-7)
Supplement: Supplementary file 1 — Additional file 1: S1. CHW Handbook. Training manual summarising the content and delivery of the lifestyle intervention. Translated versions available through study website. S2. CHW Workbook. Case report form / intervention delivery workbook for the lifestyle intervention. Translated versions available through study website. S3. Participant Handbook for Lifestyle intervention. Written materials provided to the participant to support the CHW delivered lifestyle intervention. Translated versions available through study website. S4. Participant Handbook for Usual care. Translated versions available through study website. Supplementary Table 1: The a-priori power calculations under alternate assumptions with a sample size of 3,600 participants. [file 13063_2021_5803_MOESM1_ESM.zip › Addcorr2.zip!/S3 Participant booklet.zip!/21526 Clinic visit 4 V3 England_5.pdf]

i HEALTH T2D

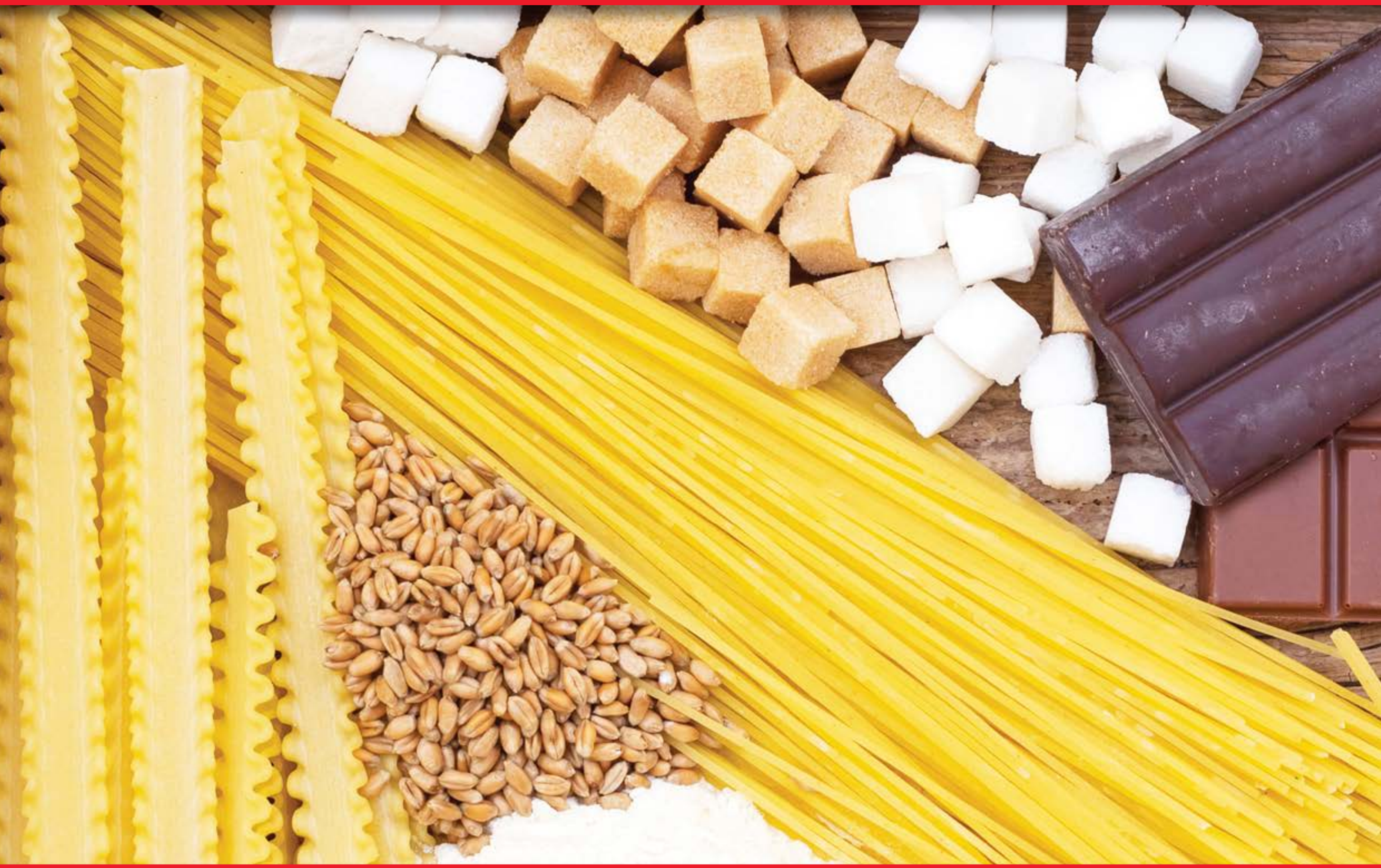

Visit 4

Carbohydrates: Starchy foods, fibre & sugar

Horizon 2020
European Union Funding
for Research & Innovation

Carbohydrates: Starchy foods, fibre and sugar

Carbohydrate containing foods give us energy which we need to get through the day. Both starch and sugar are digestible carbohydrates but starchy foods are more slowly absorbed than sugars and so they are better for us.

Most of the carbohydrate you eat should come from starchy foods.

Starchy foods are made from cereals and potato Eg. Bread, chapatti, rice, noodles, pasta and potatoes.

Fibre is carbohydrate that we cannot digest fully, so it travels all the way through our digestive tract intact and is good for bowel and heart health as well as adding bulk to foods and helps to make us feel full and satisfied after a meal. Fibre comes from the cell walls of plants (cereals and fruit and vegetables) but is often removed during food processing. White pasta, white rice and white bread flour have had most of the fibre removed.

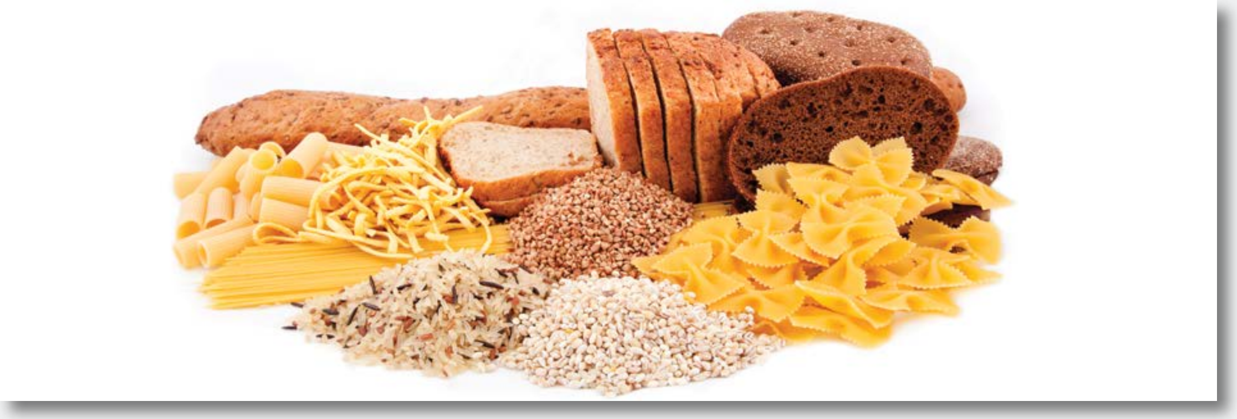

Brown/wild rice, brown/wholewheat pasta, brown/wholemeal flour and bread and oats, beans and lentils are high in fibre.

Eat these as a healthier alternatives to the 'white' starchy foods as much as possible.

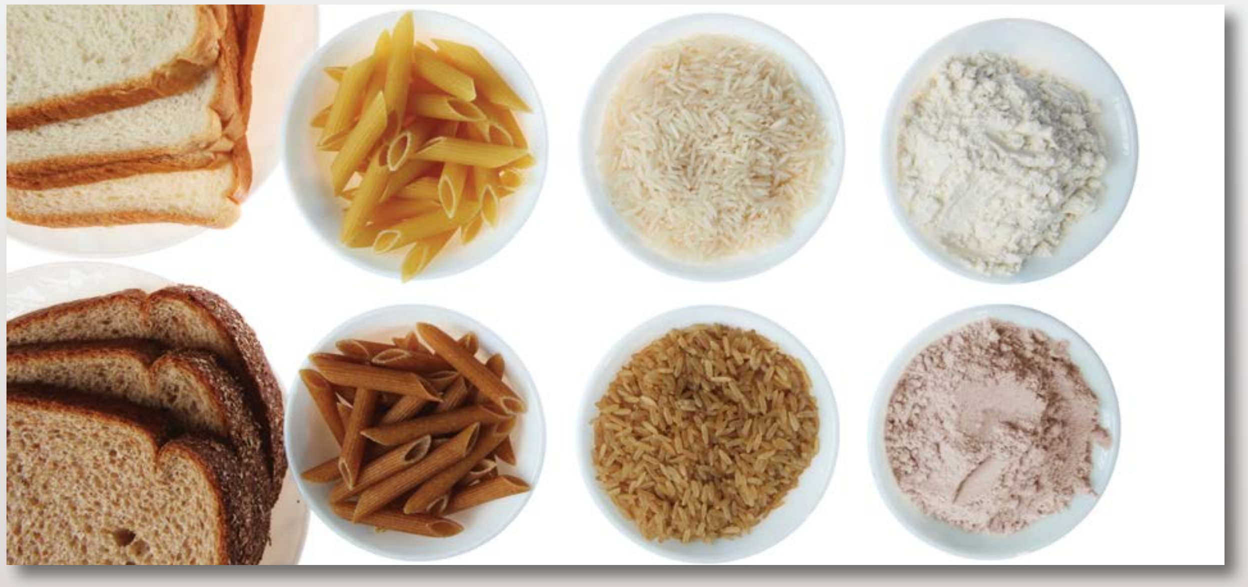

Fruits and vegetables, nuts and seeds contain fibre too

- Eat plenty of fruits and vegetables
- Eat small amounts of nuts and seeds (remember these are quite high in fat)

Hidden Sugars

500ml bottle Coca-cola or Ribena

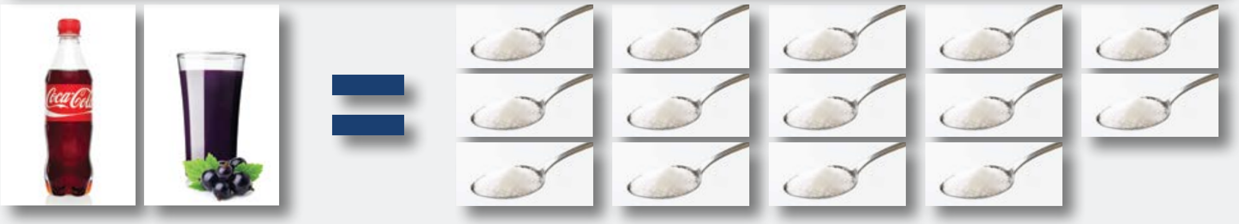

500ml bottle Sprite or Fanta

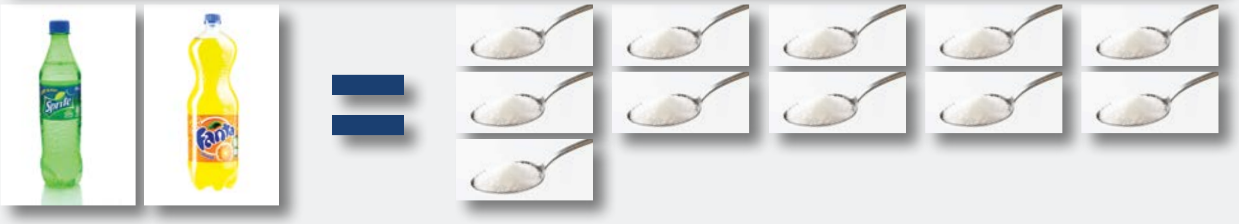

288ml carton Rubicon Mango Juice Drink

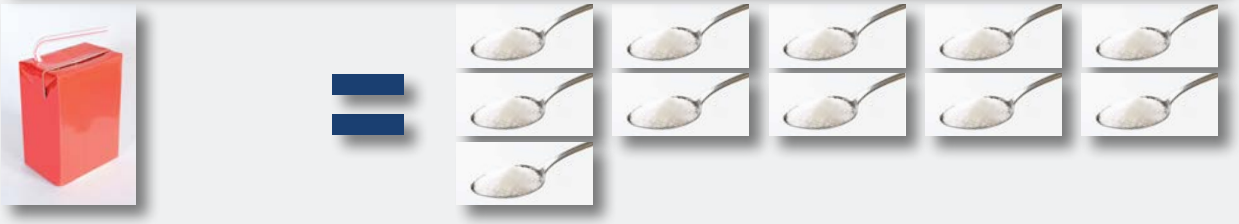

300ml bottle Pure orange juice

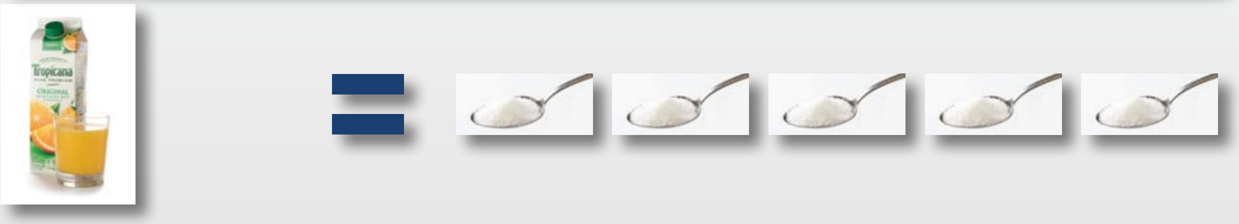

330ml carton 100% natural coconut water

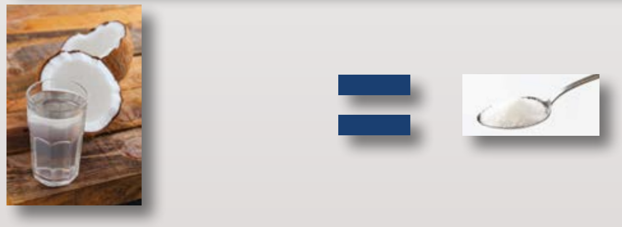

500ml bottle Diet Coke

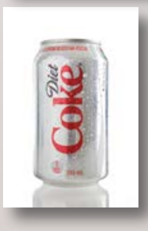

Triggers

For many people there are particular foods or situations which cause them to lose control over their eating. It can be helpful to develop a strategy to deal with them.

Trigger Foods

Trigger foods are foods that you may find very difficult to resist. If you experience any of the following when eating a food it is probably a trigger food:

- You eat the food whenever you see it
- You find it hard to stop eating it
- You eat the food even when you are not hungry
- You eat the food instead of a meal

Coping Strategies

It may be better to try and avoid eating these foods. They can be reintroduced back into your diet once you feel that you are better able to control your eating behaviour. Some people find that cutting the food out altogether makes them crave it much more. If this happens to you it may be possible to still include it in your diet, but in a much more controlled way. For example:

- Try buying single or two-packed chocolates and biscuits and single packets of crisps.
- Before you start to eat your trigger food stop and think about how you will feel afterwards.

Trigger foods exercise:

Try to think if there are any foods you find irresistible and list them below:

What coping strategies have been discussed?

Trigger Situations

Sometimes it is not what you eat, but when and how you eat it that causes you to lose control. Try to identify your trigger situations.

Here are some common ones:

- I eat very little all day, but snack all evening.
- I often eat while watching TV.
- I often eat standing up.
- I eat while preparing food.
- I eat immediately when I come home from work.
- If I wake up at night, I eat something to help me get back to sleep.
- I often eat out with friends after work.
- I eat on the way home from the supermarket.
- If I open a packet of biscuits or chocolates I usually eat them all.
- I often eat while I walk home from the pub.

Identifying trigger situations is a big step towards coping with them.

Trigger situations exercise:

Try to think of times when you are triggered from sticking with your plans (eating or activity) and list them below

What coping strategies have been discussed?

Eating behavior exercise

Psychologists recognise eating as a chain of events which generally starts with buying or being presented with a food and ends with eating the food. In between exists a chain of events which can best be explained through the example below:

Poonam is trying very hard to lose weight. Poonam ate half a packet of biscuits, felt guilty and then ate even more. Her eating was a chain of events with many links before and after eating. We can look at the chain that started when Poonam bought the biscuits.

There are 12 links in this chain:

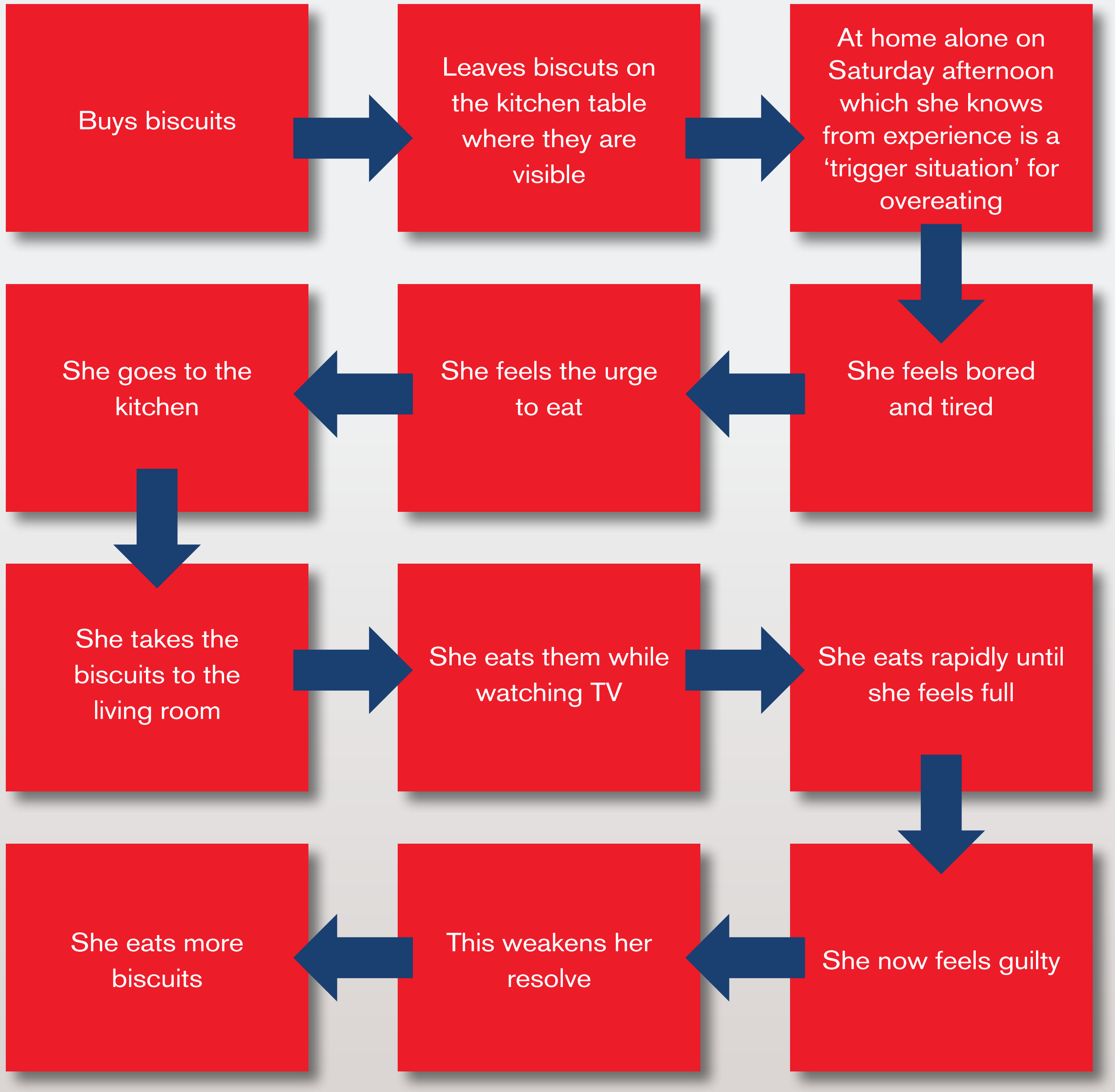

Can you see from this example there are many opportunities for Poonam to remove herself from her 'trigger situations' and her trigger food?

Here are some of the ways Poonam could have 'broken' the links in the chain which led her to eating her biscuits, and gained more control over her eating habits.

Link	Breaking the link
**1. Buy biscuits**	• Shop from a list and on a full stomach.<br>• Avoid the biscuit aisle.<br>• Buy some healthy alternatives to biscuits.<br>• Buy something that requires preparation.<br>• Shop with a partner or friend.
**2. Biscuits on the table**	• Store in opaque container.<br>• Freeze biscuits.<br>• Store out of sight and reach.
**3. Home at a high risk time**	• Do something you enjoy e.g. go for a walk.<br>• Get out of the house.
**4. Tired and bored**	• Do some exercise.<br>• Relax in a hot bath.<br>• Read a book/magazine.<br>• Household chores.
**5. Urge to eat**	• Learn to separate hunger from cravings.<br>• Rate your hunger on a scale of 1-10, if you rate it less than 7 then resolve not to eat.
**6. Going into the kitchen**	• Steer clear of the kitchen.<br>• Make sure a low calorie food is available.
**7. Taking biscuits into the living room**	• Resolve to eat in one room only.
**8. Eating while watching TV**	• Do nothing else while eating.
**9. Eating rapidly until full**	• Serve one biscuit at a time.<br>• Pause during eating.<br>• Chew your food well.<br>• Think about everything you eat.
**10. Feeling guilty/a failure**	• Think positively about achievements.<br>• Plan how to limit the damage.<br>• Review realistic goals.
**11. Restraint weakens**	• Resolve to increase control
**12. More eating**	• Examine chain.<br>• Watch attitude traps.

Think back and pick a time when you ate something that you did not really want to eat. Use the behaviour chain below to examine that situation.

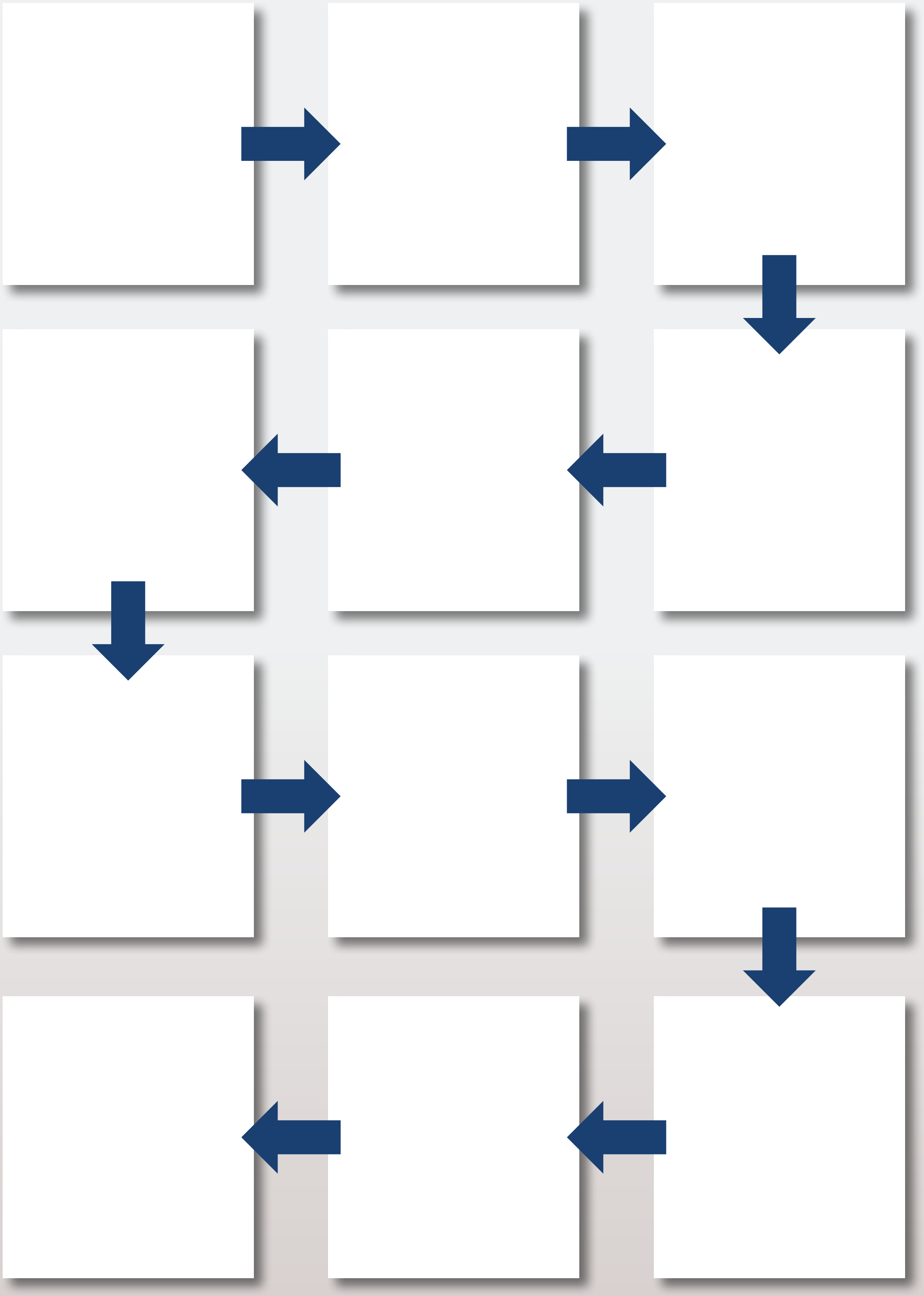

Link	My strategies to breaking the link
1.	
2.	
3.	
4.	
5.	
6.	
7.	
8.	
9.	
10.	
11.	
12.	

Visit 4: Personal Target Setting

Date	Weight	Waist Circumference

You may find it useful to ask yourself the following questions to help you achieve your targets:

How will I go about achieving this target? How will I know if I have met this target? Is it realistic for me to achieve this target in the amount of time I have available?

Targets Set
1
2
3
4
5
6

Targets Met
1
2
3
4
5
6

Notes

Notes

i HEALTH
T 2 D

Horizon 2020
European Union Funding
for Research & Innovation

Designed by ESP Bioscience 2016
http://www.espstc.com
