# Supplementary material for: The iHealth-T2D study, prevention of type 2 diabetes amongst South Asians with central obesity and prediabetes: study protocol for a randomised controlled trial
Source: Trials. 2021 Dec 18;22:928. doi: 10.1186/s13063-021-05803-7 (PMC8684177; doi:10.1186/s13063-021-05803-7)
Supplement: Supplementary file 1 — Additional file 1: S1. CHW Handbook. Training manual summarising the content and delivery of the lifestyle intervention. Translated versions available through study website. S2. CHW Workbook. Case report form / intervention delivery workbook for the lifestyle intervention. Translated versions available through study website. S3. Participant Handbook for Lifestyle intervention. Written materials provided to the participant to support the CHW delivered lifestyle intervention. Translated versions available through study website. S4. Participant Handbook for Usual care. Translated versions available through study website. Supplementary Table 1: The a-priori power calculations under alternate assumptions with a sample size of 3,600 participants. [file 13063_2021_5803_MOESM1_ESM.zip › Addcorr2.zip!/S3 Participant booklet.zip!/21526 Clinic visit 5 V3 England_4.pdf]

i HEALTH T 2 D

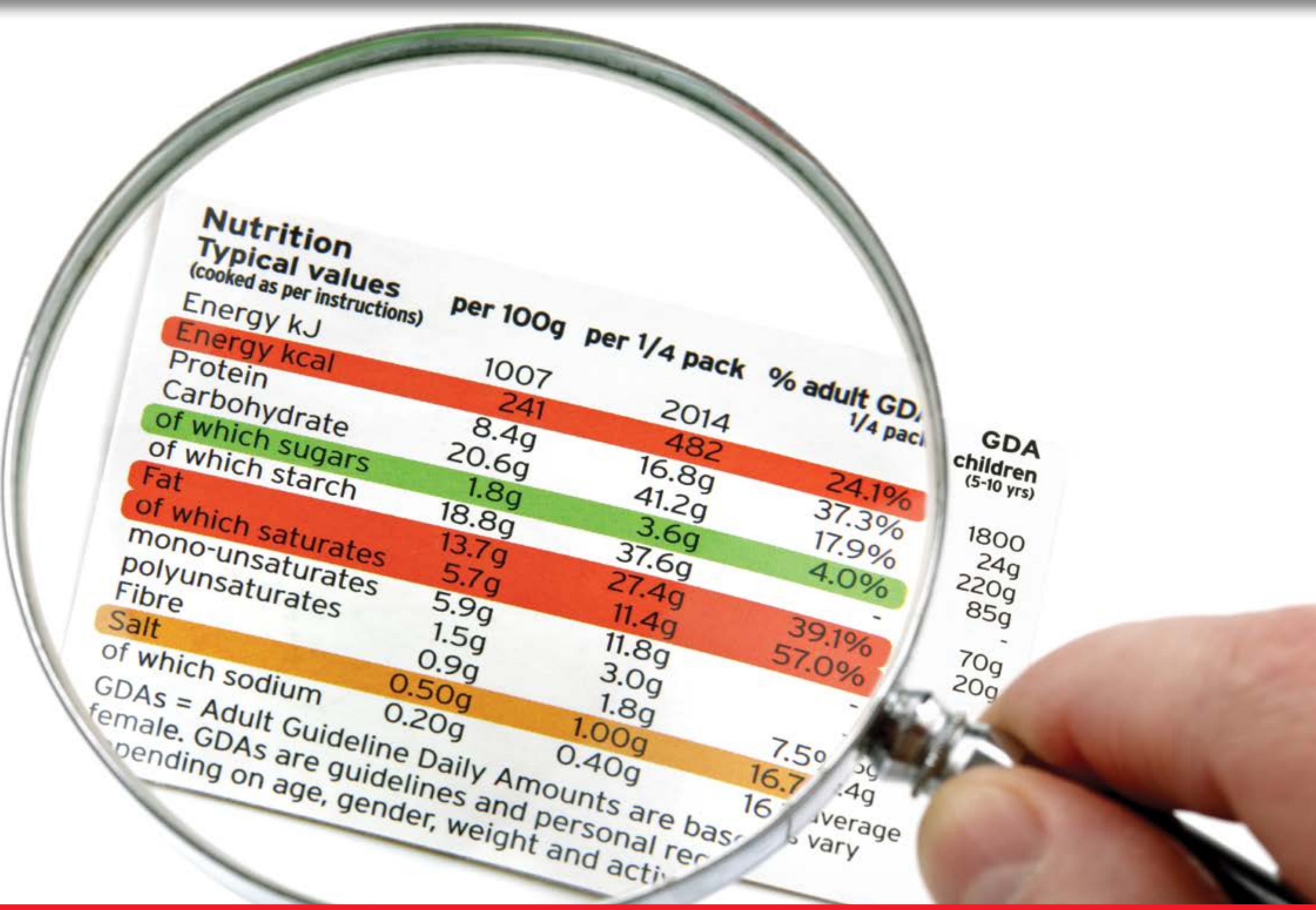


# Visit 5

## Confused About Food Labels?

Horizon 2020
European Union Funding
for Research & Innovation

## Confused About Food Labels?

Being able to understand food labels can help you make better choices about packaged foods.

**For a quick glance see front of pack labelling (Traffic light labelling)**

Most supermarkets and food manufacturers now highlight the energy, fat, saturated fat, sugars and salt content per portion on the front of the packaging, alongside the contribution of this food (%) to the reference intake (RI) of an average adult. Be aware that the manufacturer's idea of a portion may be different from yours.

**Per biscuit**

| ENERGY | FAT | SATURATES | SUGARS | SALT |
|---|---|---|---|---|
| 257kJ 61kcal | 2.8g | 1.4g | 2.7g | 0.14g |
| 3% | 3% | 7% | 3% | 2% |

**% of the reference intakes**

Typical values Per 100g: Energy 2052kJ/490 kcal

RI= Reference Intakes of average adult (8400kJ/2000kcal)

**Some front-of-pack nutrition labels use red, amber and green colour coding.**

- red means high (Eat occasionally)
- amber means medium (Eat some of the time)
- green means low (Healthiest choice)

| All measures per 100g | Low<br>A healthier choice | Medium<br>Most of the time | High<br>Eat occasionally |
|---|---|---|---|
| Sugars | 5g or less | 5.1g – 15g | More than 15g |
| Fat | 3g or less | 3.1g – 20g | More than 20g |
| Saturates | 1.5g or less | 1.6g – 5g | More than 5g |
| Salt | 0.3g or less | 0.31g – 1.5g | More than 1.5g |

# What are reference intakes (RI)?

Reference Intakes (RI) have been set by European law. The values are maximum amounts that should be eaten in one day by an average adult. They are a good guide but they are not individual recommendations and your needs may be different to the RI, depending on your age, gender and how physically active you are.

**For more detail see back of pack labelling (Nutrition table)**

| Typical Values | Per 100g | Per biscuit | % adult RI per biscuit | RI for an average adult |
|---|---|---|---|---|
| Energy | 2052kJ | 257kJ | – | 8400kJ |
| | 490kcal | 61kcal | 3% | 2000kcal |
| Fat | 23g | 2.8g | 4% | 70g |
| of which saturates | 11g | 1.4g | 7% | 20g |
| Carbohydrate | 63g | 7.8g | 3% | |
| Of which sugars | 22g | 2.7g | 3% | 90g |
| Fibre | 3.8g | <0.5g | – | |
| Protein | 6.8g | 0.9g | 2% | |
| Salt | 1.1g | 0.14g | 2% | 6g |

RI = Reference Intakes of an average adult

This is telling you that each biscuit contains 61kcal

This is telling you that each biscuit contains 2.8g of fat, and that this contributes 4% of the average adult's maximum reference intake (RI)

# Shopping

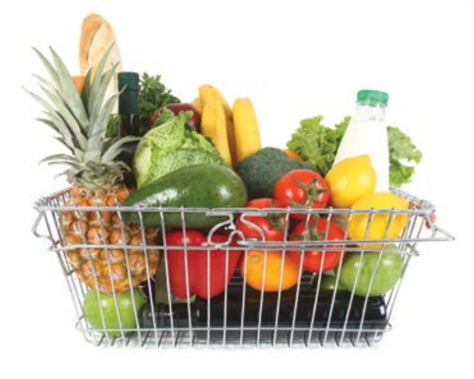

You may find shopping for food a difficult time. There are lots of temptations.

- **Plan for the week ahead.** Write a list before you go and don't buy anything that isn't on the list.
- Choose seasonal fruits and vegetables or those on special offer if you find them expensive. Remember tinned or frozen foods can be just as healthy as fresh fruit and vegetables.
- Where possible, avoid the cakes, biscuits and snack aisles or other foods you know you will find tempting.
- Bigger packets may not be a bargain in the long run, if they mean you eat more.
- Go shopping with family or a friend if you think it would help.
- Don't shop when you are hungry, stressed, tired, angry or depressed.

# Holidays and special occasions

Holidays and special occasions are a time to enjoy. However if they occur regularly, or your holiday lasts a number of days and you are not mindful, you will find that a lot of the weight you have worked so hard to lose over previous months can be re-gained very quickly. This need not be the case.

### Serving sizes

You may be sampling new foods. Remember to keep portion sizes small, especially if you are trying lots of different dishes. Listen to when your body is getting full, and try to leave some food on the plate.

### Indulgences

Holidays and celebrations would not be same if we cannot indulge in our favourite treats. Allow yourself one per day on holiday or one per occasion, so you do not restrict your enjoyment.

### Interaction

For many, the holidays and celebrations are 'food, food and more food.' Aim to shift the focus to catching up with friends and family, observing new things, meeting new people, or trying a new activity.

### Exercise

Don't let the holidays be a time to abandon your exercise plan. Take advantage of your new fitness level and the extra time you have available by going on walks, swimming or playing games.

### Alcohol

Remember that alcohol is high in calories and reduces your self control.



# Eating out

'Eating out' includes eating at the cafeteria at work, at a restaurant or café or a takeaway. If you eat out regularly (once a week or more) the choices you make are important and will affect your ability to lose weight. If you rarely eat out (once a month or less) ENJOY your favourite foods.

### Tips:

1: Choosing a starter portion as a main course means you can enjoy more courses without increasing your calories too much

2: Choose broth type soups rather than 'cream of' soups or higher fat starters

3: Choose fruit/sorbets for dessert rather than richer cakes and puddings

4: Choose tomato based sauces rather than cream or butter based sauces

5: Ask for your dressing on the side so you can control how much you have

6: At buffets or BBQs take smaller portions of high fat foods, fill up on the healthy options available

7: Restrict the amount of alcohol you drink; it has calories and lowers your self-control.

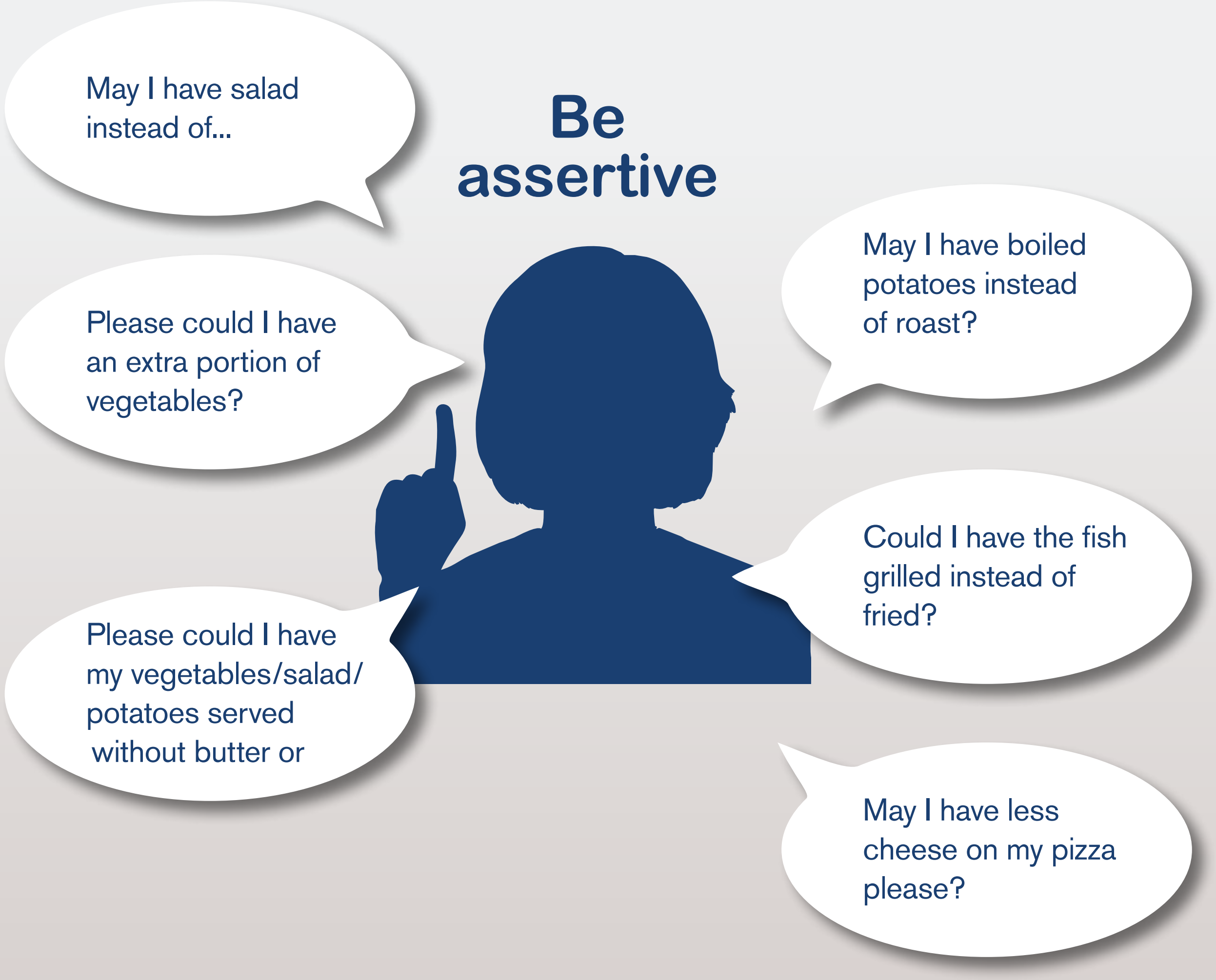

### Eating out exercise

| What are your favourite takeaways and places to eat out? |
|---|

| How can you improve your choices? |
|---|

**Collect some menus from restaurants or take-away venues you use. Try and pick out the healthier options and be prepared to discuss them on your next phone call.**



# Dealing with pressures to eat

There will always be occasions when it may be more difficult for you to stick to your healthy eating plan. It could be holidays, parties, or even a hard day at work.

### The problem solving approach

These situations are challenges and it can be helpful to take a '*problem solving approach*' to these situations.

**The problem solving approach has 3 elements:**
1. Stop and think about the problem
2. Look at your options
3. Resolve to respond positively and assertively

### Example:

You come home from work and you find that your partner has left a plate of chocolate biscuits out in the kitchen. You feel annoyed and upset at his/her thoughtlessness. You feel tempted to eat them…

| | |
|---|---|
| **1. Stop and think about the problem** | When you take a step back and think, you realise you have several options |
| **2. Look at your options** | You could:<br>• Eat the biscuits<br>• Throw the biscuits out<br>• Put the biscuits in the container<br>• Go and talk to your partner<br>Think carefully how each of these responses would make you feel. |
| **3. Resolve to respond positively and assertively** | This means an honest and appropriate expression of your feelings while acknowledging other people's right to fairness and respect. For instance, in the example given above, you could:<br>• Tell your partner that it is easier for you, if he/she puts the biscuits away.<br>• Explain to your partner that you would find it easier to keep to the eating plan if there were no biscuits in the house and that you are going to stop buying chocolate biscuits altogether. |

**Pressure to eat from other people**

You will come across times when other people encourage you to eat. It's a normal behaviour to share or offer food.

They may think they're being helpful, or they're uncomfortable eating alone in front of you or even that they want to test your determination.

The most important thing for you to do is to remind yourself that you are doing this for yourself and the reasons you want to lose weight.

Try to be prepared for situations where you will be offered food and have in mind how you will respond.

**No thanks, I've just eaten**

**No thanks, I'm not hungry at the moment**

**It looks delicious, but I don't want to spoil my dinner**

**I won't have it now, can I take some home?**

Think about some of your own experiences in dealing with pressures to eat and how you have responded in the past. How might you deal with these situations now?

| | How I may have responded in the past | How I could I respond in future? |
|---|---|---|
| **Example 1** Tempting food at a buffet | Starved myself all day, so felt I deserved to eat anything I liked – over ate and felt very full afterwards. I felt that I had sabotaged my diet. | Eat normally before going out. Survey choice of food available at buffet, go for healthier choices, and move away from the table. |
| **Example 2** A friend eats in front of you | | |
| **Example 3** | | |
| **Example 4** | | |



# Thought patterns

All our actions begin with a thought about the action. Losing weight involves many new thoughts as we try to establish new healthier habits.

Sometimes people give themselves too hard a time about trying to lose weight and set impossible targets and deadlines. This can lead to you feeling unhappy with yourself and your abilities to achieve change of any kind. You fall into the trap of expecting too much too soon. This type of thought process is called an 'attitude trap'.

## Unhelpful Thought

**Black and white thinking:**
"Either I stick to my eating plan completely or I have blown it altogether and may as well give up"

**Catastrophising:**
"Oh no. I've blown it again. I had a second helping so all my hard work this week has been a waste of time"

**Over-generalisation:**
"I ate that big bag of crisps. I never have any willpower"

**Minimising the good things:**
"I've had a good day today, but it won't last. I know what I am like"

**Magnifying the bad things:**
"I ate that pudding. That's so typical of me"

## Helpful Thought

"I will try and stick to my eating plan as best I can, but expect the occasional slip".

"I have had a temporary lapse. The most important thing is that I try and get back on track, then this will not affect my overall progress"

"I ate that bag of crisps. I find them very hard to resist. Next time I will include crisps in my weekly eating plan"

"I've had a good day today. I am really happy with my progress"

"I ate that pudding. This is my first lapse all week so overall I have done well"

Recognising your negative thought processes is the first step in learning how to change them for the better. Here are some words which are common pitfalls when trying to make goals or targets. Think about your unhelpful thoughts and how you could change them into being more helpful ones.

## Unhelpful Thought

**I must not…**
**I must…**
**I will always…**
**I will never…**

## Helpful Thought

**I will try and avoid…**
**My goal is to…**
**I will try to…**
**I will try not too…**

This slight alteration in the way you think can seem very obvious but it will really help when you look back on what you have been able to achieve.

# Visit 5: Personal Target Setting

| Date | Weight | Waist Circumference |
| --- | --- | --- |
| | | |

| Targets Set |
| --- |
| 1 |
| 2 |
| 3 |
| 4 |
| 5 |
| 6 |



**You may find it useful to ask yourself the following questions to help you achieve your targets:**

How will I go about achieving this target? How will I know if I have met this target? Is it realistic for me to achieve this target in the amount of time I have available?

| Targets Met |
| --- |
| 1 |
| 2 |
| 3 |
| 4 |
| 5 |
| 6 |

## Notes



## Notes

i HEALTH T 2 D

Horizon 2020
European Union Funding
for Research & Innovation

**Designed by ESP Bioscience 2016**
**http://www.espstc.com**
