# Supplementary material for: The iHealth-T2D study, prevention of type 2 diabetes amongst South Asians with central obesity and prediabetes: study protocol for a randomised controlled trial
Source: Trials. 2021 Dec 18;22:928. doi: 10.1186/s13063-021-05803-7 (PMC8684177; doi:10.1186/s13063-021-05803-7)
Supplement: Supplementary file 1 — Additional file 1: S1. CHW Handbook. Training manual summarising the content and delivery of the lifestyle intervention. Translated versions available through study website. S2. CHW Workbook. Case report form / intervention delivery workbook for the lifestyle intervention. Translated versions available through study website. S3. Participant Handbook for Lifestyle intervention. Written materials provided to the participant to support the CHW delivered lifestyle intervention. Translated versions available through study website. S4. Participant Handbook for Usual care. Translated versions available through study website. Supplementary Table 1: The a-priori power calculations under alternate assumptions with a sample size of 3,600 participants. [file 13063_2021_5803_MOESM1_ESM.zip › Addcorr2.zip!/S3 Participant booklet.zip!/21526 Clinic visit 8 V3 England_4.pdf]

i HEALTH
T 2 D

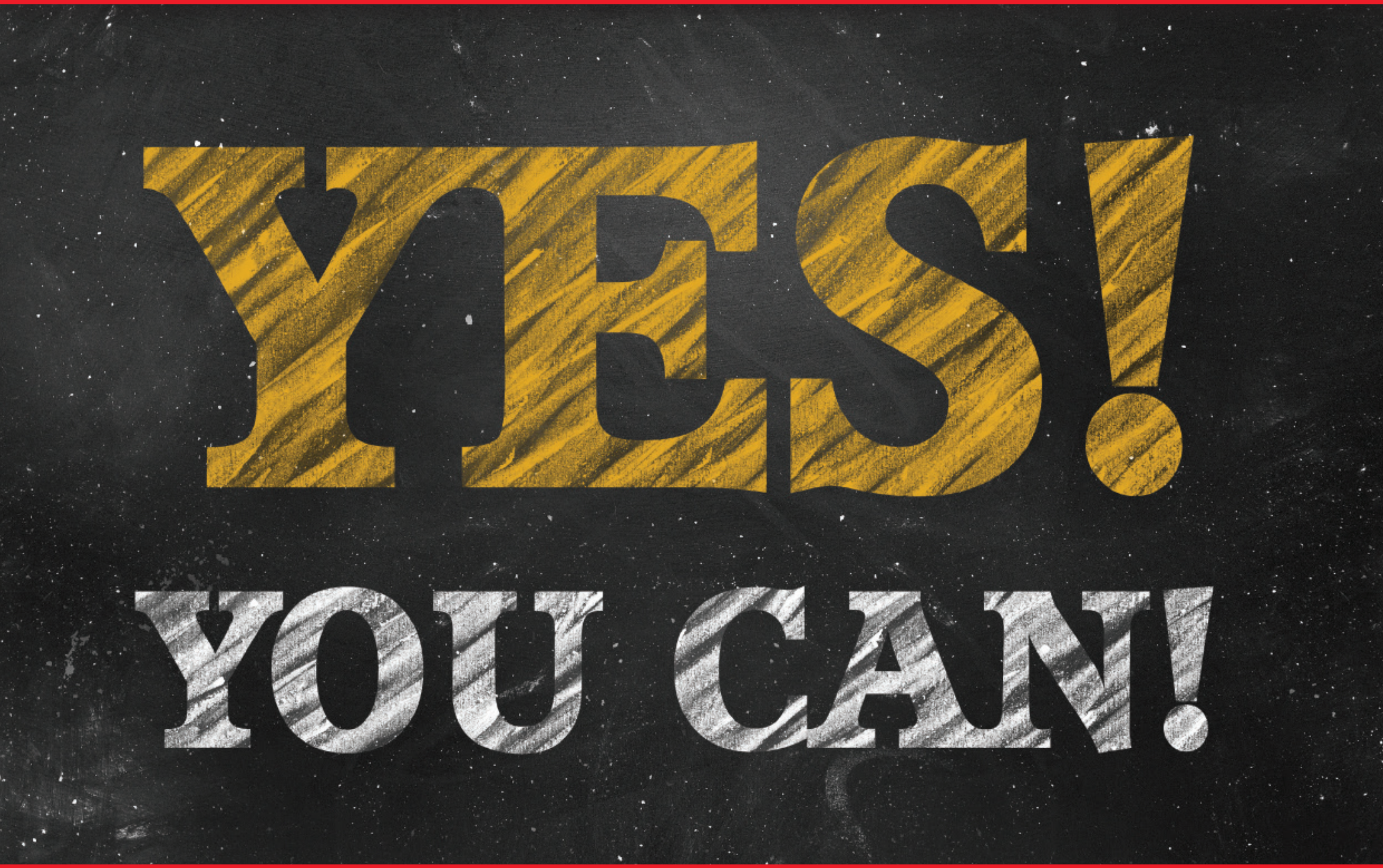

Visit 8

Review

Horizon 2020
European Union Funding
for Research & Innovation

	Visit 7	Visit 8
**Weight**		
**BMI**		
**Waist circumference**		

List some of the lifestyle changes you have made since visit 7:

List some of the changes you found easiest to achieve:

List some things you have found most difficult to do but achieved them anyway:

What obstacles or difficulties do you foresee in the months to come and how might you deal with them?

Visit 8: Personal Target Setting

Date	Weight	Waist Circumference

You may find it useful to ask yourself the following questions to help you achieve your targets:

How will I go about achieving this target? How will I know if I have met this target? Is it realistic for me to achieve this target in the amount of time I have available?

Targets Set
1
2
3
4
5
6

Targets Met
1
2
3
4
5
6

i HEALTH
T 2 D

Horizon 2020
European Union Funding
for Research & Innovation

Designed by ESP Bioscience 2016
http://www.espstc.com
