# Supplementary material for: The iHealth-T2D study, prevention of type 2 diabetes amongst South Asians with central obesity and prediabetes: study protocol for a randomised controlled trial
Source: Trials. 2021 Dec 18;22:928. doi: 10.1186/s13063-021-05803-7 (PMC8684177; doi:10.1186/s13063-021-05803-7)
Supplement: Supplementary file 1 — Additional file 1: S1. CHW Handbook. Training manual summarising the content and delivery of the lifestyle intervention. Translated versions available through study website. S2. CHW Workbook. Case report form / intervention delivery workbook for the lifestyle intervention. Translated versions available through study website. S3. Participant Handbook for Lifestyle intervention. Written materials provided to the participant to support the CHW delivered lifestyle intervention. Translated versions available through study website. S4. Participant Handbook for Usual care. Translated versions available through study website. Supplementary Table 1: The a-priori power calculations under alternate assumptions with a sample size of 3,600 participants. [file 13063_2021_5803_MOESM1_ESM.zip › Addcorr2.zip!/S3 Participant booklet.zip!/21526 Clinic visit 9 V3 England_3.pdf]

i HEALTH
T 2 D

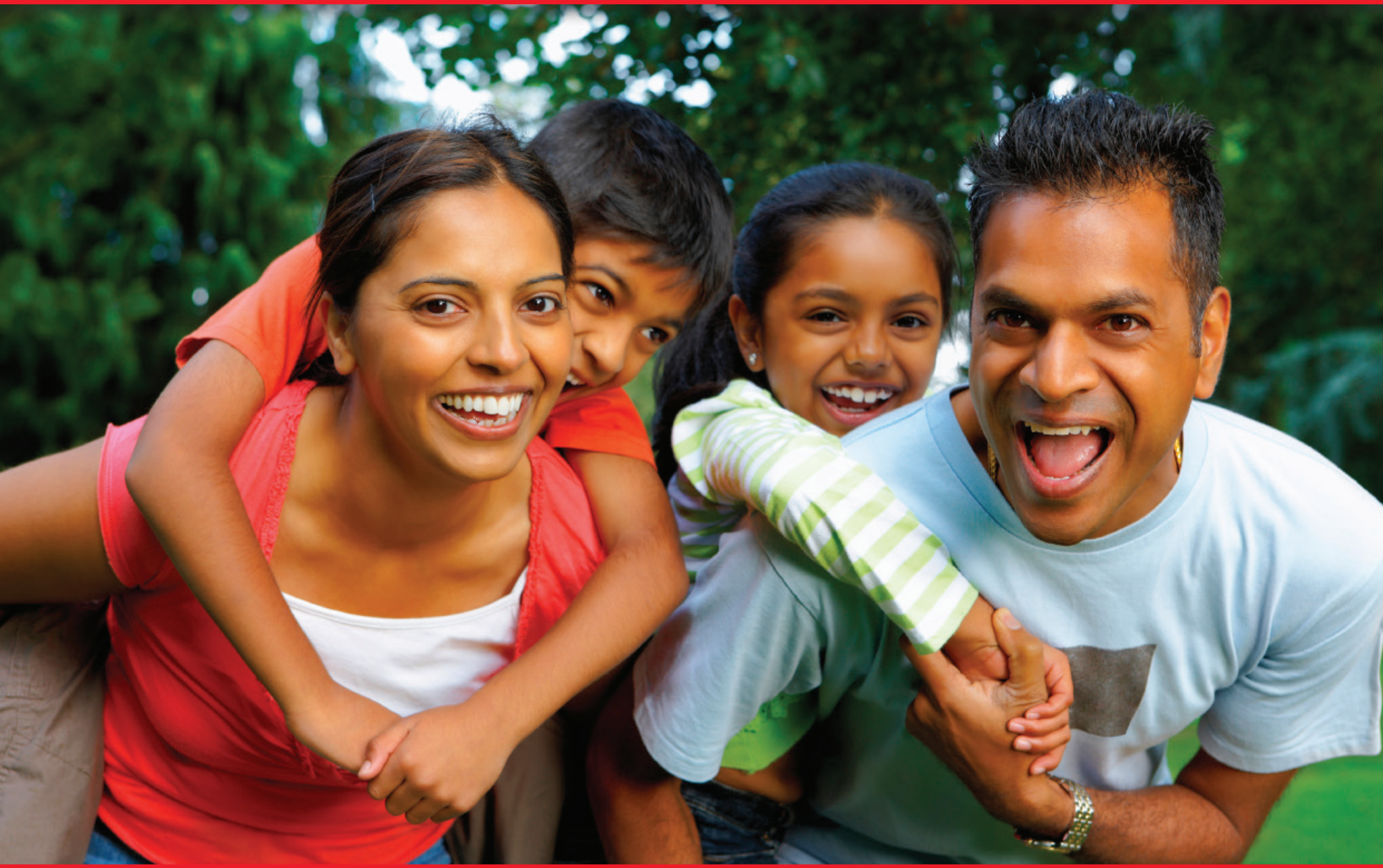

Visit 9

Review

Horizon 2020
European Union Funding
for Research & Innovation

Review

At this visit we will review your achievements and discuss how you would like to proceed.

You may decide to continue to lose weight or to maintain the weight that you have already lost.

Now is the time to look back over your progress, to recognise what you have achieved and to discuss what you may find difficult in the months to come as there are no further appointments with your CHW.

	Visit 1	Visit 9
Weight		
BMI		
Waist circumference		

List some of the lifestyle changes you have made since visit 1:

List some of the changes you found easiest to achieve:

List some things you have found most difficult to do:

Importance of weight loss

How **important** is it for you to lose/maintain weight in the next 6 months? (please mark from 1-10)

1	2	3	4	5	6	7	8	9	10
Not important at all		Fairly important			important				Very important

Confidence

How **confident** are you that you can lose weight to in the next 6 months? (please mark from 1-10)

1	2	3	4	5	6	7	8	9	10
Not confident at all		Fairly confident			confident				Very confident

What obstacles or difficulties do you foresee in the months to come and how might you deal with them?

My Eating Plan

The energy prescription that has been calculated for me allows me to have ______ Kcal per day.

This allows me a certain number of 'servings' per day from the 5 food groups.

______ Servings of Bread, chapatti and alternatives

______ Servings of Milk and dairy foods

______ Servings of Meat, fish and dahls

______ Servings of Fats and oils

______ Servings of Fruit and vegetables

Visit 9: Personal Target Setting

Date	Weight	Waist Circumference

Targets Set
1
2
3
4
5
6

You may find it useful to ask yourself the following questions to help you achieve your targets:

How will I go about achieving this target? How will I know if I have met this target? Is it realistic for me to achieve this target in the amount of time I have available?

Targets Met
1
2
3
4
5
6

Horizon 2020
European Union Funding
for Research & Innovation

Designed by ESP Bioscience 2016
http://www.espstc.com
